# Using biological information to analyze potential miRNA-mRNA regulatory networks in the plasma of patients with non-small cell lung cancer

**DOI:** 10.1186/s12885-022-09281-1

**Published:** 2022-03-21

**Authors:** Wei Zhang, Qian Zhang, Li Che, Zhefan Xie, Xingdong Cai, Ling Gong, Zhu Li, Daishun Liu, Shengming Liu

**Affiliations:** 1 Department of Pulmonary and Critical Care Medicine, The First Affiliated Hospital of Jinan University, No. 613, Huangpu Road West, Tianhe District, Guangzhou, 510630 China; 2 Department of Pulmonary and Critical Care Medicine, The Third Affiliated Hospital of Zunyi Medical University (The First People’s Hospital of Zunyi), No. 98, Fenghuang Road North, Zunyi, 563000 Guizhou China; 3 Department of Renal Medicine, The First Affiliated Hospital of Jinan University, No. 613, Huangpu Road West, Tianhe District, Guangzhou, 510630 China

**Keywords:** Non-small cell lung cancer, microRNA, Bioinformatics, miRNA-mRNA regulatory network

## Abstract

**Background:**

Lung cancer is the most common malignant tumor, and it has a high mortality rate. However, the study of miRNA-mRNA regulatory networks in the plasma of patients with non-small cell lung cancer (NSCLC) is insufficient. Therefore, this study explored the differential expression of mRNA and miRNA in the plasma of NSCLC patients.

**Methods:**

The Gene Expression Omnibus (GEO) database was used to download microarray datasets, and the differentially expressed miRNAs (DEMs) were analyzed. We predicted transcription factors and target genes of the DEMs by using FunRich software and the TargetScanHuman database, respectively. The Database for Annotation, Visualization, and Integrated Discovery (DAVID) was used for GO annotation and KEGG enrichment analysis of downstream target genes. We constructed protein-protein interaction (PPI) and DEM-hub gene networks using the STRING database and Cytoscape software. The GSE20189 dataset was used to screen out the key hub gene. Using The Cancer Genome Atlas (TCGA) and UALCAN databases to analyze the expression and prognosis of the key hub gene and DEMs. Then, GSE17681 and GSE137140 datasets were used to validate DEMs expression. Finally, the receiver operating characteristic (ROC) curve was used to verify the ability of the DEMs to distinguish lung cancer patients from healthy patients.

**Results:**

Four upregulated candidate DEMs (hsa-miR199a-5p, hsa-miR-186-5p, hsa-miR-328-3p, and hsa-let-7d-3p) were screened from 3 databases, and 6 upstream transcription factors and 2253 downstream target genes were predicted. These genes were mainly enriched in cancer pathways and PI3k-Akt pathways. Among the top 30 hub genes, the expression of *KLHL3* was consistent with the GSE20189 dataset. Except for let-7d-3p, the expression of other DEMs and *KLHL3* in tissues were consistent with those in plasma. LUSC patients with high let-7d-3p expression had poor overall survival rates (OS). External validation demonstrated that the expression of hsa-miR-199a-5p and hsa-miR-186-5p in peripheral blood of NSCLC patients was higher than the healthy controls. The ROC curve confirmed that the DEMs could better distinguish lung cancer patients from healthy people.

**Conclusion:**

The results showed that miR-199a-5p and miR-186-5p may be noninvasive diagnostic biomarkers for NSCLC patients. MiR-199a-5p-KLHL3 may be involved in the occurrence and development of NSCLC.

**Supplementary Information:**

The online version contains supplementary material available at 10.1186/s12885-022-09281-1.

## Background

Lung cancer is the most common malignant tumor, and it is the main cause of cancer-related death worldwide. In 2018, about 2.09 million people developed lung cancer, and nearly 1.8 million people died of lung cancer [1]. In China, lung cancer is the leading tumor in terms of male morbidity, male mortality, and female mortality, and it is the second leading tumor in terms of female morbidity [2]. Non-small cell lung cancer (NSCLC) is the most common type of lung cancer, accounting for about 85% of lung cancer cases [3]. The main subtypes of NSCLC are lung adenocarcinoma (LUAD), lung squamous cell carcinoma (LUSC), and large cell carcinoma [3]. The pathogenesis of NSCLC is complex and involves genetic and immunologic changes. For instance, when tumor oncogenes are upregulated or when tumor suppressor genes are downregulated, the downstream signaling pathways of the genes are activated or inhibited, respectively. On the one hand, the migration, invasion, and proliferation abilities of the tumor cells are promoted; on the other hand, the tumor cells develop resistance to anti-tumor drugs [4]. Patients with early-stage NSCLC usually have no obvious symptoms; therefore, most patients have stage III or IV NSCLC at the time of diagnosis, which seriously affects their quality of life and subsequent treatment [4, 5]. With continuous medical technology advancements, new treatment methods, such as tumor immunotherapy and targeted therapy, have been widely developed in clinical settings. Moreover, precise and individualized treatment plans for tumors are gradually maturing. However, due to the lack of early diagnostic markers, NSCLC patients often miss the best opportunity for early treatment, and their 5-year survival rate is only 23% [6]. Therefore, identifying markers for the diagnosis and treatment of lung cancer has important clinical significance when it comes to improving the rate of early NSCLC diagnosis and selecting appropriate medical treatments.

MicroRNAs (miRNAs) are very short noncoding RNAs comprising about 20–24 nucleotides. MiRNAs were discovered in the research of *Caenorhabditis elegans* in 1993 [7]. There are obviously conserved miRNA sequences, which play vital roles in the regulatory pathways between mononuclear and multinuclear eukaryotes [7, 8]. MiRNA specifically binds to the 3′ untranslated region of target gene messenger RNA (mRNA) through base complementary pairing; it thus regulates the expression of target genes and participates in the regulation of cell migration, invasion, proliferation, apoptosis, and other biological processes [9]. A variety of miRNAs play important roles in the occurrence and development of lung cancer. By combining with corresponding target genes and regulating their expression levels, miRNAs can function as tumor suppressor genes or oncogenes to regulate the biological processes of lung cancer [10–15]. Previous studies have found that miRNAs can be stably expressed in blood, and different diseases have a distinct serum-miRNA profile [16]. Mitchell et al. found that many tumor-derived miRNAs existed in the peripheral blood of mice with prostate cancer xenograft models [17]. The expression of miRNAs in the blood may be related to miRNAs released by tumor cells into the surrounding environment [18]. Therefore, miRNAs in the circulation may provide new insight for the diagnosis and treatment of lung cancer. Leng et al. found that plasma miRNA biomarkers may be helpful for the early diagnosis of lung cancer and the classification of lung cancer subtypes [19]. They also found that some miRNAs (miR-126, miR-145, miR-210, and miR-205-5p) in plasma have high sensitivity and specificity when it comes to the early diagnosis of lung cancer and are independent of the stage and histological type of lung tumor as well as the age, sex, and race of the patient. Plasma miRNA signals may provide a blood-based detection method for the early diagnosis of lung cancer, thereby reducing related patient mortality and economic costs [19, 20]. Szczyrek et al. found that the expression levels of miR-27a-3p, miR-31, miR-182, and miR-195 in the plasma of lung cancer patients are different from those in the plasma of healthy people; and the detection of these miRNAs in the plasma can be useful in the noninvasive diagnosis of lung cancer [21]. Therefore, actively searching for miRNAs and mRNAs closely related to the occurrence of NSCLC can provide new targets for the diagnosis and treatment of the disease, which in turn may improve the prognosis of the disease.

Although many studies have reported on the expression levels and functions of miRNAs in lung cancer, there are still few studies regarding the miRNA-mRNA regulatory networks in the plasma of lung cancer patients. Therefore, the purpose of this study was to establish a potential miRNA-mRNA regulatory network in the plasma of NSCLC patients through corresponding analysis in order to provide new targets for the diagnosis and treatment of lung cancer.

## Methods

### Microarray data information

In the Gene Expression Omnibus (GEO) database (https://www.ncbi.nlm.nih.gov/geo/), the screening of miRNA datasets related to lung cancer plasma was accomplished by using the terms “lung cancer” (research keyword), “non-coding RNA profile” (research type), and “*Homo sapiens*” (organism). Three miRNA datasets (GSE24709 [22], GSE31568 [23], and GSE61741 [24]) were selected for subsequent analysis. These datasets were all based on the GPL9040 platform (febit *Homo Sapiens* miRBase 13.0). The GSE24709 dataset included 47 samples (28 lung cancer samples and 19 healthy controls), the GSE31568 dataset included 102 samples (32 lung cancer samples and 70 healthy controls), and the GSE61741 dataset included 167 samples (73 lung cancer samples and 94 healthy controls). The details of the 3 datasets are shown in Table 1.

**Table 1:** Details of lung cancer data in GEO datasets

Accession	Platform	Sample	Normal	Lung cancer	Gene/microRNA
GSE24079	GPL9040	Blood	19	28	microRNA
GSE31568	GPL9040	Blood	70	32	microRNA
GSE61741	GPL9040	Blood	94	73	microRNA
GSE20189	GPL571–17391	Blood	81	81	gene
GSE17681	GPL9040	Blood	19	17	microRNA
GSE137140	GPL21263	Blood	2178	1746	microRNA

### Screening of DEMs

**Fig. 1:**
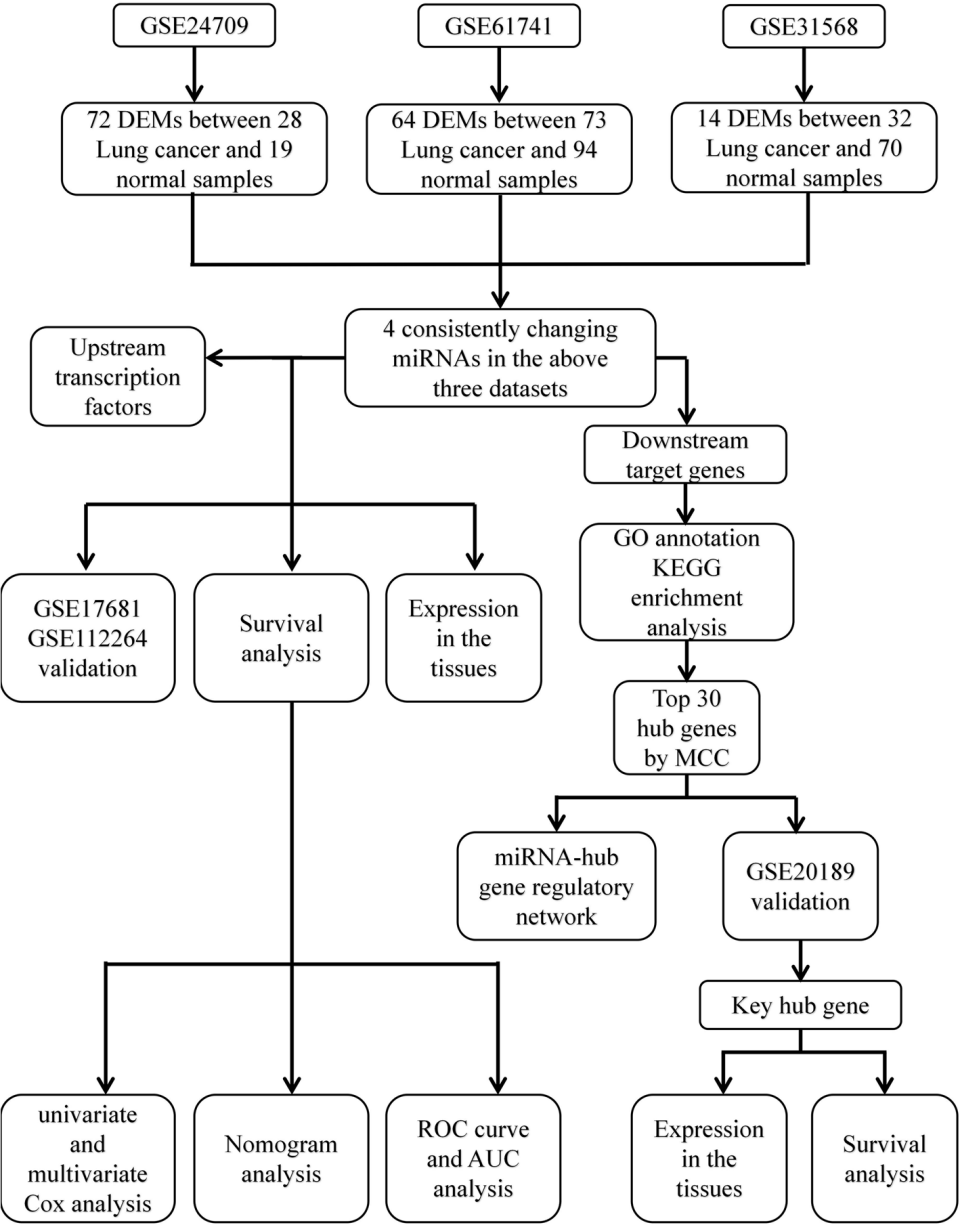
Flow chart of the construction of the miRNA-mRNA network of lung cancer patients

The miRNA gene names within the datasets were obtained by using the R software (3.6.1) and relevant annotation packages. The limma package (3.40.6) was used to compare and screen differentially expressed miRNAs (DEMs) between lung cancer patients and healthy groups and to make corresponding thermograms and volcano maps. Adjusted *P*-values were obtained by using the Benjamini-Hochberg false discovery rate method. The adjusted *P*-values were used to correct the occurrence of false positives. Adjusted *P*-values < 0.05 and |log fold change| values > 1 were set as the threshold for identifying DEMs within the 3 datasets (GSE24709, GSE61741, and GSE31568; Tables S1, S2 and S3). A Venn diagram was used to analyze DEM overlap within the 3 datasets, and the overlapping miRNAs were selected as candidate DEMs. The research and design process is shown in Fig. 1.

### Forecasting upstream transcription factors of DEMs

FunRich (http://www.funrich.org/) is an independent software tool that is mainly used to analyze the functional enrichment of genes and proteins and to perform interaction network analyses. It was used to predict the potential upstream transcription factors of the candidate DEMs. *P*-values < 0.05 were considered statistically significant.

### Forecasting downstream target genes of DEMs

The online database TargetScanHuman Release 7.2 (http://www.targetscan.org/vert_72/) was used to predict the biological targets of the miRNAs (Table S4), including targets with conserved sites, as well as targets irrespective of conserved sites.

### GO and KEGG analyses of target genes

To study the biological functions of the target genes of the DEMs, the online tool Database for Annotation, Visualization, and Integrated Discovery (DAVID; https://david.ncifcrf.gov/) was used for Gene Ontology (GO) and Kyoto Encyclopedia of Genes and Genomes (KEGG) pathway analyses.

### Building the PPI network and screening hub genes

The Search Tool for the Retrieval of Interacting Genes/Proteins (STRING) online database (https://string-db.org/) was used to construct the protein-protein interaction (PPI) network of the target genes of the DEMs. The network was then visualized by using Cytoscape software (version 3.6.1). By using the cytoHubba plug-in, the nodes in the preloaded PPI network were sorted according to several topology algorithms and their features. The Maximal Clique Centrality (MCC) method was used to select the hub genes, which were considered to be the top 30 nodes of the PPI network, as this method has good performance when it comes to accurately predicting essential proteins [25].

### Analysis of hub gene expression in the GSE20189 dataset

Since there is no other data on mRNA expression in the plasma of lung cancer patients, the GSE20189 dataset [26] was downloaded from the GEO database in order to analyze hub gene expression levels. This dataset, based on the GPL571 platform (Affymetrix Human Genome U133A 2.0 Array), contained 81 lung cancer patient plasma samples and 81 normal patient plasma samples. Student’s t-test was used to identify differentially expressed genes in the lung cancer and normal plasma samples. The key hub gene had to meet the following conditions: first, the upregulated target genes of the DEMs were downregulated or the downregulated target genes were upregulated; second, the *P*-value was < 0.05.

### Verifying the expression levels of the key hub gene and DEMs in tissues and performing the survival analysis

The University of Alabama Cancer (UALCAN) database (http://ualcan.path.uab.edu) is a website for the online analysis and mining of datasets from The Cancer Genome Atlas (TCGA). It was used to analyze the expression levels of the key hub gene and DEMs in LUAD and LUSC tissues as well as the different stages of LUAD and LUSC, and to analyze the overall survival (OS) associated with the expression levels of these hub genes and DEMs in patients with lung cancer. A *P*-value < 0.05 was considered statistically significant.

The raw counts of the RNA sequencing data of the key hub gene and DEMs (Supplementary Tables S5, S6, S7, S8, S9, S10, S11, S12, S13, S14) and the clinical information of NSCLC patients (Supplementary Table S15) were obtained from the TCGA dataset (https://portal.gdc.cancer.gov/). The Kaplan-Meier survival analysis with the log-rank test was used to compare the 2 groups’ progression-free survival (PFS). All of the analytical methods described above were performed using R software. A *P*-values < 0.05 were considered statistically significant.

Subsequently, univariate and multivariate Cox analyses were performed to evaluate the proportional hazards model using the survival package of the R software. Variables included age, sex, and tumor stage. Nomogram and receiver operating characteristic (ROC) curves were used to evaluate the impact of DMEs on patient’s prognosis.

### Verifying the translation levels of the key hub gene

The Human Protein Atlas (HPA) database (https://www.proteinatlas.org/) provides information regarding the distribution of human proteins in tissues and cells. The expression of target protein in tissues is characterized by the annotations *Intensity* and *Quantity*. According to the previous study, we transformed the four values (Strong, Moderate, Weak, and Negative) that used to describe *Intensity* into 3,2,1, and 0, respectively; and also transformed the five values (>75, 75–25%, <25%, Rare, and Negative) that used to describe *Quantity* into 75,50,25,5, and 0, respectively [27]. *I* and *Q* expressed the transformed *Intensity* and *Quantity*. The expression of the hub gene in NSCLC tissues and normal tissues was calculated using *I* × *Q* (Tables S16, S17, S18) [27]. Student’s t-test and Wilcoxon test were used to compare the expression of key genes in LUAD and normal tissues, and LUSC and normal tissues, respectively. A *P*-values < 0.05 were considered statistically significant.

### External validation and efficacy evaluation

The GSE17681 [28] and GSE137140 [29] datasets were used for external validation data to verify DEMs expression in the peripheral blood of lung cancer patients and healthy controls. The GSE17681 dataset, based on the GPL9040 platform, included 17 lung cancer samples and 19 control samples, and the GSE137140 dataset, based on the GPL21263 platform, included 1746 lung cancer samples and 2178 control samples. GraphPad Prism 8.0 software was used to calculate ROC curves to assess the ability of the DEMs to distinguish between lung cancer patients and healthy people. The student’s t-test was used to compare the expression of 4 DEMs in blood of lung cancer patients and healthy groups. A *P*-values < 0.05 were considered statistically significant.

## Results

### Identification of DEMs in the plasma of lung cancer patients

**Fig. 2:**
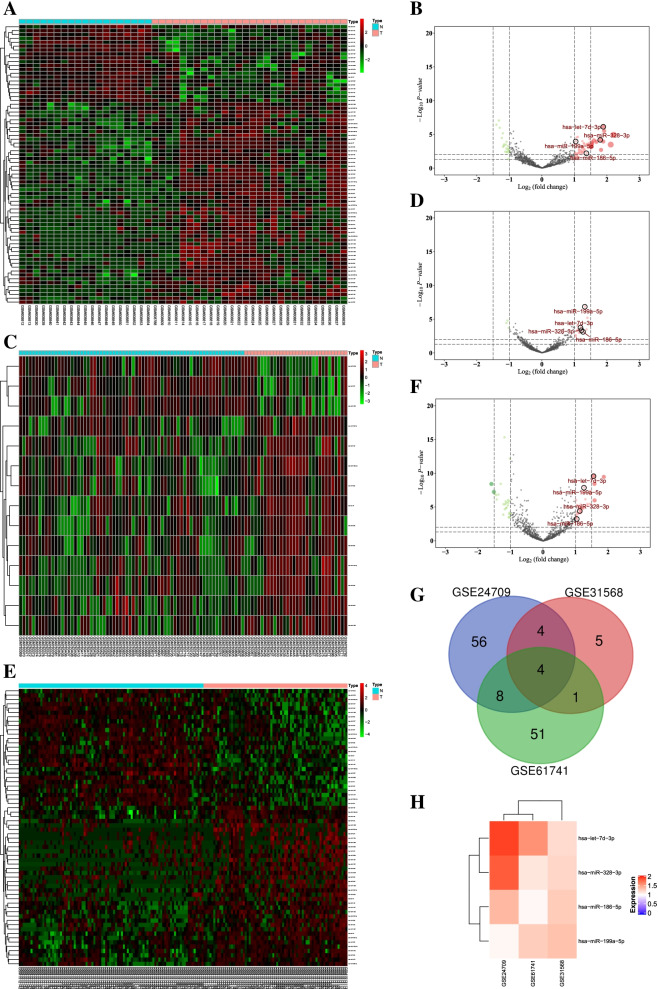
Heat and volcano maps of DEMs in lung cancer and normal plasma samples. **A**, **B**. GSE24709 dataset. **C, D**. GSE31568 dataset. **E, F**. GSE61741 dataset. Red indicates higher expression, and green indicates lower expression. **G**. Venn diagram of the expression levels of the DEMs in the 3 datasets. **H**. Log fold change heat map of the candidate DEMs

After screening with adjusted *P*-values < 0.05 and |log fold change| values > 1, a total of 72 DEMs were found in the GSE24709 dataset, of which 52 were upregulated and 20 were downregulated; 14 DEMs were found in the GSE31568 dataset, of which 11 were upregulated and 3 were downregulated; and 64 DEMs were found in the GSE61741 dataset, of which 37 were upregulated and 27 were downregulated. The heat and volcano maps of these data are shown in Fig. 2A-F. Through observation of Venn diagram intersections, 4 upregulated DEMs (hsa-miR-199a-5p, hsa-miR-328-3p, hsa-miR-186-5p, and hsa-let-7d-3p) were found to overlap between the 3 datasets (Fig. 2G, H).

### Prediction of upstream transcription factors and downstream target genes of the DEMs

**Fig. 3:**
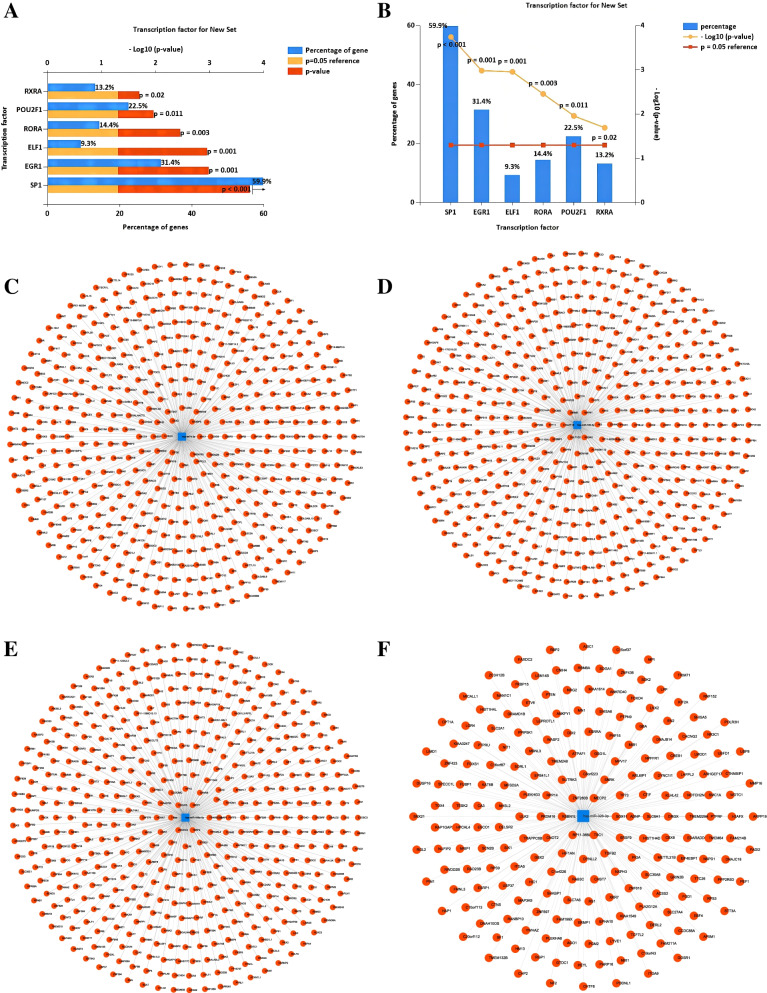
The upstream transcription factors and downstream target genes of the DEMs. **A-B**. FunRich prediction of potential upstream transcription factors of the candidate DEMs. **C-F**. miRNA-target genes network diagram of the 4 DEMs. **C**. hsa-let-7d-3p. **D**. hsa-miR-186-5p. **E**. hsa-miR-199a-5p. **F**. hsa-miR-328-3p

The FunRich software was used to predict the upstream transcription factors of the 4 candidate upregulated DEMs. Specificity protein 1 (SP1), early growth response protein 1 (EGR1), POU domain class 2 transcription factor 1 (POU2F1), RXR-alpha (RXRA), ROR-alpha (RORA), and E74-like factor1 (ELF1) were considered to potentially be involved in regulating the expression of the candidate DEMs (Fig. 3A, B). Further, a total of 2253 potential downstream target genes of the DEMs were predicted by using the TargetScanHuman 7.2 database (Fig. 3C-F). The number of target genes corresponding to each DEM is listed in Table 2.

**Table 2:** Potential Target Genes of the DEMs

DEMs	Number
hsa-miR-199a-5p	621
hsa-let-7d-3p	479
hsa-miR-328-3p	204
hsa-miR-186-5p	949
Total	2253

### GO and KEGG enrichment analyses of the downstream target genes of the DEMs

**Fig. 4:**
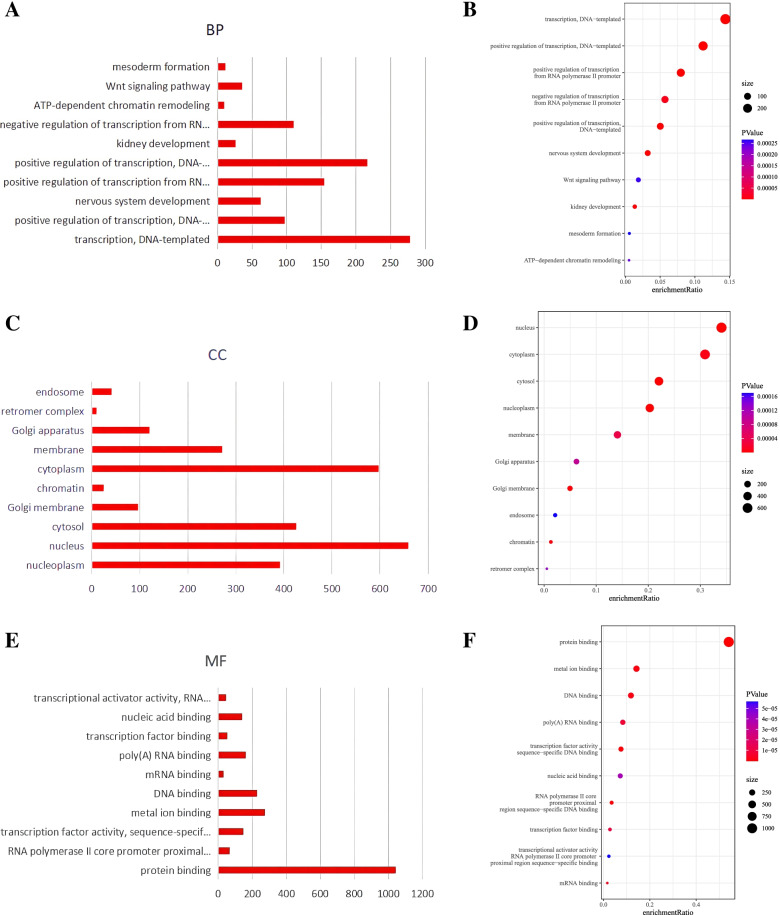
GO annotation analysis of the DEMs target genes in biological processes, cell components, and molecular functions. **A, C, E**. Bar plots. **B, D, F**. Bubble charts

**Fig. 5:**
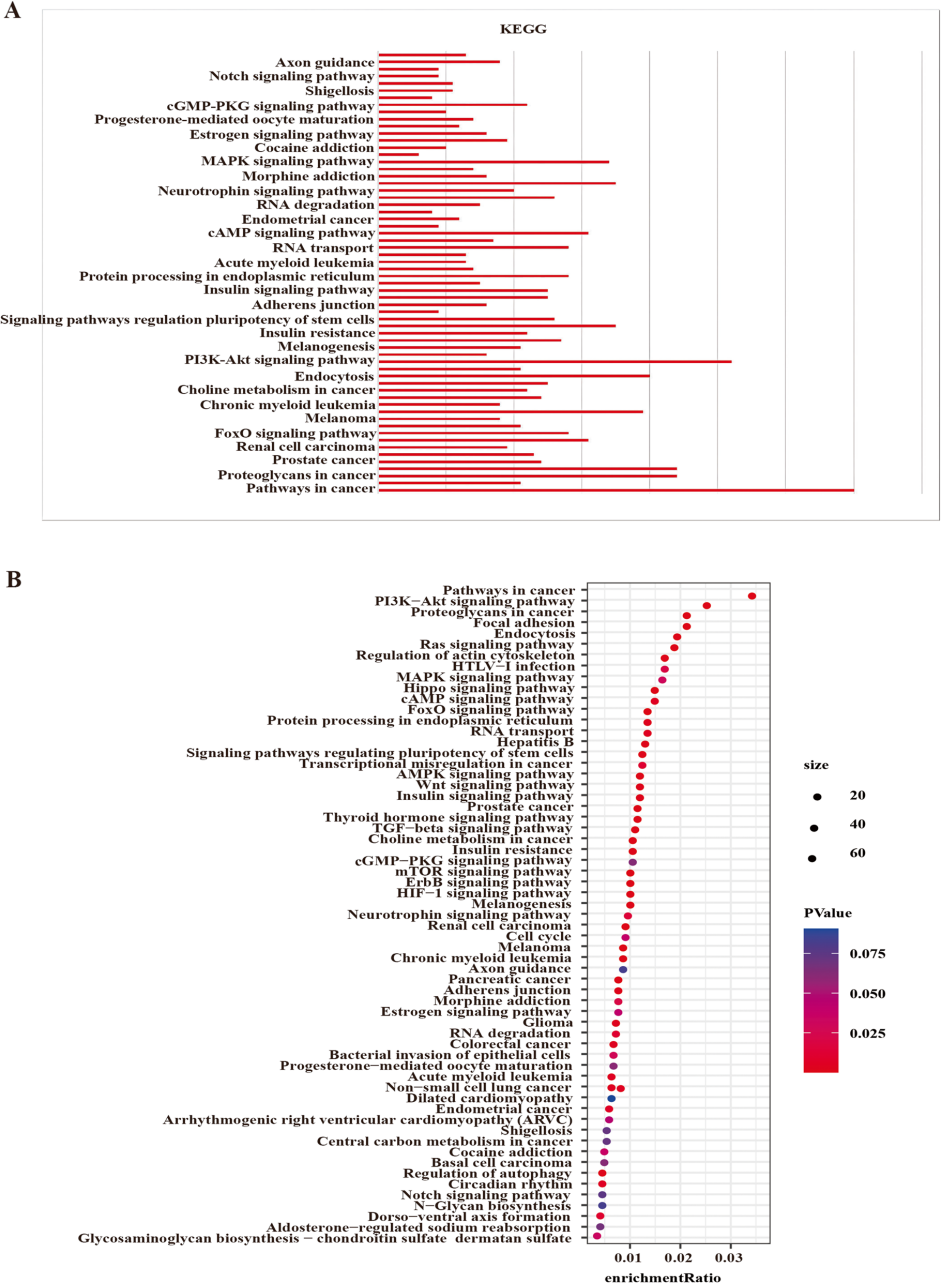
KEGG pathway enrichment analysis of the DEMs target genes. **A**. Bar plots. **B**. Bubble charts

The DAVID database was used to perform GO and KEGG enrichment analyses of the 2253 target genes of the DEMs. As shown in Fig. 4A-F, biological process analysis results showed that the target genes were mainly concentrated in transcription, positive regulation of transcription, positive regulation of transcription from RNA polymerase II promoter, and negative regulation of transcription from RNA polymerase II promoter. Additionally, cell component analysis results showed that the target genes were mainly enriched in the nucleus, cytoplasm, and nucleoplasm. Furthermore, molecular function analysis results showed that the target genes were enriched in protein binding, metal ion binding, DNA binding, poly(A) RNA binding, and transcription factor activity sequence-specific DNA binding. KEGG pathway analysis results showed that the target genes were significantly enriched in cancer pathways, PI3K-Akt signaling pathways, proteoglycans, focal adhesions, endocytosis, Ras signaling pathways, actin cytoskeleton regulation, HTLV-I infection, and MAPK signaling pathways (Fig. 5A, B).

### PPI and DEM-hub gene networks

**Fig. 6:**
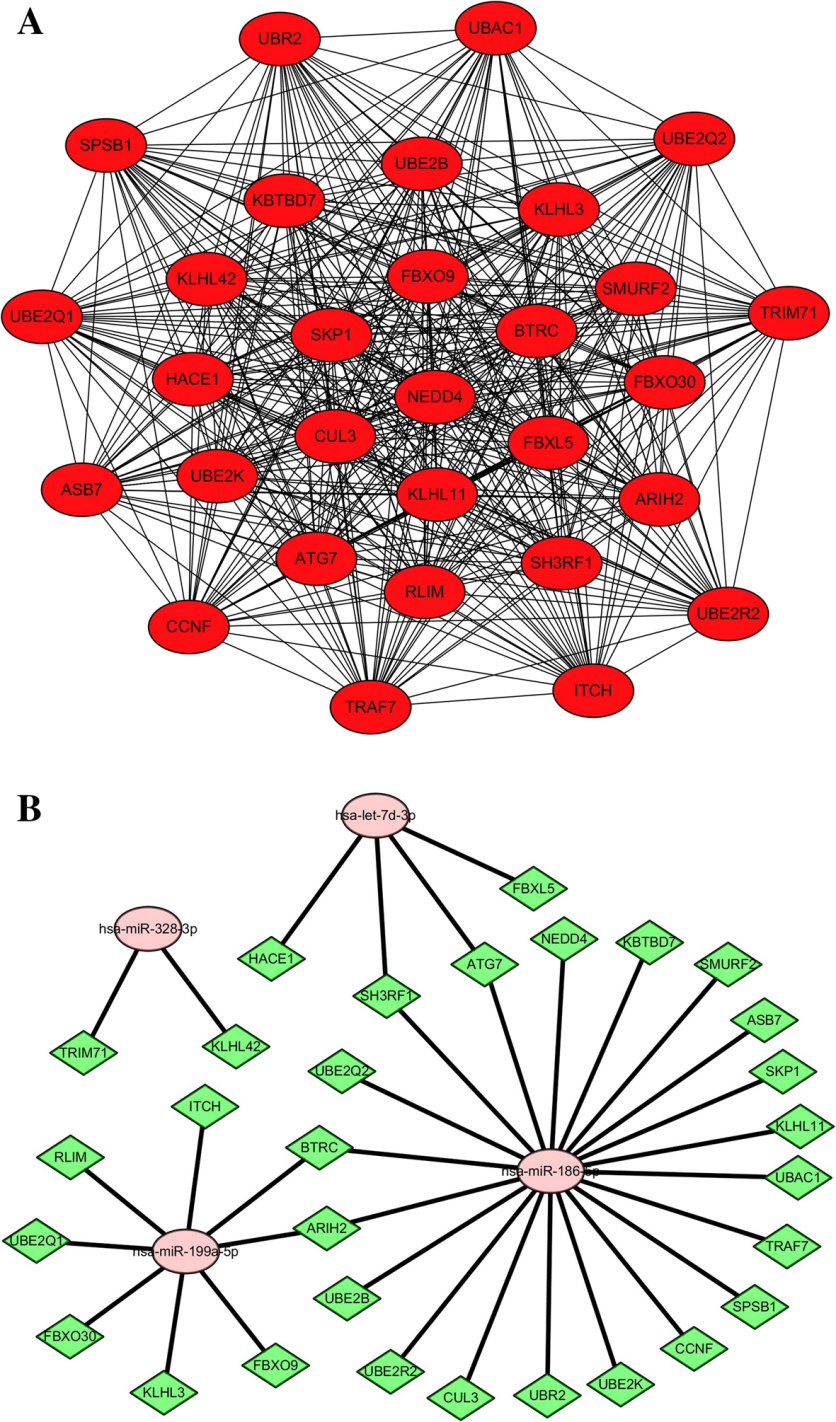
PPI and DEM-hub gene networks. **A**. PPI network of the top 30 hub genes of the DEMs. **B**. miRNA-hub gene regulatory network

The STRING database was used to establish a PPI network for the target genes of the DEMs, then the cytoHubba plug-in was used to screen the top 30 hub genes (Fig. 6A, Table 3). To better study the molecular mechanisms of these DEMs in the plasma of lung cancer patients, Cytoscape was used to construct a DEM-hub gene network (Fig. 6B). The interactions between the miRNAs and genes were as follows: miR-199a-5p interacted with 8 hub genes (*KLHL3*, *FBXO9*, *BTRC*, *ARIH2*, *ITCH*, *UBE2Q1*, *FBXO30,* and *RLIM*); let-7d-3p interacted with 4 hub genes (*FBXL5*, *ATG7*, *HACE1*, and *SH3RF1*); miR-328-3p interacted with 2 hub genes (*TRIM71* and *KLHL42*); and miR-186-5p interacted with 20 hub genes (*NEDD4*, *SKP1*, *CUL3*, *KLHL11*, *BTRC*, *CCNF*, *ATG7*, *SMURF2*, *KBTBD7*, *SPSB1*, *ASB7*, *UBE2K*, *TRAF7*, *ARIH2*, *UBE2R2*, *UBE2Q2*, *SH3RF1*, *UBAC1*, *UBE2B*, and *UBR2*).

**Table 3:** Top 30 Hub genes of the DEMs in the PPI Network Ranked by MCC

Gene Symbol	Score	Gene Symbol	Score	Gene Symbol	Score
NEDD4	8.68E+ 36	TRIM71	8.68E+ 36	ARIH2	8.68E+ 36
SKP1	8.68E+ 36	ITCH	8.68E+ 36	HACE1	8.68E+ 36
FBXO9	8.68E+ 36	FBXO30	8.68E+ 36	UBE2R2	8.68E+ 36
CUL3	8.68E+ 36	KLHL3	8.68E+ 36	UBE2B	8.68E+ 36
KLHL11	8.68E+ 36	KLHL42	8.68E+ 36	UBE2Q2	8.68E+ 36
FBXL5	8.68E+ 36	KBTBD7	8.68E+ 36	SH3RF1	8.68E+ 36
BTRC	8.68E+ 36	SPSB1	8.68E+ 36	UBE2Q1	8.68E+ 36
CCNF	8.68E+ 36	ASB7	8.68E+ 36	RLIM	8.68E+ 36
ATG7	8.68E+ 36	UBE2K	8.68E+ 36	UBAC1	8.68E+ 36
SMURF2	8.68E+ 36	TRAF7	8.68E+ 36	UBR2	8.68E+ 36

### Verification of hub gene expression

**Fig. 7:**
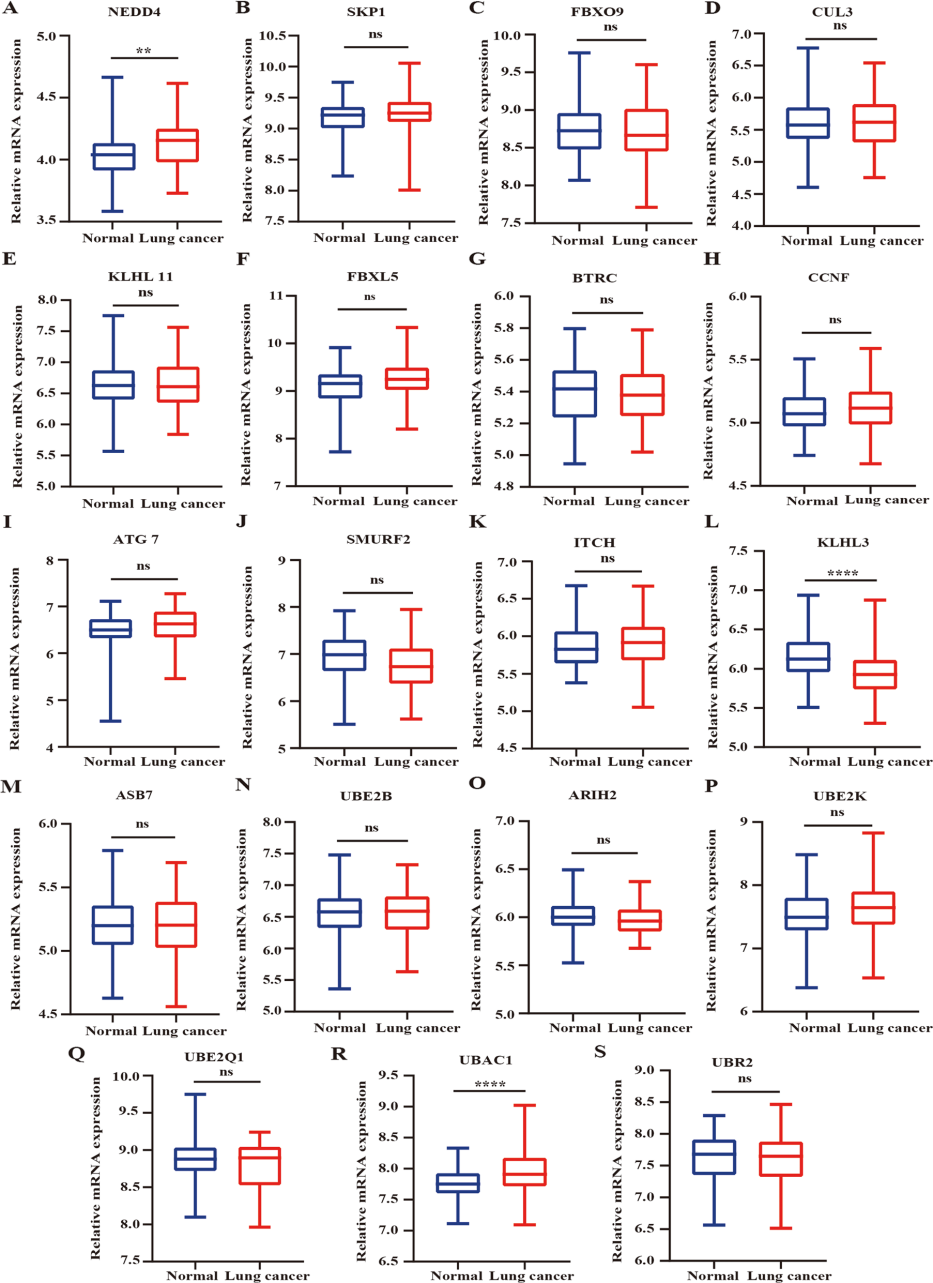
Identification of the mRNA expression levels of the top 30 hub genes in the GSE20189 dataset. **A**. *NEDD4*. **B**. *SKP1*. **C**. *FBXO9*. **D**. *CUL3*. **E**. *KLHL11.* **F**. *FBXL5*. **G**. *BTRC*. **H**. *CCNF*. **I**. *ATG7*. **J**. *SMURF2*. **K**. *ITCH*. **L**. *KLHL3*. **M**. *ASB7*. **N**. *UBE2B*. **O**. *ARIH2*. **P**. *UBE2K*. **Q**. *UBE2Q1*. **R**. *UBAC1*. **S**. *UBR2*. **p<0.01, ****p<0.0001

The GSE20189 dataset was used to identify the expression levels of the first 30 hub genes in the plasma samples. Some of these genes (*FBXO30*, *RLIM*, *HACE1*, *SH3RF1*, *KLHL42*, *KBTBD7*, *SPSB1*, *TRAF7*, *UBE2R2*, *UBE2Q2*, and *SH3RF1*) were not checked in the dataset. Compared with the healthy people, in the plasma of lung cancer patients, only the expression of *KLHL3* was continuously downregulated, while *NEDD4* and *UBAC1* were increased. (Fig. 7A-S). Therefore, miR-199a-5p-KLHL3 may be a potential regulatory pathway in lung cancer plasma.

### Expression levels of the DEMs and *KLHL3* in tissues

**Fig. 8:**
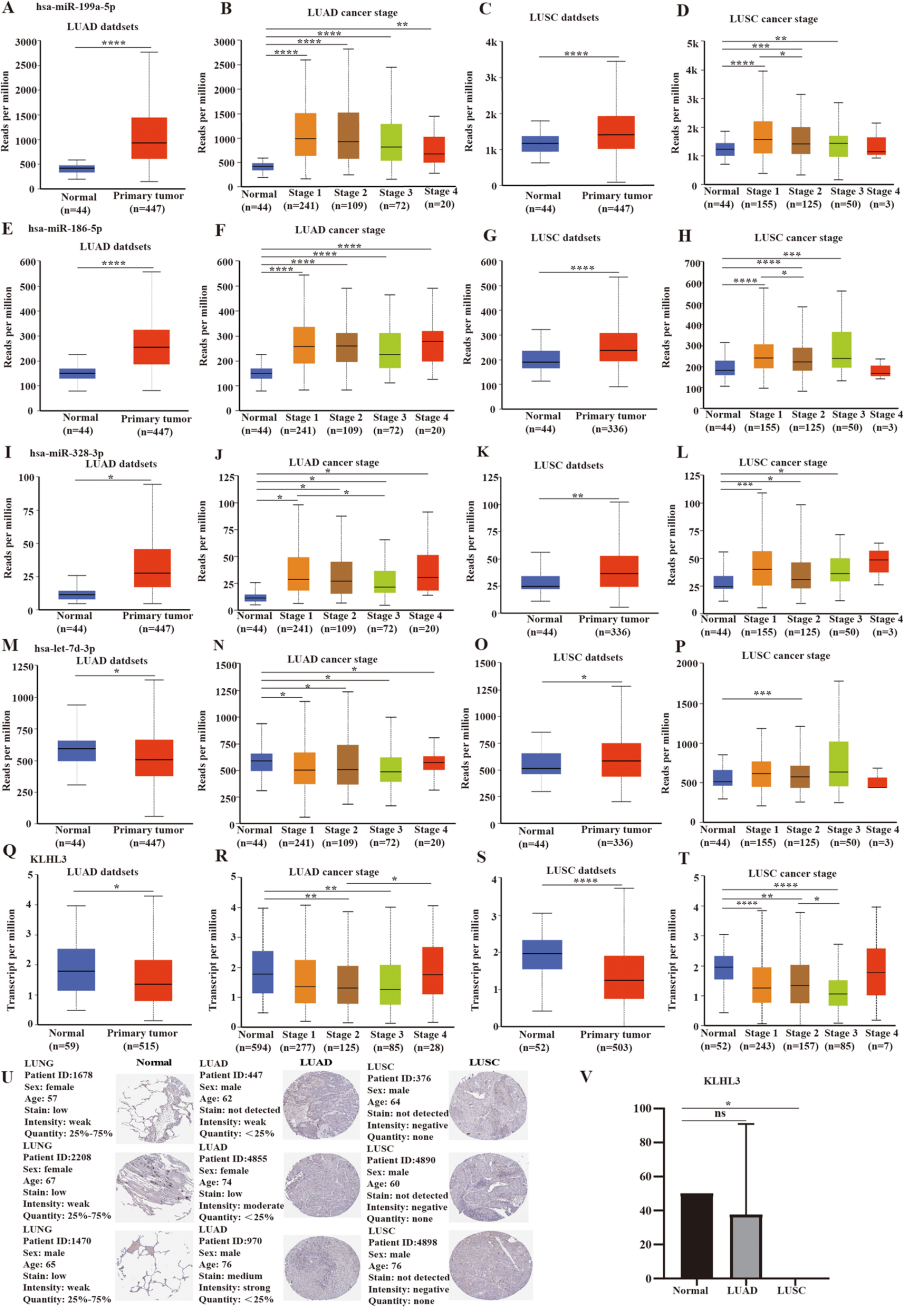
Expression levels of the DEMs and *KLHL3* in NSCLC tissues. **A-D**. hsa-miR-199a-5p. **E-H**. hsa-miR-186-5p. **I-L**. hsa-miR-328-3p. **M-P**. hsa-let-7d-3p. **Q-T**. *KLHL3*. **U**. KLHL3 protein expression in lung cancer tissue and normal tissue was verified by using the Human Protein Atlas database. Sample size (8 LUAD, 4 LUSC, 3 normal). *p<0.05, ***p<0.001, ****p<0.0001

To further explore the function of DEMs and KLHL3 in lung cancer, we analyze the expression of the DEMs and KLHL3 in lung cancerous tissues by using UALCAN database. As a result, an elevated expression of miR-199a-5p, miR-186-5p, and miR-328-3p were observed in LUAD and LUSC tissues compared to normal tissues (Fig. 8A, C, E, G, I, K). Also, the miR-199a-5p, miR-328-3p, and miR-186-5p expression were increased in diverse stages in LUAD patients (Fig. 8B, F, J). Nevertheless, in LUSC patients with stage 4, the expression of miR-199a-5p, miR-328-3p, miR-186-5p were not significantly increased (Fig. 8D, H, L). The expression of let-7d-3p in LUAD samples and diverse stages of LUAD patients were lower than the normal controls (Fig. 8M, N). In LUSC samples, the let-7d-3p expression was increased compared to the normal samples, but the expression of let-7d-3p was not obvious in different cancer stages with LUSC patients (Fig. 8O, P). In addition, the expression of KIHL3 was decreased in LUAD and LUSC patients, but the phenomenon was unconspicuous in different cancer stages (Fig. 8Q, R, S, T). Next, using HPA database, we found that a lower expression of KLHL3 protein was explored in LUAD and LUSC tissue versus normal tissues (Fig. 8U). As shown in Fig. 8V, the expression of KLHL3 was reduced in LUSC tissues.

### Survival analysis and prognostic roles of the DEMs and *KLHL3*

**Fig. 9:**
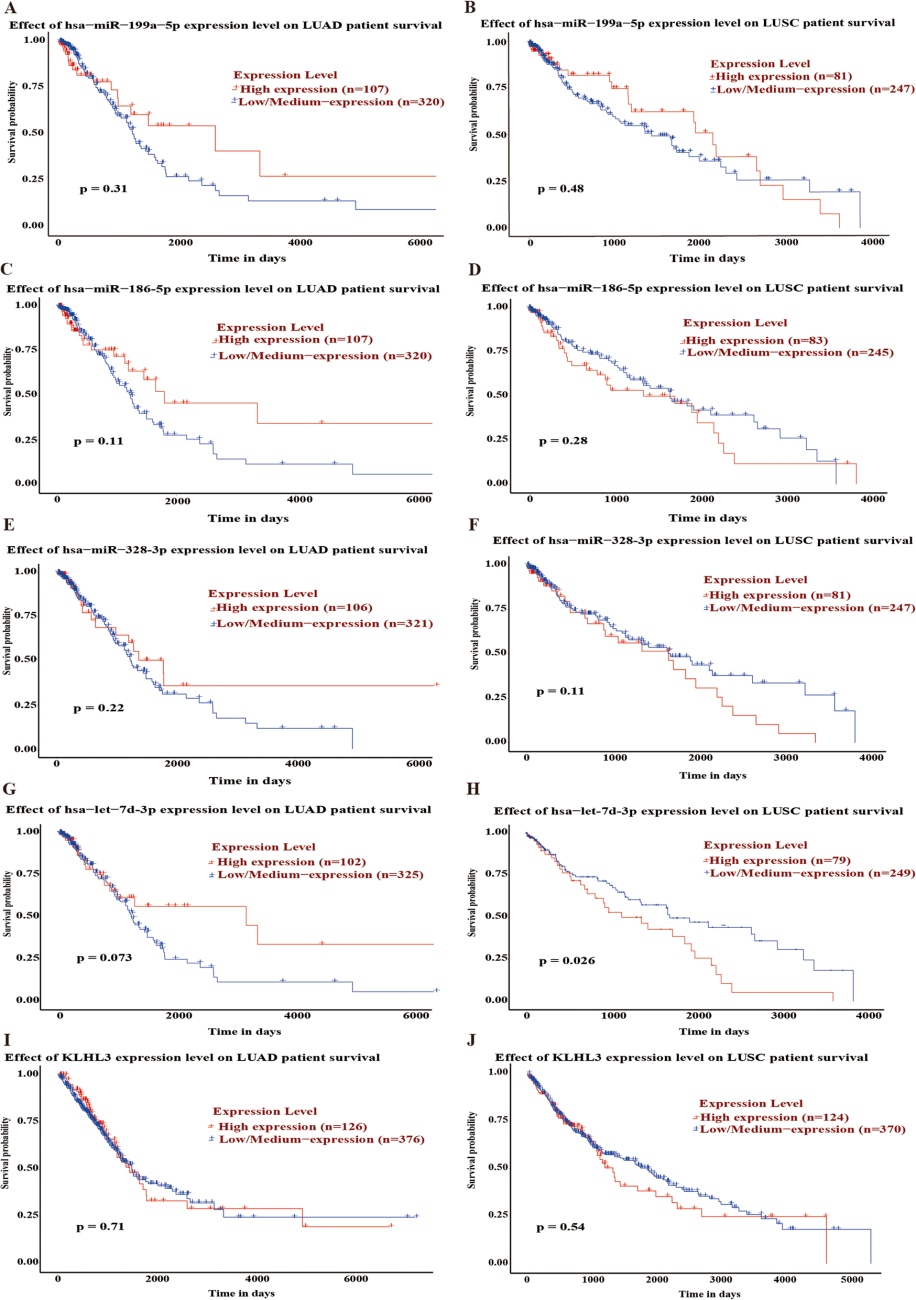
Overall survival analysis of the DEMs and *KLHL3* in NSCLC patients. **A-B**. hsa-miR-199a-5p. **C-D**. hsa-miR-186-5p. **E-F.** hsa-miR-328-3p. **G-H**. hsa-let-7d-3p. I-J. *KLHL3*

**Fig. 10:**
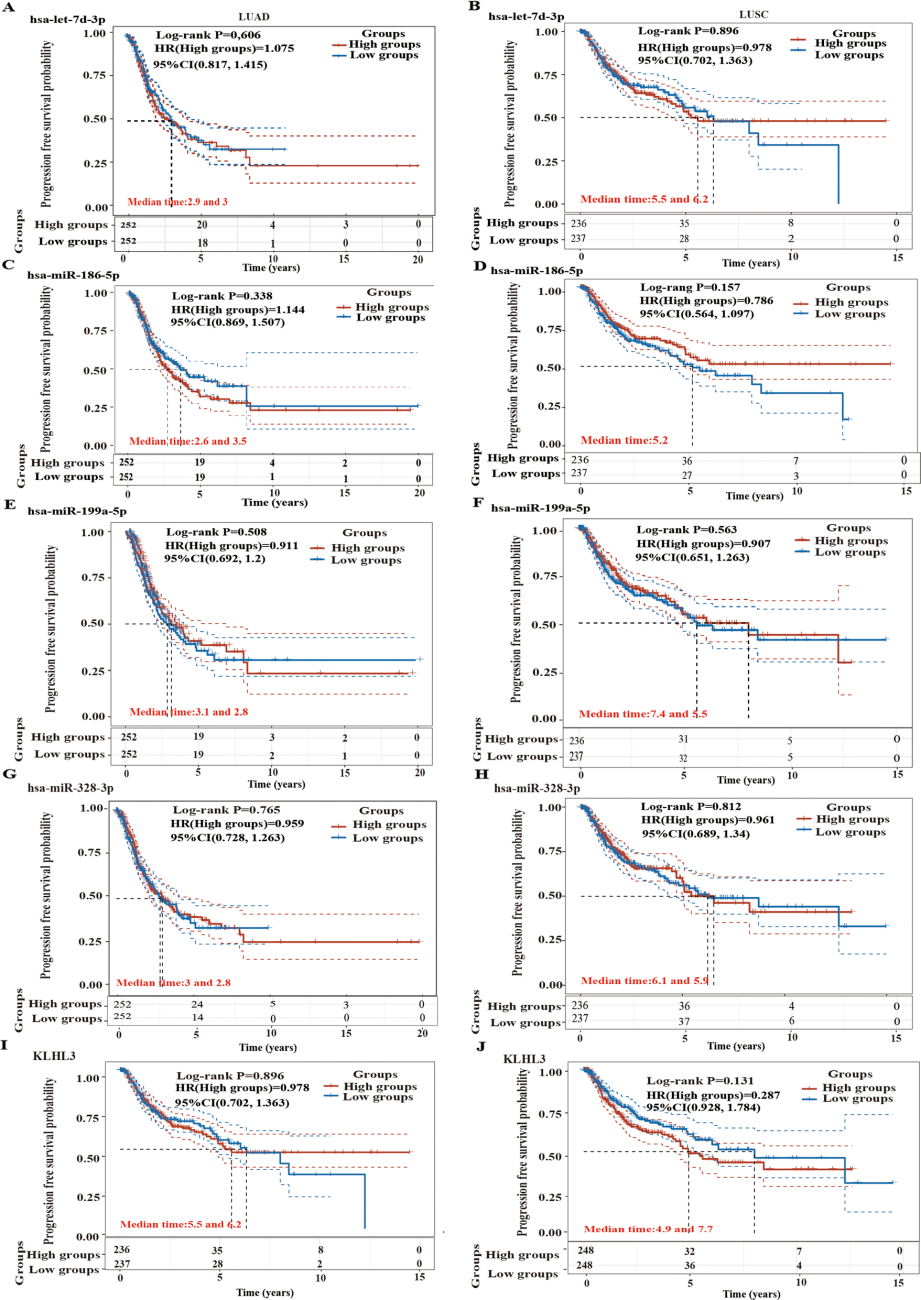
Progression free survival analysis of the DEMs and *KLHL3* in NSCLC patients. **A-B**. hsa-let-7d-3p. **C-D**. hsa-miR-186-5p. **E-F**. hsa-miR-199a-5p. **G-H**. hsa-miR-328-3p. **I-J**. *KLHL3*

Subsequently, we explored the prognostic value of the DEMs and *KLHL3* in lung cancer patients. The expression levels of miR-199a-5p, miR-186-5p, miR-328-3p, and *KLHL3* in the LUAD and LUSC patients did not affect OS (Fig. 9A-F, I-J). For LUSC, patients with high let-7d-3p expression had poor OS, but not in the LUAD (Fig. 9G, H). Therefore, let-7d-3p may be a potential prognostic marker of LUSC. The expression levels of the DEMs and *KLHL3* did not affect PFS in NSCLC patients (Fig. 10).

Kaplan-Meier curves showed that high hsa-let-7d-3p expression was closely associated with low OS in LUSC patients. Univariate and multivariate Cox regression analyses, nomograms, and ROC curves are required to evaluate the accuracy of differential expression genes for patient prognosis [30, 31]. Therefore, we researched whether hsa-let-7d-3p and variables such as age, sex, and tumor stage were risk factors for survival in LUSC patients.

**Fig. 11:**
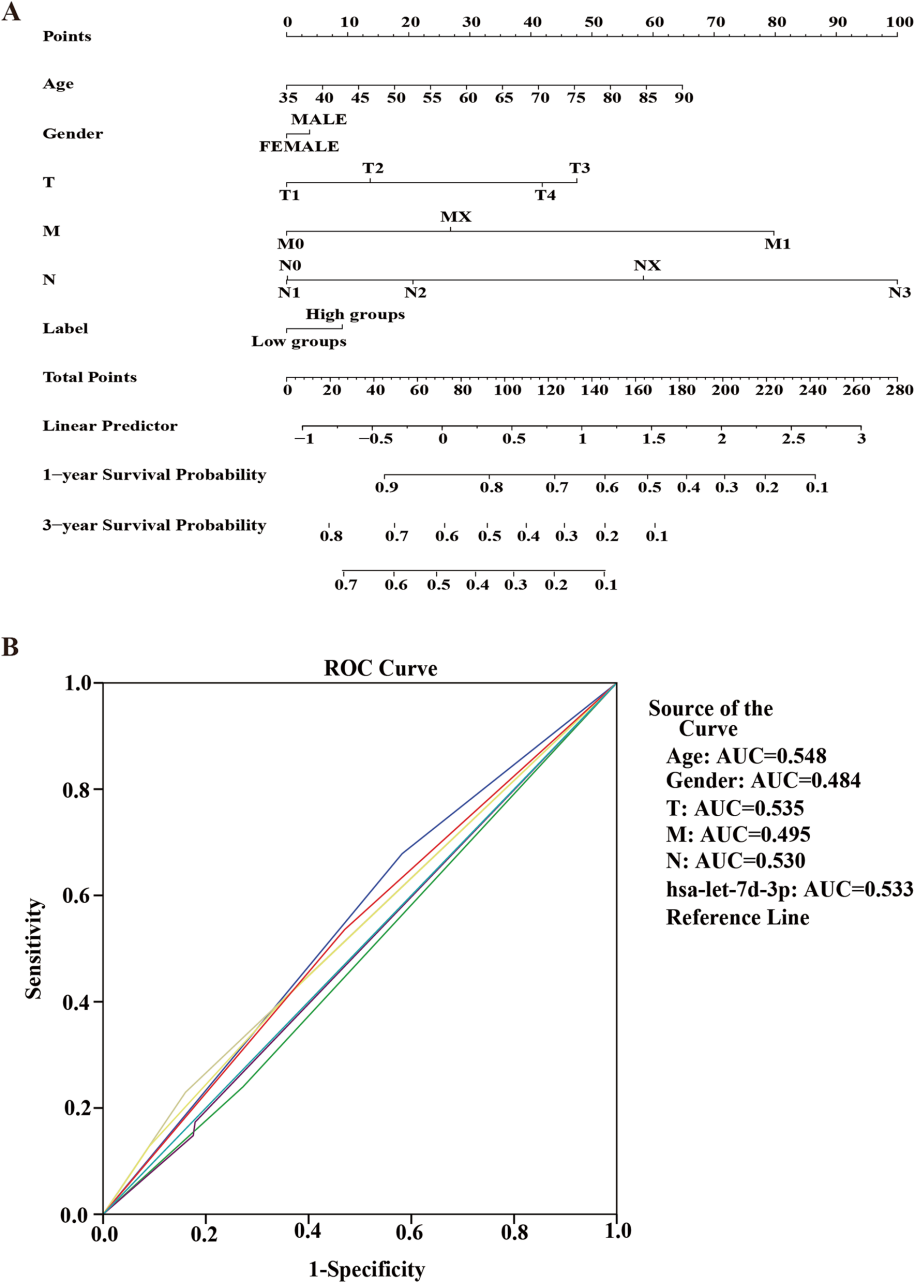
Nomogram and ROC curve for the prognostic value of hsa-let-7D-3p. **A**. Nomogram. **B**. ROC curve

Using univariate Cox regression, we know that hsa-let-7d-3p was not a risk factor in LUSC patients. The multivariate Cox regression revealed that let-7d-3p was not an independent prognostic factor in LUSC patients (Table 4). We also constructed a nomogram and ROC curve (Fig. 11A, B).

**Table 4:** Univariate and multivariate Cox proportional risk regression analysis of factors affecting the overall survival of LUSC patients

Variables	Number of patients	Univariate		Multivariate	
		HR (95% CI)	*p* Value	HR (95% CI)	*p* Value
Age(y)					
≤ 65	178	reference			
>65	290	1.274(0.9313–1.742)	0.13		
Gender					
Male	350	reference			
Female	123	0.932(0.6665–1.304	0.682		
Tumor stage					
stageI/II	384	reference			
stageIII/IV	85	0.999(0.5198–1.992)	0.9986		
T stage					
T1/T2	383	reference		reference	
T3/T4	90	1.761(1.1188–2.773)	0.0145*	1.721(1.23–2.408)	0.0015**
N stage					
N0	297	reference			
N1	126	1.025(0.7169–1.466)	0.8912		
N2	40	1.373(0.6291–2.995)	0.4262		
N3	4	4.473(0.9326–21.456)	0.0611		
NX	6	2.342(0.7388–7.421)			
M stage					
M0	387	reference		reference	
M1	6	3.418(1.2156–9.611)	0.0198*	3.308(1.349–8.115)	0.009**
MX	76	1.566(1.044–2.348)	0.0301*	1.611(1.078–2.401)	0.0201*
hsa-let-7d-3p					
Low expression	236	reference			
High expression	237	0.873(0.6523–1.168)	0.3593		

*HR* Hazard ratio, *CI* Confifidence interval

^*^signifificant risk factor, *p* < 0.05

^**^signifificant risk factor, *p* < 0.01

### External validation and effectiveness assessment

**Fig. 12:**
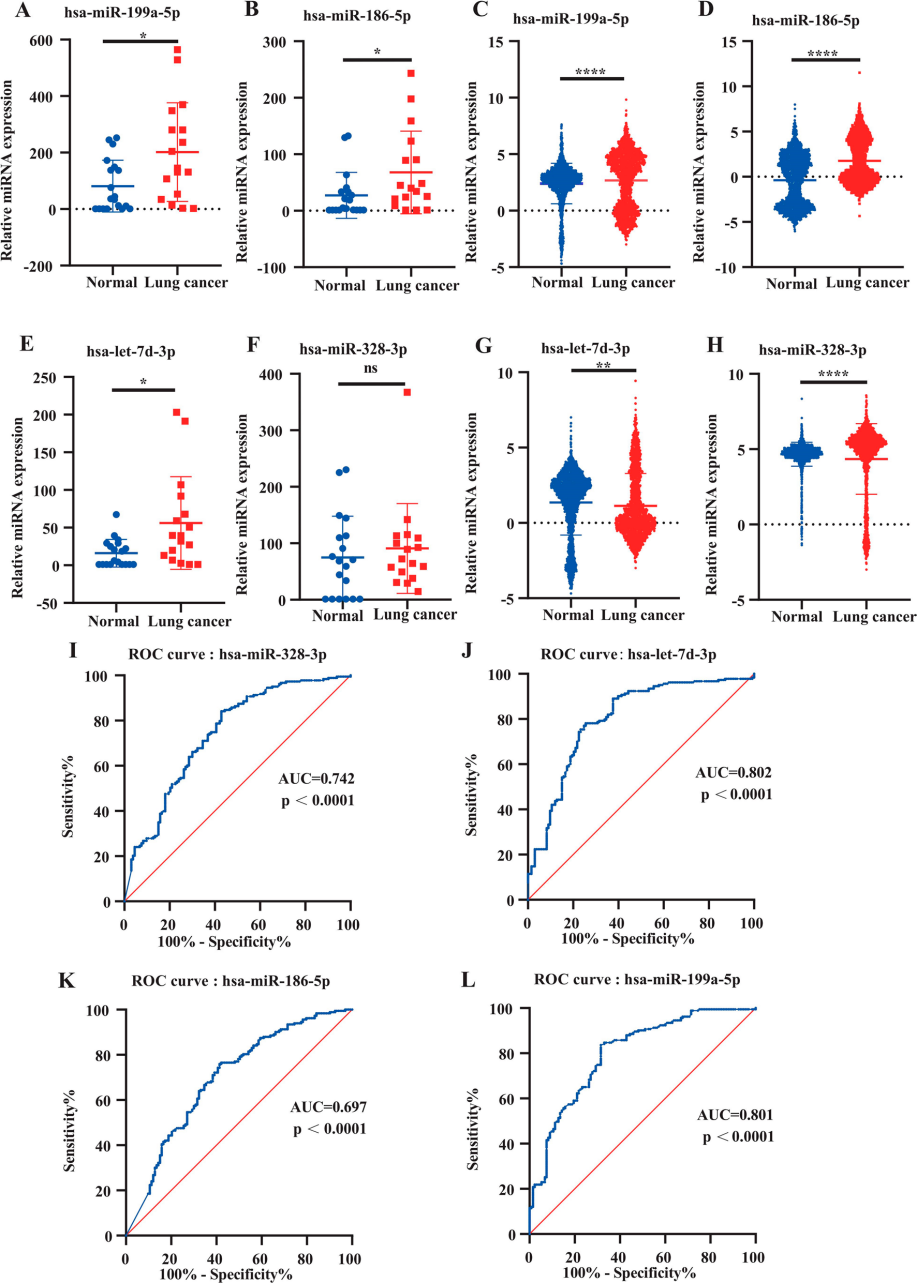
External validation and ROC analysis of the 4 DEMs. **A-H**. External validation of the 4 DEMs. **A**. hsa-miR-199a-5p (GSE17681). **B**. hsa-miR-186a-5p (GSE17681). **C**. hsa-miR-199a-5p (GSE137140). **D**. hsa-186-5p (GSE137140). **E**. hsa-let-7d-3p (GSE176181). **F**. hsa-miR-328-3p (GSE176181). **G**. hsa-let-7d-3p (GSE137140). **H**. hsa-328-3p (GSE137140). **I-J**. ROC analysis of the 4 DEMs. **I**. hsa-miR-328-3p. **J**. hsa-let-7d-3p. **K**. hsa-miR-186-5p. **L**. hsa-miR-199a-5p. *p<0.05, ****p<0.0001

To further verify the reliability of our results, we used GSE17681 and GSE137140 datasets to verify the expression levels of the 4 DEMs in the peripheral blood of lung cancer patients and healthy controls. In both GSE datasets, we found that the expression of hsa-miR-199a-5p and hsa-miR-186-5p in lung cancer patients were higher than those in the healthy controls, while the expression of hsa-let-7d-3p and hsa-328-3p was contrary to previous prediction results (Fig. 12A-H). In addition, ROC curves were used to assess the ability of the 4 DEMs to distinguish between lung cancer patients and healthy people. As shown in Fig. 12I-L, the 4 DEMs had good accuracy in the diagnosis of lung cancer, with the following results: hsa-miR-328-3p (AUC = 0.7416, 95%CI [0.6856–0.7977], *P* < 0.0001); hsa-let-7d-3p (AUC = 0.8021, 95%CI [0.7513–0.8529], *P* < 0.0001); hsa-miR-186-5p (AUC = 0.6970, 95%CI [0.6375–0.7565], *P* < 0.0001); and hsa-miR-199a-5p (AUC = 0.8013, 95%CI [0.7520–0.8506], *P* < 0.0001).

## Discussion

Although many studies regarding the diagnosis and treatment of lung cancer have been performed in recent years, the 5-year survival rate of lung cancer patients remains low due to the lack of biomarkers for early diagnosis and insufficient research on lung cancer pathogenesis. The number of studies involving microarrays is gradually increasing, which will help to reveal the genetic changes involved in disease progression. However, most micro-matrix studies on lung cancer are focused on tumors and adjacent tissues. Invasive operations can cause complications, such as hemothorax, pneumothorax, and infection; therefore, it is extremely important to actively seek effective noninvasive operation inspection strategies. Leidinger et al. found that miRNA in the blood can be used as a diagnostic marker for lung cancer, and it can also distinguish lung cancer from chronic obstructive pulmonary disease [32]. In this study, we found that 4 upregulated DEMs (hsa-miR-328-3p, hsa-miR-199a-5p, hsa-miR-186-5p, and let-7d-3p) had overlap in the 3 datasets, so they were used as candidate DEMs for further research. Recent studies have found that these DEMs play important roles in the diagnosis, treatment, and progression of different tumors. Ma et al. found that overexpressed miR-328-3p can improve the sensitivity of cancer cells to radiotherapy by changing the DNA damage and repair signaling pathway in NSCLC patients [33]. Yang et al. found that hsa-miR-574-5p, hsa-miR-328-3p, and hsa-miR-423-3p are involved in the Wnt/β-catenin signaling pathway to promote bone metastasis in lung cancer and may be biomarkers for bone metastasis in NSCLC patients [34]. Zheng et al. showed that plasma exosomal miR-30d-5p and let-7d-3p can be used as valuable biomarkers for the noninvasive screening of cervical cancer and its precancerous lesions [35]. Furthermore, the upregulation of miR-186-5p expression can increase the sensitivity of NSCLC to cisplatin by targeting the expression of the SIX1 protein; on the other hand, targeting the expression of the PTEN protein can promote LUAD migration, invasion, and proliferation [36, 37]. Some studies have found that miR-199a-5p dysregulation is related to the progression and pathogenesis of cancer. However, miR-199a-5p has conflicting roles in tumor progression and carcinogenesis. In thyroid cancer, oral squamous cell carcinoma, liver cancer, and ovarian cancer, miR-199a-5p can inhibit tumor growth and metastasis [38–41]. Conversely, miR-199a-5p can promote tumor growth and invasion in osteosarcoma, cervical cancer, and skin squamous cell carcinoma [42–44]. MiR-199a-5p can also inhibit the growth of lung cancer cells by targeting the MAP3K11 protein, as shown through in vivo and in vitro experiments of lung cancer [45]. It is believed that miR-199a-5p and miR-495 can be used in NSCLC as diagnostic biomarkers for activated unfolded protein response. Ahmadi et al. found that miR-199a-5p and miR-495 can regulate the endoplasmic reticulum stress response by regulating the expression of the GRP78 protein, thereby regulating the progression of lung cancer. In addition, the manipulation of the expression of these miRNAs may have potential therapeutic applications when it comes to lung cancer [46]. A recent study found that miR-199a-5p expression was significantly reduced in doxorubicin-resistant A549 and H469 cells. Increasing the expression of miR-199a-5p leads to the resistance of NSCLC to doxorubicin via the regulation of the expression of the ABCC1 and HIF1A proteins [47]. Therefore, the roles of miRNA-mRNA regulatory networks in the occurrence and development of lung cancer deserve further research.

Transcription factors can regulate the expression of miRNAs, so the transcription factors that might regulate the candidate DEMs in this study were predicted. We found that SP1, EGR1, and POU2F1 accounted for a high proportion of the transcription factors of the candidate DEMs. These transcription factors have been widely reported in studies of lung cancer and other tumors. Hu et al. found that high SP1 expression can inhibit the expression of the prenyl diphosphate synthase subunit 2 (PDSS2) promoter in lung cancer cells [48]. Higher SP1 expression and lower PDSS2 expression have been found to be significantly associated with poor prognosis in lung cancer patients [48]. SP1 can be combined with long noncoding RNA (lncRNA) promoters to promote the proliferation, migration, and invasion of lung cancer cells by regulating lncRNA expression [49, 50]. When hypoxia occurs, the level of EGR1 expression decreases, but SP1 and HIF1A expression levels increase, which can induce erythropoietin secretion and promote NSCLC growth [51]. POU2F1, which is also known as OCT1, promotes tumor growth and metastasis by activating downstream signaling pathways in liver cancer, colon cancer, ovarian cancer, and gastric cancer [52–55]. Xiao et al. found that ELF1/CASC2/miR-18a axis-mediated IRF2 expression is significantly related to the proliferation, migration, and invasion of cisplatin-resistant NSCLC, and they believe that this regulatory axis may be a new therapeutic target for NSCLC [56]. In general, many transcription factors have been reported in lung cancer, which supports the importance of the candidate DEMs in the pathogenesis of lung cancer.

The KEGG pathway enrichment analysis results of this study showed that the target genes of the DEMs were mainly enriched in cancer pathways, PI3K-Akt signaling pathways, proteoglycans, focal adhesions, endocytosis, Ras signaling pathways, actin cytoskeleton regulation in cancer, HTLV-I infection, and MAPK signaling pathways. In addition, cancer pathways included many pathways of growth factors, such as insulin-like growth factor I, fibroblast growth factor, recombinant human epidermal growth factor, platelet-derived growth factor, and human hepatocyte growth factor. These factors play important roles in the development and resistance of lung cancer [57–64]. Previous studies have shown that PI3k/Akt signaling pathway activation is closely related to lung cancer progression. Li et al. found that overexpressed miR-133b inhibits the proliferation of cisplatin-induced NSCLC cells through the regulation of the PI3K/Akt and JAK2/STAT3 signaling pathways by targeting epidermal growth factor receptor (EGFR) [65]. Wu et al. showed that the PAX6 protein can directly bind to the promoter region of the *ZEB2* gene and mediate the downregulation of E-cadherin through the PI3K/AKT signaling pathway, thereby mediating cell migration, stem cell transformation, and cisplatin resistance, and ultimately affecting the survival of NSCLC patients [66]. In addition, some studies have found that the Ras and MAPK signaling pathways are involved in lung cancer progression. Wei et al. showed that miR-330-3p promotes NSCLC brain metastasis by activating MAPK/ERK signaling pathways and enhancing cell proliferation, migration, invasion, and angiogenesis [67]. A recent study found that miR-148a-3p, which is a tumor suppressor, is often downregulated in NSCLC cells and can inhibit the proliferation and epithelial-mesenchymal transition processes of NSCLC by regulating SOS2/MAPK/ERK signal transduction, thereby providing new insight into the pathogenesis of NSCLC [68]. These studies suggest that the downstream target genes of DEMs are involved in many biological processes of lung cancer. They further suggest that the 4 upregulated DEMs in this study play key roles in the pathogenesis of lung cancer.

A DEM-hub gene network was constructed, and it was found that most of the hub genes may be targeted by miR-328-3p, miR-199a-5p, miR-186-5p, and let-7d-3p. Due to different samples, among the top 30 hub genes, only the expression of *KLHL3* was consistent with the expression in the GSE20189 database. Further, it was verified that the expression levels of *KLHL3* in tissues were consistent with those in plasma. The KLHL3 (kelch-like 3) and CUL3 proteins are components of the Cullin-RING E3 ubiquitin ligase complex, and they belong to the ubiquitin proteasome system. Their function is to degrade proteins, and they have important roles in maintaining cell function. The complex interacts with WNK1 and WNK4, which are part of the WNK kinase family, to induce WNK1 and WNK4 ubiquitination and regulate the levels of these proteins through proteasome degradation [69]. The secreted protein acidic and rich in cysteine (SPARC) secreted by extracellular matrix components can activate the expression of the WNK1 protein to promote the migration and invasion of lung cancer cells [70]. Hsu et al. found that activated lung fibroblasts produce the tryptophan metabolite kynurenine, which promotes the migration and growth of lung cancer cells by activating AKT and WNK1 [71, 72]. KLHL3 can degrade the expression of the WNK1 protein through ubiquitination, so it may have an important role in the progression of lung cancer.

Our research found that the expression levels of miR-199a-5p and miR-186-5p were increased in both blood and tissues. In this study, only GSE24709, GSE137140, and GSE20189 datasets provide the clinical information of patients, who were mainly concentrated in TNM stages I and II. Therefore, we speculated that the identified DEMs might be useful for diagnosing early stage lung cancer, while more experiments are needed to validate our hypothesis. Survival analysis showed that let-7d-3p expression was negatively correlated with the prognosis of patients with LUSC. However, the univariate Cox regression, the multivariate Cox regression and ROC curves showed that hsa-let-7d-3p might not be predicting the outcomes of LUSC patients. Through the above assessments, a potential miRNA-mRNA regulatory network in the plasma of NSCLC patients was constructed and analyzed for the first time.

There are some limitations in our research. First, we only focused on the expression levels of miRNAs and mRNAs in the plasma of lung cancer patients and healthy subjects. However, some miRNA and mRNA expression levels may differ in the peripheral blood of lung cancer patients with different tumor stages, tissue types, sexes, ages, and smoking statuses. Second, our data are mainly from public databases, some of which do not provide clinical information of patients, and due to the limited sample sizes of these datasets, the results may be biased. Third, we did not perform external experimental verification. The molecular mechanisms and roles of the miRNAs and target genes in regulating lung cancer’s migration, invasion, proliferation, and immune microenvironment need to be further studied. Therefore, in subsequent research, we will collect more clinical samples to verify the ability of the DEMs and target genes on the diagnosis and prognosis of NSCLC patients. At the same time, we need to further study the effects of DEMs and target genes on lung cancer biological characteristics and the immune microenvironment by in vitro and in vivo experiments.

## Conclusion

In conclusion, we found that miR-199a-5p and miR-186-5p may be noninvasive diagnostic biomarkers for NSCLC patients. MiR-199a-5p-KLHL3 may be involved in the development of NSCLC. We hope that more deep studies can provide new targets for the noninvasive diagnosis and treatment of lung cancer.

## Supplementary Information

**Additional file 1: Table S1.** DEMs between Lung cancer and normal plasma from GSE24709 dataset. **Table S2.** DEMs between Luan cancer and normal plasma from GSE31568 dataset. **Table S3.** DEMs between Luan cancer and normal plasma from GSE61741 dataset. TargetScanHuman database. **Table S4.** Target genes of DEMs predicted by TargetScanHuman. **Table S5.** Raw counts of RNA-sequencing data of hsa-let-7d-3p in LUAD from the TCGA. **Table S6.** Raw counts of RNA-sequencing data of hsa-miR-186-5p in LUAD from the TCGA. **Table S7.** Raw counts of RNA-sequencing data of hsa-miR-199a-5p in LUAD from the TCGA. **Table S8.** Raw counts of RNA-sequencing data of hsa-miR-328-3p in LUAD from the TCGA. **Table S9.** Raw counts of RNA-sequencing data of *KLHL3* in LUAD from the TCGA. **Table S10.** Raw counts of RNA-sequencing data of hsa-let-7d-3p in LUSC from the TCGA. **Table S11.** Raw counts of RNA-sequencing data of hsa-miR-186-5p in LUSC from the TCGA. **Table S12.** Raw counts of RNA-sequencing data of hsa-miR-199a-5p in LUSC from the TCGA. **Table S13.** Raw counts of RNA-sequencing data of hsa-miR-328-3p in LUSC from the TCGA. **Table S14.** Raw counts of RNA-sequencing data of *KLHL3* in LUSC from the TCGA. **Table S14.** Raw counts of RNA-sequencing data of *KLHL3* in LUSC from the TCGA. **Table S16.** The immunohistochemical score of KLHL3 in LUAD patients. **Table S17.** The immunohistochemical score of KLHL3 in LUSC patients. **Table S18.** The immunohistochemical score of KLHL3 in normal group.

## Data Availability

In the study, the Gene Expression Omnibus database was used to download microarray datasets (GSE24709, GSE61741, GSE31568, GSE20189, GSE17681, GSE137140) about miRNA and mRNA microarray datasets (https://www.ncbi.nlm.nih.gov/geo/).The datasets generated and analyzed during this study are available in the TCGA database (https://portal.gdc.cancer.gov).

## Abbreviations

NSCLC: Non-small cell lung cancer
LUAD: Lung Adenocarcinoma
LUSC: Lung Squamous Cell Carcinoma
miRNAs: microRNAs
DEGs: Differentially expressed genes
DEM: Differentially expressed miRNA
GO: Gene Ontology
PPI: Protein-protein interaction
GEO: Gene Expression Omnibus
FC: Fold change
KEGG: Kyoto Encyclopedia of Genes and Genomes
UALCAN: University of Alabama Cancer
HPA: Human Protein Atlas
TCGA: The Cancer Genome Atlas
OS: Overall survival
PFS: Progression free survival.
HR: Hazard ratio
CI: Confifidence interval
