# Supplementary material for: Using biological information to analyze potential miRNA-mRNA regulatory networks in the plasma of patients with non-small cell lung cancer
Source: BMC Cancer. 2022 Mar 21;22:299. doi: 10.1186/s12885-022-09281-1 (PMC8939143; doi:10.1186/s12885-022-09281-1)
Supplement: Supplementary file 1 — Additional file 1: Table S1. DEMs between Lung cancer and normal plasma from GSE24709 dataset. Table S2. DEMs between Luan cancer and normal plasma from GSE31568 dataset. Table S3. DEMs between Luan cancer and normal plasma from GSE61741 dataset. TargetScanHuman database. Table S4. Target genes of DEMs predicted by TargetScanHuman. Table S5. Raw counts of RNA-sequencing data of hsa-let-7d-3p in LUAD from the TCGA. Table S6. Raw counts of RNA-sequencing data of hsa-miR-186-5p in LUAD from the TCGA. Table S7. Raw counts of RNA-sequencing data of hsa-miR-199a-5p in LUAD from the TCGA. Table S8. Raw counts of RNA-sequencing data of hsa-miR-328-3p in LUAD from the TCGA. Table S9. Raw counts of RNA-sequencing data of KLHL3 in LUAD from the TCGA. Table S10. Raw counts of RNA-sequencing data of hsa-let-7d-3p in LUSC from the TCGA. Table S11. Raw counts of RNA-sequencing data of hsa-miR-186-5p in LUSC from the TCGA. Table S12. Raw counts of RNA-sequencing data of hsa-miR-199a-5p in LUSC from the TCGA. Table S13. Raw counts of RNA-sequencing data of hsa-miR-328-3p in LUSC from the TCGA. Table S14. Raw counts of RNA-sequencing data of KLHL3 in LUSC from the TCGA. Table S14. Raw counts of RNA-sequencing data of KLHL3 in LUSC from the TCGA. Table S16. The immunohistochemical score of KLHL3 in LUAD patients. Table S17. The immunohistochemical score of KLHL3 in LUSC patients. Table S18. The immunohistochemical score of KLHL3 in normal group. [file 12885_2022_9281_MOESM1_ESM.docx]

**Table S1.** DEMs between Lung cancer and normal plasma from GSE24709 dataset.

| id | logFC | AveExpr | t | P.Value | adj.P.Val | B |
| --- | --- | --- | --- | --- | --- | --- |
| hsa-miR-942 | 2.2002 | 5.1887 | 4.8462 | 0.0000 | 0.0010 | 3.1554 |
| hsa-miR-29c* | 2.1085 | 4.5689 | 3.8296 | 0.0004 | 0.0073 | 0.0084 |
| hsa-miR-432 | 1.9069 | 3.7360 | 5.2157 | 0.0000 | 0.0005 | 4.3678 |
| hsa-let-7d* | 1.8889 | 6.7841 | 5.6440 | 0.0000 | 0.0002 | 5.8026 |
| hsa-miR-601 | 1.8804 | 4.1654 | 4.1592 | 0.0001 | 0.0049 | 0.9914 |
| hsa-miR-142-3p | 1.8112 | 5.5199 | 3.2314 | 0.0022 | 0.0233 | -1.6574 |
| hsa-miR-328 | 1.7951 | 6.5656 | 4.3586 | 0.0001 | 0.0032 | 1.6050 |
| hsa-miR-130b* | 1.6700 | 4.4187 | 4.1260 | 0.0001 | 0.0049 | 0.8907 |
| hsa-miR-613 | 1.5959 | 3.7741 | 4.2733 | 0.0001 | 0.0040 | 1.3412 |
| hsa-miR-154 | 1.5391 | 3.6227 | 3.9472 | 0.0002 | 0.0063 | 0.3543 |
| hsa-miR-185* | 1.5239 | 4.3148 | 3.6079 | 0.0007 | 0.0112 | -0.6284 |
| hsa-miR-520d-3p | 1.5207 | 3.5795 | 4.0639 | 0.0002 | 0.0055 | 0.7030 |
| hsa-miR-132* | 1.5144 | 3.5592 | 3.8267 | 0.0004 | 0.0073 | -0.0004 |
| hsa-miR-1246 | 1.5029 | 3.7052 | 3.5617 | 0.0008 | 0.0118 | -0.7583 |
| hsa-miR-1914 | 1.5021 | 5.3086 | 3.7164 | 0.0005 | 0.0092 | -0.3196 |
| hsa-miR-769-3p | 1.4650 | 4.3568 | 3.6685 | 0.0006 | 0.0100 | -0.4565 |
| hsa-miR-1261 | 1.4568 | 3.7208 | 4.4094 | 0.0001 | 0.0029 | 1.7637 |
| hsa-miR-302b | 1.4434 | 3.6159 | 4.0100 | 0.0002 | 0.0061 | 0.5414 |
| hsa-miR-140-5p | 1.4137 | 4.9365 | 3.6491 | 0.0006 | 0.0102 | -0.5117 |
| hsa-miR-184 | 1.4079 | 4.7370 | 3.7930 | 0.0004 | 0.0079 | -0.0984 |
| hsa-let-7b* | 1.4069 | 4.5127 | 3.4838 | 0.0010 | 0.0142 | -0.9752 |
| hsa-miR-1321 | 1.3852 | 4.1738 | 3.9170 | 0.0003 | 0.0066 | 0.2648 |
| hsa-miR-190b | 1.3801 | 3.4441 | 3.8413 | 0.0003 | 0.0073 | 0.0423 |
| hsa-miR-186 | 1.3690 | 6.2097 | 2.8681 | 0.0060 | 0.0425 | -2.5792 |
| hsa-miR-1265 | 1.3372 | 4.0893 | 3.7863 | 0.0004 | 0.0079 | -0.1176 |
| hsa-miR-651 | 1.3014 | 3.7967 | 3.2304 | 0.0022 | 0.0233 | -1.6601 |
| hsa-miR-1282 | 1.2995 | 3.5555 | 3.1781 | 0.0025 | 0.0256 | -1.7973 |
| hsa-miR-211 | 1.2994 | 3.7526 | 3.3405 | 0.0016 | 0.0188 | -1.3667 |
| hsa-miR-1825 | 1.2923 | 6.2256 | 3.9339 | 0.0003 | 0.0064 | 0.3150 |
| hsa-miR-505 | 1.2843 | 5.3972 | 3.9715 | 0.0002 | 0.0063 | 0.4266 |
| hsa-miR-206 | 1.2635 | 3.9393 | 2.9947 | 0.0042 | 0.0344 | -2.2666 |
| hsa-miR-1262 | 1.2618 | 3.4065 | 3.5676 | 0.0008 | 0.0118 | -0.7419 |
| hsa-miR-1299 | 1.2598 | 4.7250 | 2.9821 | 0.0044 | 0.0349 | -2.2982 |
| hsa-miR-373 | 1.2382 | 3.5780 | 3.3426 | 0.0016 | 0.0188 | -1.3609 |
| hsa-miR-624* | 1.2377 | 6.1456 | 4.1995 | 0.0001 | 0.0047 | 1.1145 |
| hsa-miR-1294 | 1.2352 | 4.1166 | 2.8660 | 0.0060 | 0.0425 | -2.5844 |
| hsa-miR-23a* | 1.2283 | 3.8884 | 3.0686 | 0.0034 | 0.0312 | -2.0798 |
| hsa-miR-502-5p | 1.2181 | 4.9666 | 2.9824 | 0.0044 | 0.0349 | -2.2973 |
| hsa-miR-376a* | 1.2102 | 3.9721 | 3.5678 | 0.0008 | 0.0118 | -0.7412 |
| hsa-miR-224 | 1.1987 | 4.9980 | 3.0352 | 0.0038 | 0.0332 | -2.1645 |
| hsa-miR-1224-5p | 1.1781 | 5.2636 | 3.1929 | 0.0024 | 0.0250 | -1.7586 |
| hsa-miR-92a-2* | 1.1549 | 3.8606 | 2.8313 | 0.0066 | 0.0447 | -2.6682 |
| hsa-miR-556-3p | 1.1419 | 3.7647 | 2.8871 | 0.0057 | 0.0412 | -2.5330 |
| hsa-miR-485-3p | 1.1408 | 6.5152 | 3.2769 | 0.0019 | 0.0214 | -1.5370 |
| hsa-miR-593 | 1.1396 | 3.5839 | 2.9962 | 0.0042 | 0.0344 | -2.2629 |
| hsa-miR-1468 | 1.1291 | 3.7203 | 3.2057 | 0.0023 | 0.0244 | -1.7252 |
| hsa-miR-550* | 1.1016 | 9.3120 | 4.6364 | 0.0000 | 0.0018 | 2.4804 |
| hsa-miR-378* | 1.1015 | 6.2918 | 2.8268 | 0.0067 | 0.0447 | -2.6792 |
| hsa-miR-1227 | 1.0612 | 6.5994 | 4.5755 | 0.0000 | 0.0020 | 2.2868 |
| hsa-miR-199a-5p | 1.0401 | 8.9231 | 4.1937 | 0.0001 | 0.0047 | 1.0967 |
| hsa-miR-483-5p | 1.0206 | 7.4472 | 2.8662 | 0.0060 | 0.0425 | -2.5837 |
| hsa-miR-548d-5p | 1.0080 | 3.3998 | 2.8376 | 0.0065 | 0.0445 | -2.6530 |
| hsa-miR-202* | -1.0030 | 6.6257 | -3.0723 | 0.0034 | 0.0312 | -2.0703 |
| hsa-miR-655 | -1.0136 | 5.4883 | -2.9122 | 0.0053 | 0.0401 | -2.4714 |
| hsa-miR-136* | -1.0162 | 5.9818 | -3.1452 | 0.0028 | 0.0269 | -1.8829 |
| hsa-miR-186* | -1.0382 | 6.5998 | -3.3686 | 0.0014 | 0.0177 | -1.2908 |
| hsa-miR-606 | -1.0434 | 6.5202 | -3.6627 | 0.0006 | 0.0100 | -0.4732 |
| hsa-miR-154* | -1.0435 | 5.5718 | -3.2712 | 0.0019 | 0.0215 | -1.5519 |
| hsa-miR-592 | -1.0769 | 5.6765 | -3.0179 | 0.0040 | 0.0335 | -2.2084 |
| hsa-miR-562 | -1.0858 | 5.1645 | -2.9579 | 0.0047 | 0.0367 | -2.3584 |
| hsa-miR-126 | -1.0931 | 10.6127 | -4.1351 | 0.0001 | 0.0049 | 0.9183 |
| hsa-miR-487a | -1.0999 | 6.1704 | -3.3786 | 0.0014 | 0.0177 | -1.2635 |
| hsa-miR-208b | -1.1204 | 6.1487 | -3.8289 | 0.0004 | 0.0073 | 0.0062 |
| hsa-miR-618 | -1.1322 | 4.4311 | -3.0291 | 0.0038 | 0.0334 | -2.1800 |
| hsa-miR-144 | -1.1684 | 10.9011 | -3.8332 | 0.0003 | 0.0073 | 0.0187 |
| hsa-miR-548p | -1.1846 | 7.0612 | -3.6634 | 0.0006 | 0.0100 | -0.4711 |
| hsa-miR-9* | -1.1943 | 6.3873 | -3.6006 | 0.0007 | 0.0112 | -0.6490 |
| hsa-miR-103 | -1.2076 | 12.2677 | -4.9912 | 0.0000 | 0.0008 | 3.6279 |
| hsa-miR-20a | -1.2372 | 11.9549 | -4.5208 | 0.0000 | 0.0023 | 2.1135 |
| hsa-miR-18a | -1.3031 | 10.4524 | -5.5199 | 0.0000 | 0.0002 | 5.3839 |
| hsa-miR-20b | -1.3291 | 11.5504 | -6.2097 | 0.0000 | 0.0001 | 7.7293 |
| hsa-miR-18b | -1.4040 | 8.3716 | -5.8088 | 0.0000 | 0.0002 | 6.3609 |

**Table S2.** DEMs between Luan cancer and normal plasma from GSE31568 dataset.

| id | logFC | AveExpr | t | P.Value | adj.P.Val | B |
| --- | --- | --- | --- | --- | --- | --- |
| hsa-miR-624* | 1.3911 | 4.9367 | 4.7200 | 0.0000 | 0.0029 | 3.6113 |
| hsa-miR-142-3p | 1.3672 | 4.7200 | 3.6812 | 0.0004 | 0.0185 | 0.0136 |
| hsa-miR-199a-5p | 1.3203 | 8.0852 | 5.6153 | 0.0000 | 0.0001 | 7.1684 |
| hsa-miR-186 | 1.2680 | 5.3080 | 3.4679 | 0.0007 | 0.0256 | -0.6418 |
| hsa-miR-328 | 1.2168 | 5.7764 | 3.6246 | 0.0004 | 0.0197 | -0.1633 |
| hsa-let-7d* | 1.1873 | 6.1232 | 3.8819 | 0.0002 | 0.0147 | 0.6575 |
| hsa-miR-942 | 1.1742 | 4.5152 | 3.3825 | 0.0010 | 0.0327 | -0.8955 |
| hsa-miR-625 | 1.1360 | 6.0187 | 3.3381 | 0.0011 | 0.0328 | -1.0253 |
| hsa-miR-485-3p | 1.0946 | 5.5939 | 3.6617 | 0.0004 | 0.0185 | -0.0476 |
| hsa-miR-1303 | 1.0869 | 4.9238 | 4.0336 | 0.0001 | 0.0144 | 1.1615 |
| hsa-miR-500 | 1.0721 | 7.7746 | 3.5549 | 0.0006 | 0.0227 | -0.3782 |
| hsa-miR-1256 | -1.0273 | 5.5246 | -3.8357 | 0.0002 | 0.0148 | 0.5069 |
| hsa-miR-647 | -1.0657 | 5.6833 | -4.4877 | 0.0000 | 0.0050 | 2.7525 |
| hsa-miR-519e* | -1.0974 | 5.4816 | -4.2723 | 0.0000 | 0.0083 | 1.9828 |

**Table S3.** DEMs between Luan cancer and normal plasma from GSE61741 dataset.

| id | logFC | AveExpr | t | P.Value | adj.P.Val | B |
| --- | --- | --- | --- | --- | --- | --- |
| hsa-miR-135a* | 1.8644 | 3.3848 | 6.6498 | 0.0000 | 0.0000 | 12.7585 |
| hsa-miR-483-3p | 1.5813 | 4.6965 | 5.0774 | 0.0000 | 0.0000 | 5.2029 |
| hsa-miR-130b* | 1.5782 | 2.7682 | 6.2063 | 0.0000 | 0.0000 | 10.4950 |
| hsa-let-7d* | 1.5565 | 6.5507 | 6.7132 | 0.0000 | 0.0000 | 13.0897 |
| hsa-miR-892a | 1.4440 | 3.3081 | 5.7234 | 0.0000 | 0.0000 | 8.1439 |
| hsa-miR-132* | 1.4434 | 2.8169 | 5.4864 | 0.0000 | 0.0000 | 7.0362 |
| hsa-miR-409-3p | 1.4401 | 4.8974 | 4.8111 | 0.0000 | 0.0001 | 4.0656 |
| hsa-miR-877* | 1.3623 | 5.5047 | 4.8019 | 0.0000 | 0.0001 | 4.0271 |
| hsa-miR-296-3p | 1.3612 | 5.0426 | 4.6891 | 0.0000 | 0.0001 | 3.5599 |
| hsa-miR-29a* | 1.2976 | 2.7980 | 5.1613 | 0.0000 | 0.0000 | 5.5706 |
| hsa-miR-199a-5p | 1.2615 | 8.0620 | 5.9626 | 0.0000 | 0.0000 | 9.2932 |
| hsa-miR-1261 | 1.2548 | 3.1701 | 4.5822 | 0.0000 | 0.0001 | 3.1250 |
| hsa-miR-138 | 1.2508 | 4.6875 | 4.4620 | 0.0000 | 0.0002 | 2.6456 |
| hsa-miR-1275 | 1.2139 | 6.0230 | 5.6030 | 0.0000 | 0.0000 | 7.5770 |
| hsa-miR-658 | 1.2080 | 5.1200 | 4.3985 | 0.0000 | 0.0002 | 2.3962 |
| hsa-miR-204 | 1.1889 | 2.6561 | 4.4736 | 0.0000 | 0.0002 | 2.6912 |
| hsa-miR-202 | 1.1835 | 3.8293 | 4.1607 | 0.0001 | 0.0005 | 1.4877 |
| hsa-miR-1303 | 1.1624 | 4.4082 | 4.3528 | 0.0000 | 0.0002 | 2.2186 |
| hsa-miR-939 | 1.1516 | 5.4257 | 4.7611 | 0.0000 | 0.0001 | 3.8574 |
| hsa-miR-373 | 1.1479 | 3.2309 | 4.0592 | 0.0001 | 0.0006 | 1.1129 |
| hsa-miR-302c | 1.1473 | 3.2224 | 4.2531 | 0.0000 | 0.0003 | 1.8360 |
| hsa-miR-34a | 1.1418 | 3.7731 | 4.2975 | 0.0000 | 0.0003 | 2.0053 |
| hsa-miR-328 | 1.1323 | 5.8716 | 4.2244 | 0.0000 | 0.0004 | 1.7274 |
| hsa-miR-330-5p | 1.1216 | 3.8633 | 4.0746 | 0.0001 | 0.0006 | 1.1690 |
| hsa-miR-548i | 1.1069 | 2.8733 | 4.5643 | 0.0000 | 0.0001 | 3.0532 |
| hsa-miR-576-5p | 1.1003 | 2.5414 | 4.4801 | 0.0000 | 0.0002 | 2.7169 |
| hsa-miR-302d | 1.0973 | 2.6996 | 4.3471 | 0.0000 | 0.0002 | 2.1963 |
| hsa-let-7e* | 1.0908 | 3.2577 | 4.0172 | 0.0001 | 0.0007 | 0.9596 |
| hsa-miR-302c* | 1.0514 | 3.9539 | 3.9664 | 0.0001 | 0.0008 | 0.7762 |
| hsa-miR-1225-5p | 1.0471 | 5.8784 | 4.7260 | 0.0000 | 0.0001 | 3.7120 |
| hsa-miR-186 | 1.0400 | 5.1396 | 3.5034 | 0.0006 | 0.0031 | -0.8038 |
| hsa-miR-194* | 1.0372 | 4.8721 | 4.1279 | 0.0001 | 0.0005 | 1.3658 |
| hsa-miR-206 | 1.0272 | 3.0570 | 3.6662 | 0.0003 | 0.0019 | -0.2673 |
| hsa-miR-1290 | 1.0138 | 2.7848 | 3.7545 | 0.0002 | 0.0015 | 0.0327 |
| hsa-miR-135a | 1.0130 | 2.6679 | 3.8881 | 0.0001 | 0.0010 | 0.4977 |
| hsa-miR-145 | 1.0114 | 7.2296 | 5.0949 | 0.0000 | 0.0000 | 5.2794 |
| hsa-miR-1321 | 1.0107 | 3.4635 | 3.6357 | 0.0004 | 0.0021 | -0.3691 |
| hsa-miR-411 | -1.0031 | 4.7165 | -3.9961 | 0.0001 | 0.0007 | 0.8833 |
| hsa-miR-450b-3p | -1.0255 | 4.1322 | -3.7017 | 0.0003 | 0.0018 | -0.1473 |
| hsa-miR-144* | -1.0263 | 8.9707 | -7.7600 | 0.0000 | 0.0000 | 18.7961 |
| hsa-miR-219-5p | -1.0390 | 4.8827 | -4.0856 | 0.0001 | 0.0006 | 1.2095 |
| hsa-miR-548c-3p | -1.0426 | 4.4412 | -3.9642 | 0.0001 | 0.0008 | 0.7684 |
| hsa-miR-146b-3p | -1.0453 | 5.5435 | -5.0344 | 0.0000 | 0.0000 | 5.0165 |
| hsa-miR-374a* | -1.0511 | 4.4466 | -3.6602 | 0.0003 | 0.0020 | -0.2874 |
| hsa-miR-1537 | -1.0557 | 4.4350 | -3.6795 | 0.0003 | 0.0019 | -0.2223 |
| hsa-miR-9* | -1.0750 | 5.8860 | -4.8994 | 0.0000 | 0.0000 | 4.4379 |
| hsa-miR-1289 | -1.0805 | 6.0071 | -5.0948 | 0.0000 | 0.0000 | 5.2791 |
| hsa-miR-551b | -1.0833 | 5.4606 | -4.6867 | 0.0000 | 0.0001 | 3.5500 |
| hsa-miR-1247 | -1.1311 | 5.2808 | -4.8907 | 0.0000 | 0.0000 | 4.4008 |
| hsa-miR-934 | -1.1318 | 5.3359 | -4.4964 | 0.0000 | 0.0001 | 2.7818 |
| hsa-miR-597 | -1.1344 | 5.1846 | -4.4903 | 0.0000 | 0.0001 | 2.7576 |
| hsa-miR-214* | -1.1524 | 4.9556 | -4.9935 | 0.0000 | 0.0000 | 4.8397 |
| hsa-miR-519d | -1.1714 | 4.9713 | -4.7833 | 0.0000 | 0.0001 | 3.9493 |
| hsa-miR-558 | -1.1807 | 5.5832 | -4.3825 | 0.0000 | 0.0002 | 2.3339 |
| hsa-miR-107 | -1.1908 | 10.2305 | -8.9687 | 0.0000 | 0.0000 | 25.8433 |
| hsa-miR-640 | -1.2176 | 5.5763 | -4.7838 | 0.0000 | 0.0001 | 3.9516 |
| hsa-miR-223* | -1.2263 | 4.7284 | -3.9222 | 0.0001 | 0.0009 | 0.6186 |
| hsa-miR-545 | -1.3062 | 6.1104 | -6.2031 | 0.0000 | 0.0000 | 10.4791 |
| hsa-miR-367* | -1.3769 | 4.8212 | -5.4764 | 0.0000 | 0.0000 | 6.9902 |
| hsa-miR-516b | -1.4289 | 4.1038 | -5.1421 | 0.0000 | 0.0000 | 5.4862 |
| hsa-miR-561 | -1.4473 | 4.3745 | -5.4312 | 0.0000 | 0.0000 | 6.7831 |
| hsa-miR-548d-3p | -1.4830 | 4.5691 | -5.5524 | 0.0000 | 0.0000 | 7.3414 |
| hsa-miR-208b | -1.5163 | 4.7958 | -5.6581 | 0.0000 | 0.0000 | 7.8353 |
| hsa-miR-34a* | -1.5916 | 5.6385 | -6.1987 | 0.0000 | 0.0000 | 10.4573 |

**Table S4.** Target genes of DEMs predicted by TargetScanHuman.

| ID | Gene |
| --- | --- |
| hsa-miR-199a-5p | ZNF776 |
| hsa-miR-199a-5p | ZNF439 |
| hsa-miR-199a-5p | ZNF544 |
| hsa-miR-199a-5p | ZNF791 |
| hsa-miR-199a-5p | ZNF788 |
| hsa-miR-199a-5p | ZIK1 |
| hsa-miR-199a-5p | ZNF772 |
| hsa-miR-199a-5p | ZNF709 |
| hsa-miR-199a-5p | ZNF709 |
| hsa-miR-199a-5p | ZNF584 |
| hsa-miR-199a-5p | ZNF625 |
| hsa-miR-199a-5p | ZNF256 |
| hsa-miR-199a-5p | ZNF547 |
| hsa-miR-199a-5p | MAP3K11 |
| hsa-miR-199a-5p | LIN7C |
| hsa-miR-199a-5p | PVRL2 |
| hsa-miR-199a-5p | RP11-1396O13.13 |
| hsa-miR-199a-5p | ZNF23 |
| hsa-miR-199a-5p | BCAM |
| hsa-miR-199a-5p | SHOC2 |
| hsa-miR-199a-5p | HAPLN1 |
| hsa-miR-199a-5p | ZFP2 |
| hsa-miR-199a-5p | TST |
| hsa-miR-199a-5p | MYRF |
| hsa-miR-199a-5p | CELSR1 |
| hsa-miR-199a-5p | GPR63 |
| hsa-miR-199a-5p | NSG1 |
| hsa-miR-199a-5p | RAD23B |
| hsa-miR-199a-5p | ZBTB42 |
| hsa-miR-199a-5p | CRYBG3 |
| hsa-miR-199a-5p | RBM47 |
| hsa-miR-199a-5p | PDPN |
| hsa-miR-199a-5p | HMCN1 |
| hsa-miR-199a-5p | MGAT4B |
| hsa-miR-199a-5p | MAB21L1 |
| hsa-miR-199a-5p | FP15737 |
| hsa-miR-199a-5p | LCOR |
| hsa-miR-199a-5p | RP11-122A3.2 |
| hsa-miR-199a-5p | ARHGAP21 |
| hsa-miR-199a-5p | ZNF225 |
| hsa-miR-199a-5p | TSPAN6 |
| hsa-miR-199a-5p | ECE1 |
| hsa-miR-199a-5p | SULF1 |
| hsa-miR-199a-5p | GCNT2 |
| hsa-miR-199a-5p | AKAP1 |
| hsa-miR-199a-5p | FAM222B |
| hsa-miR-199a-5p | ALS2 |
| hsa-miR-199a-5p | GPR89B |
| hsa-miR-199a-5p | MAGT1 |
| hsa-miR-199a-5p | SLC24A3 |
| hsa-miR-199a-5p | ZNF846 |
| hsa-miR-199a-5p | B3GNT1 |
| hsa-miR-199a-5p | TGFB2 |
| hsa-miR-199a-5p | ZNF329 |
| hsa-miR-199a-5p | EPB41L1 |
| hsa-miR-199a-5p | VPS26A |
| hsa-miR-199a-5p | FLRT3 |
| hsa-miR-199a-5p | CCDC43 |
| hsa-miR-199a-5p | GJA5 |
| hsa-miR-199a-5p | PODXL |
| hsa-miR-199a-5p | FZD6 |
| hsa-miR-199a-5p | ANGEL2 |
| hsa-miR-199a-5p | ARF6 |
| hsa-miR-199a-5p | PPP1R2 |
| hsa-miR-199a-5p | FAM107B |
| hsa-miR-199a-5p | ACPT |
| hsa-miR-199a-5p | ARHGAP12 |
| hsa-miR-199a-5p | TAF9B |
| hsa-miR-199a-5p | GPR89A |
| hsa-miR-199a-5p | RASSF2 |
| hsa-miR-199a-5p | TMEM66 |
| hsa-miR-199a-5p | METTL21A |
| hsa-miR-199a-5p | UBL3 |
| hsa-miR-199a-5p | GPR89C |
| hsa-miR-199a-5p | ABHD17C |
| hsa-miR-199a-5p | ASRGL1 |
| hsa-miR-199a-5p | SLC25A23 |
| hsa-miR-199a-5p | CCNJ |
| hsa-miR-199a-5p | LEPREL1 |
| hsa-miR-199a-5p | SUN1 |
| hsa-miR-199a-5p | SOS2 |
| hsa-miR-199a-5p | C1GALT1 |
| hsa-miR-199a-5p | RAB9B |
| hsa-miR-199a-5p | MARCH8 |
| hsa-miR-199a-5p | NINL |
| hsa-miR-199a-5p | CACUL1 |
| hsa-miR-199a-5p | ZNF579 |
| hsa-miR-199a-5p | BICC1 |
| hsa-miR-199a-5p | UNG |
| hsa-miR-199a-5p | NAA40 |
| hsa-miR-199a-5p | CCNL1 |
| hsa-miR-199a-5p | GRB10 |
| hsa-miR-199a-5p | FAM188A |
| hsa-miR-199a-5p | ZFYVE27 |
| hsa-miR-199a-5p | GNG5 |
| hsa-miR-199a-5p | RALGAPA1 |
| hsa-miR-199a-5p | KCND3 |
| hsa-miR-199a-5p | HSPA5 |
| hsa-miR-199a-5p | LYSMD3 |
| hsa-miR-199a-5p | SNAI1 |
| hsa-miR-199a-5p | DPP8 |
| hsa-miR-199a-5p | NTNG1 |
| hsa-miR-199a-5p | AP1G1 |
| hsa-miR-199a-5p | GPRC5A |
| hsa-miR-199a-5p | CACNB2 |
| hsa-miR-199a-5p | PPP1R9A |
| hsa-miR-199a-5p | MYEF2 |
| hsa-miR-199a-5p | CCDC120 |
| hsa-miR-199a-5p | FZD4 |
| hsa-miR-199a-5p | SRRM1 |
| hsa-miR-199a-5p | PPARGC1A |
| hsa-miR-199a-5p | CLTC |
| hsa-miR-199a-5p | CDKN2AIP |
| hsa-miR-199a-5p | CSGALNACT1 |
| hsa-miR-199a-5p | EVX2 |
| hsa-miR-199a-5p | WNT2 |
| hsa-miR-199a-5p | MICAL3 |
| hsa-miR-199a-5p | BAAT |
| hsa-miR-199a-5p | GRIP1 |
| hsa-miR-199a-5p | IPO8 |
| hsa-miR-199a-5p | ABCC1 |
| hsa-miR-199a-5p | SACS |
| hsa-miR-199a-5p | JUNB |
| hsa-miR-199a-5p | MUC21 |
| hsa-miR-199a-5p | PAX3 |
| hsa-miR-199a-5p | CYLC2 |
| hsa-miR-199a-5p | CDCA7L |
| hsa-miR-199a-5p | RNF11 |
| hsa-miR-199a-5p | ACHE |
| hsa-miR-199a-5p | ZNF594 |
| hsa-miR-199a-5p | SORCS3 |
| hsa-miR-199a-5p | ETS1 |
| hsa-miR-199a-5p | HGF |
| hsa-miR-199a-5p | TMEM63B |
| hsa-miR-199a-5p | MARCH7 |
| hsa-miR-199a-5p | OSR1 |
| hsa-miR-199a-5p | GIT1 |
| hsa-miR-199a-5p | ZNF516 |
| hsa-miR-199a-5p | FPGS |
| hsa-miR-199a-5p | SAT1 |
| hsa-miR-199a-5p | LARGE |
| hsa-miR-199a-5p | NPAS2 |
| hsa-miR-199a-5p | ZCCHC4 |
| hsa-miR-199a-5p | PKN2 |
| hsa-miR-199a-5p | KPNA4 |
| hsa-miR-199a-5p | DRAM1 |
| hsa-miR-199a-5p | SLC9A8 |
| hsa-miR-199a-5p | HSPA12A |
| hsa-miR-199a-5p | RBM23 |
| hsa-miR-199a-5p | CAPRIN1 |
| hsa-miR-199a-5p | RLIM |
| hsa-miR-199a-5p | EXOC8 |
| hsa-miR-199a-5p | TSPAN3 |
| hsa-miR-199a-5p | KIAA1109 |
| hsa-miR-199a-5p | RANBP2 |
| hsa-miR-199a-5p | NAALADL2 |
| hsa-miR-199a-5p | PNPLA6 |
| hsa-miR-199a-5p | UHMK1 |
| hsa-miR-199a-5p | RAB27B |
| hsa-miR-199a-5p | BTBD3 |
| hsa-miR-199a-5p | TSPAN5 |
| hsa-miR-199a-5p | NAB2 |
| hsa-miR-199a-5p | RHEB |
| hsa-miR-199a-5p | CSDC2 |
| hsa-miR-199a-5p | ITGA8 |
| hsa-miR-199a-5p | TBC1D8 |
| hsa-miR-199a-5p | PVRL1 |
| hsa-miR-199a-5p | RTN4RL2 |
| hsa-miR-199a-5p | ZNF286B |
| hsa-miR-199a-5p | UBE2G1 |
| hsa-miR-199a-5p | VGLL2 |
| hsa-miR-199a-5p | FER |
| hsa-miR-199a-5p | PXN |
| hsa-miR-199a-5p | RBPMS |
| hsa-miR-199a-5p | ATXN7L1 |
| hsa-miR-199a-5p | UBAP1 |
| hsa-miR-199a-5p | PAN3 |
| hsa-miR-199a-5p | TBC1D14 |
| hsa-miR-199a-5p | DUSP14 |
| hsa-miR-199a-5p | MAPRE2 |
| hsa-miR-199a-5p | UBALD2 |
| hsa-miR-199a-5p | CHCHD4 |
| hsa-miR-199a-5p | CDKN1B |
| hsa-miR-199a-5p | PPFIBP1 |
| hsa-miR-199a-5p | EIF5B |
| hsa-miR-199a-5p | RAB21 |
| hsa-miR-199a-5p | PHACTR4 |
| hsa-miR-199a-5p | WDR44 |
| hsa-miR-199a-5p | CREBRF |
| hsa-miR-199a-5p | ANK3 |
| hsa-miR-199a-5p | ITGA3 |
| hsa-miR-199a-5p | TMEM215 |
| hsa-miR-199a-5p | ZNF629 |
| hsa-miR-199a-5p | CHN2 |
| hsa-miR-199a-5p | MCFD2 |
| hsa-miR-199a-5p | EHD4 |
| hsa-miR-199a-5p | MINK1 |
| hsa-miR-199a-5p | ZNF654 |
| hsa-miR-199a-5p | ACVR1B |
| hsa-miR-199a-5p | GSK3B |
| hsa-miR-199a-5p | E2F6 |
| hsa-miR-199a-5p | MFSD6 |
| hsa-miR-199a-5p | TTC9 |
| hsa-miR-199a-5p | DERL2 |
| hsa-miR-199a-5p | TAB3 |
| hsa-miR-199a-5p | FBXO30 |
| hsa-miR-199a-5p | CCBL2 |
| hsa-miR-199a-5p | NLK |
| hsa-miR-199a-5p | BROX |
| hsa-miR-199a-5p | ATP13A2 |
| hsa-miR-199a-5p | FIGN |
| hsa-miR-199a-5p | KIAA0040 |
| hsa-miR-199a-5p | OTX1 |
| hsa-miR-199a-5p | PAXBP1 |
| hsa-miR-199a-5p | PLXNC1 |
| hsa-miR-199a-5p | AQP11 |
| hsa-miR-199a-5p | HSPA4 |
| hsa-miR-199a-5p | CCDC88C |
| hsa-miR-199a-5p | PDE4D |
| hsa-miR-199a-5p | TOX3 |
| hsa-miR-199a-5p | APMAP |
| hsa-miR-199a-5p | STK4 |
| hsa-miR-199a-5p | FKBP5 |
| hsa-miR-199a-5p | TMED8 |
| hsa-miR-199a-5p | GPR180 |
| hsa-miR-199a-5p | EMC10 |
| hsa-miR-199a-5p | SIRT1 |
| hsa-miR-199a-5p | MARK4 |
| hsa-miR-199a-5p | KLHL29 |
| hsa-miR-199a-5p | SUCO |
| hsa-miR-199a-5p | FSTL4 |
| hsa-miR-199a-5p | BEND3 |
| hsa-miR-199a-5p | ZNF652 |
| hsa-miR-199a-5p | MIER3 |
| hsa-miR-199a-5p | ADD3 |
| hsa-miR-199a-5p | EMC7 |
| hsa-miR-199a-5p | NFIL3 |
| hsa-miR-199a-5p | GOSR1 |
| hsa-miR-199a-5p | ZNF667 |
| hsa-miR-199a-5p | PARP12 |
| hsa-miR-199a-5p | DDI2 |
| hsa-miR-199a-5p | MAP4K3 |
| hsa-miR-199a-5p | SLC35B3 |
| hsa-miR-199a-5p | SULT4A1 |
| hsa-miR-199a-5p | TM9SF3 |
| hsa-miR-199a-5p | BCAP29 |
| hsa-miR-199a-5p | CDKN1C |
| hsa-miR-199a-5p | KLHL23 |
| hsa-miR-199a-5p | CECR2 |
| hsa-miR-199a-5p | FUT9 |
| hsa-miR-199a-5p | NLRC3 |
| hsa-miR-199a-5p | KIAA0753 |
| hsa-miR-199a-5p | ATXN7 |
| hsa-miR-199a-5p | PMP22 |
| hsa-miR-199a-5p | ERLIN1 |
| hsa-miR-199a-5p | RNF38 |
| hsa-miR-199a-5p | WDTC1 |
| hsa-miR-199a-5p | XYLT1 |
| hsa-miR-199a-5p | RASSF3 |
| hsa-miR-199a-5p | WIPI2 |
| hsa-miR-199a-5p | DYRK1A |
| hsa-miR-199a-5p | NCSTN |
| hsa-miR-199a-5p | ZNF24 |
| hsa-miR-199a-5p | HLF |
| hsa-miR-199a-5p | TRAF3 |
| hsa-miR-199a-5p | STX4 |
| hsa-miR-199a-5p | RUNX1T1 |
| hsa-miR-199a-5p | DENND6A |
| hsa-miR-199a-5p | HOXA7 |
| hsa-miR-199a-5p | COL5A3 |
| hsa-miR-199a-5p | SEMA3F |
| hsa-miR-199a-5p | ATG4D |
| hsa-miR-199a-5p | DENND2C |
| hsa-miR-199a-5p | GPD2 |
| hsa-miR-199a-5p | PRPF40A |
| hsa-miR-199a-5p | TMEM245 |
| hsa-miR-199a-5p | CNN1 |
| hsa-miR-199a-5p | PPP4R1L |
| hsa-miR-199a-5p | ARHGAP17 |
| hsa-miR-199a-5p | PLXNA2 |
| hsa-miR-199a-5p | SEC24C |
| hsa-miR-199a-5p | FOXC2 |
| hsa-miR-199a-5p | JPH3 |
| hsa-miR-199a-5p | SNX6 |
| hsa-miR-199a-5p | ZBTB18 |
| hsa-miR-199a-5p | TET2 |
| hsa-miR-199a-5p | HIF1A |
| hsa-miR-199a-5p | APPBP2 |
| hsa-miR-199a-5p | ACTG1 |
| hsa-miR-199a-5p | TAOK1 |
| hsa-miR-199a-5p | ITFG3 |
| hsa-miR-199a-5p | USP27X |
| hsa-miR-199a-5p | TRMT1L |
| hsa-miR-199a-5p | ZNF784 |
| hsa-miR-199a-5p | MATN2 |
| hsa-miR-199a-5p | SET |
| hsa-miR-199a-5p | PRDM5 |
| hsa-miR-199a-5p | KIAA1958 |
| hsa-miR-199a-5p | PREPL |
| hsa-miR-199a-5p | USP37 |
| hsa-miR-199a-5p | CLK2 |
| hsa-miR-199a-5p | SMARCD1 |
| hsa-miR-199a-5p | DEPDC1B |
| hsa-miR-199a-5p | MINPP1 |
| hsa-miR-199a-5p | RBBP4 |
| hsa-miR-199a-5p | RORB |
| hsa-miR-199a-5p | DDX3Y |
| hsa-miR-199a-5p | THUMPD3 |
| hsa-miR-199a-5p | ACER2 |
| hsa-miR-199a-5p | IKBKB |
| hsa-miR-199a-5p | ZNF618 |
| hsa-miR-199a-5p | ZNF641 |
| hsa-miR-199a-5p | BTRC |
| hsa-miR-199a-5p | SLC24A4 |
| hsa-miR-199a-5p | TMEM135 |
| hsa-miR-199a-5p | ZBTB37 |
| hsa-miR-199a-5p | SMARCAD1 |
| hsa-miR-199a-5p | CLIP1 |
| hsa-miR-199a-5p | TRPM4 |
| hsa-miR-199a-5p | FXR1 |
| hsa-miR-199a-5p | LAMC1 |
| hsa-miR-199a-5p | EPB41L3 |
| hsa-miR-199a-5p | FAM169A |
| hsa-miR-199a-5p | SLC35E1 |
| hsa-miR-199a-5p | FBXO33 |
| hsa-miR-199a-5p | RGS17 |
| hsa-miR-199a-5p | ZMAT2 |
| hsa-miR-199a-5p | TIGD5 |
| hsa-miR-199a-5p | XPOT |
| hsa-miR-199a-5p | SRGAP3 |
| hsa-miR-199a-5p | RFX3 |
| hsa-miR-199a-5p | STAG1 |
| hsa-miR-199a-5p | CLCN3 |
| hsa-miR-199a-5p | ZCCHC2 |
| hsa-miR-199a-5p | VEGFA |
| hsa-miR-199a-5p | MN1 |
| hsa-miR-199a-5p | HSPBAP1 |
| hsa-miR-199a-5p | STRADB |
| hsa-miR-199a-5p | GMEB1 |
| hsa-miR-199a-5p | EPHA7 |
| hsa-miR-199a-5p | CLOCK |
| hsa-miR-199a-5p | PI4KA |
| hsa-miR-199a-5p | UBE2Q1 |
| hsa-miR-199a-5p | SORL1 |
| hsa-miR-199a-5p | SRRM4 |
| hsa-miR-199a-5p | OSTM1 |
| hsa-miR-199a-5p | NOTUM |
| hsa-miR-199a-5p | ETV6 |
| hsa-miR-199a-5p | PPP6C |
| hsa-miR-199a-5p | MYH9 |
| hsa-miR-199a-5p | ZNF415 |
| hsa-miR-199a-5p | MINOS1-NBL1 |
| hsa-miR-199a-5p | ITGA4 |
| hsa-miR-199a-5p | HHIP |
| hsa-miR-199a-5p | NBL1 |
| hsa-miR-199a-5p | ZNF148 |
| hsa-miR-199a-5p | FAM76B |
| hsa-miR-199a-5p | SLAMF8 |
| hsa-miR-199a-5p | AUTS2 |
| hsa-miR-199a-5p | ONECUT2 |
| hsa-miR-199a-5p | SLC25A37 |
| hsa-miR-199a-5p | RGMA |
| hsa-miR-199a-5p | ZFP91 |
| hsa-miR-199a-5p | PPM1L |
| hsa-miR-199a-5p | ZNF394 |
| hsa-miR-199a-5p | ARHGEF5 |
| hsa-miR-199a-5p | FSD1 |
| hsa-miR-199a-5p | ROCK1 |
| hsa-miR-199a-5p | KANK2 |
| hsa-miR-199a-5p | SOX4 |
| hsa-miR-199a-5p | ARHGAP29 |
| hsa-miR-199a-5p | RRAGC |
| hsa-miR-199a-5p | SNN |
| hsa-miR-199a-5p | RFESD |
| hsa-miR-199a-5p | KLF9 |
| hsa-miR-199a-5p | AFTPH |
| hsa-miR-199a-5p | SLC24A2 |
| hsa-miR-199a-5p | NUPL1 |
| hsa-miR-199a-5p | ARHGEF12 |
| hsa-miR-199a-5p | AGO1 |
| hsa-miR-199a-5p | CAV1 |
| hsa-miR-199a-5p | EXOSC3 |
| hsa-miR-199a-5p | ZSWIM4 |
| hsa-miR-199a-5p | ERBB4 |
| hsa-miR-199a-5p | MYO5A |
| hsa-miR-199a-5p | ZBTB8A |
| hsa-miR-199a-5p | GBP1 |
| hsa-miR-199a-5p | PCYOX1 |
| hsa-miR-199a-5p | PAOX |
| hsa-miR-199a-5p | SLC35E4 |
| hsa-miR-199a-5p | SH3PXD2A |
| hsa-miR-199a-5p | WBP11 |
| hsa-miR-199a-5p | PLEKHH1 |
| hsa-miR-199a-5p | MGAT3 |
| hsa-miR-199a-5p | RPS6KA5 |
| hsa-miR-199a-5p | TMEM123 |
| hsa-miR-199a-5p | RGMB |
| hsa-miR-199a-5p | MFHAS1 |
| hsa-miR-199a-5p | JAG1 |
| hsa-miR-199a-5p | DNAJB5 |
| hsa-miR-199a-5p | STOX2 |
| hsa-miR-199a-5p | SLC35A3 |
| hsa-miR-199a-5p | PDE7A |
| hsa-miR-199a-5p | YAF2 |
| hsa-miR-199a-5p | MPP5 |
| hsa-miR-199a-5p | STON2 |
| hsa-miR-199a-5p | FAM126B |
| hsa-miR-199a-5p | TERF2 |
| hsa-miR-199a-5p | MAP3K5 |
| hsa-miR-199a-5p | ZNF704 |
| hsa-miR-199a-5p | ZNF710 |
| hsa-miR-199a-5p | POU3F1 |
| hsa-miR-199a-5p | BTBD9 |
| hsa-miR-199a-5p | LRP4 |
| hsa-miR-199a-5p | KPNB1 |
| hsa-miR-199a-5p | PBRM1 |
| hsa-miR-199a-5p | ABCA1 |
| hsa-miR-199a-5p | NFE2L1 |
| hsa-miR-199a-5p | RIMS1 |
| hsa-miR-199a-5p | E2F3 |
| hsa-miR-199a-5p | ATXN3 |
| hsa-miR-199a-5p | C18orf25 |
| hsa-miR-199a-5p | ARIH2 |
| hsa-miR-199a-5p | HOXB6 |
| hsa-miR-199a-5p | TENM2 |
| hsa-miR-199a-5p | RB1 |
| hsa-miR-199a-5p | NAA15 |
| hsa-miR-199a-5p | RAD51L3-RFFL |
| hsa-miR-199a-5p | USP46 |
| hsa-miR-199a-5p | WAPAL |
| hsa-miR-199a-5p | MIOS |
| hsa-miR-199a-5p | ADAMTS5 |
| hsa-miR-199a-5p | PHTF2 |
| hsa-miR-199a-5p | RNMTL1 |
| hsa-miR-199a-5p | BRD7 |
| hsa-miR-199a-5p | ATP1A2 |
| hsa-miR-199a-5p | CDH2 |
| hsa-miR-199a-5p | MANEAL |
| hsa-miR-199a-5p | REEP2 |
| hsa-miR-199a-5p | PAK4 |
| hsa-miR-199a-5p | R3HDM1 |
| hsa-miR-199a-5p | ST6GAL1 |
| hsa-miR-199a-5p | RFFL |
| hsa-miR-199a-5p | ARHGAP19 |
| hsa-miR-199a-5p | SMARCE1 |
| hsa-miR-199a-5p | ZNF512B |
| hsa-miR-199a-5p | WNT7B |
| hsa-miR-199a-5p | ZEB1 |
| hsa-miR-199a-5p | ULK3 |
| hsa-miR-199a-5p | MTMR3 |
| hsa-miR-199a-5p | CDH8 |
| hsa-miR-199a-5p | RANBP10 |
| hsa-miR-199a-5p | WNK3 |
| hsa-miR-199a-5p | KIT |
| hsa-miR-199a-5p | KIAA0355 |
| hsa-miR-199a-5p | CRIM1 |
| hsa-miR-199a-5p | CBL |
| hsa-miR-199a-5p | SRSF1 |
| hsa-miR-199a-5p | MEF2D |
| hsa-miR-199a-5p | POGK |
| hsa-miR-199a-5p | SP2 |
| hsa-miR-199a-5p | COL8A1 |
| hsa-miR-199a-5p | CD276 |
| hsa-miR-199a-5p | CSRNP2 |
| hsa-miR-199a-5p | GANAB |
| hsa-miR-199a-5p | ZNF226 |
| hsa-miR-199a-5p | ATP2B2 |
| hsa-miR-199a-5p | AL590483.1 |
| hsa-miR-199a-5p | ARID2 |
| hsa-miR-199a-5p | MAX |
| hsa-miR-199a-5p | HAS2 |
| hsa-miR-199a-5p | BCL7A |
| hsa-miR-199a-5p | NFYA |
| hsa-miR-199a-5p | KLF12 |
| hsa-miR-199a-5p | NIPAL3 |
| hsa-miR-199a-5p | TXNRD1 |
| hsa-miR-199a-5p | PPM1B |
| hsa-miR-199a-5p | CTNNA2 |
| hsa-miR-199a-5p | CEP72 |
| hsa-miR-199a-5p | UBN2 |
| hsa-miR-199a-5p | CDC73 |
| hsa-miR-199a-5p | SP1 |
| hsa-miR-199a-5p | EXTL3 |
| hsa-miR-199a-5p | CCDC176 |
| hsa-miR-199a-5p | NUFIP2 |
| hsa-miR-199a-5p | GPR124 |
| hsa-miR-199a-5p | KLHL6 |
| hsa-miR-199a-5p | CEP85L |
| hsa-miR-199a-5p | PPP1R12A |
| hsa-miR-199a-5p | ACVR2B |
| hsa-miR-199a-5p | LASP1 |
| hsa-miR-199a-5p | GRIK3 |
| hsa-miR-199a-5p | MBD5 |
| hsa-miR-199a-5p | PURA |
| hsa-miR-199a-5p | HEATR3 |
| hsa-miR-199a-5p | MAPK4 |
| hsa-miR-199a-5p | MAP2 |
| hsa-miR-199a-5p | KIAA2018 |
| hsa-miR-199a-5p | RBM24 |
| hsa-miR-199a-5p | DLC1 |
| hsa-miR-199a-5p | KLHL3 |
| hsa-miR-199a-5p | TIMM10 |
| hsa-miR-199a-5p | TMEM178B |
| hsa-miR-199a-5p | UBQLN4 |
| hsa-miR-199a-5p | RANBP3 |
| hsa-miR-199a-5p | PLXND1 |
| hsa-miR-199a-5p | HDLBP |
| hsa-miR-199a-5p | RIN2 |
| hsa-miR-199a-5p | FBXO9 |
| hsa-miR-199a-5p | ARHGAP20 |
| hsa-miR-199a-5p | CSNK1D |
| hsa-miR-199a-5p | SPRED3 |
| hsa-miR-199a-5p | AKAP13 |
| hsa-miR-199a-5p | SLC46A1 |
| hsa-miR-199a-5p | SPRYD7 |
| hsa-miR-199a-5p | USP19 |
| hsa-miR-199a-5p | ZBTB5 |
| hsa-miR-199a-5p | RRP15 |
| hsa-miR-199a-5p | NCOR1 |
| hsa-miR-199a-5p | SOX6 |
| hsa-miR-199a-5p | ABCC5 |
| hsa-miR-199a-5p | ANKRD52 |
| hsa-miR-199a-5p | INO80D |
| hsa-miR-199a-5p | KIAA0226 |
| hsa-miR-199a-5p | ZNF740 |
| hsa-miR-199a-5p | ZNF614 |
| hsa-miR-199a-5p | SMARCA4 |
| hsa-miR-199a-5p | PNP |
| hsa-miR-199a-5p | KIAA1324L |
| hsa-miR-199a-5p | SRL |
| hsa-miR-199a-5p | HIP1R |
| hsa-miR-199a-5p | PTGIS |
| hsa-miR-199a-5p | PPL |
| hsa-miR-199a-5p | SLC36A4 |
| hsa-miR-199a-5p | NOTO |
| hsa-miR-199a-5p | M6PR |
| hsa-miR-199a-5p | ACVR2A |
| hsa-miR-199a-5p | DDX3X |
| hsa-miR-199a-5p | RAB10 |
| hsa-miR-199a-5p | ANKRD13C |
| hsa-miR-199a-5p | NCOA2 |
| hsa-miR-199a-5p | ETS2 |
| hsa-miR-199a-5p | RGS10 |
| hsa-miR-199a-5p | MAML3 |
| hsa-miR-199a-5p | PLXNB1 |
| hsa-miR-199a-5p | DESI1 |
| hsa-miR-199a-5p | PRDM16 |
| hsa-miR-199a-5p | ATG14 |
| hsa-miR-199a-5p | AGO3 |
| hsa-miR-199a-5p | LIMD2 |
| hsa-miR-199a-5p | AMN |
| hsa-miR-199a-5p | PARVA |
| hsa-miR-199a-5p | SNTB1 |
| hsa-miR-199a-5p | DNAH3 |
| hsa-miR-199a-5p | DST |
| hsa-miR-199a-5p | FAM179A |
| hsa-miR-199a-5p | GATAD2B |
| hsa-miR-199a-5p | STRN |
| hsa-miR-199a-5p | BLCAP |
| hsa-miR-199a-5p | LMAN2 |
| hsa-miR-199a-5p | RAD54L2 |
| hsa-miR-199a-5p | WDR76 |
| hsa-miR-199a-5p | ZNF706 |
| hsa-miR-199a-5p | CNOT6 |
| hsa-miR-199a-5p | PRR14L |
| hsa-miR-199a-5p | MKL2 |
| hsa-miR-199a-5p | ADRBK2 |
| hsa-miR-199a-5p | TLN1 |
| hsa-miR-199a-5p | SAR1A |
| hsa-miR-199a-5p | TBL1XR1 |
| hsa-miR-199a-5p | FANCA |
| hsa-miR-199a-5p | ZDHHC21 |
| hsa-miR-199a-5p | MRPS25 |
| hsa-miR-199a-5p | RGP1 |
| hsa-miR-199a-5p | SERPINE1 |
| hsa-miR-199a-5p | PCDH17 |
| hsa-miR-199a-5p | ABTB2 |
| hsa-miR-199a-5p | ADAMTSL3 |
| hsa-miR-199a-5p | TANC2 |
| hsa-miR-199a-5p | HIF1AN |
| hsa-miR-199a-5p | USP31 |
| hsa-miR-199a-5p | RSF1 |
| hsa-miR-199a-5p | TENM4 |
| hsa-miR-199a-5p | MON1B |
| hsa-miR-199a-5p | CNOT6L |
| hsa-miR-199a-5p | DONSON |
| hsa-miR-199a-5p | PACRGL |
| hsa-miR-199a-5p | PTPN9 |
| hsa-miR-199a-5p | C17orf85 |
| hsa-miR-199a-5p | CA12 |
| hsa-miR-199a-5p | CABP7 |
| hsa-miR-199a-5p | BTG1 |
| hsa-miR-199a-5p | ZBED4 |
| hsa-miR-199a-5p | ZNF480 |
| hsa-miR-199a-5p | CBX5 |
| hsa-miR-199a-5p | GRHPR |
| hsa-miR-199a-5p | NCKAP1 |
| hsa-miR-199a-5p | IST1 |
| hsa-miR-199a-5p | GINM1 |
| hsa-miR-199a-5p | PTPMT1 |
| hsa-miR-199a-5p | INTS8 |
| hsa-miR-199a-5p | EIF2B2 |
| hsa-miR-199a-5p | TSPYL1 |
| hsa-miR-199a-5p | YIPF6 |
| hsa-miR-199a-5p | MAPK8 |
| hsa-miR-199a-5p | FCHSD1 |
| hsa-miR-199a-5p | TSR2 |
| hsa-miR-199a-5p | AHSA2 |
| hsa-miR-199a-5p | ZBTB20 |
| hsa-miR-199a-5p | ITCH |
| hsa-miR-199a-5p | YIPF2 |
| hsa-miR-199a-5p | SWAP70 |
| hsa-miR-199a-5p | C15orf61 |
| hsa-miR-199a-5p | AIP |
| hsa-miR-199a-5p | TRIP4 |
| hsa-miR-199a-5p | MRPL22 |
| hsa-miR-199a-5p | P2RX7 |
| hsa-miR-199a-5p | ZNF703 |
| hsa-miR-199a-5p | DDX5 |
| hsa-miR-199a-5p | DDX6 |
| hsa-miR-199a-5p | ZC2HC1C |
| hsa-let-7d-3p | TFRC |
| hsa-let-7d-3p | GSTK1 |
| hsa-let-7d-3p | GNAO1 |
| hsa-let-7d-3p | NKX6-1 |
| hsa-let-7d-3p | SIGLEC6 |
| hsa-let-7d-3p | PDGFC |
| hsa-let-7d-3p | PARP11 |
| hsa-let-7d-3p | GSX2 |
| hsa-let-7d-3p | HMGA2 |
| hsa-let-7d-3p | PRKACB |
| hsa-let-7d-3p | BEND2 |
| hsa-let-7d-3p | NXT2 |
| hsa-let-7d-3p | GCSAML |
| hsa-let-7d-3p | SNTN |
| hsa-let-7d-3p | NARS |
| hsa-let-7d-3p | KLHL31 |
| hsa-let-7d-3p | PTGIS |
| hsa-let-7d-3p | ABCA8 |
| hsa-let-7d-3p | SH3RF1 |
| hsa-let-7d-3p | KANSL1 |
| hsa-let-7d-3p | KCND3 |
| hsa-let-7d-3p | ZNF782 |
| hsa-let-7d-3p | ZNF486 |
| hsa-let-7d-3p | EIF3E |
| hsa-let-7d-3p | CTSV |
| hsa-let-7d-3p | LSR |
| hsa-let-7d-3p | CDH19 |
| hsa-let-7d-3p | FSD2 |
| hsa-let-7d-3p | SRP19 |
| hsa-let-7d-3p | EXOSC7 |
| hsa-let-7d-3p | ZNF672 |
| hsa-let-7d-3p | TMEM163 |
| hsa-let-7d-3p | DGKI |
| hsa-let-7d-3p | FAM219A |
| hsa-let-7d-3p | RIBC2 |
| hsa-let-7d-3p | CYP27C1 |
| hsa-let-7d-3p | ARL14 |
| hsa-let-7d-3p | ZIC1 |
| hsa-let-7d-3p | ZNF611 |
| hsa-let-7d-3p | UFM1 |
| hsa-let-7d-3p | DSC1 |
| hsa-let-7d-3p | CNIH1 |
| hsa-let-7d-3p | CLEC3A |
| hsa-let-7d-3p | TLR4 |
| hsa-let-7d-3p | NOM1 |
| hsa-let-7d-3p | C1orf145 |
| hsa-let-7d-3p | MBTD1 |
| hsa-let-7d-3p | GRPEL2 |
| hsa-let-7d-3p | GLYAT |
| hsa-let-7d-3p | CDNF |
| hsa-let-7d-3p | TBC1D5 |
| hsa-let-7d-3p | PSMD10 |
| hsa-let-7d-3p | ADH7 |
| hsa-let-7d-3p | NRG3 |
| hsa-let-7d-3p | LINC00999 |
| hsa-let-7d-3p | TMEM26 |
| hsa-let-7d-3p | BEND6 |
| hsa-let-7d-3p | TMEM231 |
| hsa-let-7d-3p | F2RL2 |
| hsa-let-7d-3p | RCAN1 |
| hsa-let-7d-3p | NUP85 |
| hsa-let-7d-3p | ZNF623 |
| hsa-let-7d-3p | ZNF90 |
| hsa-let-7d-3p | CCDC6 |
| hsa-let-7d-3p | CDKL4 |
| hsa-let-7d-3p | NCMAP |
| hsa-let-7d-3p | A4GNT |
| hsa-let-7d-3p | SNRPF |
| hsa-let-7d-3p | GLIPR1 |
| hsa-let-7d-3p | ZSCAN12 |
| hsa-let-7d-3p | TMEM117 |
| hsa-let-7d-3p | F11 |
| hsa-let-7d-3p | ZNF841 |
| hsa-let-7d-3p | GRB2 |
| hsa-let-7d-3p | C7orf41 |
| hsa-let-7d-3p | ISCA2 |
| hsa-let-7d-3p | SDPR |
| hsa-let-7d-3p | FYTTD1 |
| hsa-let-7d-3p | CDH11 |
| hsa-let-7d-3p | CHRNA9 |
| hsa-let-7d-3p | TFAM |
| hsa-let-7d-3p | CT62 |
| hsa-let-7d-3p | FAM193B |
| hsa-let-7d-3p | ARHGEF10 |
| hsa-let-7d-3p | RLN2 |
| hsa-let-7d-3p | RAD52 |
| hsa-let-7d-3p | GAS1 |
| hsa-let-7d-3p | OTUD6A |
| hsa-let-7d-3p | SLC17A4 |
| hsa-let-7d-3p | PPP4R1 |
| hsa-let-7d-3p | LHX1 |
| hsa-let-7d-3p | DTNA |
| hsa-let-7d-3p | LPL |
| hsa-let-7d-3p | EXOC8 |
| hsa-let-7d-3p | FUBP3 |
| hsa-let-7d-3p | RETSAT |
| hsa-let-7d-3p | ZNF331 |
| hsa-let-7d-3p | RGS7BP |
| hsa-let-7d-3p | C11orf44 |
| hsa-let-7d-3p | SLC13A1 |
| hsa-let-7d-3p | TTC26 |
| hsa-let-7d-3p | AC022498.1 |
| hsa-let-7d-3p | COX19 |
| hsa-let-7d-3p | MSH3 |
| hsa-let-7d-3p | CEND1 |
| hsa-let-7d-3p | CASD1 |
| hsa-let-7d-3p | CMTM6 |
| hsa-let-7d-3p | IL1RN |
| hsa-let-7d-3p | HPGD |
| hsa-let-7d-3p | TDRP |
| hsa-let-7d-3p | GPR88 |
| hsa-let-7d-3p | RASSF8 |
| hsa-let-7d-3p | PLD5 |
| hsa-let-7d-3p | SSH3 |
| hsa-let-7d-3p | RBFOX1 |
| hsa-let-7d-3p | AUTS2 |
| hsa-let-7d-3p | SIDT1 |
| hsa-let-7d-3p | GPX8 |
| hsa-let-7d-3p | ZIK1 |
| hsa-let-7d-3p | PM20D2 |
| hsa-let-7d-3p | ATMIN |
| hsa-let-7d-3p | NFASC |
| hsa-let-7d-3p | PRKAR1A |
| hsa-let-7d-3p | ARL15 |
| hsa-let-7d-3p | CTU1 |
| hsa-let-7d-3p | AL033381.1 |
| hsa-let-7d-3p | CNGB3 |
| hsa-let-7d-3p | ZNF616 |
| hsa-let-7d-3p | VEZF1 |
| hsa-let-7d-3p | ATG16L1 |
| hsa-let-7d-3p | EYA1 |
| hsa-let-7d-3p | TMPRSS11D |
| hsa-let-7d-3p | TRIM64B |
| hsa-let-7d-3p | YY1 |
| hsa-let-7d-3p | XRN1 |
| hsa-let-7d-3p | NUDT19 |
| hsa-let-7d-3p | ZCCHC6 |
| hsa-let-7d-3p | IL1RL1 |
| hsa-let-7d-3p | ZNF24 |
| hsa-let-7d-3p | LRIT2 |
| hsa-let-7d-3p | GOLGA8G |
| hsa-let-7d-3p | ERC2 |
| hsa-let-7d-3p | PRICKLE3 |
| hsa-let-7d-3p | PEX5L |
| hsa-let-7d-3p | PTAR1 |
| hsa-let-7d-3p | GOLGA8F |
| hsa-let-7d-3p | AL117190.3 |
| hsa-let-7d-3p | CLEC5A |
| hsa-let-7d-3p | KLF6 |
| hsa-let-7d-3p | URM1 |
| hsa-let-7d-3p | MTMR6 |
| hsa-let-7d-3p | KCNT2 |
| hsa-let-7d-3p | EYS |
| hsa-let-7d-3p | SPOPL |
| hsa-let-7d-3p | ZNF605 |
| hsa-let-7d-3p | CLDN16 |
| hsa-let-7d-3p | GATA4 |
| hsa-let-7d-3p | ZFP69B |
| hsa-let-7d-3p | KIAA0754 |
| hsa-let-7d-3p | LETMD1 |
| hsa-let-7d-3p | CEP170 |
| hsa-let-7d-3p | ACTR1B |
| hsa-let-7d-3p | SLC36A1 |
| hsa-let-7d-3p | FAM133B |
| hsa-let-7d-3p | SPRY3 |
| hsa-let-7d-3p | GAS7 |
| hsa-let-7d-3p | FMR1 |
| hsa-let-7d-3p | C3orf72 |
| hsa-let-7d-3p | RAB2B |
| hsa-let-7d-3p | TTC9 |
| hsa-let-7d-3p | RSPH10B |
| hsa-let-7d-3p | WDR82 |
| hsa-let-7d-3p | IMPG1 |
| hsa-let-7d-3p | BCL10 |
| hsa-let-7d-3p | PWP1 |
| hsa-let-7d-3p | MYO9A |
| hsa-let-7d-3p | GTF3C3 |
| hsa-let-7d-3p | APPL1 |
| hsa-let-7d-3p | ZNF302 |
| hsa-let-7d-3p | AMMECR1 |
| hsa-let-7d-3p | PI15 |
| hsa-let-7d-3p | COMMD2 |
| hsa-let-7d-3p | AHCTF1 |
| hsa-let-7d-3p | BNC1 |
| hsa-let-7d-3p | DGKB |
| hsa-let-7d-3p | SS18L1 |
| hsa-let-7d-3p | MESDC2 |
| hsa-let-7d-3p | GLS |
| hsa-let-7d-3p | LSM11 |
| hsa-let-7d-3p | GOLGA6L9 |
| hsa-let-7d-3p | AP3D1 |
| hsa-let-7d-3p | SH3BP5 |
| hsa-let-7d-3p | SMCHD1 |
| hsa-let-7d-3p | KCTD15 |
| hsa-let-7d-3p | ANKRD34C |
| hsa-let-7d-3p | METTL6 |
| hsa-let-7d-3p | GOLGA6L10 |
| hsa-let-7d-3p | PHC1 |
| hsa-let-7d-3p | JHDM1D |
| hsa-let-7d-3p | GOLGA6L9 |
| hsa-let-7d-3p | PDCL |
| hsa-let-7d-3p | GOLGA6L4 |
| hsa-let-7d-3p | ZNF532 |
| hsa-let-7d-3p | HEATR1 |
| hsa-let-7d-3p | CD47 |
| hsa-let-7d-3p | TNFAIP8L1 |
| hsa-let-7d-3p | GPR107 |
| hsa-let-7d-3p | RP13-996F3.5 |
| hsa-let-7d-3p | BCAT1 |
| hsa-let-7d-3p | PDE2A |
| hsa-let-7d-3p | PHOX2B |
| hsa-let-7d-3p | FAM230A |
| hsa-let-7d-3p | PPAPDC2 |
| hsa-let-7d-3p | PHYHIPL |
| hsa-let-7d-3p | PIGN |
| hsa-let-7d-3p | NUP37 |
| hsa-let-7d-3p | FUT11 |
| hsa-let-7d-3p | SLC6A6 |
| hsa-let-7d-3p | ABCA1 |
| hsa-let-7d-3p | RGL1 |
| hsa-let-7d-3p | SLC4A4 |
| hsa-let-7d-3p | MYO1H |
| hsa-let-7d-3p | KLF9 |
| hsa-let-7d-3p | NRF1 |
| hsa-let-7d-3p | ALDH3A2 |
| hsa-let-7d-3p | ARRDC4 |
| hsa-let-7d-3p | C3orf70 |
| hsa-let-7d-3p | PLCE1 |
| hsa-let-7d-3p | TMEM170A |
| hsa-let-7d-3p | ASRGL1 |
| hsa-let-7d-3p | ABCA10 |
| hsa-let-7d-3p | OCLN |
| hsa-let-7d-3p | ABCC4 |
| hsa-let-7d-3p | MARCH6 |
| hsa-let-7d-3p | ZNF71 |
| hsa-let-7d-3p | TMED7-TICAM2 |
| hsa-let-7d-3p | MAVS |
| hsa-let-7d-3p | FLVCR1 |
| hsa-let-7d-3p | SKA1 |
| hsa-let-7d-3p | TICAM2 |
| hsa-let-7d-3p | NECAB1 |
| hsa-let-7d-3p | TET3 |
| hsa-let-7d-3p | FNDC3A |
| hsa-let-7d-3p | MCTP1 |
| hsa-let-7d-3p | MLLT3 |
| hsa-let-7d-3p | DCUN1D3 |
| hsa-let-7d-3p | BSN |
| hsa-let-7d-3p | SEMA5A |
| hsa-let-7d-3p | GAB1 |
| hsa-let-7d-3p | GABRG1 |
| hsa-let-7d-3p | RP11-766F14.2 |
| hsa-let-7d-3p | PTPN4 |
| hsa-let-7d-3p | CTD-2368P22.1 |
| hsa-let-7d-3p | ZBTB21 |
| hsa-let-7d-3p | AAK1 |
| hsa-let-7d-3p | GTF3C4 |
| hsa-let-7d-3p | SERINC3 |
| hsa-let-7d-3p | ADAM12 |
| hsa-let-7d-3p | TBL1X |
| hsa-let-7d-3p | ZNF264 |
| hsa-let-7d-3p | RAB27B |
| hsa-let-7d-3p | CXCR5 |
| hsa-let-7d-3p | BTBD7 |
| hsa-let-7d-3p | MFSD8 |
| hsa-let-7d-3p | CREG2 |
| hsa-let-7d-3p | ZNF85 |
| hsa-let-7d-3p | GBP1 |
| hsa-let-7d-3p | PTPRD |
| hsa-let-7d-3p | LPP |
| hsa-let-7d-3p | HOOK3 |
| hsa-let-7d-3p | GRSF1 |
| hsa-let-7d-3p | INO80D |
| hsa-let-7d-3p | ZBTB3 |
| hsa-let-7d-3p | RIF1 |
| hsa-let-7d-3p | CYP20A1 |
| hsa-let-7d-3p | ERBB4 |
| hsa-let-7d-3p | SLC15A2 |
| hsa-let-7d-3p | CELF1 |
| hsa-let-7d-3p | KLF12 |
| hsa-let-7d-3p | SGMS2 |
| hsa-let-7d-3p | CCNL2 |
| hsa-let-7d-3p | HOOK1 |
| hsa-let-7d-3p | PRSS12 |
| hsa-let-7d-3p | URB2 |
| hsa-let-7d-3p | PRICKLE1 |
| hsa-let-7d-3p | KLB |
| hsa-let-7d-3p | ZNF417 |
| hsa-let-7d-3p | TIGD7 |
| hsa-let-7d-3p | QKI |
| hsa-let-7d-3p | ZNF845 |
| hsa-let-7d-3p | THAP2 |
| hsa-let-7d-3p | UHRF1BP1L |
| hsa-let-7d-3p | PDGFD |
| hsa-let-7d-3p | NBEAL1 |
| hsa-let-7d-3p | LMBR1 |
| hsa-let-7d-3p | CHSY1 |
| hsa-let-7d-3p | IL2RB |
| hsa-let-7d-3p | KCNH5 |
| hsa-let-7d-3p | PTBP2 |
| hsa-let-7d-3p | TRPC3 |
| hsa-let-7d-3p | CTBP2 |
| hsa-let-7d-3p | RNF157 |
| hsa-let-7d-3p | THBS2 |
| hsa-let-7d-3p | CHCHD4 |
| hsa-let-7d-3p | WRN |
| hsa-let-7d-3p | AGO3 |
| hsa-let-7d-3p | NEDD4L |
| hsa-let-7d-3p | ZC3H12B |
| hsa-let-7d-3p | PPCDC |
| hsa-let-7d-3p | FAM20B |
| hsa-let-7d-3p | EFCAB6 |
| hsa-let-7d-3p | GPR133 |
| hsa-let-7d-3p | TMEM30A |
| hsa-let-7d-3p | C12orf5 |
| hsa-let-7d-3p | LDLRAD4 |
| hsa-let-7d-3p | PHF14 |
| hsa-let-7d-3p | TMPPE |
| hsa-let-7d-3p | ZNF804B |
| hsa-let-7d-3p | SIT1 |
| hsa-let-7d-3p | METTL14 |
| hsa-let-7d-3p | VPS35 |
| hsa-let-7d-3p | SLC30A7 |
| hsa-let-7d-3p | GXYLT2 |
| hsa-let-7d-3p | KLF8 |
| hsa-let-7d-3p | ASXL2 |
| hsa-let-7d-3p | ADAM22 |
| hsa-let-7d-3p | MOB4 |
| hsa-let-7d-3p | GLTSCR1L |
| hsa-let-7d-3p | EMR2 |
| hsa-let-7d-3p | LRRC3DN |
| hsa-let-7d-3p | C2orf72 |
| hsa-let-7d-3p | LRIG2 |
| hsa-let-7d-3p | SHF |
| hsa-let-7d-3p | RRP15 |
| hsa-let-7d-3p | ANKRD9 |
| hsa-let-7d-3p | KLHL15 |
| hsa-let-7d-3p | PHC3 |
| hsa-let-7d-3p | LIFR |
| hsa-let-7d-3p | CALU |
| hsa-let-7d-3p | ACVR2A |
| hsa-let-7d-3p | ZZZ3 |
| hsa-let-7d-3p | C11orf1 |
| hsa-let-7d-3p | DMRTA1 |
| hsa-let-7d-3p | HSPE1-MOB4 |
| hsa-let-7d-3p | STAMBP |
| hsa-let-7d-3p | RIMKLA |
| hsa-let-7d-3p | HACE1 |
| hsa-let-7d-3p | ZMYND11 |
| hsa-let-7d-3p | MLXIP |
| hsa-let-7d-3p | TATDN3 |
| hsa-let-7d-3p | REPS2 |
| hsa-let-7d-3p | HNMT |
| hsa-let-7d-3p | BDNF |
| hsa-let-7d-3p | MMADHC |
| hsa-let-7d-3p | SHC4 |
| hsa-let-7d-3p | GFM1 |
| hsa-let-7d-3p | C2CD4A |
| hsa-let-7d-3p | IRS4 |
| hsa-let-7d-3p | XPOT |
| hsa-let-7d-3p | COL12A1 |
| hsa-let-7d-3p | CFL2 |
| hsa-let-7d-3p | SNRPD3 |
| hsa-let-7d-3p | CDH7 |
| hsa-let-7d-3p | YME1L1 |
| hsa-let-7d-3p | CMSS1 |
| hsa-let-7d-3p | KDM5C |
| hsa-let-7d-3p | CCSER2 |
| hsa-let-7d-3p | GSR |
| hsa-let-7d-3p | VPS8 |
| hsa-let-7d-3p | URB1 |
| hsa-let-7d-3p | TBRG4 |
| hsa-let-7d-3p | ESF1 |
| hsa-let-7d-3p | CA5B |
| hsa-let-7d-3p | WDR5B |
| hsa-let-7d-3p | DIS3 |
| hsa-let-7d-3p | ZC3H14 |
| hsa-let-7d-3p | PCSK9 |
| hsa-let-7d-3p | TTL |
| hsa-let-7d-3p | TTLL7 |
| hsa-let-7d-3p | ABCG1 |
| hsa-let-7d-3p | TRIM27 |
| hsa-let-7d-3p | SOD2 |
| hsa-let-7d-3p | TIMM9 |
| hsa-let-7d-3p | CNKSR3 |
| hsa-let-7d-3p | ARSG |
| hsa-let-7d-3p | CPSF6 |
| hsa-let-7d-3p | UGT3A1 |
| hsa-let-7d-3p | ANKRD13C |
| hsa-let-7d-3p | PAK1IP1 |
| hsa-let-7d-3p | GEMIN4 |
| hsa-let-7d-3p | MAML2 |
| hsa-let-7d-3p | FUNDC2 |
| hsa-let-7d-3p | IMP4 |
| hsa-let-7d-3p | VGLL3 |
| hsa-let-7d-3p | MPC2 |
| hsa-let-7d-3p | PLA2G12A |
| hsa-let-7d-3p | MYO1E |
| hsa-let-7d-3p | C1orf27 |
| hsa-let-7d-3p | ITM2B |
| hsa-let-7d-3p | XPR1 |
| hsa-let-7d-3p | TP53RK |
| hsa-let-7d-3p | TSPAN14 |
| hsa-let-7d-3p | ERBB2 |
| hsa-let-7d-3p | RAB33B |
| hsa-let-7d-3p | SP3 |
| hsa-let-7d-3p | ZFP14 |
| hsa-let-7d-3p | ATG12 |
| hsa-let-7d-3p | PDE8B |
| hsa-let-7d-3p | CEP78 |
| hsa-let-7d-3p | ATG5 |
| hsa-let-7d-3p | ANXA7 |
| hsa-let-7d-3p | SCN9A |
| hsa-let-7d-3p | METTL10 |
| hsa-let-7d-3p | TMTC3 |
| hsa-let-7d-3p | AKIRIN1 |
| hsa-let-7d-3p | CCNT2 |
| hsa-let-7d-3p | CSGALNACT2 |
| hsa-let-7d-3p | BOD1 |
| hsa-let-7d-3p | TMEM106B |
| hsa-let-7d-3p | RRM1 |
| hsa-let-7d-3p | RFX2 |
| hsa-let-7d-3p | MAPK1 |
| hsa-let-7d-3p | PDCD10 |
| hsa-let-7d-3p | HNRNPA0 |
| hsa-let-7d-3p | SLC25A4 |
| hsa-let-7d-3p | TIMMDC1 |
| hsa-let-7d-3p | CDK17 |
| hsa-let-7d-3p | NUDCD3 |
| hsa-let-7d-3p | TFG |
| hsa-let-7d-3p | ATG7 |
| hsa-let-7d-3p | XPO4 |
| hsa-let-7d-3p | STX7 |
| hsa-let-7d-3p | TM9SF3 |
| hsa-let-7d-3p | EIF4E |
| hsa-let-7d-3p | DCUN1D5 |
| hsa-let-7d-3p | FAM98B |
| hsa-let-7d-3p | PDK1 |
| hsa-let-7d-3p | NBPF10 |
| hsa-let-7d-3p | HDAC2 |
| hsa-let-7d-3p | DNAJC10 |
| hsa-let-7d-3p | G3BP2 |
| hsa-let-7d-3p | NCKAP1 |
| hsa-let-7d-3p | TCEAL6 |
| hsa-let-7d-3p | CANX |
| hsa-let-7d-3p | ZFYVE16 |
| hsa-let-7d-3p | SIGLEC14 |
| hsa-let-7d-3p | MOBP |
| hsa-let-7d-3p | SPATS2 |
| hsa-let-7d-3p | C14orf23 |
| hsa-let-7d-3p | FBXO47 |
| hsa-let-7d-3p | TRAT1 |
| hsa-let-7d-3p | NKAPL |
| hsa-let-7d-3p | RP13-996F3.4 |
| hsa-let-7d-3p | PLG |
| hsa-let-7d-3p | HCN1 |
| hsa-let-7d-3p | JUND |
| hsa-let-7d-3p | SSTR2 |
| hsa-let-7d-3p | SEC24D |
| hsa-let-7d-3p | TCTEX1D1 |
| hsa-let-7d-3p | PCDHB15 |
| hsa-let-7d-3p | KCNJ3 |
| hsa-let-7d-3p | ZNF141 |
| hsa-let-7d-3p | SC5D |
| hsa-let-7d-3p | DUSP3 |
| hsa-let-7d-3p | FBXL5 |
| hsa-let-7d-3p | RWDD1 |
| hsa-let-7d-3p | FAM229B |
| hsa-let-7d-3p | ZNF827 |
| hsa-let-7d-3p | XRCC5 |
| hsa-let-7d-3p | MRPL39 |
| hsa-let-7d-3p | FAM208A |
| hsa-let-7d-3p | GHR |
| hsa-let-7d-3p | CRISPLD2 |
| hsa-let-7d-3p | TNNI1 |
| hsa-let-7d-3p | GPC6 |
| hsa-let-7d-3p | SOCS5 |
| hsa-let-7d-3p | LILRB2 |
| hsa-let-7d-3p | CD59 |
| hsa-let-7d-3p | EXOSC10 |
| hsa-miR-328-3p | DRGX |
| hsa-miR-328-3p | EIF4EBP1 |
| hsa-miR-328-3p | PGM2 |
| hsa-miR-328-3p | FOXS1 |
| hsa-miR-328-3p | CTNNBIP1 |
| hsa-miR-328-3p | CHP2 |
| hsa-miR-328-3p | DUSP16 |
| hsa-miR-328-3p | CHST7 |
| hsa-miR-328-3p | KLHL42 |
| hsa-miR-328-3p | EDARADD |
| hsa-miR-328-3p | LYVE1 |
| hsa-miR-328-3p | FUBP1 |
| hsa-miR-328-3p | UBFD1 |
| hsa-miR-328-3p | NF2 |
| hsa-miR-328-3p | SNX21 |
| hsa-miR-328-3p | NXPH3 |
| hsa-miR-328-3p | DYNC1I1 |
| hsa-miR-328-3p | TMEM229A |
| hsa-miR-328-3p | TCF7L2 |
| hsa-miR-328-3p | TESK2 |
| hsa-miR-328-3p | NR3C1 |
| hsa-miR-328-3p | CHTF8 |
| hsa-miR-328-3p | RGL2 |
| hsa-miR-328-3p | PIGA |
| hsa-miR-328-3p | NPFFR1 |
| hsa-miR-328-3p | NOTCH2NL |
| hsa-miR-328-3p | RANBP10 |
| hsa-miR-328-3p | SLC2A1 |
| hsa-miR-328-3p | FAM214B |
| hsa-miR-328-3p | RWDD2B |
| hsa-miR-328-3p | FAXDC2 |
| hsa-miR-328-3p | SRSF9 |
| hsa-miR-328-3p | ULK2 |
| hsa-miR-328-3p | ITGA5 |
| hsa-miR-328-3p | C16orf87 |
| hsa-miR-328-3p | EN2 |
| hsa-miR-328-3p | PARP16 |
| hsa-miR-328-3p | MAN1C1 |
| hsa-miR-328-3p | MMP16 |
| hsa-miR-328-3p | ADNP |
| hsa-miR-328-3p | TRAPPC6B |
| hsa-miR-328-3p | DDI2 |
| hsa-miR-328-3p | GBA |
| hsa-miR-328-3p | DERL2 |
| hsa-miR-328-3p | HPCAL4 |
| hsa-miR-328-3p | SHISA5 |
| hsa-miR-328-3p | PODNL1 |
| hsa-miR-328-3p | PIM1 |
| hsa-miR-328-3p | HIST1H4D |
| hsa-miR-328-3p | SLC30A8 |
| hsa-miR-328-3p | USP37 |
| hsa-miR-328-3p | KAT6B |
| hsa-miR-328-3p | LNX2 |
| hsa-miR-328-3p | MXI1 |
| hsa-miR-328-3p | HIST1H4L |
| hsa-miR-328-3p | USP8 |
| hsa-miR-328-3p | TP73 |
| hsa-miR-328-3p | GBX2 |
| hsa-miR-328-3p | MAP3K9 |
| hsa-miR-328-3p | CA3 |
| hsa-miR-328-3p | FOXO4 |
| hsa-miR-328-3p | C19orf43 |
| hsa-miR-328-3p | LGR4 |
| hsa-miR-328-3p | POLR3H |
| hsa-miR-328-3p | SNRK |
| hsa-miR-328-3p | HIC1 |
| hsa-miR-328-3p | ZNF697 |
| hsa-miR-328-3p | ESCO1 |
| hsa-miR-328-3p | TTC26 |
| hsa-miR-328-3p | C10orf113 |
| hsa-miR-328-3p | ZNF436 |
| hsa-miR-328-3p | STT3A |
| hsa-miR-328-3p | ZNF280B |
| hsa-miR-328-3p | ARL6IP1 |
| hsa-miR-328-3p | WASF2 |
| hsa-miR-328-3p | PTPN9 |
| hsa-miR-328-3p | NKPD1 |
| hsa-miR-328-3p | DNAH10OS |
| hsa-miR-328-3p | SOGA1 |
| hsa-miR-328-3p | PEF1 |
| hsa-miR-328-3p | RSBN1L |
| hsa-miR-328-3p | MPV17 |
| hsa-miR-328-3p | MBNL3 |
| hsa-miR-328-3p | LHFPL2 |
| hsa-miR-328-3p | SLC27A4 |
| hsa-miR-328-3p | NRIP1 |
| hsa-miR-328-3p | KIF2A |
| hsa-miR-328-3p | ITGA9 |
| hsa-miR-328-3p | ZC3H12B |
| hsa-miR-328-3p | HIF1AN |
| hsa-miR-328-3p | METTL21B |
| hsa-miR-328-3p | ACSS2 |
| hsa-miR-328-3p | YWHAZ |
| hsa-miR-328-3p | PTPRU |
| hsa-miR-328-3p | H2AFX |
| hsa-miR-328-3p | HAP1 |
| hsa-miR-328-3p | MICALL1 |
| hsa-miR-328-3p | C6orf223 |
| hsa-miR-328-3p | SHKBP1 |
| hsa-miR-328-3p | FAM199X |
| hsa-miR-328-3p | SCN2B |
| hsa-miR-328-3p | ANKRD40 |
| hsa-miR-328-3p | FAM211A |
| hsa-miR-328-3p | KIAA0247 |
| hsa-miR-328-3p | RNF152 |
| hsa-miR-328-3p | SLITRK3 |
| hsa-miR-328-3p | SLC7A6 |
| hsa-miR-328-3p | ERMP1 |
| hsa-miR-328-3p | RPS9 |
| hsa-miR-328-3p | KIAA1614 |
| hsa-miR-328-3p | CCDC88A |
| hsa-miR-328-3p | ZNF423 |
| hsa-miR-328-3p | TRIM71 |
| hsa-miR-328-3p | RPP14 |
| hsa-miR-328-3p | RS1 |
| hsa-miR-328-3p | EPHA10 |
| hsa-miR-328-3p | ESRP1 |
| hsa-miR-328-3p | NRG2 |
| hsa-miR-328-3p | EBF4 |
| hsa-miR-328-3p | SPECC1L |
| hsa-miR-328-3p | MPI |
| hsa-miR-328-3p | PRDM16 |
| hsa-miR-328-3p | XKR7 |
| hsa-miR-328-3p | SORL1 |
| hsa-miR-328-3p | SHISA6 |
| hsa-miR-328-3p | TMEM64 |
| hsa-miR-328-3p | RAD23B |
| hsa-miR-328-3p | WDTC1 |
| hsa-miR-328-3p | C20orf112 |
| hsa-miR-328-3p | CPT1A |
| hsa-miR-328-3p | C1orf226 |
| hsa-miR-328-3p | ZNF618 |
| hsa-miR-328-3p | KIAA1549 |
| hsa-miR-328-3p | MN1 |
| hsa-miR-328-3p | PTPRF |
| hsa-miR-328-3p | SF1 |
| hsa-miR-328-3p | RBM8A |
| hsa-miR-328-3p | PADI2 |
| hsa-miR-328-3p | RP11-366L20.2 |
| hsa-miR-328-3p | GSG1L |
| hsa-miR-328-3p | MFSD2A |
| hsa-miR-328-3p | ANKFY1 |
| hsa-miR-328-3p | SMC1A |
| hsa-miR-328-3p | HM13 |
| hsa-miR-328-3p | CNIH4 |
| hsa-miR-328-3p | ARPP19 |
| hsa-miR-328-3p | TSC1 |
| hsa-miR-328-3p | ATPAF1 |
| hsa-miR-328-3p | MIB1 |
| hsa-miR-328-3p | GRIN2B |
| hsa-miR-328-3p | PLEKHA6 |
| hsa-miR-328-3p | PTEN |
| hsa-miR-328-3p | RPS3 |
| hsa-miR-328-3p | TOX4 |
| hsa-miR-328-3p | C15orf37 |
| hsa-miR-328-3p | DYNLL2 |
| hsa-miR-328-3p | TMEM248 |
| hsa-miR-328-3p | NHSL2 |
| hsa-miR-328-3p | LEPROTL1 |
| hsa-miR-328-3p | ARHGEF17 |
| hsa-miR-328-3p | RGP1 |
| hsa-miR-328-3p | LSM14B |
| hsa-miR-328-3p | GOSR1 |
| hsa-miR-328-3p | SOX11 |
| hsa-miR-328-3p | SLC8A1 |
| hsa-miR-328-3p | PHF15 |
| hsa-miR-328-3p | CREB1 |
| hsa-miR-328-3p | AGO1 |
| hsa-miR-328-3p | ETV6 |
| hsa-miR-328-3p | PPP2R5D |
| hsa-miR-328-3p | RAP1GAP2 |
| hsa-miR-328-3p | ASIC1 |
| hsa-miR-328-3p | CNOT2 |
| hsa-miR-328-3p | EPB41L1 |
| hsa-miR-328-3p | CELSR2 |
| hsa-miR-328-3p | PPIP5K1 |
| hsa-miR-328-3p | LMOD1 |
| hsa-miR-328-3p | GTDC1 |
| hsa-miR-328-3p | LPP |
| hsa-miR-328-3p | AP5M1 |
| hsa-miR-328-3p | MECP2 |
| hsa-miR-328-3p | CTIF |
| hsa-miR-328-3p | ESRRA |
| hsa-miR-328-3p | DNAJB14 |
| hsa-miR-328-3p | FGD1 |
| hsa-miR-328-3p | FMNL3 |
| hsa-miR-328-3p | SDK2 |
| hsa-miR-328-3p | TMEM132B |
| hsa-miR-328-3p | LIMD1 |
| hsa-miR-328-3p | RAB3C |
| hsa-miR-328-3p | CBX8 |
| hsa-miR-328-3p | PLA2G12A |
| hsa-miR-328-3p | CTNS |
| hsa-miR-328-3p | GRAMD1B |
| hsa-miR-328-3p | DNAJC18 |
| hsa-miR-328-3p | NUFIP2 |
| hsa-miR-328-3p | RBP2 |
| hsa-miR-328-3p | TGFB2 |
| hsa-miR-328-3p | PLEKHG3 |
| hsa-miR-328-3p | SIX1 |
| hsa-miR-328-3p | NIT1 |
| hsa-miR-328-3p | CACNG2 |
| hsa-miR-328-3p | HEYL |
| hsa-miR-328-3p | FKBP15 |
| hsa-miR-186-5p | FZD3 |
| hsa-miR-186-5p | AC114546.1 |
| hsa-miR-186-5p | CXCL13 |
| hsa-miR-186-5p | GRIN2B |
| hsa-miR-186-5p | EIF2D |
| hsa-miR-186-5p | MAP3K2 |
| hsa-miR-186-5p | MAPKAPK5 |
| hsa-miR-186-5p | KB-1980E6.3 |
| hsa-miR-186-5p | SMIM15 |
| hsa-miR-186-5p | DNAJC8 |
| hsa-miR-186-5p | ARAP2 |
| hsa-miR-186-5p | AC079341.1 |
| hsa-miR-186-5p | RPS6KB1 |
| hsa-miR-186-5p | ARCN1 |
| hsa-miR-186-5p | HOXB2 |
| hsa-miR-186-5p | DUS3L |
| hsa-miR-186-5p | SYCP1 |
| hsa-miR-186-5p | FUT8 |
| hsa-miR-186-5p | NIT2 |
| hsa-miR-186-5p | ZMYM6 |
| hsa-miR-186-5p | PID1 |
| hsa-miR-186-5p | HNMT |
| hsa-miR-186-5p | FOXN2 |
| hsa-miR-186-5p | IRF8 |
| hsa-miR-186-5p | GABRA4 |
| hsa-miR-186-5p | INSM1 |
| hsa-miR-186-5p | BMPR1A |
| hsa-miR-186-5p | UHMK1 |
| hsa-miR-186-5p | DSPP |
| hsa-miR-186-5p | DUT |
| hsa-miR-186-5p | PRKAA2 |
| hsa-miR-186-5p | UBE2K |
| hsa-miR-186-5p | RHBDF2 |
| hsa-miR-186-5p | ACSM2A |
| hsa-miR-186-5p | ACSM2B |
| hsa-miR-186-5p | CLPX |
| hsa-miR-186-5p | UBE2R2 |
| hsa-miR-186-5p | LIN7C |
| hsa-miR-186-5p | TOB1 |
| hsa-miR-186-5p | BDH2 |
| hsa-miR-186-5p | SUMO2 |
| hsa-miR-186-5p | MXD1 |
| hsa-miR-186-5p | MOB1A |
| hsa-miR-186-5p | BCAT1 |
| hsa-miR-186-5p | GPR37 |
| hsa-miR-186-5p | ZDHHC13 |
| hsa-miR-186-5p | UBE2B |
| hsa-miR-186-5p | SCN2A |
| hsa-miR-186-5p | FAM160B1 |
| hsa-miR-186-5p | STK17B |
| hsa-miR-186-5p | THEMIS |
| hsa-miR-186-5p | SUCLA2 |
| hsa-miR-186-5p | PSPH |
| hsa-miR-186-5p | ANK3 |
| hsa-miR-186-5p | CLLU1 |
| hsa-miR-186-5p | PTH |
| hsa-miR-186-5p | TPR |
| hsa-miR-186-5p | DSTN |
| hsa-miR-186-5p | CA1 |
| hsa-miR-186-5p | TMEM251 |
| hsa-miR-186-5p | ZNF644 |
| hsa-miR-186-5p | CHST2 |
| hsa-miR-186-5p | SAMD12 |
| hsa-miR-186-5p | TRAPPC8 |
| hsa-miR-186-5p | PIGF |
| hsa-miR-186-5p | FAM193B |
| hsa-miR-186-5p | BCAR3 |
| hsa-miR-186-5p | MIB1 |
| hsa-miR-186-5p | CALB1 |
| hsa-miR-186-5p | TMED2 |
| hsa-miR-186-5p | NOG |
| hsa-miR-186-5p | YY1 |
| hsa-miR-186-5p | RAB2A |
| hsa-miR-186-5p | RAB11B |
| hsa-miR-186-5p | EED |
| hsa-miR-186-5p | BET1 |
| hsa-miR-186-5p | ANO2 |
| hsa-miR-186-5p | C7orf76 |
| hsa-miR-186-5p | CDK5R1 |
| hsa-miR-186-5p | KHDRBS3 |
| hsa-miR-186-5p | MRPS10 |
| hsa-miR-186-5p | AGO2 |
| hsa-miR-186-5p | PGM3 |
| hsa-miR-186-5p | CDC73 |
| hsa-miR-186-5p | NCBP2 |
| hsa-miR-186-5p | LMO3 |
| hsa-miR-186-5p | BMP5 |
| hsa-miR-186-5p | RORB |
| hsa-miR-186-5p | SNRNP48 |
| hsa-miR-186-5p | SALL1 |
| hsa-miR-186-5p | C1QTNF3 |
| hsa-miR-186-5p | TEAD1 |
| hsa-miR-186-5p | RGS22 |
| hsa-miR-186-5p | ARHGAP5 |
| hsa-miR-186-5p | UBR2 |
| hsa-miR-186-5p | C11orf65 |
| hsa-miR-186-5p | PPM1B |
| hsa-miR-186-5p | RB1CC1 |
| hsa-miR-186-5p | DYNLRB1 |
| hsa-miR-186-5p | CISD1 |
| hsa-miR-186-5p | ERI1 |
| hsa-miR-186-5p | PTS |
| hsa-miR-186-5p | TMF1 |
| hsa-miR-186-5p | RNF125 |
| hsa-miR-186-5p | GYPA |
| hsa-miR-186-5p | GPR183 |
| hsa-miR-186-5p | APTX |
| hsa-miR-186-5p | ELOVL6 |
| hsa-miR-186-5p | PABPC1 |
| hsa-miR-186-5p | CDK6 |
| hsa-miR-186-5p | NBEAL1 |
| hsa-miR-186-5p | PDE10A |
| hsa-miR-186-5p | KDELR2 |
| hsa-miR-186-5p | EPC2 |
| hsa-miR-186-5p | GJA3 |
| hsa-miR-186-5p | TERF1 |
| hsa-miR-186-5p | NDFIP2 |
| hsa-miR-186-5p | RSU1 |
| hsa-miR-186-5p | XIAP |
| hsa-miR-186-5p | VEZT |
| hsa-miR-186-5p | NRL |
| hsa-miR-186-5p | SPRYD7 |
| hsa-miR-186-5p | DUSP13 |
| hsa-miR-186-5p | ANO6 |
| hsa-miR-186-5p | NXPH1 |
| hsa-miR-186-5p | NKX2-1 |
| hsa-miR-186-5p | SRSF9 |
| hsa-miR-186-5p | RIOK3 |
| hsa-miR-186-5p | ADRA1A |
| hsa-miR-186-5p | SLC16A9 |
| hsa-miR-186-5p | CABS1 |
| hsa-miR-186-5p | ALG14 |
| hsa-miR-186-5p | CAMLG |
| hsa-miR-186-5p | GPATCH2L |
| hsa-miR-186-5p | CADM2 |
| hsa-miR-186-5p | PXDC1 |
| hsa-miR-186-5p | ELK4 |
| hsa-miR-186-5p | INTS2 |
| hsa-miR-186-5p | GIPC2 |
| hsa-miR-186-5p | GK5 |
| hsa-miR-186-5p | SNAPC3 |
| hsa-miR-186-5p | TMED7-TICAM2 |
| hsa-miR-186-5p | AK3 |
| hsa-miR-186-5p | KLHL23 |
| hsa-miR-186-5p | GTF2A2 |
| hsa-miR-186-5p | ONECUT2 |
| hsa-miR-186-5p | AKAP5 |
| hsa-miR-186-5p | GINM1 |
| hsa-miR-186-5p | FAR1 |
| hsa-miR-186-5p | PPP1CC |
| hsa-miR-186-5p | SMIM8 |
| hsa-miR-186-5p | MAML2 |
| hsa-miR-186-5p | SLC17A8 |
| hsa-miR-186-5p | TICAM2 |
| hsa-miR-186-5p | DENND5B |
| hsa-miR-186-5p | SP4 |
| hsa-miR-186-5p | HMGN5 |
| hsa-miR-186-5p | ZNF710 |
| hsa-miR-186-5p | ZNF608 |
| hsa-miR-186-5p | TCF21 |
| hsa-miR-186-5p | DUSP7 |
| hsa-miR-186-5p | ZCCHC11 |
| hsa-miR-186-5p | ZFP36L2 |
| hsa-miR-186-5p | UBR3 |
| hsa-miR-186-5p | SYAP1 |
| hsa-miR-186-5p | HCN1 |
| hsa-miR-186-5p | SORT1 |
| hsa-miR-186-5p | EFEMP1 |
| hsa-miR-186-5p | AGO3 |
| hsa-miR-186-5p | CHIC1 |
| hsa-miR-186-5p | PDCD10 |
| hsa-miR-186-5p | DNAJC27 |
| hsa-miR-186-5p | TRPC1 |
| hsa-miR-186-5p | XPOT |
| hsa-miR-186-5p | KLF12 |
| hsa-miR-186-5p | CLK4 |
| hsa-miR-186-5p | SGPP2 |
| hsa-miR-186-5p | MOSPD1 |
| hsa-miR-186-5p | KCNK13 |
| hsa-miR-186-5p | BMP2K |
| hsa-miR-186-5p | HELZ |
| hsa-miR-186-5p | PDGFC |
| hsa-miR-186-5p | TMEM170B |
| hsa-miR-186-5p | CYCS |
| hsa-miR-186-5p | SOGA3 |
| hsa-miR-186-5p | DNAJB4 |
| hsa-miR-186-5p | INO80D |
| hsa-miR-186-5p | TMOD1 |
| hsa-miR-186-5p | FAM114A2 |
| hsa-miR-186-5p | RP11-625H11.1 |
| hsa-miR-186-5p | RNF181 |
| hsa-miR-186-5p | B3GAT2 |
| hsa-miR-186-5p | NUP35 |
| hsa-miR-186-5p | FUT9 |
| hsa-miR-186-5p | CREBRF |
| hsa-miR-186-5p | GPCPD1 |
| hsa-miR-186-5p | KLHL31 |
| hsa-miR-186-5p | CISD2 |
| hsa-miR-186-5p | HOOK3 |
| hsa-miR-186-5p | MAP2 |
| hsa-miR-186-5p | CNOT7 |
| hsa-miR-186-5p | ATP6V1C2 |
| hsa-miR-186-5p | GOSR1 |
| hsa-miR-186-5p | AGFG1 |
| hsa-miR-186-5p | PHIP |
| hsa-miR-186-5p | ST13 |
| hsa-miR-186-5p | BTF3 |
| hsa-miR-186-5p | MITF |
| hsa-miR-186-5p | FAM105A |
| hsa-miR-186-5p | RAB21 |
| hsa-miR-186-5p | ZFP36L1 |
| hsa-miR-186-5p | LIN7A |
| hsa-miR-186-5p | PARG |
| hsa-miR-186-5p | SRI |
| hsa-miR-186-5p | RGPD6 |
| hsa-miR-186-5p | RAB6A |
| hsa-miR-186-5p | PDK3 |
| hsa-miR-186-5p | ZNF652 |
| hsa-miR-186-5p | NECAB1 |
| hsa-miR-186-5p | S100PBP |
| hsa-miR-186-5p | CASP9 |
| hsa-miR-186-5p | RUNX1T1 |
| hsa-miR-186-5p | GPR128 |
| hsa-miR-186-5p | ATAD2 |
| hsa-miR-186-5p | WNT4 |
| hsa-miR-186-5p | TMEM168 |
| hsa-miR-186-5p | ARIH1 |
| hsa-miR-186-5p | OTUD4 |
| hsa-miR-186-5p | KLF7 |
| hsa-miR-186-5p | KIAA0087 |
| hsa-miR-186-5p | WNK3 |
| hsa-miR-186-5p | PEG3 |
| hsa-miR-186-5p | RIMS2 |
| hsa-miR-186-5p | AC140061.12 |
| hsa-miR-186-5p | ZC3H6 |
| hsa-miR-186-5p | MACC1 |
| hsa-miR-186-5p | SNX30 |
| hsa-miR-186-5p | FAM124B |
| hsa-miR-186-5p | VGLL3 |
| hsa-miR-186-5p | NADK2 |
| hsa-miR-186-5p | BTBD3 |
| hsa-miR-186-5p | ZBTB21 |
| hsa-miR-186-5p | NUP155 |
| hsa-miR-186-5p | FHL5 |
| hsa-miR-186-5p | TMEM183A |
| hsa-miR-186-5p | DGKH |
| hsa-miR-186-5p | USP37 |
| hsa-miR-186-5p | RBFOX2 |
| hsa-miR-186-5p | C5orf51 |
| hsa-miR-186-5p | TMTC1 |
| hsa-miR-186-5p | CLVS2 |
| hsa-miR-186-5p | MAP7 |
| hsa-miR-186-5p | MAN1A2 |
| hsa-miR-186-5p | FKBP14 |
| hsa-miR-186-5p | MORC1 |
| hsa-miR-186-5p | IRX2 |
| hsa-miR-186-5p | TENM1 |
| hsa-miR-186-5p | LHFPL2 |
| hsa-miR-186-5p | C4orf32 |
| hsa-miR-186-5p | ZNF706 |
| hsa-miR-186-5p | LPGAT1 |
| hsa-miR-186-5p | LAMP2 |
| hsa-miR-186-5p | EPN2 |
| hsa-miR-186-5p | IMPG2 |
| hsa-miR-186-5p | SLC30A8 |
| hsa-miR-186-5p | DARS |
| hsa-miR-186-5p | SHANK2 |
| hsa-miR-186-5p | REL |
| hsa-miR-186-5p | QKI |
| hsa-miR-186-5p | PAPD5 |
| hsa-miR-186-5p | AKIRIN1 |
| hsa-miR-186-5p | RAB41 |
| hsa-miR-186-5p | AFTPH |
| hsa-miR-186-5p | JAG1 |
| hsa-miR-186-5p | RP11-422N16.3 |
| hsa-miR-186-5p | ASB7 |
| hsa-miR-186-5p | KLHL11 |
| hsa-miR-186-5p | JHDM1D |
| hsa-miR-186-5p | CENPK |
| hsa-miR-186-5p | RBFOX1 |
| hsa-miR-186-5p | ACTR3 |
| hsa-miR-186-5p | RP1-170O19.20 |
| hsa-miR-186-5p | C18orf25 |
| hsa-miR-186-5p | NEGR1 |
| hsa-miR-186-5p | CNTNAP2 |
| hsa-miR-186-5p | IDS |
| hsa-miR-186-5p | SNX27 |
| hsa-miR-186-5p | MBLAC2 |
| hsa-miR-186-5p | ARPC2 |
| hsa-miR-186-5p | FOXK1 |
| hsa-miR-186-5p | TMEM35 |
| hsa-miR-186-5p | SCD |
| hsa-miR-186-5p | EAF1 |
| hsa-miR-186-5p | BTD |
| hsa-miR-186-5p | KIAA0408 |
| hsa-miR-186-5p | SPAST |
| hsa-miR-186-5p | FGF7 |
| hsa-miR-186-5p | C6orf62 |
| hsa-miR-186-5p | RORC |
| hsa-miR-186-5p | DYNLL2 |
| hsa-miR-186-5p | SEC62 |
| hsa-miR-186-5p | PDE4D |
| hsa-miR-186-5p | CHURC1 |
| hsa-miR-186-5p | ERI2 |
| hsa-miR-186-5p | DEK |
| hsa-miR-186-5p | GMFB |
| hsa-miR-186-5p | BRWD3 |
| hsa-miR-186-5p | FAM126B |
| hsa-miR-186-5p | SKP1 |
| hsa-miR-186-5p | FIGN |
| hsa-miR-186-5p | CEP57 |
| hsa-miR-186-5p | RPS6KA3 |
| hsa-miR-186-5p | PAK3 |
| hsa-miR-186-5p | MCFD2 |
| hsa-miR-186-5p | GRIA2 |
| hsa-miR-186-5p | CGNL1 |
| hsa-miR-186-5p | ZFAND3 |
| hsa-miR-186-5p | UNC45B |
| hsa-miR-186-5p | TMCO1 |
| hsa-miR-186-5p | PAPPA2 |
| hsa-miR-186-5p | OCLN |
| hsa-miR-186-5p | FAM117B |
| hsa-miR-186-5p | SHPRH |
| hsa-miR-186-5p | MCTP1 |
| hsa-miR-186-5p | GCC2 |
| hsa-miR-186-5p | RAD54L2 |
| hsa-miR-186-5p | TMED8 |
| hsa-miR-186-5p | SH3RF1 |
| hsa-miR-186-5p | SPTSSA |
| hsa-miR-186-5p | G2E3 |
| hsa-miR-186-5p | NHLRC2 |
| hsa-miR-186-5p | EIF3A |
| hsa-miR-186-5p | ZKSCAN3 |
| hsa-miR-186-5p | LMBR1 |
| hsa-miR-186-5p | WIPI2 |
| hsa-miR-186-5p | AZIN1 |
| hsa-miR-186-5p | C21orf91 |
| hsa-miR-186-5p | NUCKS1 |
| hsa-miR-186-5p | LDHAL6A |
| hsa-miR-186-5p | KRR1 |
| hsa-miR-186-5p | FGF5 |
| hsa-miR-186-5p | CPEB3 |
| hsa-miR-186-5p | CPD |
| hsa-miR-186-5p | USF2 |
| hsa-miR-186-5p | SLAMF6 |
| hsa-miR-186-5p | KIAA1462 |
| hsa-miR-186-5p | PPTC7 |
| hsa-miR-186-5p | MAPK6 |
| hsa-miR-186-5p | PTAR1 |
| hsa-miR-186-5p | PTEN |
| hsa-miR-186-5p | CDC5L |
| hsa-miR-186-5p | RAD23B |
| hsa-miR-186-5p | PRDX6 |
| hsa-miR-186-5p | LCOR |
| hsa-miR-186-5p | ARMC10 |
| hsa-miR-186-5p | PPP1R12B |
| hsa-miR-186-5p | C15orf56 |
| hsa-miR-186-5p | NCKAP5 |
| hsa-miR-186-5p | PPIP5K2 |
| hsa-miR-186-5p | GCNT4 |
| hsa-miR-186-5p | DKK1 |
| hsa-miR-186-5p | TTC13 |
| hsa-miR-186-5p | RNF217 |
| hsa-miR-186-5p | CDC42 |
| hsa-miR-186-5p | PRKCA |
| hsa-miR-186-5p | PLEK |
| hsa-miR-186-5p | SMARCA5 |
| hsa-miR-186-5p | MPLKIP |
| hsa-miR-186-5p | ELF1 |
| hsa-miR-186-5p | CAST |
| hsa-miR-186-5p | SGMS2 |
| hsa-miR-186-5p | SHOC2 |
| hsa-miR-186-5p | ZDHHC23 |
| hsa-miR-186-5p | KIAA1324L |
| hsa-miR-186-5p | PURB |
| hsa-miR-186-5p | FOXD1 |
| hsa-miR-186-5p | RPS15A |
| hsa-miR-186-5p | NDN |
| hsa-miR-186-5p | MAFG |
| hsa-miR-186-5p | CD55 |
| hsa-miR-186-5p | ZFYVE16 |
| hsa-miR-186-5p | RAD18 |
| hsa-miR-186-5p | SMC3 |
| hsa-miR-186-5p | LMAN1 |
| hsa-miR-186-5p | ITGA6 |
| hsa-miR-186-5p | COL6A6 |
| hsa-miR-186-5p | ZBTB39 |
| hsa-miR-186-5p | PSPC1 |
| hsa-miR-186-5p | PAK7 |
| hsa-miR-186-5p | TMEM236 |
| hsa-miR-186-5p | PTPN11 |
| hsa-miR-186-5p | TDRD15 |
| hsa-miR-186-5p | TMEM236 |
| hsa-miR-186-5p | TMEM132B |
| hsa-miR-186-5p | PAX5 |
| hsa-miR-186-5p | PDE3B |
| hsa-miR-186-5p | OXSR1 |
| hsa-miR-186-5p | SYT11 |
| hsa-miR-186-5p | YIPF6 |
| hsa-miR-186-5p | SCOC |
| hsa-miR-186-5p | C16orf52 |
| hsa-miR-186-5p | PP13439 |
| hsa-miR-186-5p | ZBTB8B |
| hsa-miR-186-5p | IGF1 |
| hsa-miR-186-5p | SYTL3 |
| hsa-miR-186-5p | MAPK1 |
| hsa-miR-186-5p | IL2 |
| hsa-miR-186-5p | EIF2S2 |
| hsa-miR-186-5p | WDR44 |
| hsa-miR-186-5p | KBTBD7 |
| hsa-miR-186-5p | PARVA |
| hsa-miR-186-5p | ACAP2 |
| hsa-miR-186-5p | EIF4E3 |
| hsa-miR-186-5p | C10orf118 |
| hsa-miR-186-5p | PDE3A |
| hsa-miR-186-5p | HERPUD2 |
| hsa-miR-186-5p | CREB3L2 |
| hsa-miR-186-5p | TBC1D12 |
| hsa-miR-186-5p | FAM84A |
| hsa-miR-186-5p | IGSF11 |
| hsa-miR-186-5p | SRGAP2 |
| hsa-miR-186-5p | SULT1C2 |
| hsa-miR-186-5p | KCTD16 |
| hsa-miR-186-5p | CREG2 |
| hsa-miR-186-5p | UBQLN2 |
| hsa-miR-186-5p | LNPEP |
| hsa-miR-186-5p | PARD3B |
| hsa-miR-186-5p | ACVR2B |
| hsa-miR-186-5p | CYB5R4 |
| hsa-miR-186-5p | ENPEP |
| hsa-miR-186-5p | PCSK1 |
| hsa-miR-186-5p | SPSB1 |
| hsa-miR-186-5p | CCNT2 |
| hsa-miR-186-5p | SERAC1 |
| hsa-miR-186-5p | PMEPA1 |
| hsa-miR-186-5p | BICD2 |
| hsa-miR-186-5p | ODF2L |
| hsa-miR-186-5p | TSC22D2 |
| hsa-miR-186-5p | BPTF |
| hsa-miR-186-5p | GPR85 |
| hsa-miR-186-5p | LPPR4 |
| hsa-miR-186-5p | ATXN7 |
| hsa-miR-186-5p | ASXL3 |
| hsa-miR-186-5p | ZNF592 |
| hsa-miR-186-5p | CNTN5 |
| hsa-miR-186-5p | PCDH17 |
| hsa-miR-186-5p | CCDC144A |
| hsa-miR-186-5p | FAM13A |
| hsa-miR-186-5p | SYT10 |
| hsa-miR-186-5p | SNAP25 |
| hsa-miR-186-5p | MAP4K3 |
| hsa-miR-186-5p | KCNJ15 |
| hsa-miR-186-5p | OSER1 |
| hsa-miR-186-5p | SIPA1L3 |
| hsa-miR-186-5p | ATP2B4 |
| hsa-miR-186-5p | SYT6 |
| hsa-miR-186-5p | IGSF3 |
| hsa-miR-186-5p | STRN |
| hsa-miR-186-5p | MFAP3L |
| hsa-miR-186-5p | ZBTB8A |
| hsa-miR-186-5p | PHF21A |
| hsa-miR-186-5p | BRINP2 |
| hsa-miR-186-5p | RP11-10A14.4 |
| hsa-miR-186-5p | OSBPL3 |
| hsa-miR-186-5p | PSMD11 |
| hsa-miR-186-5p | DCP1A |
| hsa-miR-186-5p | FAM83B |
| hsa-miR-186-5p | TULP4 |
| hsa-miR-186-5p | AFF1 |
| hsa-miR-186-5p | SRPK2 |
| hsa-miR-186-5p | RRAGD |
| hsa-miR-186-5p | TBCEL |
| hsa-miR-186-5p | FGF2 |
| hsa-miR-186-5p | BCL2L11 |
| hsa-miR-186-5p | SEPT7 |
| hsa-miR-186-5p | NFAT5 |
| hsa-miR-186-5p | MEX3B |
| hsa-miR-186-5p | ZCCHC24 |
| hsa-miR-186-5p | PIK3R3 |
| hsa-miR-186-5p | DSN1 |
| hsa-miR-186-5p | SERINC3 |
| hsa-miR-186-5p | LAMP1 |
| hsa-miR-186-5p | CDC42BPA |
| hsa-miR-186-5p | CLSTN2 |
| hsa-miR-186-5p | IPO5 |
| hsa-miR-186-5p | UBXN7 |
| hsa-miR-186-5p | PBX1 |
| hsa-miR-186-5p | NSD1 |
| hsa-miR-186-5p | LRP12 |
| hsa-miR-186-5p | CNTNAP1 |
| hsa-miR-186-5p | CSNK2A1 |
| hsa-miR-186-5p | NPR3 |
| hsa-miR-186-5p | RPA3 |
| hsa-miR-186-5p | RBM7 |
| hsa-miR-186-5p | CCDC80 |
| hsa-miR-186-5p | COBLL1 |
| hsa-miR-186-5p | MSRB3 |
| hsa-miR-186-5p | SETD9 |
| hsa-miR-186-5p | NIPSNAP3B |
| hsa-miR-186-5p | ARHGAP22 |
| hsa-miR-186-5p | PIAS2 |
| hsa-miR-186-5p | ATL1 |
| hsa-miR-186-5p | HAS2 |
| hsa-miR-186-5p | KIAA1468 |
| hsa-miR-186-5p | FXR1 |
| hsa-miR-186-5p | KLHL24 |
| hsa-miR-186-5p | CEP41 |
| hsa-miR-186-5p | DHODH |
| hsa-miR-186-5p | RBM38 |
| hsa-miR-186-5p | CCDC171 |
| hsa-miR-186-5p | GCLM |
| hsa-miR-186-5p | FICD |
| hsa-miR-186-5p | FTO |
| hsa-miR-186-5p | SREK1 |
| hsa-miR-186-5p | FAM120A |
| hsa-miR-186-5p | RDH10 |
| hsa-miR-186-5p | TERF2 |
| hsa-miR-186-5p | CDKN1B |
| hsa-miR-186-5p | SESTD1 |
| hsa-miR-186-5p | NKAP |
| hsa-miR-186-5p | TSPYL1 |
| hsa-miR-186-5p | TMED7 |
| hsa-miR-186-5p | JMY |
| hsa-miR-186-5p | KNDC1 |
| hsa-miR-186-5p | WNT7A |
| hsa-miR-186-5p | ICK |
| hsa-miR-186-5p | SMPD3 |
| hsa-miR-186-5p | CRAMP1L |
| hsa-miR-186-5p | MTF1 |
| hsa-miR-186-5p | PEG10 |
| hsa-miR-186-5p | RAB3IP |
| hsa-miR-186-5p | MTMR6 |
| hsa-miR-186-5p | BCL11B |
| hsa-miR-186-5p | ZNF81 |
| hsa-miR-186-5p | SETD8 |
| hsa-miR-186-5p | PDS5B |
| hsa-miR-186-5p | NUS1 |
| hsa-miR-186-5p | ACSBG1 |
| hsa-miR-186-5p | ZNF831 |
| hsa-miR-186-5p | ZBTB7A |
| hsa-miR-186-5p | FAM161A |
| hsa-miR-186-5p | ZNF449 |
| hsa-miR-186-5p | ROCK1 |
| hsa-miR-186-5p | WWC3 |
| hsa-miR-186-5p | GRIN3A |
| hsa-miR-186-5p | JPH3 |
| hsa-miR-186-5p | SLC39A14 |
| hsa-miR-186-5p | DLC1 |
| hsa-miR-186-5p | ZDBF2 |
| hsa-miR-186-5p | CDKN2AIP |
| hsa-miR-186-5p | MLTK |
| hsa-miR-186-5p | PAQR3 |
| hsa-miR-186-5p | APBB2 |
| hsa-miR-186-5p | C2orf69 |
| hsa-miR-186-5p | FRMD4A |
| hsa-miR-186-5p | ZBTB34 |
| hsa-miR-186-5p | MYPN |
| hsa-miR-186-5p | LHX4 |
| hsa-miR-186-5p | CA5B |
| hsa-miR-186-5p | NEDD4 |
| hsa-miR-186-5p | PLXDC2 |
| hsa-miR-186-5p | ABCC1 |
| hsa-miR-186-5p | PDCD4 |
| hsa-miR-186-5p | PRPF40A |
| hsa-miR-186-5p | ZZZ3 |
| hsa-miR-186-5p | SDC2 |
| hsa-miR-186-5p | TRIM67 |
| hsa-miR-186-5p | HUNK |
| hsa-miR-186-5p | SLC30A7 |
| hsa-miR-186-5p | ATP1A2 |
| hsa-miR-186-5p | ATP9A |
| hsa-miR-186-5p | HMGB1 |
| hsa-miR-186-5p | ZIC5 |
| hsa-miR-186-5p | FAM104A |
| hsa-miR-186-5p | TIPARP |
| hsa-miR-186-5p | WTIP |
| hsa-miR-186-5p | FAM126A |
| hsa-miR-186-5p | SLC26A4 |
| hsa-miR-186-5p | EIF1AX |
| hsa-miR-186-5p | STON2 |
| hsa-miR-186-5p | PTCHD1 |
| hsa-miR-186-5p | SATB1 |
| hsa-miR-186-5p | CCND2 |
| hsa-miR-186-5p | GLIPR1 |
| hsa-miR-186-5p | CHRM3 |
| hsa-miR-186-5p | NR5A2 |
| hsa-miR-186-5p | RSBN1L |
| hsa-miR-186-5p | VPS37A |
| hsa-miR-186-5p | CDK14 |
| hsa-miR-186-5p | JAG2 |
| hsa-miR-186-5p | KLB |
| hsa-miR-186-5p | SNX2 |
| hsa-miR-186-5p | C17orf85 |
| hsa-miR-186-5p | CHD9 |
| hsa-miR-186-5p | MLLT3 |
| hsa-miR-186-5p | FNDC3B |
| hsa-miR-186-5p | ANKRD12 |
| hsa-miR-186-5p | ABCC2 |
| hsa-miR-186-5p | SAMD8 |
| hsa-miR-186-5p | KPNA6 |
| hsa-miR-186-5p | TRHDE |
| hsa-miR-186-5p | BMPR2 |
| hsa-miR-186-5p | PSME3 |
| hsa-miR-186-5p | CTDSPL2 |
| hsa-miR-186-5p | MFSD6 |
| hsa-miR-186-5p | C7orf55-LUC7L2 |
| hsa-miR-186-5p | PTGES3 |
| hsa-miR-186-5p | CUL3 |
| hsa-miR-186-5p | PDE12 |
| hsa-miR-186-5p | UBE2Q2 |
| hsa-miR-186-5p | METTL21A |
| hsa-miR-186-5p | SCN9A |
| hsa-miR-186-5p | SEC63 |
| hsa-miR-186-5p | GTDC1 |
| hsa-miR-186-5p | MBD5 |
| hsa-miR-186-5p | KIAA0355 |
| hsa-miR-186-5p | SPATA5 |
| hsa-miR-186-5p | UHRF1BP1L |
| hsa-miR-186-5p | BZW1 |
| hsa-miR-186-5p | CREBBP |
| hsa-miR-186-5p | HPSE |
| hsa-miR-186-5p | C8orf46 |
| hsa-miR-186-5p | RNF216 |
| hsa-miR-186-5p | ERCC6L |
| hsa-miR-186-5p | SLC35A3 |
| hsa-miR-186-5p | TLN1 |
| hsa-miR-186-5p | LUC7L2 |
| hsa-miR-186-5p | BNC2 |
| hsa-miR-186-5p | ZCCHC4 |
| hsa-miR-186-5p | NUDCD2 |
| hsa-miR-186-5p | RFC1 |
| hsa-miR-186-5p | SOX11 |
| hsa-miR-186-5p | FAM204A |
| hsa-miR-186-5p | NSG2 |
| hsa-miR-186-5p | SEC61A1 |
| hsa-miR-186-5p | CNKSR3 |
| hsa-miR-186-5p | AGPAT5 |
| hsa-miR-186-5p | IGF1R |
| hsa-miR-186-5p | NAA15 |
| hsa-miR-186-5p | OSBPL8 |
| hsa-miR-186-5p | DCAF10 |
| hsa-miR-186-5p | TRPM7 |
| hsa-miR-186-5p | TMEM184A |
| hsa-miR-186-5p | ANKRD28 |
| hsa-miR-186-5p | SRSF10 |
| hsa-miR-186-5p | EIF4E |
| hsa-miR-186-5p | SP3 |
| hsa-miR-186-5p | SCAF8 |
| hsa-miR-186-5p | TGFBR2 |
| hsa-miR-186-5p | LRRTM3 |
| hsa-miR-186-5p | POFUT2 |
| hsa-miR-186-5p | BLMH |
| hsa-miR-186-5p | PHC1 |
| hsa-miR-186-5p | DPP9 |
| hsa-miR-186-5p | FRMD4B |
| hsa-miR-186-5p | PCDHA2 |
| hsa-miR-186-5p | PSMD5 |
| hsa-miR-186-5p | NCOA4 |
| hsa-miR-186-5p | EXD1 |
| hsa-miR-186-5p | KRT77 |
| hsa-miR-186-5p | PCDHA12 |
| hsa-miR-186-5p | EP300 |
| hsa-miR-186-5p | PCDHA6 |
| hsa-miR-186-5p | RBMX2 |
| hsa-miR-186-5p | RSPH10B |
| hsa-miR-186-5p | CCDC30 |
| hsa-miR-186-5p | MIER1 |
| hsa-miR-186-5p | MYT1L |
| hsa-miR-186-5p | PCDHA4 |
| hsa-miR-186-5p | SNX18 |
| hsa-miR-186-5p | MYT1 |
| hsa-miR-186-5p | RNF11 |
| hsa-miR-186-5p | PCDHA10 |
| hsa-miR-186-5p | GPR12 |
| hsa-miR-186-5p | DOCK3 |
| hsa-miR-186-5p | PCDHAC2 |
| hsa-miR-186-5p | PCDHA1 |
| hsa-miR-186-5p | MORC2 |
| hsa-miR-186-5p | SLC5A12 |
| hsa-miR-186-5p | PLEKHA3 |
| hsa-miR-186-5p | PCDHA7 |
| hsa-miR-186-5p | TMEM151B |
| hsa-miR-186-5p | NASP |
| hsa-miR-186-5p | TLR3 |
| hsa-miR-186-5p | PCDHAC1 |
| hsa-miR-186-5p | PCDHA11 |
| hsa-miR-186-5p | PALD1 |
| hsa-miR-186-5p | PCDHA3 |
| hsa-miR-186-5p | FOXK2 |
| hsa-miR-186-5p | PCDHA5 |
| hsa-miR-186-5p | CCNF |
| hsa-miR-186-5p | CCZ1B |
| hsa-miR-186-5p | DIRAS2 |
| hsa-miR-186-5p | AQPEP |
| hsa-miR-186-5p | PCDHA8 |
| hsa-miR-186-5p | C9orf3 |
| hsa-miR-186-5p | PDS5A |
| hsa-miR-186-5p | SLC26A7 |
| hsa-miR-186-5p | PCDHA9 |
| hsa-miR-186-5p | KIAA1614 |
| hsa-miR-186-5p | ZNF385A |
| hsa-miR-186-5p | PCDHA13 |
| hsa-miR-186-5p | SENP7 |
| hsa-miR-186-5p | STOX2 |
| hsa-miR-186-5p | ARHGAP42 |
| hsa-miR-186-5p | NYAP2 |
| hsa-miR-186-5p | KCNB1 |
| hsa-miR-186-5p | ZC3H18 |
| hsa-miR-186-5p | EHF |
| hsa-miR-186-5p | RAB7L1 |
| hsa-miR-186-5p | DIXDC1 |
| hsa-miR-186-5p | MDFIC |
| hsa-miR-186-5p | PUM2 |
| hsa-miR-186-5p | AKAP1 |
| hsa-miR-186-5p | UBE2W |
| hsa-miR-186-5p | NDRG3 |
| hsa-miR-186-5p | MTSS1L |
| hsa-miR-186-5p | CHMP4C |
| hsa-miR-186-5p | CASK |
| hsa-miR-186-5p | DHX40 |
| hsa-miR-186-5p | EMP2 |
| hsa-miR-186-5p | ATP8A2 |
| hsa-miR-186-5p | CNTN4 |
| hsa-miR-186-5p | CYLD |
| hsa-miR-186-5p | NR4A2 |
| hsa-miR-186-5p | PAN3 |
| hsa-miR-186-5p | UPF1 |
| hsa-miR-186-5p | PIK3CA |
| hsa-miR-186-5p | NARS |
| hsa-miR-186-5p | NMT1 |
| hsa-miR-186-5p | HOOK1 |
| hsa-miR-186-5p | CHERP |
| hsa-miR-186-5p | PDGFA |
| hsa-miR-186-5p | FECH |
| hsa-miR-186-5p | CPEB2 |
| hsa-miR-186-5p | TNIK |
| hsa-miR-186-5p | C20orf112 |
| hsa-miR-186-5p | VPS26B |
| hsa-miR-186-5p | KPNA1 |
| hsa-miR-186-5p | GFPT1 |
| hsa-miR-186-5p | ARIH2 |
| hsa-miR-186-5p | ZCCHC14 |
| hsa-miR-186-5p | PGGT1B |
| hsa-miR-186-5p | PPP1R12A |
| hsa-miR-186-5p | ZNF70 |
| hsa-miR-186-5p | CHSY1 |
| hsa-miR-186-5p | PYGO1 |
| hsa-miR-186-5p | PPP1R15B |
| hsa-miR-186-5p | EIF5 |
| hsa-miR-186-5p | SAMD4A |
| hsa-miR-186-5p | TNKS2 |
| hsa-miR-186-5p | ZNF281 |
| hsa-miR-186-5p | PHLDA3 |
| hsa-miR-186-5p | BACH1 |
| hsa-miR-186-5p | POLH |
| hsa-miR-186-5p | PDP2 |
| hsa-miR-186-5p | ARHGAP26 |
| hsa-miR-186-5p | CELF1 |
| hsa-miR-186-5p | CXXC5 |
| hsa-miR-186-5p | STAG2 |
| hsa-miR-186-5p | SH3PXD2A |
| hsa-miR-186-5p | CHSY3 |
| hsa-miR-186-5p | MME |
| hsa-miR-186-5p | THRB |
| hsa-miR-186-5p | MEX3A |
| hsa-miR-186-5p | TPRG1 |
| hsa-miR-186-5p | DDX3X |
| hsa-miR-186-5p | AKT3 |
| hsa-miR-186-5p | NAA35 |
| hsa-miR-186-5p | MPP3 |
| hsa-miR-186-5p | RAB4A |
| hsa-miR-186-5p | BAZ2B |
| hsa-miR-186-5p | ELP2 |
| hsa-miR-186-5p | FNDC3A |
| hsa-miR-186-5p | SSBP3 |
| hsa-miR-186-5p | PSD3 |
| hsa-miR-186-5p | GNAZ |
| hsa-miR-186-5p | TMEM218 |
| hsa-miR-186-5p | SYT7 |
| hsa-miR-186-5p | PSMD12 |
| hsa-miR-186-5p | SLC35D1 |
| hsa-miR-186-5p | GDF6 |
| hsa-miR-186-5p | MMAA |
| hsa-miR-186-5p | NRG2 |
| hsa-miR-186-5p | SLC23A2 |
| hsa-miR-186-5p | TMEM242 |
| hsa-miR-186-5p | CLDN1 |
| hsa-miR-186-5p | SLC22A15 |
| hsa-miR-186-5p | ATP6V0E1 |
| hsa-miR-186-5p | UBAC1 |
| hsa-miR-186-5p | ZBTB10 |
| hsa-miR-186-5p | HEMK1 |
| hsa-miR-186-5p | TRAF7 |
| hsa-miR-186-5p | DDX52 |
| hsa-miR-186-5p | HNRNPU |
| hsa-miR-186-5p | SPRYD3 |
| hsa-miR-186-5p | VPS53 |
| hsa-miR-186-5p | AP5M1 |
| hsa-miR-186-5p | SLITRK4 |
| hsa-miR-186-5p | SMLR1 |
| hsa-miR-186-5p | HNRNPR |
| hsa-miR-186-5p | FAM175B |
| hsa-miR-186-5p | TMEM30B |
| hsa-miR-186-5p | FASTKD2 |
| hsa-miR-186-5p | RPRD2 |
| hsa-miR-186-5p | PKNOX1 |
| hsa-miR-186-5p | ZBTB20 |
| hsa-miR-186-5p | SENP6 |
| hsa-miR-186-5p | CTNS |
| hsa-miR-186-5p | TMEM167A |
| hsa-miR-186-5p | FBXO47 |
| hsa-miR-186-5p | KIAA1522 |
| hsa-miR-186-5p | C2orf91 |
| hsa-miR-186-5p | HNRNPC |
| hsa-miR-186-5p | LRRC10B |
| hsa-miR-186-5p | HIF1A |
| hsa-miR-186-5p | CDR2L |
| hsa-miR-186-5p | RP11-770J1.4 |
| hsa-miR-186-5p | CELF3 |
| hsa-miR-186-5p | MIP |
| hsa-miR-186-5p | CHTF8 |
| hsa-miR-186-5p | MAPK1IP1L |
| hsa-miR-186-5p | RILPL1 |
| hsa-miR-186-5p | SMAD6 |
| hsa-miR-186-5p | MDGA1 |
| hsa-miR-186-5p | PTCD1 |
| hsa-miR-186-5p | TMED4 |
| hsa-miR-186-5p | ATP5J2-PTCD1 |
| hsa-miR-186-5p | CKAP4 |
| hsa-miR-186-5p | WAC |
| hsa-miR-186-5p | C14orf37 |
| hsa-miR-186-5p | DMD |
| hsa-miR-186-5p | RBM26 |
| hsa-miR-186-5p | RSRC2 |
| hsa-miR-186-5p | HNRNPUL2 |
| hsa-miR-186-5p | BAHD1 |
| hsa-miR-186-5p | SLC22A23 |
| hsa-miR-186-5p | DHX9 |
| hsa-miR-186-5p | DCLRE1C |
| hsa-miR-186-5p | TXNIP |
| hsa-miR-186-5p | SPPL2A |
| hsa-miR-186-5p | UGGT1 |
| hsa-miR-186-5p | PIGV |
| hsa-miR-186-5p | SRPK1 |
| hsa-miR-186-5p | CAB39L |
| hsa-miR-186-5p | VSIG10 |
| hsa-miR-186-5p | SMAD2 |
| hsa-miR-186-5p | ADCY5 |
| hsa-miR-186-5p | IRS1 |
| hsa-miR-186-5p | YAF2 |
| hsa-miR-186-5p | FBXO48 |
| hsa-miR-186-5p | ANTXR2 |
| hsa-miR-186-5p | EIF5B |
| hsa-miR-186-5p | GFRA1 |
| hsa-miR-186-5p | KIF3A |
| hsa-miR-186-5p | BTRC |
| hsa-miR-186-5p | ARFGEF1 |
| hsa-miR-186-5p | SESN3 |
| hsa-miR-186-5p | LARP4B |
| hsa-miR-186-5p | SBNO1 |
| hsa-miR-186-5p | SPTLC2 |
| hsa-miR-186-5p | AGPS |
| hsa-miR-186-5p | TFAP2B |
| hsa-miR-186-5p | WIPF2 |
| hsa-miR-186-5p | NAA40 |
| hsa-miR-186-5p | EXT2 |
| hsa-miR-186-5p | RNF170 |
| hsa-miR-186-5p | LGALS8 |
| hsa-miR-186-5p | MTMR3 |
| hsa-miR-186-5p | SLC16A10 |
| hsa-miR-186-5p | TOP2B |
| hsa-miR-186-5p | KAZN |
| hsa-miR-186-5p | DDR2 |
| hsa-miR-186-5p | AFG3L2 |
| hsa-miR-186-5p | EPC1 |
| hsa-miR-186-5p | SUZ12 |
| hsa-miR-186-5p | MASTL |
| hsa-miR-186-5p | ATP5G3 |
| hsa-miR-186-5p | PANX1 |
| hsa-miR-186-5p | MXI1 |
| hsa-miR-186-5p | SMIM12 |
| hsa-miR-186-5p | TMEM184C |
| hsa-miR-186-5p | LOX |
| hsa-miR-186-5p | MON2 |
| hsa-miR-186-5p | TIGD7 |
| hsa-miR-186-5p | RAB11A |
| hsa-miR-186-5p | ICMT |
| hsa-miR-186-5p | HNRNPA0 |
| hsa-miR-186-5p | GNAI1 |
| hsa-miR-186-5p | IFT74 |
| hsa-miR-186-5p | DDX42 |
| hsa-miR-186-5p | OTUD6A |
| hsa-miR-186-5p | TTC37 |
| hsa-miR-186-5p | ABI2 |
| hsa-miR-186-5p | FAM168A |
| hsa-miR-186-5p | ARMC8 |
| hsa-miR-186-5p | TFAP2A |
| hsa-miR-186-5p | TFDP2 |
| hsa-miR-186-5p | IGSF10 |
| hsa-miR-186-5p | ATXN7L3B |
| hsa-miR-186-5p | ING2 |
| hsa-miR-186-5p | AIDA |
| hsa-miR-186-5p | GLRX2 |
| hsa-miR-186-5p | LAMTOR5 |
| hsa-miR-186-5p | SUV420H1 |
| hsa-miR-186-5p | DTD2 |
| hsa-miR-186-5p | SMURF2 |
| hsa-miR-186-5p | MTDH |
| hsa-miR-186-5p | XRCC5 |
| hsa-miR-186-5p | CADM1 |
| hsa-miR-186-5p | TOMM6 |
| hsa-miR-186-5p | AHSA2 |
| hsa-miR-186-5p | FAM216A |
| hsa-miR-186-5p | KIAA1328 |
| hsa-miR-186-5p | MURC |
| hsa-miR-186-5p | MMAB |
| hsa-miR-186-5p | RAPGEF4 |
| hsa-miR-186-5p | DAZAP2 |
| hsa-miR-186-5p | MBD2 |
| hsa-miR-186-5p | KCMF1 |
| hsa-miR-186-5p | INVS |
| hsa-miR-186-5p | BBIP1 |
| hsa-miR-186-5p | GLP2R |
| hsa-miR-186-5p | SEC31B |
| hsa-miR-186-5p | NDUFB9 |
| hsa-miR-186-5p | ATG7 |
| hsa-miR-186-5p | TRIP4 |
| hsa-miR-186-5p | GID4 |
| hsa-miR-186-5p | TMEM30C |
| hsa-miR-186-5p | CCNDBP1 |
| hsa-miR-186-5p | TMBIM6 |
| hsa-miR-186-5p | PDCD6IP |
| hsa-miR-186-5p | NLE1 |
| hsa-miR-186-5p | SMNDC1 |
| hsa-miR-186-5p | EIF2B2 |
| hsa-miR-186-5p | OCIAD2 |
| hsa-miR-186-5p | RGPD4 |
| hsa-miR-186-5p | GPR65 |
| hsa-miR-186-5p | LETMD1 |
| hsa-miR-186-5p | CMC1 |
| hsa-miR-186-5p | ASAP3 |
| hsa-miR-186-5p | METTL24 |
| hsa-miR-186-5p | SLC10A6 |
| hsa-miR-186-5p | NUP37 |
| hsa-miR-186-5p | FAM229B |
| hsa-miR-186-5p | GTF3C2 |
| hsa-miR-186-5p | TTPA |
| hsa-miR-186-5p | BSND |
| hsa-miR-186-5p | CACNG2 |

**Table S5.** Raw counts of RNA-sequencing data of hsa-let-7d-3p in LUAD from the TCGA.

| sampleID | time | Staus | RS | Label |
| --- | --- | --- | --- | --- |
| TCGA-05-4244-01 | 0 | 0 | 7.738765405 | Low groups |
| TCGA-05-4249-01 | 4.17260274 | 0 | 7.3902553 | Low groups |
| TCGA-05-4250-01 | 0.331506849 | 0 | 7.795614949 | Low groups |
| TCGA-05-4382-01 | 0.915068493 | 1 | 8.215673549 | High groups |
| TCGA-05-4384-01 | 0.501369863 | 1 | 7.949783881 | Low groups |
| TCGA-05-4389-01 | 3.750684932 | 0 | 7.982812343 | Low groups |
| TCGA-05-4390-01 | 1.082191781 | 1 | 7.820882839 | Low groups |
| TCGA-05-4395-01 | 0 | 0 | 8.908573059 | High groups |
| TCGA-05-4396-01 | 0.830136986 | 0 | 8.586852733 | High groups |
| TCGA-05-4397-01 | 2.002739726 | 0 | 10.08054548 | High groups |
| TCGA-05-4398-01 | 3.920547945 | 0 | 8.019455515 | Low groups |
| TCGA-05-4402-01 | 0.668493151 | 0 | 8.063749374 | Low groups |
| TCGA-05-4403-01 | 1.583561644 | 0 | 8.108415728 | Low groups |
| TCGA-05-4405-01 | 1.671232877 | 0 | 7.753828937 | Low groups |
| TCGA-05-4410-01 | 0 | 0 | 7.99213411 | Low groups |
| TCGA-05-4415-01 | 0.164383562 | 1 | 6.527913033 | Low groups |
| TCGA-05-4417-01 | 1.246575342 | 0 | 8.131794725 | Low groups |
| TCGA-05-4418-01 | 0.750684932 | 0 | 7.702754842 | Low groups |
| TCGA-05-4420-01 | 2.498630137 | 0 | 8.472243727 | High groups |
| TCGA-05-4422-01 | 1 | 0 | 8.80499025 | High groups |
| TCGA-05-4424-01 | 0.419178082 | 1 | 9.019437277 | High groups |
| TCGA-05-4425-01 | 1.832876712 | 0 | 8.900169377 | High groups |
| TCGA-05-4426-01 | 1.252054795 | 1 | 8.699006855 | High groups |
| TCGA-05-4427-01 | 2.167123288 | 0 | 8.237829219 | High groups |
| TCGA-05-4430-01 | 2.084931507 | 0 | 7.886794417 | Low groups |
| TCGA-05-4432-01 | 2.084931507 | 0 | 7.094476569 | Low groups |
| TCGA-05-4433-01 | 2 | 0 | 9.259068593 | High groups |
| TCGA-05-4434-01 | 1.252054795 | 0 | 7.891872692 | Low groups |
| TCGA-05-5420-01 | 0.671232877 | 1 | 6.924676065 | Low groups |
| TCGA-05-5423-01 | 0.41369863 | 0 | 7.167978555 | Low groups |
| TCGA-05-5425-01 | 1.331506849 | 1 | 8.234528336 | High groups |
| TCGA-05-5428-01 | 1.835616438 | 0 | 6.89635711 | Low groups |
| TCGA-05-5429-01 | 0.753424658 | 0 | 7.816502687 | Low groups |
| TCGA-05-5715-01 | 0.169863014 | 0 | 7.927146889 | Low groups |
| TCGA-35-3615-01 | 0.038356164 | 0 | 7.0397986 | Low groups |
| TCGA-35-4122-01 | 0.616438356 | 0 | 6.339817052 | Low groups |
| TCGA-35-4123-01 | 0.498630137 | 0 | 8.450094859 | High groups |
| TCGA-35-5375-01 | 0.723287671 | 0 | 8.784016415 | High groups |
| TCGA-38-4625-01 | 8.145205479 | 0 | 8.905452203 | High groups |
| TCGA-38-4626-01 | 6.898630137 | 1 | 9.022680456 | High groups |
| TCGA-38-4627-01 | 3.142465753 | 0 | 8.591692281 | High groups |
| TCGA-38-4628-01 | 2.967123288 | 1 | 7.852453229 | Low groups |
| TCGA-38-4629-01 | 1.038356164 | 1 | 7.35657055 | Low groups |
| TCGA-38-4630-01 | 1.435616438 | 1 | 8.188463881 | High groups |
| TCGA-38-4631-01 | 0.969863014 | 1 | 5.942876354 | Low groups |
| TCGA-38-4632-01 | 1.863013699 | 1 | 8.087248012 | Low groups |
| TCGA-38-6178-01 | 1.22739726 | 0 | 7.96520738 | Low groups |
| TCGA-38-7271-01 | 0.832876712 | 1 | 7.773836742 | Low groups |
| TCGA-38-A44F-01 | 0.364383562 | 0 | 7.290998441 | Low groups |
| TCGA-44-2655-01 | 2.764383562 | 1 | 6.944832215 | Low groups |
| TCGA-44-2656-01 | 1.556164384 | 1 | 7.545945209 | Low groups |
| TCGA-44-2657-01 | 3.701369863 | 0 | 7.759571321 | Low groups |
| TCGA-44-2659-01 | 3.139726027 | 1 | 7.513325243 | Low groups |
| TCGA-44-2661-01 | 3.175342466 | 0 | 7.139125029 | Low groups |
| TCGA-44-2662-01 | 0.671232877 | 1 | 7.786279496 | Low groups |
| TCGA-44-2665-01 | 3.564383562 | 0 | 7.455590992 | Low groups |
| TCGA-44-2666-01 | 0.265753425 | 1 | 7.696894868 | Low groups |
| TCGA-44-2668-01 | 1.18630137 | 1 | 8.411458237 | High groups |
| TCGA-44-3396-01 | 3.095890411 | 0 | 8.075121841 | Low groups |
| TCGA-44-3398-01 | 3.18630137 | 0 | 8.169942434 | Low groups |
| TCGA-44-3918-01 | 1.4 | 1 | 8.036876542 | Low groups |
| TCGA-44-3919-01 | 2.523287671 | 1 | 8.949030164 | High groups |
| TCGA-44-4112-01 | 1.695890411 | 1 | 8.561646407 | High groups |
| TCGA-44-5643-01 | 2.775342466 | 0 | 10.04932527 | High groups |
| TCGA-44-5644-01 | 2.364383562 | 0 | 8.667657746 | High groups |
| TCGA-44-5645-01 | 2.334246575 | 0 | 8.3727956 | High groups |
| TCGA-44-6144-01 | 1.093150685 | 1 | 9.109557757 | High groups |
| TCGA-44-6145-01 | 1.630136986 | 0 | 9.556447043 | High groups |
| TCGA-44-6146-01 | 1.728767123 | 1 | 10.05938164 | High groups |
| TCGA-44-6147-01 | 2.315068493 | 0 | 9.465985889 | High groups |
| TCGA-44-6148-01 | 1.928767123 | 0 | 8.851157696 | High groups |
| TCGA-44-6774-01 | 1.802739726 | 0 | 7.558342568 | Low groups |
| TCGA-44-6775-01 | 1.873972603 | 1 | 10.19354791 | High groups |
| TCGA-44-6776-01 | 7.167123288 | 0 | 9.046531986 | High groups |
| TCGA-44-6777-01 | 2.704109589 | 0 | 7.738732039 | Low groups |
| TCGA-44-6778-01 | 5.106849315 | 0 | 8.086061049 | Low groups |
| TCGA-44-6779-01 | 0.635616438 | 1 | 8.273052152 | High groups |
| TCGA-44-7659-01 | 1.893150685 | 0 | 8.484544759 | High groups |
| TCGA-44-7660-01 | 0.693150685 | 1 | 8.33339645 | High groups |
| TCGA-44-7661-01 | 0.917808219 | 1 | 7.247547807 | Low groups |
| TCGA-44-7662-01 | 0.597260274 | 0 | 7.380736634 | Low groups |
| TCGA-44-7667-01 | 3.005479452 | 0 | 7.133351035 | Low groups |
| TCGA-44-7669-01 | 1.068493151 | 1 | 7.535196201 | Low groups |
| TCGA-44-7670-01 | 2.416438356 | 0 | 7.959968906 | Low groups |
| TCGA-44-7671-01 | 2.419178082 | 1 | 8.049624397 | Low groups |
| TCGA-44-7672-01 | 1.969863014 | 0 | 7.574424746 | Low groups |
| TCGA-44-8117-01 | 1.054794521 | 0 | 8.771966932 | High groups |
| TCGA-44-8119-01 | 0.780821918 | 0 | 7.671216649 | Low groups |
| TCGA-44-8120-01 | 0.712328767 | 0 | 8.520786255 | High groups |
| TCGA-44-A479-01 | 1.197260274 | 1 | 7.377508845 | Low groups |
| TCGA-44-A47A-01 | 1.087671233 | 1 | 8.055289486 | Low groups |
| TCGA-44-A47B-01 | 0.78630137 | 0 | 7.378046574 | Low groups |
| TCGA-44-A47F-01 | 0.923287671 | 0 | 7.480869108 | Low groups |
| TCGA-44-A47G-01 | 0.961643836 | 0 | 7.200142077 | Low groups |
| TCGA-44-A4SS-01 | 1.136986301 | 0 | 7.322339295 | Low groups |
| TCGA-44-A4SU-01 | 0.712328767 | 1 | 8.695227456 | High groups |
| TCGA-49-4486-01 | 5.602739726 | 1 | 7.745559643 | Low groups |
| TCGA-49-4487-01 | 1.909589041 | 1 | 8.296117067 | High groups |
| TCGA-49-4488-01 | 1.736986301 | 1 | 9.553197477 | High groups |
| TCGA-49-4490-01 | 1.054794521 | 1 | 8.015819452 | Low groups |
| TCGA-49-4494-01 | 2.961643836 | 1 | 7.46676308 | Low groups |
| TCGA-49-4501-01 | 1.493150685 | 1 | 8.024607412 | Low groups |
| TCGA-49-4505-01 | 1.142465753 | 1 | 8.095932329 | Low groups |
| TCGA-49-4506-01 | 1.895890411 | 1 | 8.380733863 | High groups |
| TCGA-49-4507-01 | 0.432876712 | 1 | 8.150568206 | Low groups |
| TCGA-49-4510-01 | 1.375342466 | 1 | 6.923908607 | Low groups |
| TCGA-49-4512-01 | 2.479452055 | 1 | 8.413949855 | High groups |
| TCGA-49-4514-01 | 4.657534247 | 0 | 8.038192796 | Low groups |
| TCGA-49-6742-01 | 0.58630137 | 1 | 8.254647327 | High groups |
| TCGA-49-6743-01 | 4.44109589 | 0 | 8.148466413 | Low groups |
| TCGA-49-6744-01 | 4.610958904 | 0 | 7.599123755 | Low groups |
| TCGA-49-6745-01 | 1.430136986 | 0 | 7.805090756 | Low groups |
| TCGA-49-6761-01 | 0.969863014 | 0 | 8.497441518 | High groups |
| TCGA-49-6767-01 | 1.854794521 | 0 | 6.491988062 | Low groups |
| TCGA-49-AAQV-01 | 1.394520548 | 1 | 7.305102218 | Low groups |
| TCGA-49-AAR0-01 | 13.05479452 | 0 | 8.628050208 | High groups |
| TCGA-49-AAR2-01 | 6.093150685 | 0 | 6.415803784 | Low groups |
| TCGA-49-AAR3-01 | 5.18630137 | 1 | 7.851886372 | Low groups |
| TCGA-49-AAR4-01 | 2.408219178 | 1 | 7.754150337 | Low groups |
| TCGA-49-AAR9-01 | 0.712328767 | 1 | 8.121833681 | Low groups |
| TCGA-49-AARE-01 | 1.063013699 | 1 | 7.753408851 | Low groups |
| TCGA-49-AARN-01 | 3.109589041 | 0 | 8.069544664 | Low groups |
| TCGA-49-AARO-01 | 3.134246575 | 1 | 7.778492001 | Low groups |
| TCGA-49-AARQ-01 | 18.44383562 | 0 | 9.689695428 | High groups |
| TCGA-49-AARR-01 | 3.010958904 | 1 | 8.110205941 | Low groups |
| TCGA-4B-A93V-01 | 0.687671233 | 1 | 10.10778096 | High groups |
| TCGA-50-5044-01 | 1.323287671 | 1 | 8.339541839 | High groups |
| TCGA-50-5045-01 | 3.926027397 | 1 | 7.335675114 | Low groups |
| TCGA-50-5049-01 | 4.295890411 | 1 | 7.571592175 | Low groups |
| TCGA-50-5051-01 | 0.504109589 | 1 | 8.477122977 | High groups |
| TCGA-50-5055-01 | 2.112328767 | 1 | 7.378367867 | Low groups |
| TCGA-50-5066-01 | 1.306849315 | 1 | 6.892714503 | Low groups |
| TCGA-50-5068-01 | 1.879452055 | 1 | 7.37384315 | Low groups |
| TCGA-50-5072-01 | 0.583561644 | 1 | 7.709709086 | Low groups |
| TCGA-50-5930-01 | 0.484931507 | 1 | 8.344081308 | High groups |
| TCGA-50-5931-01 | 1.164383562 | 1 | 9.696426571 | High groups |
| TCGA-50-5932-01 | 2.98630137 | 1 | 8.3110135 | High groups |
| TCGA-50-5933-01 | 6.556164384 | 0 | 8.060481943 | Low groups |
| TCGA-50-5935-01 | 1.789041096 | 0 | 8.826803415 | High groups |
| TCGA-50-5936-01 | 0.410958904 | 1 | 7.938721334 | Low groups |
| TCGA-50-5939-01 | 1.260273973 | 0 | 8.673146014 | High groups |
| TCGA-50-5941-01 | 4.038356164 | 0 | 8.374908192 | High groups |
| TCGA-50-5942-01 | 3.775342466 | 1 | 9.169775464 | High groups |
| TCGA-50-5944-01 | 4.794520548 | 0 | 8.399126778 | High groups |
| TCGA-50-5946-01 | 0.605479452 | 1 | 8.750362708 | High groups |
| TCGA-50-6590-01 | 3.528767123 | 0 | 9.353645859 | High groups |
| TCGA-50-6591-01 | 0.326027397 | 1 | 8.838508512 | High groups |
| TCGA-50-6592-01 | 2.128767123 | 1 | 8.969659061 | High groups |
| TCGA-50-6593-01 | 0.728767123 | 1 | 8.504449738 | High groups |
| TCGA-50-6594-01 | 0.780821918 | 1 | 7.143159305 | Low groups |
| TCGA-50-6595-01 | 0.498630137 | 1 | 8.084314865 | Low groups |
| TCGA-50-6597-01 | 3.473972603 | 0 | 9.248243306 | High groups |
| TCGA-50-6673-01 | 0.060273973 | 0 | 8.851172848 | High groups |
| TCGA-50-7109-01 | 0.04109589 | 1 | 7.539230324 | Low groups |
| TCGA-50-8457-01 | 3.082191781 | 0 | 8.554208828 | High groups |
| TCGA-50-8459-01 | 1.183561644 | 1 | 7.384291618 | Low groups |
| TCGA-50-8460-01 | 2.271232877 | 0 | 7.097557706 | Low groups |
| TCGA-53-7624-01 | 1.095890411 | 1 | 8.215794869 | High groups |
| TCGA-53-7626-01 | 2.369863014 | 1 | 7.828774049 | Low groups |
| TCGA-53-7813-01 | 1.161643836 | 0 | 8.552806637 | High groups |
| TCGA-53-A4EZ-01 | 2.934246575 | 0 | 9.114561978 | High groups |
| TCGA-55-1592-01 | 1.238356164 | 1 | 8.039934772 | Low groups |
| TCGA-55-1594-01 | 3.22739726 | 0 | 6.762786315 | Low groups |
| TCGA-55-1595-01 | 4.052054795 | 0 | 8.686862556 | High groups |
| TCGA-55-1596-01 | 5.657534247 | 0 | 8.529627247 | High groups |
| TCGA-55-5899-01 | 2.547945205 | 0 | 7.324982291 | Low groups |
| TCGA-55-6543-01 | 1.191780822 | 0 | 9.083908907 | High groups |
| TCGA-55-6642-01 | 6.709589041 | 0 | 7.625925487 | Low groups |
| TCGA-55-6712-01 | 0.468493151 | 1 | 7.393604025 | Low groups |
| TCGA-55-6968-01 | 3.542465753 | 1 | 8.436758049 | High groups |
| TCGA-55-6969-01 | 3.394520548 | 0 | 8.663521956 | High groups |
| TCGA-55-6970-01 | 1.252054795 | 1 | 9.23157365 | High groups |
| TCGA-55-6971-01 | 3.835616438 | 0 | 9.00683798 | High groups |
| TCGA-55-6972-01 | 4.471232877 | 0 | 9.219961785 | High groups |
| TCGA-55-6978-01 | 0.115068493 | 1 | 8.618712696 | High groups |
| TCGA-55-6979-01 | 0.534246575 | 1 | 9.392476368 | High groups |
| TCGA-55-6980-01 | 5.778082192 | 0 | 9.392225963 | High groups |
| TCGA-55-6981-01 | 3.778082192 | 0 | 8.61397727 | High groups |
| TCGA-55-6982-01 | 0.501369863 | 1 | 8.788329332 | High groups |
| TCGA-55-6983-01 | 7.734246575 | 0 | 8.552901716 | High groups |
| TCGA-55-6984-01 | 1.983561644 | 1 | 8.890841939 | High groups |
| TCGA-55-6985-01 | 3.378082192 | 0 | 8.677543301 | High groups |
| TCGA-55-6986-01 | 8.934246575 | 0 | 9.464311306 | High groups |
| TCGA-55-6987-01 | 5.854794521 | 0 | 9.090797947 | High groups |
| TCGA-55-7227-01 | 0.698630137 | 1 | 8.104311601 | Low groups |
| TCGA-55-7281-01 | 0.928767123 | 1 | 7.503251169 | Low groups |
| TCGA-55-7283-01 | 1.668493151 | 0 | 8.594874328 | High groups |
| TCGA-55-7284-01 | 0.632876712 | 1 | 8.513155654 | High groups |
| TCGA-55-7570-01 | 2.257534247 | 0 | 8.404952922 | High groups |
| TCGA-55-7573-01 | 1.334246575 | 0 | 8.333200979 | High groups |
| TCGA-55-7574-01 | 1.309589041 | 1 | 8.366001988 | High groups |
| TCGA-55-7576-01 | 1.835616438 | 0 | 7.907585146 | Low groups |
| TCGA-55-7724-01 | 1.931506849 | 0 | 8.533275801 | High groups |
| TCGA-55-7725-01 | 1.210958904 | 0 | 9.009610867 | High groups |
| TCGA-55-7726-01 | 1.78630137 | 0 | 8.035466326 | Low groups |
| TCGA-55-7727-01 | 0.326027397 | 0 | 9.948764137 | High groups |
| TCGA-55-7728-01 | 1.928767123 | 0 | 7.421382795 | Low groups |
| TCGA-55-7815-01 | 1.276712329 | 1 | 7.375734539 | Low groups |
| TCGA-55-7816-01 | 1.282191781 | 1 | 8.082971196 | Low groups |
| TCGA-55-7903-01 | 1.553424658 | 0 | 8.608649074 | High groups |
| TCGA-55-7907-01 | 0.805479452 | 1 | 9.079516784 | High groups |
| TCGA-55-7910-01 | 2.789041096 | 1 | 8.872960559 | High groups |
| TCGA-55-7911-01 | 1.410958904 | 1 | 8.130037658 | Low groups |
| TCGA-55-7913-01 | 1.315068493 | 1 | 9.077698497 | High groups |
| TCGA-55-7914-01 | 0.512328767 | 1 | 8.97707066 | High groups |
| TCGA-55-7994-01 | 1.652054795 | 0 | 8.105059032 | Low groups |
| TCGA-55-7995-01 | 1.282191781 | 1 | 9.885549107 | High groups |
| TCGA-55-8085-01 | 2.476712329 | 0 | 7.89139391 | Low groups |
| TCGA-55-8087-01 | 1.265753425 | 0 | 9.654208885 | High groups |
| TCGA-55-8089-01 | 1.923287671 | 0 | 8.697336566 | High groups |
| TCGA-55-8090-01 | 1.501369863 | 1 | 8.468117494 | High groups |
| TCGA-55-8091-01 | 1.643835616 | 0 | 8.306522184 | High groups |
| TCGA-55-8092-01 | 0.347945205 | 1 | 9.422255921 | High groups |
| TCGA-55-8094-01 | 1.482191781 | 0 | 8.077843018 | Low groups |
| TCGA-55-8096-01 | 1.550684932 | 1 | 9.012084677 | High groups |
| TCGA-55-8097-01 | 1.304109589 | 0 | 9.703108834 | High groups |
| TCGA-55-8203-01 | 1.498630137 | 0 | 8.671562862 | High groups |
| TCGA-55-8204-01 | 1.410958904 | 0 | 7.785401397 | Low groups |
| TCGA-55-8205-01 | 1.356164384 | 1 | 8.769753891 | High groups |
| TCGA-55-8206-01 | 2.432876712 | 0 | 8.363970673 | High groups |
| TCGA-55-8207-01 | 2.676712329 | 0 | 8.289369949 | High groups |
| TCGA-55-8208-01 | 1.4 | 1 | 8.55970135 | High groups |
| TCGA-55-8299-01 | 0.750684932 | 1 | 8.875353727 | High groups |
| TCGA-55-8301-01 | 0.652054795 | 1 | 8.951212316 | High groups |
| TCGA-55-8302-01 | 1.309589041 | 0 | 7.356482607 | Low groups |
| TCGA-55-8505-01 | 1.205479452 | 0 | 8.590641688 | High groups |
| TCGA-55-8506-01 | 0.030136986 | 0 | 8.523839374 | High groups |
| TCGA-55-8507-01 | 1.145205479 | 0 | 8.521717421 | High groups |
| TCGA-55-8508-01 | 1.690410959 | 0 | 7.997228492 | Low groups |
| TCGA-55-8510-01 | 1.476712329 | 0 | 9.018113364 | High groups |
| TCGA-55-8511-01 | 1.260273973 | 1 | 9.072794846 | High groups |
| TCGA-55-8512-01 | 1.663013699 | 1 | 8.78543769 | High groups |
| TCGA-55-8513-01 | 0.868493151 | 1 | 9.164199405 | High groups |
| TCGA-55-8514-01 | 1.424657534 | 0 | 9.148711716 | High groups |
| TCGA-55-8614-01 | 1.468493151 | 0 | 7.992018513 | Low groups |
| TCGA-55-8615-01 | 0.61369863 | 1 | 8.57790129 | High groups |
| TCGA-55-8616-01 | 0.131506849 | 0 | 9.108246711 | High groups |
| TCGA-55-8619-01 | 1.139726027 | 0 | 9.010943609 | High groups |
| TCGA-55-8620-01 | 1.02739726 | 1 | 8.461468069 | High groups |
| TCGA-55-8621-01 | 1.410958904 | 0 | 8.851811276 | High groups |
| TCGA-55-A48X-01 | 1.684931507 | 1 | 7.761394323 | Low groups |
| TCGA-55-A48Y-01 | 1.726027397 | 0 | 7.56671334 | Low groups |
| TCGA-55-A48Z-01 | 1.468493151 | 1 | 8.295350366 | High groups |
| TCGA-55-A490-01 | 0.271232877 | 0 | 6.762495789 | Low groups |
| TCGA-55-A491-01 | 1.715068493 | 0 | 7.113888511 | Low groups |
| TCGA-55-A492-01 | 1.632876712 | 0 | 7.430261852 | Low groups |
| TCGA-55-A493-01 | 0.076712329 | 0 | 6.879298236 | Low groups |
| TCGA-55-A494-01 | 1.317808219 | 0 | 7.111919991 | Low groups |
| TCGA-55-A4DF-01 | 1.410958904 | 1 | 8.071664165 | Low groups |
| TCGA-55-A4DG-01 | 1.665753425 | 0 | 7.796274705 | Low groups |
| TCGA-55-A57B-01 | 1.495890411 | 0 | 8.104174138 | Low groups |
| TCGA-62-8394-01 | 0.380821918 | 0 | 8.872106801 | High groups |
| TCGA-62-8395-01 | 1.082191781 | 1 | 8.029125415 | Low groups |
| TCGA-62-8397-01 | 3.531506849 | 0 | 8.487691256 | High groups |
| TCGA-62-8398-01 | 1.216438356 | 0 | 7.686072347 | Low groups |
| TCGA-62-8399-01 | 7.38630137 | 0 | 9.050001903 | High groups |
| TCGA-62-8402-01 | 2.115068493 | 1 | 6.623243125 | Low groups |
| TCGA-62-A46O-01 | 2.583561644 | 1 | 5.04333698 | Low groups |
| TCGA-62-A46P-01 | 0.731506849 | 1 | 7.307017882 | Low groups |
| TCGA-62-A46R-01 | 4.726027397 | 0 | 7.322086602 | Low groups |
| TCGA-62-A46S-01 | 1.443835616 | 1 | 7.687791722 | Low groups |
| TCGA-62-A46U-01 | 5.663013699 | 0 | 7.881903784 | Low groups |
| TCGA-62-A46V-01 | 6.024657534 | 0 | 7.679969266 | Low groups |
| TCGA-62-A46Y-01 | 0.860273973 | 1 | 9.268519076 | High groups |
| TCGA-62-A470-01 | 1.476712329 | 1 | 7.778780625 | Low groups |
| TCGA-62-A471-01 | 3.41369863 | 0 | 8.27577672 | High groups |
| TCGA-62-A472-01 | 0.794520548 | 1 | 8.017526504 | Low groups |
| TCGA-64-1676-01 | 4.734246575 | 0 | 6.349448348 | Low groups |
| TCGA-64-1677-01 | 0.98630137 | 1 | 8.084662783 | Low groups |
| TCGA-64-1678-01 | 3.257534247 | 0 | 7.50685579 | Low groups |
| TCGA-64-1679-01 | 6.816438356 | 0 | 7.232749388 | Low groups |
| TCGA-64-1680-01 | 3.084931507 | 0 | 6.914672028 | Low groups |
| TCGA-64-1681-01 | 1.202739726 | 1 | 7.776580617 | Low groups |
| TCGA-64-5774-01 | 0.673972603 | 1 | 8.159694621 | Low groups |
| TCGA-64-5775-01 | 0.169863014 | 1 | 8.048375705 | Low groups |
| TCGA-64-5778-01 | 2.635616438 | 1 | 8.095516759 | Low groups |
| TCGA-64-5779-01 | 2.178082192 | 1 | 8.077768743 | Low groups |
| TCGA-64-5781-01 | 0.263013699 | 1 | 8.706799893 | High groups |
| TCGA-64-5815-01 | 2.37260274 | 0 | 7.607077413 | Low groups |
| TCGA-67-3770-01 | 1.671232877 | 0 | 7.322229677 | Low groups |
| TCGA-67-3771-01 | 1.671232877 | 0 | 6.624570747 | Low groups |
| TCGA-67-3772-01 | 1.569863014 | 0 | 6.749449661 | Low groups |
| TCGA-67-3773-01 | 1.169863014 | 0 | 7.356922448 | Low groups |
| TCGA-67-3774-01 | 1.054794521 | 0 | 8.643548502 | High groups |
| TCGA-67-4679-01 | 1.22739726 | 0 | 8.671456855 | High groups |
| TCGA-67-6215-01 | 0.476712329 | 0 | 8.713814087 | High groups |
| TCGA-67-6216-01 | 0.38630137 | 0 | 8.021993552 | Low groups |
| TCGA-67-6217-01 | 0.810958904 | 1 | 9.025429041 | High groups |
| TCGA-69-7760-01 | 0.553424658 | 0 | 7.660855404 | Low groups |
| TCGA-69-7761-01 | 0.509589041 | 0 | 7.665920552 | Low groups |
| TCGA-69-7763-01 | 1.890410959 | 0 | 8.17048579 | High groups |
| TCGA-69-7764-01 | 1.134246575 | 0 | 8.480502037 | High groups |
| TCGA-69-7765-01 | 0.452054795 | 0 | 7.804029738 | Low groups |
| TCGA-69-7973-01 | 0.630136986 | 0 | 7.432867984 | Low groups |
| TCGA-69-7974-01 | 0.504109589 | 0 | 8.309647193 | High groups |
| TCGA-69-7978-01 | 0.367123288 | 0 | 8.871259037 | High groups |
| TCGA-69-7979-01 | 1.117808219 | 0 | 9.365954215 | High groups |
| TCGA-69-7980-01 | 1.126027397 | 0 | 8.024133091 | Low groups |
| TCGA-69-8253-01 | 1.167123288 | 0 | 9.090329205 | High groups |
| TCGA-69-8254-01 | 1.120547945 | 0 | 9.712061274 | High groups |
| TCGA-69-8255-01 | 0.353424658 | 0 | 7.604011704 | Low groups |
| TCGA-69-8453-01 | 0.942465753 | 1 | 9.377806793 | High groups |
| TCGA-69-A59K-01 | 1.619178082 | 0 | 7.36269527 | Low groups |
| TCGA-71-6725-01 | 0.449315068 | 1 | 8.800146882 | High groups |
| TCGA-71-8520-01 | 0.490410959 | 1 | 8.05293566 | Low groups |
| TCGA-73-4658-01 | 4.383561644 | 0 | 6.760226137 | Low groups |
| TCGA-73-4659-01 | 0.095890411 | 1 | 8.991195375 | High groups |
| TCGA-73-4662-01 | 0.057534247 | 1 | 8.102958627 | Low groups |
| TCGA-73-4675-01 | 0.989041096 | 1 | 9.046820423 | High groups |
| TCGA-73-4676-01 | 0.769863014 | 1 | 6.905656744 | Low groups |
| TCGA-73-7498-01 | 3.257534247 | 0 | 8.447893223 | High groups |
| TCGA-73-7499-01 | 3.964383562 | 1 | 7.391261808 | Low groups |
| TCGA-73-A9RS-01 | 0.293150685 | 1 | 8.506424896 | High groups |
| TCGA-75-5125-01 | 4.8 | 1 | 8.582313815 | High groups |
| TCGA-75-5146-01 | 4.857534247 | 1 | 8.029628463 | Low groups |
| TCGA-75-5147-01 | 3.652054795 | 0 | 8.35910677 | High groups |
| TCGA-75-6206-01 | 7.095890411 | 0 | 9.229140063 | High groups |
| TCGA-75-6212-01 | 3.671232877 | 1 | 8.721944729 | High groups |
| TCGA-75-6214-01 | 1.147945205 | 1 | 9.742649377 | High groups |
| TCGA-75-7025-01 | 4.057534247 | 1 | 8.594152702 | High groups |
| TCGA-75-7027-01 | 8.079452055 | 1 | 8.43813771 | High groups |
| TCGA-78-7143-01 | 4.109589041 | 1 | 8.480194101 | High groups |
| TCGA-78-7145-01 | 1.156164384 | 1 | 7.715166213 | Low groups |
| TCGA-78-7146-01 | 0.473972603 | 0 | 8.29359975 | High groups |
| TCGA-78-7147-01 | 1.583561644 | 1 | 8.45625904 | High groups |
| TCGA-78-7148-01 | 0.498630137 | 1 | 7.72389476 | Low groups |
| TCGA-78-7149-01 | 10.79452055 | 0 | 8.113159971 | Low groups |
| TCGA-78-7150-01 | 0.380821918 | 1 | 7.384371061 | Low groups |
| TCGA-78-7152-01 | 3.293150685 | 1 | 8.371527804 | High groups |
| TCGA-78-7153-01 | 9.95890411 | 0 | 7.853471565 | Low groups |
| TCGA-78-7154-01 | 1.624657534 | 0 | 7.91231358 | Low groups |
| TCGA-78-7155-01 | 0.57260274 | 1 | 5.92835265 | Low groups |
| TCGA-78-7156-01 | 2.673972603 | 0 | 8.364258384 | High groups |
| TCGA-78-7158-01 | 0.361643836 | 1 | 8.798508674 | High groups |
| TCGA-78-7159-01 | 5.408219178 | 0 | 8.076826284 | Low groups |
| TCGA-78-7160-01 | 1.909589041 | 0 | 8.631602342 | High groups |
| TCGA-78-7161-01 | 0.443835616 | 1 | 8.588461898 | High groups |
| TCGA-78-7162-01 | 6.076712329 | 1 | 9.573896703 | High groups |
| TCGA-78-7163-01 | 19.85753425 | 0 | 8.819563451 | High groups |
| TCGA-78-7166-01 | 0.706849315 | 0 | 7.603720173 | Low groups |
| TCGA-78-7167-01 | 1.989041096 | 1 | 8.180804079 | High groups |
| TCGA-78-7220-01 | 1.454794521 | 1 | 6.832055465 | Low groups |
| TCGA-78-7535-01 | 2.216438356 | 1 | 8.439284571 | High groups |
| TCGA-78-7536-01 | 0.632876712 | 1 | 9.364313831 | High groups |
| TCGA-78-7537-01 | 4.443835616 | 0 | 8.300874948 | High groups |
| TCGA-78-7539-01 | 1.884931507 | 1 | 8.64799137 | High groups |
| TCGA-78-7540-01 | 3.279452055 | 0 | 7.730672763 | Low groups |
| TCGA-78-7542-01 | 0.879452055 | 0 | 8.433738236 | High groups |
| TCGA-78-7633-01 | 3.97260274 | 1 | 7.371035355 | Low groups |
| TCGA-78-8640-01 | 19.34794521 | 0 | 8.309622321 | High groups |
| TCGA-78-8648-01 | 1.97260274 | 1 | 8.800881041 | High groups |
| TCGA-78-8655-01 | 6.465753425 | 0 | 8.137219465 | Low groups |
| TCGA-78-8660-01 | 0.663013699 | 1 | 8.909767405 | High groups |
| TCGA-78-8662-01 | 8.339726027 | 1 | 9.993465623 | High groups |
| TCGA-80-5608-01 | 7.75890411 | 0 | 9.045198744 | High groups |
| TCGA-80-5611-01 | 7.109589041 | 0 | 8.505659934 | High groups |
| TCGA-83-5908-01 | 2.257534247 | 0 | 8.89333087 | High groups |
| TCGA-86-6562-01 | 0.750684932 | 1 | 8.58167712 | High groups |
| TCGA-86-6851-01 | 0.490410959 | 0 | 9.449963342 | High groups |
| TCGA-86-7701-01 | 1.161643836 | 1 | 7.824017294 | Low groups |
| TCGA-86-7711-01 | 2.865753425 | 1 | 7.751257366 | Low groups |
| TCGA-86-7713-01 | 3.169863014 | 0 | 7.774139546 | Low groups |
| TCGA-86-7714-01 | 1.712328767 | 1 | 8.678370698 | High groups |
| TCGA-86-7953-01 | 2.731506849 | 0 | 8.808894049 | High groups |
| TCGA-86-7954-01 | 1.657534247 | 0 | 7.934566804 | Low groups |
| TCGA-86-7955-01 | 2.246575342 | 1 | 7.010717972 | Low groups |
| TCGA-86-8054-01 | 3.145205479 | 0 | 9.679584251 | High groups |
| TCGA-86-8055-01 | 0.339726027 | 0 | 8.807326036 | High groups |
| TCGA-86-8056-01 | 0.380821918 | 0 | 8.915675953 | High groups |
| TCGA-86-8073-01 | 2.02739726 | 0 | 9.086946561 | High groups |
| TCGA-86-8074-01 | 0.065753425 | 0 | 9.473202443 | High groups |
| TCGA-86-8075-01 | 0.545205479 | 1 | 8.22415877 | High groups |
| TCGA-86-8076-01 | 2.720547945 | 0 | 8.143027841 | Low groups |
| TCGA-86-8278-01 | 0.079452055 | 1 | 8.087297717 | Low groups |
| TCGA-86-8279-01 | 2.6 | 0 | 9.195991726 | High groups |
| TCGA-86-8280-01 | 1.920547945 | 0 | 8.935197175 | High groups |
| TCGA-86-8281-01 | 0 | 0 | 9.008162049 | High groups |
| TCGA-86-8358-01 | 1.789041096 | 0 | 8.169951901 | High groups |
| TCGA-86-8359-01 | 1.216438356 | 0 | 7.42630728 | Low groups |
| TCGA-86-8585-01 | 0.967123288 | 0 | 7.662178648 | Low groups |
| TCGA-86-8668-01 | 1.15890411 | 0 | 8.222729708 | High groups |
| TCGA-86-8669-01 | 2.326027397 | 1 | 8.090513354 | Low groups |
| TCGA-86-8671-01 | 2.298630137 | 0 | 8.853066788 | High groups |
| TCGA-86-8672-01 | 0.052054795 | 0 | 7.287662242 | Low groups |
| TCGA-86-8673-01 | 1.742465753 | 1 | 8.521601146 | High groups |
| TCGA-86-8674-01 | 0.915068493 | 1 | 8.223577392 | High groups |
| TCGA-86-A456-01 | 2.454794521 | 0 | 7.497529659 | Low groups |
| TCGA-86-A4D0-01 | 0.317808219 | 0 | 7.074204522 | Low groups |
| TCGA-86-A4JF-01 | 1.194520548 | 1 | 7.944876362 | Low groups |
| TCGA-86-A4P7-01 | 1.136986301 | 0 | 7.707664689 | Low groups |
| TCGA-86-A4P8-01 | 2.205479452 | 0 | 8.64479443 | High groups |
| TCGA-91-6828-01 | 0.884931507 | 0 | 8.694119653 | High groups |
| TCGA-91-6829-01 | 3.446575342 | 0 | 8.521210172 | High groups |
| TCGA-91-6830-01 | 0.049315068 | 1 | 8.959803273 | High groups |
| TCGA-91-6831-01 | 0.849315068 | 0 | 7.870262248 | Low groups |
| TCGA-91-6835-01 | 0.216438356 | 0 | 7.732241586 | Low groups |
| TCGA-91-6836-01 | 1.142465753 | 0 | 9.006053645 | High groups |
| TCGA-91-6840-01 | 1.019178082 | 0 | 9.163842609 | High groups |
| TCGA-91-6847-01 | 2.115068493 | 1 | 9.501866039 | High groups |
| TCGA-91-6848-01 | 0.61369863 | 0 | 9.227595392 | High groups |
| TCGA-91-6849-01 | 0.095890411 | 0 | 9.264634009 | High groups |
| TCGA-91-7771-01 | 1.347945205 | 0 | 8.522474154 | High groups |
| TCGA-91-8496-01 | 1.383561644 | 0 | 7.86395641 | Low groups |
| TCGA-91-8497-01 | 1.189041096 | 0 | 8.540174158 | High groups |
| TCGA-91-8499-01 | 0.098630137 | 0 | 8.880527431 | High groups |
| TCGA-91-A4BC-01 | 0.120547945 | 0 | 6.863182472 | Low groups |
| TCGA-91-A4BD-01 | 1.652054795 | 0 | 7.822159138 | Low groups |
| TCGA-93-7347-01 | 1.871232877 | 0 | 8.40958942 | High groups |
| TCGA-93-7348-01 | 1.454794521 | 0 | 8.06526328 | Low groups |
| TCGA-93-8067-01 | 0.509589041 | 0 | 8.840288111 | High groups |
| TCGA-93-A4JN-01 | 1.967123288 | 0 | 7.812197782 | Low groups |
| TCGA-93-A4JO-01 | 0.090410959 | 0 | 7.780138218 | Low groups |
| TCGA-93-A4JP-01 | 1.361643836 | 1 | 8.6886112 | High groups |
| TCGA-93-A4JQ-01 | 1.44109589 | 0 | 7.674891656 | Low groups |
| TCGA-95-7039-01 | 3.446575342 | 1 | 8.059495348 | Low groups |
| TCGA-95-7043-01 | 1.378082192 | 1 | 8.423691066 | High groups |
| TCGA-95-7562-01 | 0.238356164 | 0 | 9.657865409 | High groups |
| TCGA-95-7567-01 | 1.556164384 | 0 | 7.662192751 | Low groups |
| TCGA-95-7944-01 | 1.032876712 | 0 | 8.50745521 | High groups |
| TCGA-95-7947-01 | 1.306849315 | 0 | 9.320227869 | High groups |
| TCGA-95-7948-01 | 1.304109589 | 0 | 7.157131046 | Low groups |
| TCGA-95-8039-01 | 0.624657534 | 1 | 7.921299334 | Low groups |
| TCGA-95-8494-01 | 0.230136986 | 0 | 7.767715306 | Low groups |
| TCGA-95-A4VK-01 | 1.35890411 | 1 | 7.527026377 | Low groups |
| TCGA-95-A4VN-01 | 1.515068493 | 0 | 8.148841951 | Low groups |
| TCGA-95-A4VP-01 | 0.591780822 | 1 | 7.903875822 | Low groups |
| TCGA-97-7546-01 | 3.438356164 | 1 | 8.432098376 | High groups |
| TCGA-97-7547-01 | 2.123287671 | 1 | 8.113469539 | Low groups |
| TCGA-97-7552-01 | 2.18630137 | 1 | 8.093571162 | Low groups |
| TCGA-97-7553-01 | 5.123287671 | 0 | 7.739101658 | Low groups |
| TCGA-97-7554-01 | 2.123287671 | 0 | 7.984282415 | Low groups |
| TCGA-97-7937-01 | 1.545205479 | 0 | 9.268389477 | High groups |
| TCGA-97-7938-01 | 0.049315068 | 0 | 8.273711086 | High groups |
| TCGA-97-7941-01 | 1.326027397 | 0 | 9.084751263 | High groups |
| TCGA-97-8171-01 | 1.208219178 | 1 | 10.09052333 | High groups |
| TCGA-97-8172-01 | 1.493150685 | 0 | 8.850233009 | High groups |
| TCGA-97-8174-01 | 0.449315068 | 0 | 8.407637846 | High groups |
| TCGA-97-8175-01 | 0.805479452 | 1 | 8.421300819 | High groups |
| TCGA-97-8176-01 | 0.106849315 | 1 | 7.704764782 | Low groups |
| TCGA-97-8177-01 | 1.367123288 | 0 | 7.901084343 | Low groups |
| TCGA-97-8179-01 | 1.191780822 | 0 | 8.450845858 | High groups |
| TCGA-97-8547-01 | 1.8 | 0 | 7.446070339 | Low groups |
| TCGA-97-8552-01 | 1.715068493 | 0 | 7.625570776 | Low groups |
| TCGA-97-A4LX-01 | 1.682191781 | 0 | 7.910706283 | Low groups |
| TCGA-97-A4M0-01 | 1.78630137 | 0 | 7.807859777 | Low groups |
| TCGA-97-A4M1-01 | 1.646575342 | 0 | 8.271634367 | High groups |
| TCGA-97-A4M2-01 | 1.709589041 | 0 | 8.649479661 | High groups |
| TCGA-97-A4M3-01 | 0.147945205 | 1 | 7.816266576 | Low groups |
| TCGA-97-A4M5-01 | 1.736986301 | 0 | 7.665567227 | Low groups |
| TCGA-97-A4M6-01 | 1.556164384 | 0 | 7.393616037 | Low groups |
| TCGA-97-A4M7-01 | 1.723287671 | 0 | 7.474697885 | Low groups |
| TCGA-99-7458-01 | 2.046575342 | 0 | 7.901900349 | Low groups |
| TCGA-99-8025-01 | 2.904109589 | 0 | 8.25076479 | High groups |
| TCGA-99-8028-01 | 3.063013699 | 0 | 7.627229701 | Low groups |
| TCGA-99-8032-01 | 0.120547945 | 0 | 8.893748773 | High groups |
| TCGA-99-8033-01 | 1.797260274 | 1 | 8.660226634 | High groups |
| TCGA-99-AA5R-01 | 1.802739726 | 0 | 8.500867886 | High groups |
| TCGA-J2-8192-01 | 1.320547945 | 1 | 8.531910871 | High groups |
| TCGA-J2-8194-01 | 1.287671233 | 1 | 8.622599939 | High groups |
| TCGA-J2-A4AD-01 | 1.435616438 | 1 | 8.017207397 | Low groups |
| TCGA-J2-A4AE-01 | 2.956164384 | 0 | 7.859689452 | Low groups |
| TCGA-J2-A4AG-01 | 2.706849315 | 0 | 7.573430502 | Low groups |
| TCGA-L4-A4E5-01 | 1.583561644 | 0 | 8.820694921 | High groups |
| TCGA-L4-A4E6-01 | 1.191780822 | 0 | 8.841586896 | High groups |
| TCGA-L9-A443-01 | 0.528767123 | 0 | 6.710021918 | Low groups |
| TCGA-L9-A444-01 | 0.84109589 | 0 | 7.695069569 | Low groups |
| TCGA-L9-A50W-01 | 1.065753425 | 1 | 8.819350675 | High groups |
| TCGA-L9-A5IP-01 | 0.134246575 | 1 | 8.446091601 | High groups |
| TCGA-L9-A743-01 | 1.819178082 | 0 | 7.649909931 | Low groups |
| TCGA-L9-A7SV-01 | 1.547945205 | 0 | 9.014490595 | High groups |
| TCGA-L9-A8F4-01 | 1.304109589 | 0 | 8.251123337 | High groups |
| TCGA-MN-A4N1-01 | 2.265753425 | 0 | 8.280338222 | High groups |
| TCGA-MN-A4N4-01 | 3.219178082 | 0 | 7.656944497 | Low groups |
| TCGA-MN-A4N5-01 | 0.230136986 | 0 | 8.008241368 | Low groups |
| TCGA-MP-A4SV-01 | 7.178082192 | 0 | 7.905356075 | Low groups |
| TCGA-MP-A4SW-01 | 4.871232877 | 0 | 7.491544006 | Low groups |
| TCGA-MP-A4SY-01 | 1.339726027 | 1 | 7.626227609 | Low groups |
| TCGA-MP-A4T2-01 | 1.575342466 | 1 | 8.027248619 | Low groups |
| TCGA-MP-A4T4-01 | 7.169863014 | 0 | 7.637819388 | Low groups |
| TCGA-MP-A4T6-01 | 4.904109589 | 0 | 7.685732823 | Low groups |
| TCGA-MP-A4T7-01 | 0.457534247 | 0 | 8.652953682 | High groups |
| TCGA-MP-A4T8-01 | 0.44109589 | 0 | 8.19317853 | High groups |
| TCGA-MP-A4T9-01 | 0.934246575 | 1 | 8.724734095 | High groups |
| TCGA-MP-A4TA-01 | 1.98630137 | 1 | 8.131985332 | Low groups |
| TCGA-MP-A4TC-01 | 0.202739726 | 0 | 8.794620039 | High groups |
| TCGA-MP-A4TD-01 | 0.619178082 | 1 | 7.934586795 | Low groups |
| TCGA-MP-A4TE-01 | 0.619178082 | 1 | 7.629081753 | Low groups |
| TCGA-MP-A4TF-01 | 0.534246575 | 1 | 8.178841698 | High groups |
| TCGA-MP-A4TH-01 | 2.030136986 | 0 | 8.233686538 | High groups |
| TCGA-MP-A4TI-01 | 0.230136986 | 1 | 7.398314196 | Low groups |
| TCGA-MP-A4TJ-01 | 0.928767123 | 0 | 8.12659153 | Low groups |
| TCGA-MP-A4TK-01 | 1.087671233 | 1 | 8.309670201 | High groups |
| TCGA-MP-A5C7-01 | 6.15890411 | 0 | 8.930160883 | High groups |
| TCGA-NJ-A4YF-01 | 5.920547945 | 0 | 7.368448927 | Low groups |
| TCGA-NJ-A4YG-01 | 6.194520548 | 0 | 7.598723988 | Low groups |
| TCGA-NJ-A4YI-01 | 0.010958904 | 0 | 7.966299293 | Low groups |
| TCGA-NJ-A4YP-01 | 0.136986301 | 0 | 7.951807779 | Low groups |
| TCGA-NJ-A4YQ-01 | 3.923287671 | 0 | 7.795853686 | Low groups |
| TCGA-NJ-A55A-01 | 0.04109589 | 0 | 9.499591201 | High groups |
| TCGA-NJ-A55O-01 | 0.035616438 | 0 | 8.230221894 | High groups |
| TCGA-NJ-A55R-01 | 1.652054795 | 0 | 7.882039314 | Low groups |
| TCGA-NJ-A7XG-01 | 1.690410959 | 0 | 7.165981862 | Low groups |
| TCGA-O1-A52J-01 | 2.460273973 | 1 | 8.568026973 | High groups |
| TCGA-S2-AA1A-01 | 1.405479452 | 0 | 7.387555118 | Low groups |

**Table S6.** Raw counts of RNA-sequencing data of hsa-miR-186-5p in LUAD from the TCGA.

| sampleID | time | Staus | RS | Label |
| --- | --- | --- | --- | --- |
| TCGA-05-4244-01 | 0 | 0 | 7.616980669 | Low groups |
| TCGA-05-4249-01 | 4.17260274 | 0 | 7.577003737 | Low groups |
| TCGA-05-4250-01 | 0.331506849 | 0 | 7.981964389 | Low groups |
| TCGA-05-4382-01 | 0.915068493 | 1 | 8.284767201 | High groups |
| TCGA-05-4384-01 | 0.501369863 | 1 | 8.297440436 | High groups |
| TCGA-05-4389-01 | 3.750684932 | 0 | 7.866375831 | Low groups |
| TCGA-05-4390-01 | 1.082191781 | 1 | 7.525798672 | Low groups |
| TCGA-05-4395-01 | 0 | 0 | 7.548974452 | Low groups |
| TCGA-05-4396-01 | 0.830136986 | 0 | 7.886574899 | Low groups |
| TCGA-05-4397-01 | 2.002739726 | 0 | 9.70424044 | High groups |
| TCGA-05-4398-01 | 3.920547945 | 0 | 8.215691214 | High groups |
| TCGA-05-4402-01 | 0.668493151 | 0 | 8.938464206 | High groups |
| TCGA-05-4403-01 | 1.583561644 | 0 | 8.357561813 | High groups |
| TCGA-05-4405-01 | 1.671232877 | 0 | 6.893408933 | Low groups |
| TCGA-05-4410-01 | 0 | 0 | 7.354239683 | Low groups |
| TCGA-05-4415-01 | 0.164383562 | 1 | 8.872826174 | High groups |
| TCGA-05-4417-01 | 1.246575342 | 0 | 7.055402619 | Low groups |
| TCGA-05-4418-01 | 0.750684932 | 0 | 9.410805463 | High groups |
| TCGA-05-4420-01 | 2.498630137 | 0 | 9.850193871 | High groups |
| TCGA-05-4422-01 | 1 | 0 | 8.639864145 | High groups |
| TCGA-05-4424-01 | 0.419178082 | 1 | 7.748090448 | Low groups |
| TCGA-05-4425-01 | 1.832876712 | 0 | 8.226644354 | High groups |
| TCGA-05-4426-01 | 1.252054795 | 1 | 7.716083851 | Low groups |
| TCGA-05-4427-01 | 2.167123288 | 0 | 7.157304083 | Low groups |
| TCGA-05-4430-01 | 2.084931507 | 0 | 8.188593988 | High groups |
| TCGA-05-4432-01 | 2.084931507 | 0 | 8.647463571 | High groups |
| TCGA-05-4433-01 | 2 | 0 | 7.690219983 | Low groups |
| TCGA-05-4434-01 | 1.252054795 | 0 | 8.829683924 | High groups |
| TCGA-05-5420-01 | 0.671232877 | 1 | 8.44178141 | High groups |
| TCGA-05-5423-01 | 0.41369863 | 0 | 8.460551731 | High groups |
| TCGA-05-5425-01 | 1.331506849 | 1 | 8.494406009 | High groups |
| TCGA-05-5428-01 | 1.835616438 | 0 | 8.130756235 | High groups |
| TCGA-05-5429-01 | 0.753424658 | 0 | 7.769389927 | Low groups |
| TCGA-05-5715-01 | 0.169863014 | 0 | 7.475227021 | Low groups |
| TCGA-35-3615-01 | 0.038356164 | 0 | 8.154954678 | High groups |
| TCGA-35-4122-01 | 0.616438356 | 0 | 8.368724268 | High groups |
| TCGA-35-4123-01 | 0.498630137 | 0 | 9.409951017 | High groups |
| TCGA-35-5375-01 | 0.723287671 | 0 | 7.318718769 | Low groups |
| TCGA-38-4625-01 | 8.145205479 | 0 | 8.553176468 | High groups |
| TCGA-38-4626-01 | 6.898630137 | 1 | 9.066125257 | High groups |
| TCGA-38-4627-01 | 3.142465753 | 0 | 7.908225853 | Low groups |
| TCGA-38-4628-01 | 2.967123288 | 1 | 8.48094373 | High groups |
| TCGA-38-4629-01 | 1.038356164 | 1 | 7.669074259 | Low groups |
| TCGA-38-4630-01 | 1.435616438 | 1 | 9.14424156 | High groups |
| TCGA-38-4631-01 | 0.969863014 | 1 | 8.189278801 | High groups |
| TCGA-38-4632-01 | 1.863013699 | 1 | 8.282001175 | High groups |
| TCGA-38-6178-01 | 1.22739726 | 0 | 7.632614232 | Low groups |
| TCGA-38-7271-01 | 0.832876712 | 1 | 7.591469163 | Low groups |
| TCGA-38-A44F-01 | 0.364383562 | 0 | 7.835112456 | Low groups |
| TCGA-44-2655-01 | 2.764383562 | 1 | 8.859173151 | High groups |
| TCGA-44-2656-01 | 1.556164384 | 1 | 8.585035009 | High groups |
| TCGA-44-2657-01 | 3.701369863 | 0 | 8.236469275 | High groups |
| TCGA-44-2659-01 | 3.139726027 | 1 | 8.582784417 | High groups |
| TCGA-44-2661-01 | 3.175342466 | 0 | 7.358940897 | Low groups |
| TCGA-44-2662-01 | 0.671232877 | 1 | 8.94093019 | High groups |
| TCGA-44-2665-01 | 3.564383562 | 0 | 8.063539721 | Low groups |
| TCGA-44-2666-01 | 0.265753425 | 1 | 7.816966541 | Low groups |
| TCGA-44-2668-01 | 1.18630137 | 1 | 8.449635818 | High groups |
| TCGA-44-3396-01 | 3.095890411 | 0 | 8.887860317 | High groups |
| TCGA-44-3398-01 | 3.18630137 | 0 | 8.39374211 | High groups |
| TCGA-44-3918-01 | 1.4 | 1 | 7.644886844 | Low groups |
| TCGA-44-3919-01 | 2.523287671 | 1 | 8.97149234 | High groups |
| TCGA-44-4112-01 | 1.695890411 | 1 | 7.829715759 | Low groups |
| TCGA-44-5643-01 | 2.775342466 | 0 | 8.109701237 | High groups |
| TCGA-44-5644-01 | 2.364383562 | 0 | 7.377407418 | Low groups |
| TCGA-44-5645-01 | 2.334246575 | 0 | 8.26333118 | High groups |
| TCGA-44-6144-01 | 1.093150685 | 1 | 8.023673139 | Low groups |
| TCGA-44-6145-01 | 1.630136986 | 0 | 8.33446441 | High groups |
| TCGA-44-6146-01 | 1.728767123 | 1 | 8.520181858 | High groups |
| TCGA-44-6147-01 | 2.315068493 | 0 | 7.850058912 | Low groups |
| TCGA-44-6148-01 | 1.928767123 | 0 | 8.356940489 | High groups |
| TCGA-44-6774-01 | 1.802739726 | 0 | 7.171314429 | Low groups |
| TCGA-44-6775-01 | 1.873972603 | 1 | 8.780710847 | High groups |
| TCGA-44-6776-01 | 7.167123288 | 0 | 7.836814118 | Low groups |
| TCGA-44-6777-01 | 2.704109589 | 0 | 7.08638604 | Low groups |
| TCGA-44-6778-01 | 5.106849315 | 0 | 7.756637245 | Low groups |
| TCGA-44-6779-01 | 0.635616438 | 1 | 7.529150968 | Low groups |
| TCGA-44-7659-01 | 1.893150685 | 0 | 8.047556928 | Low groups |
| TCGA-44-7660-01 | 0.693150685 | 1 | 8.751123742 | High groups |
| TCGA-44-7661-01 | 0.917808219 | 1 | 7.178971332 | Low groups |
| TCGA-44-7662-01 | 0.597260274 | 0 | 7.130242605 | Low groups |
| TCGA-44-7667-01 | 3.005479452 | 0 | 7.746263986 | Low groups |
| TCGA-44-7669-01 | 1.068493151 | 1 | 8.992678373 | High groups |
| TCGA-44-7670-01 | 2.416438356 | 0 | 7.695172532 | Low groups |
| TCGA-44-7671-01 | 2.419178082 | 1 | 6.952244211 | Low groups |
| TCGA-44-7672-01 | 1.969863014 | 0 | 7.25963048 | Low groups |
| TCGA-44-8117-01 | 1.054794521 | 0 | 8.594868657 | High groups |
| TCGA-44-8119-01 | 0.780821918 | 0 | 7.491935408 | Low groups |
| TCGA-44-8120-01 | 0.712328767 | 0 | 7.6484223 | Low groups |
| TCGA-44-A479-01 | 1.197260274 | 1 | 8.813853484 | High groups |
| TCGA-44-A47A-01 | 1.087671233 | 1 | 9.45953744 | High groups |
| TCGA-44-A47B-01 | 0.78630137 | 0 | 8.04345661 | Low groups |
| TCGA-44-A47F-01 | 0.923287671 | 0 | 8.185342185 | High groups |
| TCGA-44-A47G-01 | 0.961643836 | 0 | 8.074958241 | Low groups |
| TCGA-44-A4SS-01 | 1.136986301 | 0 | 8.347254348 | High groups |
| TCGA-44-A4SU-01 | 0.712328767 | 1 | 8.549396946 | High groups |
| TCGA-49-4486-01 | 5.602739726 | 1 | 9.006719515 | High groups |
| TCGA-49-4487-01 | 1.909589041 | 1 | 7.912506582 | Low groups |
| TCGA-49-4488-01 | 1.736986301 | 1 | 8.1535344 | High groups |
| TCGA-49-4490-01 | 1.054794521 | 1 | 7.658555475 | Low groups |
| TCGA-49-4494-01 | 2.961643836 | 1 | 8.08292927 | Low groups |
| TCGA-49-4501-01 | 1.493150685 | 1 | 8.076298432 | Low groups |
| TCGA-49-4505-01 | 1.142465753 | 1 | 8.149179785 | High groups |
| TCGA-49-4506-01 | 1.895890411 | 1 | 8.789055178 | High groups |
| TCGA-49-4507-01 | 0.432876712 | 1 | 8.096226532 | High groups |
| TCGA-49-4510-01 | 1.375342466 | 1 | 8.691462629 | High groups |
| TCGA-49-4512-01 | 2.479452055 | 1 | 6.990645306 | Low groups |
| TCGA-49-4514-01 | 4.657534247 | 0 | 8.222860577 | High groups |
| TCGA-49-6742-01 | 0.58630137 | 1 | 7.729633182 | Low groups |
| TCGA-49-6743-01 | 4.44109589 | 0 | 7.691974541 | Low groups |
| TCGA-49-6744-01 | 4.610958904 | 0 | 7.114278936 | Low groups |
| TCGA-49-6745-01 | 1.430136986 | 0 | 6.851302974 | Low groups |
| TCGA-49-6761-01 | 0.969863014 | 0 | 7.665541718 | Low groups |
| TCGA-49-6767-01 | 1.854794521 | 0 | 7.472546339 | Low groups |
| TCGA-49-AAQV-01 | 1.394520548 | 1 | 7.806771414 | Low groups |
| TCGA-49-AAR0-01 | 13.05479452 | 0 | 9.025226952 | High groups |
| TCGA-49-AAR2-01 | 6.093150685 | 0 | 9.6445359 | High groups |
| TCGA-49-AAR3-01 | 5.18630137 | 1 | 7.927281258 | Low groups |
| TCGA-49-AAR4-01 | 2.408219178 | 1 | 8.081359868 | Low groups |
| TCGA-49-AAR9-01 | 0.712328767 | 1 | 8.358564263 | High groups |
| TCGA-49-AARE-01 | 1.063013699 | 1 | 8.141116492 | High groups |
| TCGA-49-AARN-01 | 3.109589041 | 0 | 8.154807276 | High groups |
| TCGA-49-AARO-01 | 3.134246575 | 1 | 7.498308957 | Low groups |
| TCGA-49-AARQ-01 | 18.44383562 | 0 | 8.614790699 | High groups |
| TCGA-49-AARR-01 | 3.010958904 | 1 | 8.116804926 | High groups |
| TCGA-4B-A93V-01 | 0.687671233 | 1 | 8.988244001 | High groups |
| TCGA-50-5044-01 | 1.323287671 | 1 | 7.291024511 | Low groups |
| TCGA-50-5045-01 | 3.926027397 | 1 | 7.533974576 | Low groups |
| TCGA-50-5049-01 | 4.295890411 | 1 | 8.65503463 | High groups |
| TCGA-50-5051-01 | 0.504109589 | 1 | 6.834795517 | Low groups |
| TCGA-50-5055-01 | 2.112328767 | 1 | 8.518274957 | High groups |
| TCGA-50-5066-01 | 1.306849315 | 1 | 8.191336965 | High groups |
| TCGA-50-5068-01 | 1.879452055 | 1 | 8.374819273 | High groups |
| TCGA-50-5072-01 | 0.583561644 | 1 | 7.53563239 | Low groups |
| TCGA-50-5930-01 | 0.484931507 | 1 | 8.263662771 | High groups |
| TCGA-50-5931-01 | 1.164383562 | 1 | 9.37223475 | High groups |
| TCGA-50-5932-01 | 2.98630137 | 1 | 8.385476423 | High groups |
| TCGA-50-5933-01 | 6.556164384 | 0 | 7.239325955 | Low groups |
| TCGA-50-5935-01 | 1.789041096 | 0 | 7.871054212 | Low groups |
| TCGA-50-5936-01 | 0.410958904 | 1 | 7.586991591 | Low groups |
| TCGA-50-5939-01 | 1.260273973 | 0 | 7.865375413 | Low groups |
| TCGA-50-5941-01 | 4.038356164 | 0 | 7.976879005 | Low groups |
| TCGA-50-5942-01 | 3.775342466 | 1 | 8.559168884 | High groups |
| TCGA-50-5944-01 | 4.794520548 | 0 | 7.758204556 | Low groups |
| TCGA-50-5946-01 | 0.605479452 | 1 | 7.920707039 | Low groups |
| TCGA-50-6590-01 | 3.528767123 | 0 | 7.398200025 | Low groups |
| TCGA-50-6591-01 | 0.326027397 | 1 | 7.499394436 | Low groups |
| TCGA-50-6592-01 | 2.128767123 | 1 | 8.300546813 | High groups |
| TCGA-50-6593-01 | 0.728767123 | 1 | 7.549134837 | Low groups |
| TCGA-50-6594-01 | 0.780821918 | 1 | 8.543736441 | High groups |
| TCGA-50-6595-01 | 0.498630137 | 1 | 7.111362101 | Low groups |
| TCGA-50-6597-01 | 3.473972603 | 0 | 7.515552487 | Low groups |
| TCGA-50-6673-01 | 0.060273973 | 0 | 8.192959999 | High groups |
| TCGA-50-7109-01 | 0.04109589 | 1 | 7.47810342 | Low groups |
| TCGA-50-8457-01 | 3.082191781 | 0 | 7.978919999 | Low groups |
| TCGA-50-8459-01 | 1.183561644 | 1 | 7.28267554 | Low groups |
| TCGA-50-8460-01 | 2.271232877 | 0 | 7.743974556 | Low groups |
| TCGA-53-7624-01 | 1.095890411 | 1 | 8.027142825 | Low groups |
| TCGA-53-7626-01 | 2.369863014 | 1 | 7.156283143 | Low groups |
| TCGA-53-7813-01 | 1.161643836 | 0 | 7.425911335 | Low groups |
| TCGA-53-A4EZ-01 | 2.934246575 | 0 | 8.278256343 | High groups |
| TCGA-55-1592-01 | 1.238356164 | 1 | 10.1841713 | High groups |
| TCGA-55-1594-01 | 3.22739726 | 0 | 10.42483624 | High groups |
| TCGA-55-1595-01 | 4.052054795 | 0 | 9.271437956 | High groups |
| TCGA-55-1596-01 | 5.657534247 | 0 | 9.671877597 | High groups |
| TCGA-55-5899-01 | 2.547945205 | 0 | 6.976649465 | Low groups |
| TCGA-55-6543-01 | 1.191780822 | 0 | 8.050744756 | Low groups |
| TCGA-55-6642-01 | 6.709589041 | 0 | 7.289307464 | Low groups |
| TCGA-55-6712-01 | 0.468493151 | 1 | 7.463695198 | Low groups |
| TCGA-55-6968-01 | 3.542465753 | 1 | 7.726173192 | Low groups |
| TCGA-55-6969-01 | 3.394520548 | 0 | 8.85431541 | High groups |
| TCGA-55-6970-01 | 1.252054795 | 1 | 8.315873853 | High groups |
| TCGA-55-6971-01 | 3.835616438 | 0 | 8.160626788 | High groups |
| TCGA-55-6972-01 | 4.471232877 | 0 | 8.026905824 | Low groups |
| TCGA-55-6978-01 | 0.115068493 | 1 | 7.708135493 | Low groups |
| TCGA-55-6979-01 | 0.534246575 | 1 | 8.434968305 | High groups |
| TCGA-55-6980-01 | 5.778082192 | 0 | 9.224755876 | High groups |
| TCGA-55-6981-01 | 3.778082192 | 0 | 7.91046477 | Low groups |
| TCGA-55-6982-01 | 0.501369863 | 1 | 8.246382243 | High groups |
| TCGA-55-6983-01 | 7.734246575 | 0 | 8.213931921 | High groups |
| TCGA-55-6984-01 | 1.983561644 | 1 | 8.180116027 | High groups |
| TCGA-55-6985-01 | 3.378082192 | 0 | 7.906810764 | Low groups |
| TCGA-55-6986-01 | 8.934246575 | 0 | 9.047846987 | High groups |
| TCGA-55-6987-01 | 5.854794521 | 0 | 8.857011825 | High groups |
| TCGA-55-7227-01 | 0.698630137 | 1 | 7.646066436 | Low groups |
| TCGA-55-7281-01 | 0.928767123 | 1 | 7.297704267 | Low groups |
| TCGA-55-7283-01 | 1.668493151 | 0 | 7.137887201 | Low groups |
| TCGA-55-7284-01 | 0.632876712 | 1 | 7.525564265 | Low groups |
| TCGA-55-7570-01 | 2.257534247 | 0 | 8.304483206 | High groups |
| TCGA-55-7573-01 | 1.334246575 | 0 | 8.370718788 | High groups |
| TCGA-55-7574-01 | 1.309589041 | 1 | 7.541340749 | Low groups |
| TCGA-55-7576-01 | 1.835616438 | 0 | 7.728433541 | Low groups |
| TCGA-55-7724-01 | 1.931506849 | 0 | 7.378845988 | Low groups |
| TCGA-55-7725-01 | 1.210958904 | 0 | 8.174708263 | High groups |
| TCGA-55-7726-01 | 1.78630137 | 0 | 6.401152071 | Low groups |
| TCGA-55-7727-01 | 0.326027397 | 0 | 8.157493977 | High groups |
| TCGA-55-7728-01 | 1.928767123 | 0 | 7.705063007 | Low groups |
| TCGA-55-7815-01 | 1.276712329 | 1 | 6.955222865 | Low groups |
| TCGA-55-7816-01 | 1.282191781 | 1 | 7.466749215 | Low groups |
| TCGA-55-7903-01 | 1.553424658 | 0 | 7.177809287 | Low groups |
| TCGA-55-7907-01 | 0.805479452 | 1 | 8.071245298 | Low groups |
| TCGA-55-7910-01 | 2.789041096 | 1 | 7.894732108 | Low groups |
| TCGA-55-7911-01 | 1.410958904 | 1 | 7.428587971 | Low groups |
| TCGA-55-7913-01 | 1.315068493 | 1 | 7.998181171 | Low groups |
| TCGA-55-7914-01 | 0.512328767 | 1 | 7.951667634 | Low groups |
| TCGA-55-7994-01 | 1.652054795 | 0 | 8.49170914 | High groups |
| TCGA-55-7995-01 | 1.282191781 | 1 | 8.195505581 | High groups |
| TCGA-55-8085-01 | 2.476712329 | 0 | 8.698617339 | High groups |
| TCGA-55-8087-01 | 1.265753425 | 0 | 7.94361253 | Low groups |
| TCGA-55-8089-01 | 1.923287671 | 0 | 9.102273211 | High groups |
| TCGA-55-8090-01 | 1.501369863 | 1 | 7.429951829 | Low groups |
| TCGA-55-8091-01 | 1.643835616 | 0 | 7.248054693 | Low groups |
| TCGA-55-8092-01 | 0.347945205 | 1 | 8.546952861 | High groups |
| TCGA-55-8094-01 | 1.482191781 | 0 | 8.684901191 | High groups |
| TCGA-55-8096-01 | 1.550684932 | 1 | 8.920798608 | High groups |
| TCGA-55-8097-01 | 1.304109589 | 0 | 7.471714797 | Low groups |
| TCGA-55-8203-01 | 1.498630137 | 0 | 7.913380487 | Low groups |
| TCGA-55-8204-01 | 1.410958904 | 0 | 7.492393765 | Low groups |
| TCGA-55-8205-01 | 1.356164384 | 1 | 7.542184752 | Low groups |
| TCGA-55-8206-01 | 2.432876712 | 0 | 7.991689574 | Low groups |
| TCGA-55-8207-01 | 2.676712329 | 0 | 7.545369644 | Low groups |
| TCGA-55-8208-01 | 1.4 | 1 | 8.461971844 | High groups |
| TCGA-55-8299-01 | 0.750684932 | 1 | 8.05657242 | Low groups |
| TCGA-55-8301-01 | 0.652054795 | 1 | 8.445755998 | High groups |
| TCGA-55-8302-01 | 1.309589041 | 0 | 7.331683901 | Low groups |
| TCGA-55-8505-01 | 1.205479452 | 0 | 8.504498044 | High groups |
| TCGA-55-8506-01 | 0.030136986 | 0 | 7.305415637 | Low groups |
| TCGA-55-8507-01 | 1.145205479 | 0 | 8.754915888 | High groups |
| TCGA-55-8508-01 | 1.690410959 | 0 | 8.075332386 | Low groups |
| TCGA-55-8510-01 | 1.476712329 | 0 | 8.259896515 | High groups |
| TCGA-55-8511-01 | 1.260273973 | 1 | 8.688492906 | High groups |
| TCGA-55-8512-01 | 1.663013699 | 1 | 8.46873302 | High groups |
| TCGA-55-8513-01 | 0.868493151 | 1 | 8.761885773 | High groups |
| TCGA-55-8514-01 | 1.424657534 | 0 | 8.946487964 | High groups |
| TCGA-55-8614-01 | 1.468493151 | 0 | 7.868973513 | Low groups |
| TCGA-55-8615-01 | 0.61369863 | 1 | 7.833920351 | Low groups |
| TCGA-55-8616-01 | 0.131506849 | 0 | 8.610645957 | High groups |
| TCGA-55-8619-01 | 1.139726027 | 0 | 8.205261028 | High groups |
| TCGA-55-8620-01 | 1.02739726 | 1 | 7.024791834 | Low groups |
| TCGA-55-8621-01 | 1.410958904 | 0 | 7.760945373 | Low groups |
| TCGA-55-A48X-01 | 1.684931507 | 1 | 8.240765864 | High groups |
| TCGA-55-A48Y-01 | 1.726027397 | 0 | 7.889491248 | Low groups |
| TCGA-55-A48Z-01 | 1.468493151 | 1 | 8.041943994 | Low groups |
| TCGA-55-A490-01 | 0.271232877 | 0 | 7.80833826 | Low groups |
| TCGA-55-A491-01 | 1.715068493 | 0 | 7.916772796 | Low groups |
| TCGA-55-A492-01 | 1.632876712 | 0 | 8.028740252 | Low groups |
| TCGA-55-A493-01 | 0.076712329 | 0 | 7.916601621 | Low groups |
| TCGA-55-A494-01 | 1.317808219 | 0 | 8.28114035 | High groups |
| TCGA-55-A4DF-01 | 1.410958904 | 1 | 8.348528553 | High groups |
| TCGA-55-A4DG-01 | 1.665753425 | 0 | 8.434481616 | High groups |
| TCGA-55-A57B-01 | 1.495890411 | 0 | 8.544081357 | High groups |
| TCGA-62-8394-01 | 0.380821918 | 0 | 9.24385922 | High groups |
| TCGA-62-8395-01 | 1.082191781 | 1 | 8.202388726 | High groups |
| TCGA-62-8397-01 | 3.531506849 | 0 | 8.615528709 | High groups |
| TCGA-62-8398-01 | 1.216438356 | 0 | 8.312823522 | High groups |
| TCGA-62-8399-01 | 7.38630137 | 0 | 9.995714162 | High groups |
| TCGA-62-8402-01 | 2.115068493 | 1 | 8.886728827 | High groups |
| TCGA-62-A46O-01 | 2.583561644 | 1 | 8.750637487 | High groups |
| TCGA-62-A46P-01 | 0.731506849 | 1 | 8.432533967 | High groups |
| TCGA-62-A46R-01 | 4.726027397 | 0 | 7.766587543 | Low groups |
| TCGA-62-A46S-01 | 1.443835616 | 1 | 8.970096311 | High groups |
| TCGA-62-A46U-01 | 5.663013699 | 0 | 8.008534789 | Low groups |
| TCGA-62-A46V-01 | 6.024657534 | 0 | 8.469962471 | High groups |
| TCGA-62-A46Y-01 | 0.860273973 | 1 | 9.306426078 | High groups |
| TCGA-62-A470-01 | 1.476712329 | 1 | 8.70665083 | High groups |
| TCGA-62-A471-01 | 3.41369863 | 0 | 8.971428594 | High groups |
| TCGA-62-A472-01 | 0.794520548 | 1 | 7.409546739 | Low groups |
| TCGA-64-1676-01 | 4.734246575 | 0 | 8.586298179 | High groups |
| TCGA-64-1677-01 | 0.98630137 | 1 | 8.947334488 | High groups |
| TCGA-64-1678-01 | 3.257534247 | 0 | 8.358005854 | High groups |
| TCGA-64-1679-01 | 6.816438356 | 0 | 7.301562589 | Low groups |
| TCGA-64-1680-01 | 3.084931507 | 0 | 7.060997407 | Low groups |
| TCGA-64-1681-01 | 1.202739726 | 1 | 8.04075109 | Low groups |
| TCGA-64-5774-01 | 0.673972603 | 1 | 7.593753908 | Low groups |
| TCGA-64-5775-01 | 0.169863014 | 1 | 9.790487139 | High groups |
| TCGA-64-5778-01 | 2.635616438 | 1 | 8.627855108 | High groups |
| TCGA-64-5779-01 | 2.178082192 | 1 | 8.635957854 | High groups |
| TCGA-64-5781-01 | 0.263013699 | 1 | 8.274775087 | High groups |
| TCGA-64-5815-01 | 2.37260274 | 0 | 7.000785942 | Low groups |
| TCGA-67-3770-01 | 1.671232877 | 0 | 8.48536725 | High groups |
| TCGA-67-3771-01 | 1.671232877 | 0 | 9.185975908 | High groups |
| TCGA-67-3772-01 | 1.569863014 | 0 | 7.789115806 | Low groups |
| TCGA-67-3773-01 | 1.169863014 | 0 | 7.828992198 | Low groups |
| TCGA-67-3774-01 | 1.054794521 | 0 | 9.85461176 | High groups |
| TCGA-67-4679-01 | 1.22739726 | 0 | 7.537971357 | Low groups |
| TCGA-67-6215-01 | 0.476712329 | 0 | 7.748568237 | Low groups |
| TCGA-67-6216-01 | 0.38630137 | 0 | 7.856216035 | Low groups |
| TCGA-67-6217-01 | 0.810958904 | 1 | 9.244108338 | High groups |
| TCGA-69-7760-01 | 0.553424658 | 0 | 6.431475373 | Low groups |
| TCGA-69-7761-01 | 0.509589041 | 0 | 7.629378625 | Low groups |
| TCGA-69-7763-01 | 1.890410959 | 0 | 7.412916475 | Low groups |
| TCGA-69-7764-01 | 1.134246575 | 0 | 7.438655726 | Low groups |
| TCGA-69-7765-01 | 0.452054795 | 0 | 7.854335204 | Low groups |
| TCGA-69-7973-01 | 0.630136986 | 0 | 6.929271596 | Low groups |
| TCGA-69-7974-01 | 0.504109589 | 0 | 7.699911581 | Low groups |
| TCGA-69-7978-01 | 0.367123288 | 0 | 7.768080288 | Low groups |
| TCGA-69-7979-01 | 1.117808219 | 0 | 7.915448776 | Low groups |
| TCGA-69-7980-01 | 1.126027397 | 0 | 8.054759825 | Low groups |
| TCGA-69-8253-01 | 1.167123288 | 0 | 8.378326072 | High groups |
| TCGA-69-8254-01 | 1.120547945 | 0 | 8.621209758 | High groups |
| TCGA-69-8255-01 | 0.353424658 | 0 | 8.369355241 | High groups |
| TCGA-69-8453-01 | 0.942465753 | 1 | 8.244101771 | High groups |
| TCGA-69-A59K-01 | 1.619178082 | 0 | 8.083551872 | Low groups |
| TCGA-71-6725-01 | 0.449315068 | 1 | 7.62656909 | Low groups |
| TCGA-71-8520-01 | 0.490410959 | 1 | 8.109380515 | High groups |
| TCGA-73-4658-01 | 4.383561644 | 0 | 7.316312852 | Low groups |
| TCGA-73-4659-01 | 0.095890411 | 1 | 8.613799481 | High groups |
| TCGA-73-4662-01 | 0.057534247 | 1 | 9.357869539 | High groups |
| TCGA-73-4675-01 | 0.989041096 | 1 | 7.550502084 | Low groups |
| TCGA-73-4676-01 | 0.769863014 | 1 | 7.913819186 | Low groups |
| TCGA-73-7498-01 | 3.257534247 | 0 | 8.007755581 | Low groups |
| TCGA-73-7499-01 | 3.964383562 | 1 | 7.641161751 | Low groups |
| TCGA-73-A9RS-01 | 0.293150685 | 1 | 8.276282907 | High groups |
| TCGA-75-5125-01 | 4.8 | 1 | 8.17666595 | High groups |
| TCGA-75-5146-01 | 4.857534247 | 1 | 8.773431409 | High groups |
| TCGA-75-5147-01 | 3.652054795 | 0 | 8.815353961 | High groups |
| TCGA-75-6206-01 | 7.095890411 | 0 | 8.144941503 | High groups |
| TCGA-75-6212-01 | 3.671232877 | 1 | 8.241816354 | High groups |
| TCGA-75-6214-01 | 1.147945205 | 1 | 8.297969059 | High groups |
| TCGA-75-7025-01 | 4.057534247 | 1 | 8.372175317 | High groups |
| TCGA-75-7027-01 | 8.079452055 | 1 | 7.588810262 | Low groups |
| TCGA-78-7143-01 | 4.109589041 | 1 | 7.369210831 | Low groups |
| TCGA-78-7145-01 | 1.156164384 | 1 | 7.599859595 | Low groups |
| TCGA-78-7146-01 | 0.473972603 | 0 | 7.021108901 | Low groups |
| TCGA-78-7147-01 | 1.583561644 | 1 | 8.206679654 | High groups |
| TCGA-78-7148-01 | 0.498630137 | 1 | 7.587859782 | Low groups |
| TCGA-78-7149-01 | 10.79452055 | 0 | 8.512251226 | High groups |
| TCGA-78-7150-01 | 0.380821918 | 1 | 7.667067888 | Low groups |
| TCGA-78-7152-01 | 3.293150685 | 1 | 7.553375711 | Low groups |
| TCGA-78-7153-01 | 9.95890411 | 0 | 7.190017531 | Low groups |
| TCGA-78-7154-01 | 1.624657534 | 0 | 6.89437822 | Low groups |
| TCGA-78-7155-01 | 0.57260274 | 1 | 8.053497705 | Low groups |
| TCGA-78-7156-01 | 2.673972603 | 0 | 8.404050092 | High groups |
| TCGA-78-7158-01 | 0.361643836 | 1 | 8.014801602 | Low groups |
| TCGA-78-7159-01 | 5.408219178 | 0 | 7.772908357 | Low groups |
| TCGA-78-7160-01 | 1.909589041 | 0 | 7.719850615 | Low groups |
| TCGA-78-7161-01 | 0.443835616 | 1 | 8.668356656 | High groups |
| TCGA-78-7162-01 | 6.076712329 | 1 | 7.938866212 | Low groups |
| TCGA-78-7163-01 | 19.85753425 | 0 | 6.804799218 | Low groups |
| TCGA-78-7166-01 | 0.706849315 | 0 | 7.986473284 | Low groups |
| TCGA-78-7167-01 | 1.989041096 | 1 | 8.244448753 | High groups |
| TCGA-78-7220-01 | 1.454794521 | 1 | 7.907044054 | Low groups |
| TCGA-78-7535-01 | 2.216438356 | 1 | 8.174412811 | High groups |
| TCGA-78-7536-01 | 0.632876712 | 1 | 8.810108548 | High groups |
| TCGA-78-7537-01 | 4.443835616 | 0 | 7.814670643 | Low groups |
| TCGA-78-7539-01 | 1.884931507 | 1 | 7.364475257 | Low groups |
| TCGA-78-7540-01 | 3.279452055 | 0 | 7.521623209 | Low groups |
| TCGA-78-7542-01 | 0.879452055 | 0 | 8.089790226 | High groups |
| TCGA-78-7633-01 | 3.97260274 | 1 | 8.263264035 | High groups |
| TCGA-78-8640-01 | 19.34794521 | 0 | 8.605064392 | High groups |
| TCGA-78-8648-01 | 1.97260274 | 1 | 8.215451702 | High groups |
| TCGA-78-8655-01 | 6.465753425 | 0 | 7.556488275 | Low groups |
| TCGA-78-8660-01 | 0.663013699 | 1 | 8.239044877 | High groups |
| TCGA-78-8662-01 | 8.339726027 | 1 | 8.637129947 | High groups |
| TCGA-80-5608-01 | 7.75890411 | 0 | 7.94171435 | Low groups |
| TCGA-80-5611-01 | 7.109589041 | 0 | 7.711937284 | Low groups |
| TCGA-83-5908-01 | 2.257534247 | 0 | 8.315478242 | High groups |
| TCGA-86-6562-01 | 0.750684932 | 1 | 7.110979026 | Low groups |
| TCGA-86-6851-01 | 0.490410959 | 0 | 8.677942811 | High groups |
| TCGA-86-7701-01 | 1.161643836 | 1 | 7.265098537 | Low groups |
| TCGA-86-7711-01 | 2.865753425 | 1 | 8.161154966 | High groups |
| TCGA-86-7713-01 | 3.169863014 | 0 | 8.296589694 | High groups |
| TCGA-86-7714-01 | 1.712328767 | 1 | 7.939409195 | Low groups |
| TCGA-86-7953-01 | 2.731506849 | 0 | 8.543854541 | High groups |
| TCGA-86-7954-01 | 1.657534247 | 0 | 7.796446946 | Low groups |
| TCGA-86-7955-01 | 2.246575342 | 1 | 9.221908635 | High groups |
| TCGA-86-8054-01 | 3.145205479 | 0 | 7.727285529 | Low groups |
| TCGA-86-8055-01 | 0.339726027 | 0 | 8.048664945 | Low groups |
| TCGA-86-8056-01 | 0.380821918 | 0 | 7.905995907 | Low groups |
| TCGA-86-8073-01 | 2.02739726 | 0 | 8.220331406 | High groups |
| TCGA-86-8074-01 | 0.065753425 | 0 | 8.134962533 | High groups |
| TCGA-86-8075-01 | 0.545205479 | 1 | 8.125778425 | High groups |
| TCGA-86-8076-01 | 2.720547945 | 0 | 8.121378879 | High groups |
| TCGA-86-8278-01 | 0.079452055 | 1 | 6.988892443 | Low groups |
| TCGA-86-8279-01 | 2.6 | 0 | 8.249982364 | High groups |
| TCGA-86-8280-01 | 1.920547945 | 0 | 8.728907206 | High groups |
| TCGA-86-8281-01 | 0 | 0 | 8.206698406 | High groups |
| TCGA-86-8358-01 | 1.789041096 | 0 | 7.609555378 | Low groups |
| TCGA-86-8359-01 | 1.216438356 | 0 | 7.750394801 | Low groups |
| TCGA-86-8585-01 | 0.967123288 | 0 | 7.855169043 | Low groups |
| TCGA-86-8668-01 | 1.15890411 | 0 | 7.610953509 | Low groups |
| TCGA-86-8669-01 | 2.326027397 | 1 | 8.379157137 | High groups |
| TCGA-86-8671-01 | 2.298630137 | 0 | 8.86675804 | High groups |
| TCGA-86-8672-01 | 0.052054795 | 0 | 7.493327979 | Low groups |
| TCGA-86-8673-01 | 1.742465753 | 1 | 7.985492632 | Low groups |
| TCGA-86-8674-01 | 0.915068493 | 1 | 8.518659764 | High groups |
| TCGA-86-A456-01 | 2.454794521 | 0 | 8.187503264 | High groups |
| TCGA-86-A4D0-01 | 0.317808219 | 0 | 9.309213348 | High groups |
| TCGA-86-A4JF-01 | 1.194520548 | 1 | 8.082280954 | Low groups |
| TCGA-86-A4P7-01 | 1.136986301 | 0 | 8.418696793 | High groups |
| TCGA-86-A4P8-01 | 2.205479452 | 0 | 8.801478867 | High groups |
| TCGA-91-6828-01 | 0.884931507 | 0 | 7.145361067 | Low groups |
| TCGA-91-6829-01 | 3.446575342 | 0 | 7.019560151 | Low groups |
| TCGA-91-6830-01 | 0.049315068 | 1 | 7.800922025 | Low groups |
| TCGA-91-6831-01 | 0.849315068 | 0 | 7.951053982 | Low groups |
| TCGA-91-6835-01 | 0.216438356 | 0 | 7.436726031 | Low groups |
| TCGA-91-6836-01 | 1.142465753 | 0 | 8.273206001 | High groups |
| TCGA-91-6840-01 | 1.019178082 | 0 | 8.458210713 | High groups |
| TCGA-91-6847-01 | 2.115068493 | 1 | 8.609311659 | High groups |
| TCGA-91-6848-01 | 0.61369863 | 0 | 8.551092991 | High groups |
| TCGA-91-6849-01 | 0.095890411 | 0 | 8.569793343 | High groups |
| TCGA-91-7771-01 | 1.347945205 | 0 | 7.588080385 | Low groups |
| TCGA-91-8496-01 | 1.383561644 | 0 | 8.25946929 | High groups |
| TCGA-91-8497-01 | 1.189041096 | 0 | 7.834878739 | Low groups |
| TCGA-91-8499-01 | 0.098630137 | 0 | 8.245998851 | High groups |
| TCGA-91-A4BC-01 | 0.120547945 | 0 | 7.884570022 | Low groups |
| TCGA-91-A4BD-01 | 1.652054795 | 0 | 8.454104481 | High groups |
| TCGA-93-7347-01 | 1.871232877 | 0 | 8.110536031 | High groups |
| TCGA-93-7348-01 | 1.454794521 | 0 | 7.064584774 | Low groups |
| TCGA-93-8067-01 | 0.509589041 | 0 | 8.769918751 | High groups |
| TCGA-93-A4JN-01 | 1.967123288 | 0 | 8.147314586 | High groups |
| TCGA-93-A4JO-01 | 0.090410959 | 0 | 8.264126017 | High groups |
| TCGA-93-A4JP-01 | 1.361643836 | 1 | 8.628511303 | High groups |
| TCGA-93-A4JQ-01 | 1.44109589 | 0 | 8.024648796 | Low groups |
| TCGA-95-7039-01 | 3.446575342 | 1 | 7.521617085 | Low groups |
| TCGA-95-7043-01 | 1.378082192 | 1 | 8.28696845 | High groups |
| TCGA-95-7562-01 | 0.238356164 | 0 | 9.148280311 | High groups |
| TCGA-95-7567-01 | 1.556164384 | 0 | 7.210889692 | Low groups |
| TCGA-95-7944-01 | 1.032876712 | 0 | 8.671055829 | High groups |
| TCGA-95-7947-01 | 1.306849315 | 0 | 8.826526878 | High groups |
| TCGA-95-7948-01 | 1.304109589 | 0 | 7.455917413 | Low groups |
| TCGA-95-8039-01 | 0.624657534 | 1 | 7.522590476 | Low groups |
| TCGA-95-8494-01 | 0.230136986 | 0 | 7.275605873 | Low groups |
| TCGA-95-A4VK-01 | 1.35890411 | 1 | 7.718201866 | Low groups |
| TCGA-95-A4VN-01 | 1.515068493 | 0 | 7.740198273 | Low groups |
| TCGA-95-A4VP-01 | 0.591780822 | 1 | 7.952223298 | Low groups |
| TCGA-97-7546-01 | 3.438356164 | 1 | 8.87934468 | High groups |
| TCGA-97-7547-01 | 2.123287671 | 1 | 7.936722673 | Low groups |
| TCGA-97-7552-01 | 2.18630137 | 1 | 7.534129243 | Low groups |
| TCGA-97-7553-01 | 5.123287671 | 0 | 7.625212763 | Low groups |
| TCGA-97-7554-01 | 2.123287671 | 0 | 7.495422873 | Low groups |
| TCGA-97-7937-01 | 1.545205479 | 0 | 6.939132997 | Low groups |
| TCGA-97-7938-01 | 0.049315068 | 0 | 7.152898605 | Low groups |
| TCGA-97-7941-01 | 1.326027397 | 0 | 8.153207213 | High groups |
| TCGA-97-8171-01 | 1.208219178 | 1 | 9.274058314 | High groups |
| TCGA-97-8172-01 | 1.493150685 | 0 | 7.386516705 | Low groups |
| TCGA-97-8174-01 | 0.449315068 | 0 | 8.656766565 | High groups |
| TCGA-97-8175-01 | 0.805479452 | 1 | 8.219457902 | High groups |
| TCGA-97-8176-01 | 0.106849315 | 1 | 8.446525469 | High groups |
| TCGA-97-8177-01 | 1.367123288 | 0 | 7.0335448 | Low groups |
| TCGA-97-8179-01 | 1.191780822 | 0 | 7.398344555 | Low groups |
| TCGA-97-8547-01 | 1.8 | 0 | 7.339112081 | Low groups |
| TCGA-97-8552-01 | 1.715068493 | 0 | 8.482868244 | High groups |
| TCGA-97-A4LX-01 | 1.682191781 | 0 | 8.724120895 | High groups |
| TCGA-97-A4M0-01 | 1.78630137 | 0 | 8.0240509 | Low groups |
| TCGA-97-A4M1-01 | 1.646575342 | 0 | 8.93053511 | High groups |
| TCGA-97-A4M2-01 | 1.709589041 | 0 | 8.731453586 | High groups |
| TCGA-97-A4M3-01 | 0.147945205 | 1 | 8.475792596 | High groups |
| TCGA-97-A4M5-01 | 1.736986301 | 0 | 7.320928863 | Low groups |
| TCGA-97-A4M6-01 | 1.556164384 | 0 | 8.706349824 | High groups |
| TCGA-97-A4M7-01 | 1.723287671 | 0 | 8.027674926 | Low groups |
| TCGA-99-7458-01 | 2.046575342 | 0 | 7.198839856 | Low groups |
| TCGA-99-8025-01 | 2.904109589 | 0 | 7.54028538 | Low groups |
| TCGA-99-8028-01 | 3.063013699 | 0 | 8.433337982 | High groups |
| TCGA-99-8032-01 | 0.120547945 | 0 | 8.144379363 | High groups |
| TCGA-99-8033-01 | 1.797260274 | 1 | 7.85914867 | Low groups |
| TCGA-99-AA5R-01 | 1.802739726 | 0 | 8.558289449 | High groups |
| TCGA-J2-8192-01 | 1.320547945 | 1 | 7.885158846 | Low groups |
| TCGA-J2-8194-01 | 1.287671233 | 1 | 8.194781966 | High groups |
| TCGA-J2-A4AD-01 | 1.435616438 | 1 | 8.674288821 | High groups |
| TCGA-J2-A4AE-01 | 2.956164384 | 0 | 8.530162063 | High groups |
| TCGA-J2-A4AG-01 | 2.706849315 | 0 | 8.210126694 | High groups |
| TCGA-L4-A4E5-01 | 1.583561644 | 0 | 8.786955945 | High groups |
| TCGA-L4-A4E6-01 | 1.191780822 | 0 | 9.021789065 | High groups |
| TCGA-L9-A443-01 | 0.528767123 | 0 | 7.064367157 | Low groups |
| TCGA-L9-A444-01 | 0.84109589 | 0 | 8.618774757 | High groups |
| TCGA-L9-A50W-01 | 1.065753425 | 1 | 8.320080082 | High groups |
| TCGA-L9-A5IP-01 | 0.134246575 | 1 | 8.24842084 | High groups |
| TCGA-L9-A743-01 | 1.819178082 | 0 | 8.905001232 | High groups |
| TCGA-L9-A7SV-01 | 1.547945205 | 0 | 9.440441901 | High groups |
| TCGA-L9-A8F4-01 | 1.304109589 | 0 | 8.530452183 | High groups |
| TCGA-MN-A4N1-01 | 2.265753425 | 0 | 9.603669922 | High groups |
| TCGA-MN-A4N4-01 | 3.219178082 | 0 | 7.816972491 | Low groups |
| TCGA-MN-A4N5-01 | 0.230136986 | 0 | 8.416767386 | High groups |
| TCGA-MP-A4SV-01 | 7.178082192 | 0 | 8.36531123 | High groups |
| TCGA-MP-A4SW-01 | 4.871232877 | 0 | 7.773405364 | Low groups |
| TCGA-MP-A4SY-01 | 1.339726027 | 1 | 7.988482014 | Low groups |
| TCGA-MP-A4T2-01 | 1.575342466 | 1 | 8.856046051 | High groups |
| TCGA-MP-A4T4-01 | 7.169863014 | 0 | 7.642538841 | Low groups |
| TCGA-MP-A4T6-01 | 4.904109589 | 0 | 8.243674459 | High groups |
| TCGA-MP-A4T7-01 | 0.457534247 | 0 | 8.980655799 | High groups |
| TCGA-MP-A4T8-01 | 0.44109589 | 0 | 8.346070973 | High groups |
| TCGA-MP-A4T9-01 | 0.934246575 | 1 | 8.146563228 | High groups |
| TCGA-MP-A4TA-01 | 1.98630137 | 1 | 8.725463843 | High groups |
| TCGA-MP-A4TC-01 | 0.202739726 | 0 | 8.629174377 | High groups |
| TCGA-MP-A4TD-01 | 0.619178082 | 1 | 7.473088769 | Low groups |
| TCGA-MP-A4TE-01 | 0.619178082 | 1 | 6.810075057 | Low groups |
| TCGA-MP-A4TF-01 | 0.534246575 | 1 | 8.036402557 | Low groups |
| TCGA-MP-A4TH-01 | 2.030136986 | 0 | 8.219256145 | High groups |
| TCGA-MP-A4TI-01 | 0.230136986 | 1 | 7.405575823 | Low groups |
| TCGA-MP-A4TJ-01 | 0.928767123 | 0 | 8.128009792 | High groups |
| TCGA-MP-A4TK-01 | 1.087671233 | 1 | 8.286158511 | High groups |
| TCGA-MP-A5C7-01 | 6.15890411 | 0 | 8.624057821 | High groups |
| TCGA-NJ-A4YF-01 | 5.920547945 | 0 | 7.884391135 | Low groups |
| TCGA-NJ-A4YG-01 | 6.194520548 | 0 | 7.729277787 | Low groups |
| TCGA-NJ-A4YI-01 | 0.010958904 | 0 | 6.886338215 | Low groups |
| TCGA-NJ-A4YP-01 | 0.136986301 | 0 | 7.91849423 | Low groups |
| TCGA-NJ-A4YQ-01 | 3.923287671 | 0 | 8.51860536 | High groups |
| TCGA-NJ-A55A-01 | 0.04109589 | 0 | 8.614735349 | High groups |
| TCGA-NJ-A55O-01 | 0.035616438 | 0 | 8.795418256 | High groups |
| TCGA-NJ-A55R-01 | 1.652054795 | 0 | 7.885337943 | Low groups |
| TCGA-NJ-A7XG-01 | 1.690410959 | 0 | 6.902265001 | Low groups |
| TCGA-O1-A52J-01 | 2.460273973 | 1 | 8.560147737 | High groups |
| TCGA-S2-AA1A-01 | 1.405479452 | 0 | 7.646687224 | Low groups |

**Table S7.** Raw counts of RNA-sequencing data of hsa-miR-199a-5p in LUAD from the TCGA.

| sampleID | time | Staus | RS | Label |
| --- | --- | --- | --- | --- |
| TCGA-05-4244-01 | 0 | 0 | 8.341713038 | Low groups |
| TCGA-05-4249-01 | 4.17260274 | 0 | 8.54522031 | Low groups |
| TCGA-05-4250-01 | 0.331506849 | 0 | 10.06443749 | High groups |
| TCGA-05-4382-01 | 0.915068493 | 1 | 10.89057893 | High groups |
| TCGA-05-4384-01 | 0.501369863 | 1 | 8.481574233 | Low groups |
| TCGA-05-4389-01 | 3.750684932 | 0 | 8.483127391 | Low groups |
| TCGA-05-4390-01 | 1.082191781 | 1 | 8.232839548 | Low groups |
| TCGA-05-4395-01 | 0 | 0 | 8.606574995 | Low groups |
| TCGA-05-4396-01 | 0.830136986 | 0 | 8.21187467 | Low groups |
| TCGA-05-4397-01 | 2.002739726 | 0 | 7.664874963 | Low groups |
| TCGA-05-4398-01 | 3.920547945 | 0 | 9.924269313 | High groups |
| TCGA-05-4402-01 | 0.668493151 | 0 | 10.45670986 | High groups |
| TCGA-05-4403-01 | 1.583561644 | 0 | 9.262980398 | Low groups |
| TCGA-05-4405-01 | 1.671232877 | 0 | 8.934916867 | Low groups |
| TCGA-05-4410-01 | 0 | 0 | 8.633837456 | Low groups |
| TCGA-05-4415-01 | 0.164383562 | 1 | 6.696099701 | Low groups |
| TCGA-05-4417-01 | 1.246575342 | 0 | 9.462541944 | Low groups |
| TCGA-05-4418-01 | 0.750684932 | 0 | 9.15121193 | Low groups |
| TCGA-05-4420-01 | 2.498630137 | 0 | 8.807955061 | Low groups |
| TCGA-05-4422-01 | 1 | 0 | 8.162632866 | Low groups |
| TCGA-05-4424-01 | 0.419178082 | 1 | 8.934321232 | Low groups |
| TCGA-05-4425-01 | 1.832876712 | 0 | 9.947122779 | High groups |
| TCGA-05-4426-01 | 1.252054795 | 1 | 8.967164572 | Low groups |
| TCGA-05-4427-01 | 2.167123288 | 0 | 7.908398325 | Low groups |
| TCGA-05-4430-01 | 2.084931507 | 0 | 10.16864304 | High groups |
| TCGA-05-4432-01 | 2.084931507 | 0 | 9.103433802 | Low groups |
| TCGA-05-4433-01 | 2 | 0 | 7.526058949 | Low groups |
| TCGA-05-4434-01 | 1.252054795 | 0 | 9.966998811 | High groups |
| TCGA-05-5420-01 | 0.671232877 | 1 | 8.057580131 | Low groups |
| TCGA-05-5423-01 | 0.41369863 | 0 | 9.008854476 | Low groups |
| TCGA-05-5425-01 | 1.331506849 | 1 | 8.963032057 | Low groups |
| TCGA-05-5428-01 | 1.835616438 | 0 | 9.196166117 | Low groups |
| TCGA-05-5429-01 | 0.753424658 | 0 | 10.17135547 | High groups |
| TCGA-05-5715-01 | 0.169863014 | 0 | 9.067565313 | Low groups |
| TCGA-35-3615-01 | 0.038356164 | 0 | 9.830114746 | High groups |
| TCGA-35-4122-01 | 0.616438356 | 0 | 10.51567569 | High groups |
| TCGA-35-4123-01 | 0.498630137 | 0 | 10.31241514 | High groups |
| TCGA-35-5375-01 | 0.723287671 | 0 | 8.531530206 | Low groups |
| TCGA-38-4625-01 | 8.145205479 | 0 | 9.411520521 | Low groups |
| TCGA-38-4626-01 | 6.898630137 | 1 | 9.859310212 | High groups |
| TCGA-38-4627-01 | 3.142465753 | 0 | 11.23476523 | High groups |
| TCGA-38-4628-01 | 2.967123288 | 1 | 11.05272087 | High groups |
| TCGA-38-4629-01 | 1.038356164 | 1 | 10.24222783 | High groups |
| TCGA-38-4630-01 | 1.435616438 | 1 | 8.77451615 | Low groups |
| TCGA-38-4631-01 | 0.969863014 | 1 | 8.61571239 | Low groups |
| TCGA-38-4632-01 | 1.863013699 | 1 | 8.228029946 | Low groups |
| TCGA-38-6178-01 | 1.22739726 | 0 | 8.990495524 | Low groups |
| TCGA-38-7271-01 | 0.832876712 | 1 | 8.584610801 | Low groups |
| TCGA-38-A44F-01 | 0.364383562 | 0 | 10.11660349 | High groups |
| TCGA-44-2655-01 | 2.764383562 | 1 | 9.794157929 | High groups |
| TCGA-44-2656-01 | 1.556164384 | 1 | 9.160484503 | Low groups |
| TCGA-44-2657-01 | 3.701369863 | 0 | 9.283118689 | Low groups |
| TCGA-44-2659-01 | 3.139726027 | 1 | 9.928421551 | High groups |
| TCGA-44-2661-01 | 3.175342466 | 0 | 8.739242072 | Low groups |
| TCGA-44-2662-01 | 0.671232877 | 1 | 9.827611686 | High groups |
| TCGA-44-2665-01 | 3.564383562 | 0 | 10.69801406 | High groups |
| TCGA-44-2666-01 | 0.265753425 | 1 | 8.181527334 | Low groups |
| TCGA-44-2668-01 | 1.18630137 | 1 | 9.573930019 | High groups |
| TCGA-44-3396-01 | 3.095890411 | 0 | 10.74891368 | High groups |
| TCGA-44-3398-01 | 3.18630137 | 0 | 10.85907527 | High groups |
| TCGA-44-3918-01 | 1.4 | 1 | 9.503123296 | High groups |
| TCGA-44-3919-01 | 2.523287671 | 1 | 9.651132186 | High groups |
| TCGA-44-4112-01 | 1.695890411 | 1 | 9.813038894 | High groups |
| TCGA-44-5643-01 | 2.775342466 | 0 | 7.750832712 | Low groups |
| TCGA-44-5644-01 | 2.364383562 | 0 | 6.912043945 | Low groups |
| TCGA-44-5645-01 | 2.334246575 | 0 | 9.361866287 | Low groups |
| TCGA-44-6144-01 | 1.093150685 | 1 | 10.5713687 | High groups |
| TCGA-44-6145-01 | 1.630136986 | 0 | 9.124596547 | Low groups |
| TCGA-44-6146-01 | 1.728767123 | 1 | 10.01654563 | High groups |
| TCGA-44-6147-01 | 2.315068493 | 0 | 10.27071009 | High groups |
| TCGA-44-6148-01 | 1.928767123 | 0 | 10.65579795 | High groups |
| TCGA-44-6774-01 | 1.802739726 | 0 | 9.934095379 | High groups |
| TCGA-44-6775-01 | 1.873972603 | 1 | 10.96922706 | High groups |
| TCGA-44-6776-01 | 7.167123288 | 0 | 7.906263428 | Low groups |
| TCGA-44-6777-01 | 2.704109589 | 0 | 10.20891481 | High groups |
| TCGA-44-6778-01 | 5.106849315 | 0 | 7.523585164 | Low groups |
| TCGA-44-6779-01 | 0.635616438 | 1 | 8.848809446 | Low groups |
| TCGA-44-7659-01 | 1.893150685 | 0 | 9.551636096 | High groups |
| TCGA-44-7660-01 | 0.693150685 | 1 | 9.312500772 | Low groups |
| TCGA-44-7661-01 | 0.917808219 | 1 | 8.569137423 | Low groups |
| TCGA-44-7662-01 | 0.597260274 | 0 | 9.188116276 | Low groups |
| TCGA-44-7667-01 | 3.005479452 | 0 | 8.134405013 | Low groups |
| TCGA-44-7669-01 | 1.068493151 | 1 | 7.587567068 | Low groups |
| TCGA-44-7670-01 | 2.416438356 | 0 | 8.154755338 | Low groups |
| TCGA-44-7671-01 | 2.419178082 | 1 | 9.023744541 | Low groups |
| TCGA-44-7672-01 | 1.969863014 | 0 | 9.477317439 | Low groups |
| TCGA-44-8117-01 | 1.054794521 | 0 | 9.629975098 | High groups |
| TCGA-44-8119-01 | 0.780821918 | 0 | 9.522257766 | High groups |
| TCGA-44-8120-01 | 0.712328767 | 0 | 10.55263874 | High groups |
| TCGA-44-A479-01 | 1.197260274 | 1 | 10.55185493 | High groups |
| TCGA-44-A47A-01 | 1.087671233 | 1 | 9.573950841 | High groups |
| TCGA-44-A47B-01 | 0.78630137 | 0 | 9.713201763 | High groups |
| TCGA-44-A47F-01 | 0.923287671 | 0 | 9.718855456 | High groups |
| TCGA-44-A47G-01 | 0.961643836 | 0 | 10.46093961 | High groups |
| TCGA-44-A4SS-01 | 1.136986301 | 0 | 10.56625891 | High groups |
| TCGA-44-A4SU-01 | 0.712328767 | 1 | 10.65579902 | High groups |
| TCGA-49-4486-01 | 5.602739726 | 1 | 8.068249462 | Low groups |
| TCGA-49-4487-01 | 1.909589041 | 1 | 8.998799048 | Low groups |
| TCGA-49-4488-01 | 1.736986301 | 1 | 9.193447593 | Low groups |
| TCGA-49-4490-01 | 1.054794521 | 1 | 9.824936154 | High groups |
| TCGA-49-4494-01 | 2.961643836 | 1 | 9.673082008 | High groups |
| TCGA-49-4501-01 | 1.493150685 | 1 | 10.42954159 | High groups |
| TCGA-49-4505-01 | 1.142465753 | 1 | 9.819653656 | High groups |
| TCGA-49-4506-01 | 1.895890411 | 1 | 9.100129311 | Low groups |
| TCGA-49-4507-01 | 0.432876712 | 1 | 9.043271769 | Low groups |
| TCGA-49-4510-01 | 1.375342466 | 1 | 9.085422691 | Low groups |
| TCGA-49-4512-01 | 2.479452055 | 1 | 9.696924662 | High groups |
| TCGA-49-4514-01 | 4.657534247 | 0 | 8.662326859 | Low groups |
| TCGA-49-6742-01 | 0.58630137 | 1 | 8.19296276 | Low groups |
| TCGA-49-6743-01 | 4.44109589 | 0 | 8.537120729 | Low groups |
| TCGA-49-6744-01 | 4.610958904 | 0 | 9.218611184 | Low groups |
| TCGA-49-6745-01 | 1.430136986 | 0 | 9.178206352 | Low groups |
| TCGA-49-6761-01 | 0.969863014 | 0 | 9.477611561 | Low groups |
| TCGA-49-6767-01 | 1.854794521 | 0 | 8.709617838 | Low groups |
| TCGA-49-AAQV-01 | 1.394520548 | 1 | 10.13033323 | High groups |
| TCGA-49-AAR0-01 | 13.05479452 | 0 | 10.15749069 | High groups |
| TCGA-49-AAR2-01 | 6.093150685 | 0 | 8.736362794 | Low groups |
| TCGA-49-AAR3-01 | 5.18630137 | 1 | 10.42265918 | High groups |
| TCGA-49-AAR4-01 | 2.408219178 | 1 | 9.733106797 | High groups |
| TCGA-49-AAR9-01 | 0.712328767 | 1 | 9.522516604 | High groups |
| TCGA-49-AARE-01 | 1.063013699 | 1 | 9.282416962 | Low groups |
| TCGA-49-AARN-01 | 3.109589041 | 0 | 9.790454996 | High groups |
| TCGA-49-AARO-01 | 3.134246575 | 1 | 10.54385475 | High groups |
| TCGA-49-AARQ-01 | 18.44383562 | 0 | 9.624495191 | High groups |
| TCGA-49-AARR-01 | 3.010958904 | 1 | 9.407180435 | Low groups |
| TCGA-4B-A93V-01 | 0.687671233 | 1 | 8.877479701 | Low groups |
| TCGA-50-5044-01 | 1.323287671 | 1 | 8.636012542 | Low groups |
| TCGA-50-5045-01 | 3.926027397 | 1 | 9.90225692 | High groups |
| TCGA-50-5049-01 | 4.295890411 | 1 | 9.97887202 | High groups |
| TCGA-50-5051-01 | 0.504109589 | 1 | 8.0852891 | Low groups |
| TCGA-50-5055-01 | 2.112328767 | 1 | 10.07031378 | High groups |
| TCGA-50-5066-01 | 1.306849315 | 1 | 9.738847365 | High groups |
| TCGA-50-5068-01 | 1.879452055 | 1 | 9.263472736 | Low groups |
| TCGA-50-5072-01 | 0.583561644 | 1 | 8.810325681 | Low groups |
| TCGA-50-5930-01 | 0.484931507 | 1 | 9.315235963 | Low groups |
| TCGA-50-5931-01 | 1.164383562 | 1 | 9.069942522 | Low groups |
| TCGA-50-5932-01 | 2.98630137 | 1 | 8.038568421 | Low groups |
| TCGA-50-5933-01 | 6.556164384 | 0 | 9.24368217 | Low groups |
| TCGA-50-5935-01 | 1.789041096 | 0 | 9.077613267 | Low groups |
| TCGA-50-5936-01 | 0.410958904 | 1 | 8.86662799 | Low groups |
| TCGA-50-5939-01 | 1.260273973 | 0 | 8.727592817 | Low groups |
| TCGA-50-5941-01 | 4.038356164 | 0 | 8.100045711 | Low groups |
| TCGA-50-5942-01 | 3.775342466 | 1 | 8.997494863 | Low groups |
| TCGA-50-5944-01 | 4.794520548 | 0 | 8.832300635 | Low groups |
| TCGA-50-5946-01 | 0.605479452 | 1 | 10.38249845 | High groups |
| TCGA-50-6590-01 | 3.528767123 | 0 | 8.543803436 | Low groups |
| TCGA-50-6591-01 | 0.326027397 | 1 | 11.89182254 | High groups |
| TCGA-50-6592-01 | 2.128767123 | 1 | 8.815574148 | Low groups |
| TCGA-50-6593-01 | 0.728767123 | 1 | 9.070599545 | Low groups |
| TCGA-50-6594-01 | 0.780821918 | 1 | 8.964081882 | Low groups |
| TCGA-50-6595-01 | 0.498630137 | 1 | 9.6095474 | High groups |
| TCGA-50-6597-01 | 3.473972603 | 0 | 8.90443129 | Low groups |
| TCGA-50-6673-01 | 0.060273973 | 0 | 10.45535599 | High groups |
| TCGA-50-7109-01 | 0.04109589 | 1 | 9.118035731 | Low groups |
| TCGA-50-8457-01 | 3.082191781 | 0 | 10.52772662 | High groups |
| TCGA-50-8459-01 | 1.183561644 | 1 | 10.66910438 | High groups |
| TCGA-50-8460-01 | 2.271232877 | 0 | 8.798139967 | Low groups |
| TCGA-53-7624-01 | 1.095890411 | 1 | 7.340686879 | Low groups |
| TCGA-53-7626-01 | 2.369863014 | 1 | 8.095763848 | Low groups |
| TCGA-53-7813-01 | 1.161643836 | 0 | 8.125889985 | Low groups |
| TCGA-53-A4EZ-01 | 2.934246575 | 0 | 9.579507341 | High groups |
| TCGA-55-1592-01 | 1.238356164 | 1 | 10.02942597 | High groups |
| TCGA-55-1594-01 | 3.22739726 | 0 | 8.586333536 | Low groups |
| TCGA-55-1595-01 | 4.052054795 | 0 | 10.58476139 | High groups |
| TCGA-55-1596-01 | 5.657534247 | 0 | 7.946615309 | Low groups |
| TCGA-55-5899-01 | 2.547945205 | 0 | 8.496754093 | Low groups |
| TCGA-55-6543-01 | 1.191780822 | 0 | 8.25269561 | Low groups |
| TCGA-55-6642-01 | 6.709589041 | 0 | 9.360922124 | Low groups |
| TCGA-55-6712-01 | 0.468493151 | 1 | 9.034309821 | Low groups |
| TCGA-55-6968-01 | 3.542465753 | 1 | 8.992616235 | Low groups |
| TCGA-55-6969-01 | 3.394520548 | 0 | 10.92245566 | High groups |
| TCGA-55-6970-01 | 1.252054795 | 1 | 9.034236353 | Low groups |
| TCGA-55-6971-01 | 3.835616438 | 0 | 9.02835891 | Low groups |
| TCGA-55-6972-01 | 4.471232877 | 0 | 8.840884808 | Low groups |
| TCGA-55-6978-01 | 0.115068493 | 1 | 10.97701684 | High groups |
| TCGA-55-6979-01 | 0.534246575 | 1 | 9.505758568 | High groups |
| TCGA-55-6980-01 | 5.778082192 | 0 | 11.17606703 | High groups |
| TCGA-55-6981-01 | 3.778082192 | 0 | 10.26873076 | High groups |
| TCGA-55-6982-01 | 0.501369863 | 1 | 11.79982022 | High groups |
| TCGA-55-6983-01 | 7.734246575 | 0 | 10.91086583 | High groups |
| TCGA-55-6984-01 | 1.983561644 | 1 | 9.618443429 | High groups |
| TCGA-55-6985-01 | 3.378082192 | 0 | 10.07069268 | High groups |
| TCGA-55-6986-01 | 8.934246575 | 0 | 10.4507369 | High groups |
| TCGA-55-6987-01 | 5.854794521 | 0 | 9.075851715 | Low groups |
| TCGA-55-7227-01 | 0.698630137 | 1 | 9.211830975 | Low groups |
| TCGA-55-7281-01 | 0.928767123 | 1 | 9.58177585 | High groups |
| TCGA-55-7283-01 | 1.668493151 | 0 | 7.667246471 | Low groups |
| TCGA-55-7284-01 | 0.632876712 | 1 | 9.971953744 | High groups |
| TCGA-55-7570-01 | 2.257534247 | 0 | 9.506607595 | High groups |
| TCGA-55-7573-01 | 1.334246575 | 0 | 9.029385134 | Low groups |
| TCGA-55-7574-01 | 1.309589041 | 1 | 9.1342556 | Low groups |
| TCGA-55-7576-01 | 1.835616438 | 0 | 8.659639365 | Low groups |
| TCGA-55-7724-01 | 1.931506849 | 0 | 8.132706486 | Low groups |
| TCGA-55-7725-01 | 1.210958904 | 0 | 6.627317774 | Low groups |
| TCGA-55-7726-01 | 1.78630137 | 0 | 9.866389216 | High groups |
| TCGA-55-7727-01 | 0.326027397 | 0 | 8.430605678 | Low groups |
| TCGA-55-7728-01 | 1.928767123 | 0 | 9.210907926 | Low groups |
| TCGA-55-7815-01 | 1.276712329 | 1 | 9.301812593 | Low groups |
| TCGA-55-7816-01 | 1.282191781 | 1 | 9.630061674 | High groups |
| TCGA-55-7903-01 | 1.553424658 | 0 | 7.943302846 | Low groups |
| TCGA-55-7907-01 | 0.805479452 | 1 | 9.034523435 | Low groups |
| TCGA-55-7910-01 | 2.789041096 | 1 | 7.22358357 | Low groups |
| TCGA-55-7911-01 | 1.410958904 | 1 | 7.293400926 | Low groups |
| TCGA-55-7913-01 | 1.315068493 | 1 | 7.062674471 | Low groups |
| TCGA-55-7914-01 | 0.512328767 | 1 | 8.221597271 | Low groups |
| TCGA-55-7994-01 | 1.652054795 | 0 | 8.040280514 | Low groups |
| TCGA-55-7995-01 | 1.282191781 | 1 | 9.424460552 | Low groups |
| TCGA-55-8085-01 | 2.476712329 | 0 | 9.31794898 | Low groups |
| TCGA-55-8087-01 | 1.265753425 | 0 | 10.40022762 | High groups |
| TCGA-55-8089-01 | 1.923287671 | 0 | 9.750680024 | High groups |
| TCGA-55-8090-01 | 1.501369863 | 1 | 10.76146148 | High groups |
| TCGA-55-8091-01 | 1.643835616 | 0 | 10.77717705 | High groups |
| TCGA-55-8092-01 | 0.347945205 | 1 | 9.9957938 | High groups |
| TCGA-55-8094-01 | 1.482191781 | 0 | 7.72598765 | Low groups |
| TCGA-55-8096-01 | 1.550684932 | 1 | 11.65181859 | High groups |
| TCGA-55-8097-01 | 1.304109589 | 0 | 9.840536972 | High groups |
| TCGA-55-8203-01 | 1.498630137 | 0 | 10.00160762 | High groups |
| TCGA-55-8204-01 | 1.410958904 | 0 | 10.16947196 | High groups |
| TCGA-55-8205-01 | 1.356164384 | 1 | 9.807497735 | High groups |
| TCGA-55-8206-01 | 2.432876712 | 0 | 9.689161525 | High groups |
| TCGA-55-8207-01 | 2.676712329 | 0 | 10.65066351 | High groups |
| TCGA-55-8208-01 | 1.4 | 1 | 9.908294586 | High groups |
| TCGA-55-8299-01 | 0.750684932 | 1 | 10.86926155 | High groups |
| TCGA-55-8301-01 | 0.652054795 | 1 | 10.40758973 | High groups |
| TCGA-55-8302-01 | 1.309589041 | 0 | 9.758779028 | High groups |
| TCGA-55-8505-01 | 1.205479452 | 0 | 11.18889528 | High groups |
| TCGA-55-8506-01 | 0.030136986 | 0 | 9.27810396 | Low groups |
| TCGA-55-8507-01 | 1.145205479 | 0 | 9.529359491 | High groups |
| TCGA-55-8508-01 | 1.690410959 | 0 | 9.987662155 | High groups |
| TCGA-55-8510-01 | 1.476712329 | 0 | 10.69580047 | High groups |
| TCGA-55-8511-01 | 1.260273973 | 1 | 10.7204199 | High groups |
| TCGA-55-8512-01 | 1.663013699 | 1 | 9.611205953 | High groups |
| TCGA-55-8513-01 | 0.868493151 | 1 | 10.15244546 | High groups |
| TCGA-55-8514-01 | 1.424657534 | 0 | 10.01123019 | High groups |
| TCGA-55-8614-01 | 1.468493151 | 0 | 10.93585477 | High groups |
| TCGA-55-8615-01 | 0.61369863 | 1 | 9.328601332 | Low groups |
| TCGA-55-8616-01 | 0.131506849 | 0 | 10.78521238 | High groups |
| TCGA-55-8619-01 | 1.139726027 | 0 | 10.02109893 | High groups |
| TCGA-55-8620-01 | 1.02739726 | 1 | 7.670084014 | Low groups |
| TCGA-55-8621-01 | 1.410958904 | 0 | 10.19250242 | High groups |
| TCGA-55-A48X-01 | 1.684931507 | 1 | 9.59350504 | High groups |
| TCGA-55-A48Y-01 | 1.726027397 | 0 | 10.44619243 | High groups |
| TCGA-55-A48Z-01 | 1.468493151 | 1 | 10.87576809 | High groups |
| TCGA-55-A490-01 | 0.271232877 | 0 | 9.238607037 | Low groups |
| TCGA-55-A491-01 | 1.715068493 | 0 | 9.484409779 | Low groups |
| TCGA-55-A492-01 | 1.632876712 | 0 | 9.380497538 | Low groups |
| TCGA-55-A493-01 | 0.076712329 | 0 | 9.772576363 | High groups |
| TCGA-55-A494-01 | 1.317808219 | 0 | 8.058389343 | Low groups |
| TCGA-55-A4DF-01 | 1.410958904 | 1 | 9.422091979 | Low groups |
| TCGA-55-A4DG-01 | 1.665753425 | 0 | 10.22761354 | High groups |
| TCGA-55-A57B-01 | 1.495890411 | 0 | 11.14802858 | High groups |
| TCGA-62-8394-01 | 0.380821918 | 0 | 9.527129388 | High groups |
| TCGA-62-8395-01 | 1.082191781 | 1 | 10.69447594 | High groups |
| TCGA-62-8397-01 | 3.531506849 | 0 | 9.868810597 | High groups |
| TCGA-62-8398-01 | 1.216438356 | 0 | 9.015015782 | Low groups |
| TCGA-62-8399-01 | 7.38630137 | 0 | 9.900886275 | High groups |
| TCGA-62-8402-01 | 2.115068493 | 1 | 7.685046104 | Low groups |
| TCGA-62-A46O-01 | 2.583561644 | 1 | 8.780006027 | Low groups |
| TCGA-62-A46P-01 | 0.731506849 | 1 | 9.410818413 | Low groups |
| TCGA-62-A46R-01 | 4.726027397 | 0 | 9.120019966 | Low groups |
| TCGA-62-A46S-01 | 1.443835616 | 1 | 9.041586604 | Low groups |
| TCGA-62-A46U-01 | 5.663013699 | 0 | 7.29806651 | Low groups |
| TCGA-62-A46V-01 | 6.024657534 | 0 | 10.61674134 | High groups |
| TCGA-62-A46Y-01 | 0.860273973 | 1 | 8.425266685 | Low groups |
| TCGA-62-A470-01 | 1.476712329 | 1 | 9.29828119 | Low groups |
| TCGA-62-A471-01 | 3.41369863 | 0 | 8.606972074 | Low groups |
| TCGA-62-A472-01 | 0.794520548 | 1 | 9.195720775 | Low groups |
| TCGA-64-1676-01 | 4.734246575 | 0 | 9.550195686 | High groups |
| TCGA-64-1677-01 | 0.98630137 | 1 | 8.271015116 | Low groups |
| TCGA-64-1678-01 | 3.257534247 | 0 | 9.036809907 | Low groups |
| TCGA-64-1679-01 | 6.816438356 | 0 | 10.06265737 | High groups |
| TCGA-64-1680-01 | 3.084931507 | 0 | 8.700552008 | Low groups |
| TCGA-64-1681-01 | 1.202739726 | 1 | 9.505997584 | High groups |
| TCGA-64-5774-01 | 0.673972603 | 1 | 7.713035689 | Low groups |
| TCGA-64-5775-01 | 0.169863014 | 1 | 8.291592692 | Low groups |
| TCGA-64-5778-01 | 2.635616438 | 1 | 8.949658421 | Low groups |
| TCGA-64-5779-01 | 2.178082192 | 1 | 10.02672953 | High groups |
| TCGA-64-5781-01 | 0.263013699 | 1 | 9.882968001 | High groups |
| TCGA-64-5815-01 | 2.37260274 | 0 | 10.3916177 | High groups |
| TCGA-67-3770-01 | 1.671232877 | 0 | 10.11879446 | High groups |
| TCGA-67-3771-01 | 1.671232877 | 0 | 9.42485276 | Low groups |
| TCGA-67-3772-01 | 1.569863014 | 0 | 9.717514942 | High groups |
| TCGA-67-3773-01 | 1.169863014 | 0 | 8.685629219 | Low groups |
| TCGA-67-3774-01 | 1.054794521 | 0 | 9.381985641 | Low groups |
| TCGA-67-4679-01 | 1.22739726 | 0 | 8.372712731 | Low groups |
| TCGA-67-6215-01 | 0.476712329 | 0 | 7.527293627 | Low groups |
| TCGA-67-6216-01 | 0.38630137 | 0 | 8.439354269 | Low groups |
| TCGA-67-6217-01 | 0.810958904 | 1 | 9.355634454 | Low groups |
| TCGA-69-7760-01 | 0.553424658 | 0 | 8.832549549 | Low groups |
| TCGA-69-7761-01 | 0.509589041 | 0 | 8.950635502 | Low groups |
| TCGA-69-7763-01 | 1.890410959 | 0 | 9.434472587 | Low groups |
| TCGA-69-7764-01 | 1.134246575 | 0 | 8.708774695 | Low groups |
| TCGA-69-7765-01 | 0.452054795 | 0 | 9.44519988 | Low groups |
| TCGA-69-7973-01 | 0.630136986 | 0 | 9.556947322 | High groups |
| TCGA-69-7974-01 | 0.504109589 | 0 | 9.682907879 | High groups |
| TCGA-69-7978-01 | 0.367123288 | 0 | 10.9061108 | High groups |
| TCGA-69-7979-01 | 1.117808219 | 0 | 9.601009264 | High groups |
| TCGA-69-7980-01 | 1.126027397 | 0 | 10.52075676 | High groups |
| TCGA-69-8253-01 | 1.167123288 | 0 | 9.817344684 | High groups |
| TCGA-69-8254-01 | 1.120547945 | 0 | 9.515000395 | High groups |
| TCGA-69-8255-01 | 0.353424658 | 0 | 7.298666013 | Low groups |
| TCGA-69-8453-01 | 0.942465753 | 1 | 10.19949605 | High groups |
| TCGA-69-A59K-01 | 1.619178082 | 0 | 8.917834503 | Low groups |
| TCGA-71-6725-01 | 0.449315068 | 1 | 8.495060846 | Low groups |
| TCGA-71-8520-01 | 0.490410959 | 1 | 10.19739683 | High groups |
| TCGA-73-4658-01 | 4.383561644 | 0 | 9.603402562 | High groups |
| TCGA-73-4659-01 | 0.095890411 | 1 | 10.26383455 | High groups |
| TCGA-73-4662-01 | 0.057534247 | 1 | 10.05400853 | High groups |
| TCGA-73-4675-01 | 0.989041096 | 1 | 10.4673072 | High groups |
| TCGA-73-4676-01 | 0.769863014 | 1 | 8.368977694 | Low groups |
| TCGA-73-7498-01 | 3.257534247 | 0 | 9.935532971 | High groups |
| TCGA-73-7499-01 | 3.964383562 | 1 | 10.04165847 | High groups |
| TCGA-73-A9RS-01 | 0.293150685 | 1 | 9.411246025 | Low groups |
| TCGA-75-5125-01 | 4.8 | 1 | 9.304491192 | Low groups |
| TCGA-75-5146-01 | 4.857534247 | 1 | 8.894010507 | Low groups |
| TCGA-75-5147-01 | 3.652054795 | 0 | 9.9815894 | High groups |
| TCGA-75-6206-01 | 7.095890411 | 0 | 9.265511909 | Low groups |
| TCGA-75-6212-01 | 3.671232877 | 1 | 8.425782378 | Low groups |
| TCGA-75-6214-01 | 1.147945205 | 1 | 10.21389791 | High groups |
| TCGA-75-7025-01 | 4.057534247 | 1 | 10.27338313 | High groups |
| TCGA-75-7027-01 | 8.079452055 | 1 | 9.831832283 | High groups |
| TCGA-78-7143-01 | 4.109589041 | 1 | 7.604843777 | Low groups |
| TCGA-78-7145-01 | 1.156164384 | 1 | 8.168331497 | Low groups |
| TCGA-78-7146-01 | 0.473972603 | 0 | 7.220034071 | Low groups |
| TCGA-78-7147-01 | 1.583561644 | 1 | 8.680522686 | Low groups |
| TCGA-78-7148-01 | 0.498630137 | 1 | 7.927203258 | Low groups |
| TCGA-78-7149-01 | 10.79452055 | 0 | 9.009550127 | Low groups |
| TCGA-78-7150-01 | 0.380821918 | 1 | 9.333279484 | Low groups |
| TCGA-78-7152-01 | 3.293150685 | 1 | 8.66197365 | Low groups |
| TCGA-78-7153-01 | 9.95890411 | 0 | 8.02299303 | Low groups |
| TCGA-78-7154-01 | 1.624657534 | 0 | 7.975976501 | Low groups |
| TCGA-78-7155-01 | 0.57260274 | 1 | 6.662439449 | Low groups |
| TCGA-78-7156-01 | 2.673972603 | 0 | 7.270865937 | Low groups |
| TCGA-78-7158-01 | 0.361643836 | 1 | 8.232932771 | Low groups |
| TCGA-78-7159-01 | 5.408219178 | 0 | 7.847138924 | Low groups |
| TCGA-78-7160-01 | 1.909589041 | 0 | 9.66564379 | High groups |
| TCGA-78-7161-01 | 0.443835616 | 1 | 7.800120634 | Low groups |
| TCGA-78-7162-01 | 6.076712329 | 1 | 8.888773259 | Low groups |
| TCGA-78-7163-01 | 19.85753425 | 0 | 7.962340678 | Low groups |
| TCGA-78-7166-01 | 0.706849315 | 0 | 8.869560902 | Low groups |
| TCGA-78-7167-01 | 1.989041096 | 1 | 8.520222764 | Low groups |
| TCGA-78-7220-01 | 1.454794521 | 1 | 8.223806391 | Low groups |
| TCGA-78-7535-01 | 2.216438356 | 1 | 8.41236 | Low groups |
| TCGA-78-7536-01 | 0.632876712 | 1 | 7.04045708 | Low groups |
| TCGA-78-7537-01 | 4.443835616 | 0 | 9.17997202 | Low groups |
| TCGA-78-7539-01 | 1.884931507 | 1 | 8.342224176 | Low groups |
| TCGA-78-7540-01 | 3.279452055 | 0 | 8.418872755 | Low groups |
| TCGA-78-7542-01 | 0.879452055 | 0 | 7.569780966 | Low groups |
| TCGA-78-7633-01 | 3.97260274 | 1 | 8.179555636 | Low groups |
| TCGA-78-8640-01 | 19.34794521 | 0 | 9.903942323 | High groups |
| TCGA-78-8648-01 | 1.97260274 | 1 | 11.73866103 | High groups |
| TCGA-78-8655-01 | 6.465753425 | 0 | 10.43360025 | High groups |
| TCGA-78-8660-01 | 0.663013699 | 1 | 9.564799985 | High groups |
| TCGA-78-8662-01 | 8.339726027 | 1 | 10.54882239 | High groups |
| TCGA-80-5608-01 | 7.75890411 | 0 | 8.54156014 | Low groups |
| TCGA-80-5611-01 | 7.109589041 | 0 | 7.872810418 | Low groups |
| TCGA-83-5908-01 | 2.257534247 | 0 | 9.870906492 | High groups |
| TCGA-86-6562-01 | 0.750684932 | 1 | 8.887198982 | Low groups |
| TCGA-86-6851-01 | 0.490410959 | 0 | 9.621980525 | High groups |
| TCGA-86-7701-01 | 1.161643836 | 1 | 8.163332877 | Low groups |
| TCGA-86-7711-01 | 2.865753425 | 1 | 8.084835647 | Low groups |
| TCGA-86-7713-01 | 3.169863014 | 0 | 7.357182487 | Low groups |
| TCGA-86-7714-01 | 1.712328767 | 1 | 9.372868977 | Low groups |
| TCGA-86-7953-01 | 2.731506849 | 0 | 9.232831347 | Low groups |
| TCGA-86-7954-01 | 1.657534247 | 0 | 8.514773023 | Low groups |
| TCGA-86-7955-01 | 2.246575342 | 1 | 8.561706467 | Low groups |
| TCGA-86-8054-01 | 3.145205479 | 0 | 8.14840428 | Low groups |
| TCGA-86-8055-01 | 0.339726027 | 0 | 11.54832136 | High groups |
| TCGA-86-8056-01 | 0.380821918 | 0 | 9.937621478 | High groups |
| TCGA-86-8073-01 | 2.02739726 | 0 | 11.44603767 | High groups |
| TCGA-86-8074-01 | 0.065753425 | 0 | 10.28102728 | High groups |
| TCGA-86-8075-01 | 0.545205479 | 1 | 10.57737085 | High groups |
| TCGA-86-8076-01 | 2.720547945 | 0 | 9.895268838 | High groups |
| TCGA-86-8278-01 | 0.079452055 | 1 | 10.42249583 | High groups |
| TCGA-86-8279-01 | 2.6 | 0 | 10.95114394 | High groups |
| TCGA-86-8280-01 | 1.920547945 | 0 | 10.28896882 | High groups |
| TCGA-86-8281-01 | 0 | 0 | 9.572721824 | High groups |
| TCGA-86-8358-01 | 1.789041096 | 0 | 8.64063358 | Low groups |
| TCGA-86-8359-01 | 1.216438356 | 0 | 8.639122886 | Low groups |
| TCGA-86-8585-01 | 0.967123288 | 0 | 10.44438133 | High groups |
| TCGA-86-8668-01 | 1.15890411 | 0 | 10.48278184 | High groups |
| TCGA-86-8669-01 | 2.326027397 | 1 | 8.688658508 | Low groups |
| TCGA-86-8671-01 | 2.298630137 | 0 | 10.95242268 | High groups |
| TCGA-86-8672-01 | 0.052054795 | 0 | 10.00695857 | High groups |
| TCGA-86-8673-01 | 1.742465753 | 1 | 10.19245471 | High groups |
| TCGA-86-8674-01 | 0.915068493 | 1 | 10.75146598 | High groups |
| TCGA-86-A456-01 | 2.454794521 | 0 | 9.464260246 | Low groups |
| TCGA-86-A4D0-01 | 0.317808219 | 0 | 9.678043023 | High groups |
| TCGA-86-A4JF-01 | 1.194520548 | 1 | 9.740856071 | High groups |
| TCGA-86-A4P7-01 | 1.136986301 | 0 | 9.346767324 | Low groups |
| TCGA-86-A4P8-01 | 2.205479452 | 0 | 9.854248951 | High groups |
| TCGA-91-6828-01 | 0.884931507 | 0 | 8.407838653 | Low groups |
| TCGA-91-6829-01 | 3.446575342 | 0 | 9.871096979 | High groups |
| TCGA-91-6830-01 | 0.049315068 | 1 | 9.945008855 | High groups |
| TCGA-91-6831-01 | 0.849315068 | 0 | 9.498246878 | High groups |
| TCGA-91-6835-01 | 0.216438356 | 0 | 7.952404453 | Low groups |
| TCGA-91-6836-01 | 1.142465753 | 0 | 7.121285259 | Low groups |
| TCGA-91-6840-01 | 1.019178082 | 0 | 8.520069272 | Low groups |
| TCGA-91-6847-01 | 2.115068493 | 1 | 8.494744804 | Low groups |
| TCGA-91-6848-01 | 0.61369863 | 0 | 10.84704622 | High groups |
| TCGA-91-6849-01 | 0.095890411 | 0 | 9.458714396 | Low groups |
| TCGA-91-7771-01 | 1.347945205 | 0 | 8.032700757 | Low groups |
| TCGA-91-8496-01 | 1.383561644 | 0 | 9.606835829 | High groups |
| TCGA-91-8497-01 | 1.189041096 | 0 | 9.880622473 | High groups |
| TCGA-91-8499-01 | 0.098630137 | 0 | 8.395001 | Low groups |
| TCGA-91-A4BC-01 | 0.120547945 | 0 | 10.26042597 | High groups |
| TCGA-91-A4BD-01 | 1.652054795 | 0 | 8.441670627 | Low groups |
| TCGA-93-7347-01 | 1.871232877 | 0 | 10.50904368 | High groups |
| TCGA-93-7348-01 | 1.454794521 | 0 | 9.127071165 | Low groups |
| TCGA-93-8067-01 | 0.509589041 | 0 | 10.30867793 | High groups |
| TCGA-93-A4JN-01 | 1.967123288 | 0 | 10.19331208 | High groups |
| TCGA-93-A4JO-01 | 0.090410959 | 0 | 9.569806252 | High groups |
| TCGA-93-A4JP-01 | 1.361643836 | 1 | 9.4837758 | Low groups |
| TCGA-93-A4JQ-01 | 1.44109589 | 0 | 10.24580153 | High groups |
| TCGA-95-7039-01 | 3.446575342 | 1 | 10.09970811 | High groups |
| TCGA-95-7043-01 | 1.378082192 | 1 | 8.845781258 | Low groups |
| TCGA-95-7562-01 | 0.238356164 | 0 | 9.50315451 | High groups |
| TCGA-95-7567-01 | 1.556164384 | 0 | 8.49912412 | Low groups |
| TCGA-95-7944-01 | 1.032876712 | 0 | 8.985235138 | Low groups |
| TCGA-95-7947-01 | 1.306849315 | 0 | 8.898712627 | Low groups |
| TCGA-95-7948-01 | 1.304109589 | 0 | 9.374242252 | Low groups |
| TCGA-95-8039-01 | 0.624657534 | 1 | 9.855169199 | High groups |
| TCGA-95-8494-01 | 0.230136986 | 0 | 9.904387754 | High groups |
| TCGA-95-A4VK-01 | 1.35890411 | 1 | 10.22083235 | High groups |
| TCGA-95-A4VN-01 | 1.515068493 | 0 | 9.557447618 | High groups |
| TCGA-95-A4VP-01 | 0.591780822 | 1 | 10.18833601 | High groups |
| TCGA-97-7546-01 | 3.438356164 | 1 | 9.296093108 | Low groups |
| TCGA-97-7547-01 | 2.123287671 | 1 | 8.762919581 | Low groups |
| TCGA-97-7552-01 | 2.18630137 | 1 | 7.385856621 | Low groups |
| TCGA-97-7553-01 | 5.123287671 | 0 | 7.911771982 | Low groups |
| TCGA-97-7554-01 | 2.123287671 | 0 | 9.298068985 | Low groups |
| TCGA-97-7937-01 | 1.545205479 | 0 | 9.257934172 | Low groups |
| TCGA-97-7938-01 | 0.049315068 | 0 | 8.439284197 | Low groups |
| TCGA-97-7941-01 | 1.326027397 | 0 | 10.77875412 | High groups |
| TCGA-97-8171-01 | 1.208219178 | 1 | 9.056043446 | Low groups |
| TCGA-97-8172-01 | 1.493150685 | 0 | 9.978914395 | High groups |
| TCGA-97-8174-01 | 0.449315068 | 0 | 9.930860124 | High groups |
| TCGA-97-8175-01 | 0.805479452 | 1 | 9.509993074 | High groups |
| TCGA-97-8176-01 | 0.106849315 | 1 | 9.478058915 | Low groups |
| TCGA-97-8177-01 | 1.367123288 | 0 | 10.63255551 | High groups |
| TCGA-97-8179-01 | 1.191780822 | 0 | 10.67729584 | High groups |
| TCGA-97-8547-01 | 1.8 | 0 | 10.76732656 | High groups |
| TCGA-97-8552-01 | 1.715068493 | 0 | 9.929480285 | High groups |
| TCGA-97-A4LX-01 | 1.682191781 | 0 | 10.43917015 | High groups |
| TCGA-97-A4M0-01 | 1.78630137 | 0 | 8.922553363 | Low groups |
| TCGA-97-A4M1-01 | 1.646575342 | 0 | 9.944657456 | High groups |
| TCGA-97-A4M2-01 | 1.709589041 | 0 | 9.259412795 | Low groups |
| TCGA-97-A4M3-01 | 0.147945205 | 1 | 10.14587523 | High groups |
| TCGA-97-A4M5-01 | 1.736986301 | 0 | 10.42115417 | High groups |
| TCGA-97-A4M6-01 | 1.556164384 | 0 | 10.51108155 | High groups |
| TCGA-97-A4M7-01 | 1.723287671 | 0 | 9.893033083 | High groups |
| TCGA-99-7458-01 | 2.046575342 | 0 | 9.02936785 | Low groups |
| TCGA-99-8025-01 | 2.904109589 | 0 | 9.894875803 | High groups |
| TCGA-99-8028-01 | 3.063013699 | 0 | 10.5171619 | High groups |
| TCGA-99-8032-01 | 0.120547945 | 0 | 10.94118923 | High groups |
| TCGA-99-8033-01 | 1.797260274 | 1 | 10.27291748 | High groups |
| TCGA-99-AA5R-01 | 1.802739726 | 0 | 9.676725619 | High groups |
| TCGA-J2-8192-01 | 1.320547945 | 1 | 10.90335279 | High groups |
| TCGA-J2-8194-01 | 1.287671233 | 1 | 10.23928251 | High groups |
| TCGA-J2-A4AD-01 | 1.435616438 | 1 | 11.22857185 | High groups |
| TCGA-J2-A4AE-01 | 2.956164384 | 0 | 9.250481527 | Low groups |
| TCGA-J2-A4AG-01 | 2.706849315 | 0 | 10.15667976 | High groups |
| TCGA-L4-A4E5-01 | 1.583561644 | 0 | 9.172429009 | Low groups |
| TCGA-L4-A4E6-01 | 1.191780822 | 0 | 9.826781397 | High groups |
| TCGA-L9-A443-01 | 0.528767123 | 0 | 10.31387367 | High groups |
| TCGA-L9-A444-01 | 0.84109589 | 0 | 9.409477495 | Low groups |
| TCGA-L9-A50W-01 | 1.065753425 | 1 | 9.651651846 | High groups |
| TCGA-L9-A5IP-01 | 0.134246575 | 1 | 8.733059297 | Low groups |
| TCGA-L9-A743-01 | 1.819178082 | 0 | 10.54398888 | High groups |
| TCGA-L9-A7SV-01 | 1.547945205 | 0 | 8.838868748 | Low groups |
| TCGA-L9-A8F4-01 | 1.304109589 | 0 | 10.21756796 | High groups |
| TCGA-MN-A4N1-01 | 2.265753425 | 0 | 10.6041376 | High groups |
| TCGA-MN-A4N4-01 | 3.219178082 | 0 | 11.15656057 | High groups |
| TCGA-MN-A4N5-01 | 0.230136986 | 0 | 9.661078595 | High groups |
| TCGA-MP-A4SV-01 | 7.178082192 | 0 | 9.750989469 | High groups |
| TCGA-MP-A4SW-01 | 4.871232877 | 0 | 9.714763764 | High groups |
| TCGA-MP-A4SY-01 | 1.339726027 | 1 | 11.24033669 | High groups |
| TCGA-MP-A4T2-01 | 1.575342466 | 1 | 9.381709144 | Low groups |
| TCGA-MP-A4T4-01 | 7.169863014 | 0 | 10.25395997 | High groups |
| TCGA-MP-A4T6-01 | 4.904109589 | 0 | 9.00699502 | Low groups |
| TCGA-MP-A4T7-01 | 0.457534247 | 0 | 9.398045344 | Low groups |
| TCGA-MP-A4T8-01 | 0.44109589 | 0 | 10.49380282 | High groups |
| TCGA-MP-A4T9-01 | 0.934246575 | 1 | 9.855873557 | High groups |
| TCGA-MP-A4TA-01 | 1.98630137 | 1 | 9.977149316 | High groups |
| TCGA-MP-A4TC-01 | 0.202739726 | 0 | 11.02220016 | High groups |
| TCGA-MP-A4TD-01 | 0.619178082 | 1 | 11.64343391 | High groups |
| TCGA-MP-A4TE-01 | 0.619178082 | 1 | 7.9616845 | Low groups |
| TCGA-MP-A4TF-01 | 0.534246575 | 1 | 9.030315385 | Low groups |
| TCGA-MP-A4TH-01 | 2.030136986 | 0 | 10.65110965 | High groups |
| TCGA-MP-A4TI-01 | 0.230136986 | 1 | 9.607163895 | High groups |
| TCGA-MP-A4TJ-01 | 0.928767123 | 0 | 10.39074216 | High groups |
| TCGA-MP-A4TK-01 | 1.087671233 | 1 | 11.33852052 | High groups |
| TCGA-MP-A5C7-01 | 6.15890411 | 0 | 9.589298341 | High groups |
| TCGA-NJ-A4YF-01 | 5.920547945 | 0 | 10.18566613 | High groups |
| TCGA-NJ-A4YG-01 | 6.194520548 | 0 | 10.39725053 | High groups |
| TCGA-NJ-A4YI-01 | 0.010958904 | 0 | 10.96550647 | High groups |
| TCGA-NJ-A4YP-01 | 0.136986301 | 0 | 10.01824734 | High groups |
| TCGA-NJ-A4YQ-01 | 3.923287671 | 0 | 10.01442866 | High groups |
| TCGA-NJ-A55A-01 | 0.04109589 | 0 | 10.31778229 | High groups |
| TCGA-NJ-A55O-01 | 0.035616438 | 0 | 9.845099781 | High groups |
| TCGA-NJ-A55R-01 | 1.652054795 | 0 | 10.24280064 | High groups |
| TCGA-NJ-A7XG-01 | 1.690410959 | 0 | 8.973676418 | Low groups |
| TCGA-O1-A52J-01 | 2.460273973 | 1 | 9.803620727 | High groups |
| TCGA-S2-AA1A-01 | 1.405479452 | 0 | 10.66003468 | High groups |

**Table S8.** Raw counts of RNA-sequencing data of hsa-miR-328-3p in LUAD from the TCGA.

| sampleID | time | Staus | RS | Label |
| --- | --- | --- | --- | --- |
| TCGA-05-4244-01 | 0 | 0 | 3.84410038 | Low groups |
| TCGA-05-4249-01 | 4.17260274 | 0 | 2.088770518 | Low groups |
| TCGA-05-4250-01 | 0.331506849 | 0 | 3.793551914 | Low groups |
| TCGA-05-4382-01 | 0.915068493 | 1 | 4.986524253 | High groups |
| TCGA-05-4384-01 | 0.501369863 | 1 | 5.909590476 | High groups |
| TCGA-05-4389-01 | 3.750684932 | 0 | 4.927016634 | High groups |
| TCGA-05-4390-01 | 1.082191781 | 1 | 5.233424991 | High groups |
| TCGA-05-4395-01 | 0 | 0 | 4.94500697 | High groups |
| TCGA-05-4396-01 | 0.830136986 | 0 | 3.647000145 | Low groups |
| TCGA-05-4397-01 | 2.002739726 | 0 | 7.215613468 | High groups |
| TCGA-05-4398-01 | 3.920547945 | 0 | 4.286708191 | Low groups |
| TCGA-05-4402-01 | 0.668493151 | 0 | 5.044739145 | High groups |
| TCGA-05-4403-01 | 1.583561644 | 0 | 4.309423602 | Low groups |
| TCGA-05-4405-01 | 1.671232877 | 0 | 4.043111081 | Low groups |
| TCGA-05-4410-01 | 0 | 0 | 4.640075303 | Low groups |
| TCGA-05-4415-01 | 0.164383562 | 1 | 4.179081124 | Low groups |
| TCGA-05-4417-01 | 1.246575342 | 0 | 4.305957392 | Low groups |
| TCGA-05-4418-01 | 0.750684932 | 0 | 4.258649093 | Low groups |
| TCGA-05-4420-01 | 2.498630137 | 0 | 4.523108377 | Low groups |
| TCGA-05-4422-01 | 1 | 0 | 5.235295489 | High groups |
| TCGA-05-4424-01 | 0.419178082 | 1 | 5.535395236 | High groups |
| TCGA-05-4425-01 | 1.832876712 | 0 | 4.945975633 | High groups |
| TCGA-05-4426-01 | 1.252054795 | 1 | 4.93608921 | High groups |
| TCGA-05-4427-01 | 2.167123288 | 0 | 3.046730093 | Low groups |
| TCGA-05-4430-01 | 2.084931507 | 0 | 4.229959315 | Low groups |
| TCGA-05-4432-01 | 2.084931507 | 0 | 4.765586088 | Low groups |
| TCGA-05-4433-01 | 2 | 0 | 4.506199311 | Low groups |
| TCGA-05-4434-01 | 1.252054795 | 0 | 4.739990475 | Low groups |
| TCGA-05-5420-01 | 0.671232877 | 1 | 4.456446416 | Low groups |
| TCGA-05-5423-01 | 0.41369863 | 0 | 4.857125124 | High groups |
| TCGA-05-5425-01 | 1.331506849 | 1 | 4.819059405 | Low groups |
| TCGA-05-5428-01 | 1.835616438 | 0 | 3.158884301 | Low groups |
| TCGA-05-5429-01 | 0.753424658 | 0 | 3.636411289 | Low groups |
| TCGA-05-5715-01 | 0.169863014 | 0 | 5.031030859 | High groups |
| TCGA-35-3615-01 | 0.038356164 | 0 | 2.810961168 | Low groups |
| TCGA-35-4122-01 | 0.616438356 | 0 | 3.493807425 | Low groups |
| TCGA-35-4123-01 | 0.498630137 | 0 | 4.102066404 | Low groups |
| TCGA-35-5375-01 | 0.723287671 | 0 | 4.331775859 | Low groups |
| TCGA-38-4625-01 | 8.145205479 | 0 | 5.591635439 | High groups |
| TCGA-38-4626-01 | 6.898630137 | 1 | 6.013529189 | High groups |
| TCGA-38-4627-01 | 3.142465753 | 0 | 6.23222796 | High groups |
| TCGA-38-4628-01 | 2.967123288 | 1 | 5.327610126 | High groups |
| TCGA-38-4629-01 | 1.038356164 | 1 | 4.722097489 | Low groups |
| TCGA-38-4630-01 | 1.435616438 | 1 | 5.165388093 | High groups |
| TCGA-38-4631-01 | 0.969863014 | 1 | 3.896772781 | Low groups |
| TCGA-38-4632-01 | 1.863013699 | 1 | 4.73586743 | Low groups |
| TCGA-38-6178-01 | 1.22739726 | 0 | 4.815202924 | Low groups |
| TCGA-38-7271-01 | 0.832876712 | 1 | 3.916457536 | Low groups |
| TCGA-38-A44F-01 | 0.364383562 | 0 | 3.789584244 | Low groups |
| TCGA-44-2655-01 | 2.764383562 | 1 | 4.58422299 | Low groups |
| TCGA-44-2656-01 | 1.556164384 | 1 | 4.404876562 | Low groups |
| TCGA-44-2657-01 | 3.701369863 | 0 | 4.645370686 | Low groups |
| TCGA-44-2659-01 | 3.139726027 | 1 | 4.065475549 | Low groups |
| TCGA-44-2661-01 | 3.175342466 | 0 | 2.373937139 | Low groups |
| TCGA-44-2662-01 | 0.671232877 | 1 | 5.233629263 | High groups |
| TCGA-44-2665-01 | 3.564383562 | 0 | 4.720444672 | Low groups |
| TCGA-44-2666-01 | 0.265753425 | 1 | 3.233220076 | Low groups |
| TCGA-44-2668-01 | 1.18630137 | 1 | 4.443014866 | Low groups |
| TCGA-44-3396-01 | 3.095890411 | 0 | 5.462845975 | High groups |
| TCGA-44-3398-01 | 3.18630137 | 0 | 4.200167526 | Low groups |
| TCGA-44-3918-01 | 1.4 | 1 | 4.377084314 | Low groups |
| TCGA-44-3919-01 | 2.523287671 | 1 | 4.321935092 | Low groups |
| TCGA-44-4112-01 | 1.695890411 | 1 | 2.609541166 | Low groups |
| TCGA-44-5643-01 | 2.775342466 | 0 | 4.144988791 | Low groups |
| TCGA-44-5644-01 | 2.364383562 | 0 | 4.654800637 | Low groups |
| TCGA-44-5645-01 | 2.334246575 | 0 | 6.94608913 | High groups |
| TCGA-44-6144-01 | 1.093150685 | 1 | 5.828345969 | High groups |
| TCGA-44-6145-01 | 1.630136986 | 0 | 4.685368618 | Low groups |
| TCGA-44-6146-01 | 1.728767123 | 1 | 6.752085955 | High groups |
| TCGA-44-6147-01 | 2.315068493 | 0 | 6.985953478 | High groups |
| TCGA-44-6148-01 | 1.928767123 | 0 | 7.152235044 | High groups |
| TCGA-44-6774-01 | 1.802739726 | 0 | 4.275086557 | Low groups |
| TCGA-44-6775-01 | 1.873972603 | 1 | 7.72714398 | High groups |
| TCGA-44-6776-01 | 7.167123288 | 0 | 4.101606126 | Low groups |
| TCGA-44-6777-01 | 2.704109589 | 0 | 4.142843241 | Low groups |
| TCGA-44-6778-01 | 5.106849315 | 0 | 5.132087197 | High groups |
| TCGA-44-6779-01 | 0.635616438 | 1 | 3.626804437 | Low groups |
| TCGA-44-7659-01 | 1.893150685 | 0 | 4.311089706 | Low groups |
| TCGA-44-7660-01 | 0.693150685 | 1 | 4.389580303 | Low groups |
| TCGA-44-7661-01 | 0.917808219 | 1 | 3.312567597 | Low groups |
| TCGA-44-7662-01 | 0.597260274 | 0 | 3.737751216 | Low groups |
| TCGA-44-7667-01 | 3.005479452 | 0 | 5.050382106 | High groups |
| TCGA-44-7669-01 | 1.068493151 | 1 | 4.317504943 | Low groups |
| TCGA-44-7670-01 | 2.416438356 | 0 | 4.163247688 | Low groups |
| TCGA-44-7671-01 | 2.419178082 | 1 | 3.872635337 | Low groups |
| TCGA-44-7672-01 | 1.969863014 | 0 | 3.48418622 | Low groups |
| TCGA-44-8117-01 | 1.054794521 | 0 | 5.360945733 | High groups |
| TCGA-44-8119-01 | 0.780821918 | 0 | 5.614891907 | High groups |
| TCGA-44-8120-01 | 0.712328767 | 0 | 6.348854893 | High groups |
| TCGA-44-A479-01 | 1.197260274 | 1 | 4.855039378 | High groups |
| TCGA-44-A47A-01 | 1.087671233 | 1 | 5.630241202 | High groups |
| TCGA-44-A47B-01 | 0.78630137 | 0 | 3.368548506 | Low groups |
| TCGA-44-A47F-01 | 0.923287671 | 0 | 4.390112813 | Low groups |
| TCGA-44-A47G-01 | 0.961643836 | 0 | 3.995944887 | Low groups |
| TCGA-44-A4SS-01 | 1.136986301 | 0 | 4.909600508 | High groups |
| TCGA-44-A4SU-01 | 0.712328767 | 1 | 6.266850805 | High groups |
| TCGA-49-4486-01 | 5.602739726 | 1 | 4.943420655 | High groups |
| TCGA-49-4487-01 | 1.909589041 | 1 | 3.744899696 | Low groups |
| TCGA-49-4488-01 | 1.736986301 | 1 | 5.404391101 | High groups |
| TCGA-49-4490-01 | 1.054794521 | 1 | 4.542815083 | Low groups |
| TCGA-49-4494-01 | 2.961643836 | 1 | 4.411066476 | Low groups |
| TCGA-49-4501-01 | 1.493150685 | 1 | 5.71466947 | High groups |
| TCGA-49-4505-01 | 1.142465753 | 1 | 5.192800002 | High groups |
| TCGA-49-4506-01 | 1.895890411 | 1 | 3.509034888 | Low groups |
| TCGA-49-4507-01 | 0.432876712 | 1 | 3.964841816 | Low groups |
| TCGA-49-4510-01 | 1.375342466 | 1 | 4.397162915 | Low groups |
| TCGA-49-4512-01 | 2.479452055 | 1 | 5.192620755 | High groups |
| TCGA-49-4514-01 | 4.657534247 | 0 | 4.292539415 | Low groups |
| TCGA-49-6742-01 | 0.58630137 | 1 | 4.014962151 | Low groups |
| TCGA-49-6743-01 | 4.44109589 | 0 | 3.877462946 | Low groups |
| TCGA-49-6744-01 | 4.610958904 | 0 | 3.996546838 | Low groups |
| TCGA-49-6745-01 | 1.430136986 | 0 | 2.972826993 | Low groups |
| TCGA-49-6761-01 | 0.969863014 | 0 | 4.904844414 | High groups |
| TCGA-49-6767-01 | 1.854794521 | 0 | 3.753320633 | Low groups |
| TCGA-49-AAQV-01 | 1.394520548 | 1 | 4.892580507 | High groups |
| TCGA-49-AAR0-01 | 13.05479452 | 0 | 4.912651206 | High groups |
| TCGA-49-AAR2-01 | 6.093150685 | 0 | 5.524949224 | High groups |
| TCGA-49-AAR3-01 | 5.18630137 | 1 | 4.468783091 | Low groups |
| TCGA-49-AAR4-01 | 2.408219178 | 1 | 4.477828906 | Low groups |
| TCGA-49-AAR9-01 | 0.712328767 | 1 | 3.281117198 | Low groups |
| TCGA-49-AARE-01 | 1.063013699 | 1 | 4.499077499 | Low groups |
| TCGA-49-AARN-01 | 3.109589041 | 0 | 4.560245146 | Low groups |
| TCGA-49-AARO-01 | 3.134246575 | 1 | 4.879860024 | High groups |
| TCGA-49-AARQ-01 | 18.44383562 | 0 | 6.309798827 | High groups |
| TCGA-49-AARR-01 | 3.010958904 | 1 | 5.154873426 | High groups |
| TCGA-4B-A93V-01 | 0.687671233 | 1 | 5.281024217 | High groups |
| TCGA-50-5044-01 | 1.323287671 | 1 | 4.472102806 | Low groups |
| TCGA-50-5045-01 | 3.926027397 | 1 | 4.427245856 | Low groups |
| TCGA-50-5049-01 | 4.295890411 | 1 | 4.588993645 | Low groups |
| TCGA-50-5051-01 | 0.504109589 | 1 | 3.786719613 | Low groups |
| TCGA-50-5055-01 | 2.112328767 | 1 | 5.165391028 | High groups |
| TCGA-50-5066-01 | 1.306849315 | 1 | 4.204684599 | Low groups |
| TCGA-50-5068-01 | 1.879452055 | 1 | 4.387223145 | Low groups |
| TCGA-50-5072-01 | 0.583561644 | 1 | 4.070616964 | Low groups |
| TCGA-50-5930-01 | 0.484931507 | 1 | 4.338834558 | Low groups |
| TCGA-50-5931-01 | 1.164383562 | 1 | 6.59708626 | High groups |
| TCGA-50-5932-01 | 2.98630137 | 1 | 5.154996081 | High groups |
| TCGA-50-5933-01 | 6.556164384 | 0 | 3.952612013 | Low groups |
| TCGA-50-5935-01 | 1.789041096 | 0 | 5.767747003 | High groups |
| TCGA-50-5936-01 | 0.410958904 | 1 | 3.639158406 | Low groups |
| TCGA-50-5939-01 | 1.260273973 | 0 | 4.286087447 | Low groups |
| TCGA-50-5941-01 | 4.038356164 | 0 | 4.323546324 | Low groups |
| TCGA-50-5942-01 | 3.775342466 | 1 | 5.280071538 | High groups |
| TCGA-50-5944-01 | 4.794520548 | 0 | 4.596689446 | Low groups |
| TCGA-50-5946-01 | 0.605479452 | 1 | 5.64607191 | High groups |
| TCGA-50-6590-01 | 3.528767123 | 0 | 4.779153852 | Low groups |
| TCGA-50-6591-01 | 0.326027397 | 1 | 5.141183846 | High groups |
| TCGA-50-6592-01 | 2.128767123 | 1 | 4.454234469 | Low groups |
| TCGA-50-6593-01 | 0.728767123 | 1 | 4.127383097 | Low groups |
| TCGA-50-6594-01 | 0.780821918 | 1 | 6.258471249 | High groups |
| TCGA-50-6595-01 | 0.498630137 | 1 | 3.717015579 | Low groups |
| TCGA-50-6597-01 | 3.473972603 | 0 | 5.377338818 | High groups |
| TCGA-50-6673-01 | 0.060273973 | 0 | 6.453130186 | High groups |
| TCGA-50-7109-01 | 0.04109589 | 1 | 3.214309017 | Low groups |
| TCGA-50-8457-01 | 3.082191781 | 0 | 6.049649825 | High groups |
| TCGA-50-8459-01 | 1.183561644 | 1 | 4.258245661 | Low groups |
| TCGA-50-8460-01 | 2.271232877 | 0 | 5.157879932 | High groups |
| TCGA-53-7624-01 | 1.095890411 | 1 | 3.765686964 | Low groups |
| TCGA-53-7626-01 | 2.369863014 | 1 | 3.614383346 | Low groups |
| TCGA-53-7813-01 | 1.161643836 | 0 | 3.459473718 | Low groups |
| TCGA-53-A4EZ-01 | 2.934246575 | 0 | 4.526479521 | Low groups |
| TCGA-55-1592-01 | 1.238356164 | 1 | 5.418325405 | High groups |
| TCGA-55-1594-01 | 3.22739726 | 0 | 6.762786236 | High groups |
| TCGA-55-1595-01 | 4.052054795 | 0 | 5.133049713 | High groups |
| TCGA-55-1596-01 | 5.657534247 | 0 | 6.036898021 | High groups |
| TCGA-55-5899-01 | 2.547945205 | 0 | 5.825281792 | High groups |
| TCGA-55-6543-01 | 1.191780822 | 0 | 5.368307744 | High groups |
| TCGA-55-6642-01 | 6.709589041 | 0 | 3.988843261 | Low groups |
| TCGA-55-6712-01 | 0.468493151 | 1 | 3.289250926 | Low groups |
| TCGA-55-6968-01 | 3.542465753 | 1 | 5.99366972 | High groups |
| TCGA-55-6969-01 | 3.394520548 | 0 | 5.988810548 | High groups |
| TCGA-55-6970-01 | 1.252054795 | 1 | 5.512714425 | High groups |
| TCGA-55-6971-01 | 3.835616438 | 0 | 5.032974926 | High groups |
| TCGA-55-6972-01 | 4.471232877 | 0 | 6.580949087 | High groups |
| TCGA-55-6978-01 | 0.115068493 | 1 | 4.928840579 | High groups |
| TCGA-55-6979-01 | 0.534246575 | 1 | 6.170501684 | High groups |
| TCGA-55-6980-01 | 5.778082192 | 0 | 7.199560065 | High groups |
| TCGA-55-6981-01 | 3.778082192 | 0 | 6.013798194 | High groups |
| TCGA-55-6982-01 | 0.501369863 | 1 | 6.615679216 | High groups |
| TCGA-55-6983-01 | 7.734246575 | 0 | 5.254170726 | High groups |
| TCGA-55-6984-01 | 1.983561644 | 1 | 5.24804915 | High groups |
| TCGA-55-6985-01 | 3.378082192 | 0 | 5.625758515 | High groups |
| TCGA-55-6986-01 | 8.934246575 | 0 | 6.446293951 | High groups |
| TCGA-55-6987-01 | 5.854794521 | 0 | 4.879660191 | High groups |
| TCGA-55-7227-01 | 0.698630137 | 1 | 4.20432699 | Low groups |
| TCGA-55-7281-01 | 0.928767123 | 1 | 3.191387593 | Low groups |
| TCGA-55-7283-01 | 1.668493151 | 0 | 4.018343167 | Low groups |
| TCGA-55-7284-01 | 0.632876712 | 1 | 5.611551126 | High groups |
| TCGA-55-7570-01 | 2.257534247 | 0 | 3.810403925 | Low groups |
| TCGA-55-7573-01 | 1.334246575 | 0 | 4.764479655 | Low groups |
| TCGA-55-7574-01 | 1.309589041 | 1 | 3.778440104 | Low groups |
| TCGA-55-7576-01 | 1.835616438 | 0 | 3.866910055 | Low groups |
| TCGA-55-7724-01 | 1.931506849 | 0 | 4.350817292 | Low groups |
| TCGA-55-7725-01 | 1.210958904 | 0 | 3.658010348 | Low groups |
| TCGA-55-7726-01 | 1.78630137 | 0 | 3.432905572 | Low groups |
| TCGA-55-7727-01 | 0.326027397 | 0 | 4.044362729 | Low groups |
| TCGA-55-7728-01 | 1.928767123 | 0 | 4.635676277 | Low groups |
| TCGA-55-7815-01 | 1.276712329 | 1 | 2.643617491 | Low groups |
| TCGA-55-7816-01 | 1.282191781 | 1 | 4.244445128 | Low groups |
| TCGA-55-7903-01 | 1.553424658 | 0 | 4.325989978 | Low groups |
| TCGA-55-7907-01 | 0.805479452 | 1 | 4.980747107 | High groups |
| TCGA-55-7910-01 | 2.789041096 | 1 | 3.442865356 | Low groups |
| TCGA-55-7911-01 | 1.410958904 | 1 | 3.871996188 | Low groups |
| TCGA-55-7913-01 | 1.315068493 | 1 | 5.735609744 | High groups |
| TCGA-55-7914-01 | 0.512328767 | 1 | 4.564956899 | Low groups |
| TCGA-55-7994-01 | 1.652054795 | 0 | 6.461170166 | High groups |
| TCGA-55-7995-01 | 1.282191781 | 1 | 6.250295196 | High groups |
| TCGA-55-8085-01 | 2.476712329 | 0 | 6.39051241 | High groups |
| TCGA-55-8087-01 | 1.265753425 | 0 | 6.412923841 | High groups |
| TCGA-55-8089-01 | 1.923287671 | 0 | 6.638382622 | High groups |
| TCGA-55-8090-01 | 1.501369863 | 1 | 5.884046427 | High groups |
| TCGA-55-8091-01 | 1.643835616 | 0 | 4.814985115 | Low groups |
| TCGA-55-8092-01 | 0.347945205 | 1 | 6.201701584 | High groups |
| TCGA-55-8094-01 | 1.482191781 | 0 | 6.487399442 | High groups |
| TCGA-55-8096-01 | 1.550684932 | 1 | 7.334808693 | High groups |
| TCGA-55-8097-01 | 1.304109589 | 0 | 6.785802792 | High groups |
| TCGA-55-8203-01 | 1.498630137 | 0 | 5.931222451 | High groups |
| TCGA-55-8204-01 | 1.410958904 | 0 | 4.331952666 | Low groups |
| TCGA-55-8205-01 | 1.356164384 | 1 | 5.957755049 | High groups |
| TCGA-55-8206-01 | 2.432876712 | 0 | 5.953167675 | High groups |
| TCGA-55-8207-01 | 2.676712329 | 0 | 5.688013303 | High groups |
| TCGA-55-8208-01 | 1.4 | 1 | 5.815750632 | High groups |
| TCGA-55-8299-01 | 0.750684932 | 1 | 6.056180965 | High groups |
| TCGA-55-8301-01 | 0.652054795 | 1 | 5.96783434 | High groups |
| TCGA-55-8302-01 | 1.309589041 | 0 | 4.361058556 | Low groups |
| TCGA-55-8505-01 | 1.205479452 | 0 | 4.975949649 | High groups |
| TCGA-55-8506-01 | 0.030136986 | 0 | 4.281652187 | Low groups |
| TCGA-55-8507-01 | 1.145205479 | 0 | 4.763132676 | Low groups |
| TCGA-55-8508-01 | 1.690410959 | 0 | 4.9307599 | High groups |
| TCGA-55-8510-01 | 1.476712329 | 0 | 6.404878707 | High groups |
| TCGA-55-8511-01 | 1.260273973 | 1 | 5.886000253 | High groups |
| TCGA-55-8512-01 | 1.663013699 | 1 | 5.627490461 | High groups |
| TCGA-55-8513-01 | 0.868493151 | 1 | 6.207420268 | High groups |
| TCGA-55-8514-01 | 1.424657534 | 0 | 6.036254071 | High groups |
| TCGA-55-8614-01 | 1.468493151 | 0 | 5.537791792 | High groups |
| TCGA-55-8615-01 | 0.61369863 | 1 | 5.56141825 | High groups |
| TCGA-55-8616-01 | 0.131506849 | 0 | 5.47649251 | High groups |
| TCGA-55-8619-01 | 1.139726027 | 0 | 5.434668256 | High groups |
| TCGA-55-8620-01 | 1.02739726 | 1 | 4.427357367 | Low groups |
| TCGA-55-8621-01 | 1.410958904 | 0 | 5.281718833 | High groups |
| TCGA-55-A48X-01 | 1.684931507 | 1 | 5.054643386 | High groups |
| TCGA-55-A48Y-01 | 1.726027397 | 0 | 3.709168752 | Low groups |
| TCGA-55-A48Z-01 | 1.468493151 | 1 | 4.512309008 | Low groups |
| TCGA-55-A490-01 | 0.271232877 | 0 | 4.735194664 | Low groups |
| TCGA-55-A491-01 | 1.715068493 | 0 | 4.290976147 | Low groups |
| TCGA-55-A492-01 | 1.632876712 | 0 | 4.63171721 | Low groups |
| TCGA-55-A493-01 | 0.076712329 | 0 | 4.119580118 | Low groups |
| TCGA-55-A494-01 | 1.317808219 | 0 | 6.190413967 | High groups |
| TCGA-55-A4DF-01 | 1.410958904 | 1 | 5.718410263 | High groups |
| TCGA-55-A4DG-01 | 1.665753425 | 0 | 5.843841929 | High groups |
| TCGA-55-A57B-01 | 1.495890411 | 0 | 5.857053094 | High groups |
| TCGA-62-8394-01 | 0.380821918 | 0 | 5.77669753 | High groups |
| TCGA-62-8395-01 | 1.082191781 | 1 | 5.399002097 | High groups |
| TCGA-62-8397-01 | 3.531506849 | 0 | 4.486804973 | Low groups |
| TCGA-62-8398-01 | 1.216438356 | 0 | 5.368750368 | High groups |
| TCGA-62-8399-01 | 7.38630137 | 0 | 5.658225767 | High groups |
| TCGA-62-8402-01 | 2.115068493 | 1 | 5.256821592 | High groups |
| TCGA-62-A46O-01 | 2.583561644 | 1 | 3.236655324 | Low groups |
| TCGA-62-A46P-01 | 0.731506849 | 1 | 3.126198124 | Low groups |
| TCGA-62-A46R-01 | 4.726027397 | 0 | 4.403514105 | Low groups |
| TCGA-62-A46S-01 | 1.443835616 | 1 | 4.882165267 | High groups |
| TCGA-62-A46U-01 | 5.663013699 | 0 | 4.500791539 | Low groups |
| TCGA-62-A46V-01 | 6.024657534 | 0 | 4.15898947 | Low groups |
| TCGA-62-A46Y-01 | 0.860273973 | 1 | 6.888788781 | High groups |
| TCGA-62-A470-01 | 1.476712329 | 1 | 4.668069869 | Low groups |
| TCGA-62-A471-01 | 3.41369863 | 0 | 3.415170221 | Low groups |
| TCGA-62-A472-01 | 0.794520548 | 1 | 3.832195126 | Low groups |
| TCGA-64-1676-01 | 4.734246575 | 0 | 4.378352918 | Low groups |
| TCGA-64-1677-01 | 0.98630137 | 1 | 4.25640708 | Low groups |
| TCGA-64-1678-01 | 3.257534247 | 0 | 4.108260733 | Low groups |
| TCGA-64-1679-01 | 6.816438356 | 0 | 4.245021735 | Low groups |
| TCGA-64-1680-01 | 3.084931507 | 0 | 2.854338621 | Low groups |
| TCGA-64-1681-01 | 1.202739726 | 1 | 4.770245561 | Low groups |
| TCGA-64-5774-01 | 0.673972603 | 1 | 4.31507107 | Low groups |
| TCGA-64-5775-01 | 0.169863014 | 1 | 4.125913897 | Low groups |
| TCGA-64-5778-01 | 2.635616438 | 1 | 4.377927075 | Low groups |
| TCGA-64-5779-01 | 2.178082192 | 1 | 5.950137681 | High groups |
| TCGA-64-5781-01 | 0.263013699 | 1 | 4.583853845 | Low groups |
| TCGA-64-5815-01 | 2.37260274 | 0 | 3.992808847 | Low groups |
| TCGA-67-3770-01 | 1.671232877 | 0 | 5.24682134 | High groups |
| TCGA-67-3771-01 | 1.671232877 | 0 | 4.322649983 | Low groups |
| TCGA-67-3772-01 | 1.569863014 | 0 | 4.528098052 | Low groups |
| TCGA-67-3773-01 | 1.169863014 | 0 | 3.602615674 | Low groups |
| TCGA-67-3774-01 | 1.054794521 | 0 | 5.742392926 | High groups |
| TCGA-67-4679-01 | 1.22739726 | 0 | 4.354563589 | Low groups |
| TCGA-67-6215-01 | 0.476712329 | 0 | 5.023921979 | High groups |
| TCGA-67-6216-01 | 0.38630137 | 0 | 3.940294661 | Low groups |
| TCGA-67-6217-01 | 0.810958904 | 1 | 6.322715952 | High groups |
| TCGA-69-7760-01 | 0.553424658 | 0 | 4.684770329 | Low groups |
| TCGA-69-7761-01 | 0.509589041 | 0 | 3.432389029 | Low groups |
| TCGA-69-7763-01 | 1.890410959 | 0 | 3.362198578 | Low groups |
| TCGA-69-7764-01 | 1.134246575 | 0 | 3.969114195 | Low groups |
| TCGA-69-7765-01 | 0.452054795 | 0 | 4.800865844 | Low groups |
| TCGA-69-7973-01 | 0.630136986 | 0 | 4.922861564 | High groups |
| TCGA-69-7974-01 | 0.504109589 | 0 | 5.186291364 | High groups |
| TCGA-69-7978-01 | 0.367123288 | 0 | 5.287043737 | High groups |
| TCGA-69-7979-01 | 1.117808219 | 0 | 5.330863112 | High groups |
| TCGA-69-7980-01 | 1.126027397 | 0 | 5.679982886 | High groups |
| TCGA-69-8253-01 | 1.167123288 | 0 | 5.78248117 | High groups |
| TCGA-69-8254-01 | 1.120547945 | 0 | 7.135012315 | High groups |
| TCGA-69-8255-01 | 0.353424658 | 0 | 5.620086571 | High groups |
| TCGA-69-8453-01 | 0.942465753 | 1 | 5.697247166 | High groups |
| TCGA-69-A59K-01 | 1.619178082 | 0 | 6.027646399 | High groups |
| TCGA-71-6725-01 | 0.449315068 | 1 | 5.012884932 | High groups |
| TCGA-71-8520-01 | 0.490410959 | 1 | 6.267222281 | High groups |
| TCGA-73-4658-01 | 4.383561644 | 0 | 3.723377258 | Low groups |
| TCGA-73-4659-01 | 0.095890411 | 1 | 5.715293111 | High groups |
| TCGA-73-4662-01 | 0.057534247 | 1 | 5.39997659 | High groups |
| TCGA-73-4675-01 | 0.989041096 | 1 | 5.304483772 | High groups |
| TCGA-73-4676-01 | 0.769863014 | 1 | 3.434953623 | Low groups |
| TCGA-73-7498-01 | 3.257534247 | 0 | 6.367386268 | High groups |
| TCGA-73-7499-01 | 3.964383562 | 1 | 4.810635485 | Low groups |
| TCGA-73-A9RS-01 | 0.293150685 | 1 | 4.477966356 | Low groups |
| TCGA-75-5125-01 | 4.8 | 1 | 4.992949074 | High groups |
| TCGA-75-5146-01 | 4.857534247 | 1 | 4.141200198 | Low groups |
| TCGA-75-5147-01 | 3.652054795 | 0 | 5.66588344 | High groups |
| TCGA-75-6206-01 | 7.095890411 | 0 | 6.048715725 | High groups |
| TCGA-75-6212-01 | 3.671232877 | 1 | 5.445008156 | High groups |
| TCGA-75-6214-01 | 1.147945205 | 1 | 5.695877341 | High groups |
| TCGA-75-7025-01 | 4.057534247 | 1 | 6.952638755 | High groups |
| TCGA-75-7027-01 | 8.079452055 | 1 | 4.98088778 | High groups |
| TCGA-78-7143-01 | 4.109589041 | 1 | 4.040479284 | Low groups |
| TCGA-78-7145-01 | 1.156164384 | 1 | 4.212943271 | Low groups |
| TCGA-78-7146-01 | 0.473972603 | 0 | 4.490640852 | Low groups |
| TCGA-78-7147-01 | 1.583561644 | 1 | 4.819809687 | Low groups |
| TCGA-78-7148-01 | 0.498630137 | 1 | 3.804304935 | Low groups |
| TCGA-78-7149-01 | 10.79452055 | 0 | 4.956747205 | High groups |
| TCGA-78-7150-01 | 0.380821918 | 1 | 2.989864461 | Low groups |
| TCGA-78-7152-01 | 3.293150685 | 1 | 3.388978876 | Low groups |
| TCGA-78-7153-01 | 9.95890411 | 0 | 4.180694816 | Low groups |
| TCGA-78-7154-01 | 1.624657534 | 0 | 3.961603975 | Low groups |
| TCGA-78-7155-01 | 0.57260274 | 1 | 3.114622456 | Low groups |
| TCGA-78-7156-01 | 2.673972603 | 0 | 5.142711014 | High groups |
| TCGA-78-7158-01 | 0.361643836 | 1 | 4.102362508 | Low groups |
| TCGA-78-7159-01 | 5.408219178 | 0 | 4.371533911 | Low groups |
| TCGA-78-7160-01 | 1.909589041 | 0 | 4.222928875 | Low groups |
| TCGA-78-7161-01 | 0.443835616 | 1 | 5.532136642 | High groups |
| TCGA-78-7162-01 | 6.076712329 | 1 | 5.047815367 | High groups |
| TCGA-78-7163-01 | 19.85753425 | 0 | 6.346979753 | High groups |
| TCGA-78-7166-01 | 0.706849315 | 0 | 2.976711527 | Low groups |
| TCGA-78-7167-01 | 1.989041096 | 1 | 4.875637064 | High groups |
| TCGA-78-7220-01 | 1.454794521 | 1 | 2.353796959 | Low groups |
| TCGA-78-7535-01 | 2.216438356 | 1 | 4.976635696 | High groups |
| TCGA-78-7536-01 | 0.632876712 | 1 | 4.601399034 | Low groups |
| TCGA-78-7537-01 | 4.443835616 | 0 | 3.772224139 | Low groups |
| TCGA-78-7539-01 | 1.884931507 | 1 | 4.02484951 | Low groups |
| TCGA-78-7540-01 | 3.279452055 | 0 | 3.95000455 | Low groups |
| TCGA-78-7542-01 | 0.879452055 | 0 | 4.341543694 | Low groups |
| TCGA-78-7633-01 | 3.97260274 | 1 | 4.550732197 | Low groups |
| TCGA-78-8640-01 | 19.34794521 | 0 | 5.694930046 | High groups |
| TCGA-78-8648-01 | 1.97260274 | 1 | 5.895957503 | High groups |
| TCGA-78-8655-01 | 6.465753425 | 0 | 4.524584966 | Low groups |
| TCGA-78-8660-01 | 0.663013699 | 1 | 5.052066482 | High groups |
| TCGA-78-8662-01 | 8.339726027 | 1 | 5.586964263 | High groups |
| TCGA-80-5608-01 | 7.75890411 | 0 | 5.631055231 | High groups |
| TCGA-80-5611-01 | 7.109589041 | 0 | 5.360505097 | High groups |
| TCGA-83-5908-01 | 2.257534247 | 0 | 5.843970783 | High groups |
| TCGA-86-6562-01 | 0.750684932 | 1 | 4.676727375 | Low groups |
| TCGA-86-6851-01 | 0.490410959 | 0 | 6.560605143 | High groups |
| TCGA-86-7701-01 | 1.161643836 | 1 | 3.775809627 | Low groups |
| TCGA-86-7711-01 | 2.865753425 | 1 | 4.056100526 | Low groups |
| TCGA-86-7713-01 | 3.169863014 | 0 | 4.347323153 | Low groups |
| TCGA-86-7714-01 | 1.712328767 | 1 | 3.906193124 | Low groups |
| TCGA-86-7953-01 | 2.731506849 | 0 | 5.531570044 | High groups |
| TCGA-86-7954-01 | 1.657534247 | 0 | 6.11734035 | High groups |
| TCGA-86-7955-01 | 2.246575342 | 1 | 6.481491409 | High groups |
| TCGA-86-8054-01 | 3.145205479 | 0 | 5.54212879 | High groups |
| TCGA-86-8055-01 | 0.339726027 | 0 | 6.850646426 | High groups |
| TCGA-86-8056-01 | 0.380821918 | 0 | 5.954027924 | High groups |
| TCGA-86-8073-01 | 2.02739726 | 0 | 6.386085451 | High groups |
| TCGA-86-8074-01 | 0.065753425 | 0 | 6.637204482 | High groups |
| TCGA-86-8075-01 | 0.545205479 | 1 | 5.647680318 | High groups |
| TCGA-86-8076-01 | 2.720547945 | 0 | 4.600279865 | Low groups |
| TCGA-86-8278-01 | 0.079452055 | 1 | 5.601918341 | High groups |
| TCGA-86-8279-01 | 2.6 | 0 | 7.997087439 | High groups |
| TCGA-86-8280-01 | 1.920547945 | 0 | 6.904855562 | High groups |
| TCGA-86-8281-01 | 0 | 0 | 5.903354717 | High groups |
| TCGA-86-8358-01 | 1.789041096 | 0 | 5.391842492 | High groups |
| TCGA-86-8359-01 | 1.216438356 | 0 | 3.488083447 | Low groups |
| TCGA-86-8585-01 | 0.967123288 | 0 | 5.490270714 | High groups |
| TCGA-86-8668-01 | 1.15890411 | 0 | 6.212741495 | High groups |
| TCGA-86-8669-01 | 2.326027397 | 1 | 5.437077561 | High groups |
| TCGA-86-8671-01 | 2.298630137 | 0 | 6.222293555 | High groups |
| TCGA-86-8672-01 | 0.052054795 | 0 | 3.614044167 | Low groups |
| TCGA-86-8673-01 | 1.742465753 | 1 | 5.664942427 | High groups |
| TCGA-86-8674-01 | 0.915068493 | 1 | 4.417518783 | Low groups |
| TCGA-86-A456-01 | 2.454794521 | 0 | 4.805109166 | Low groups |
| TCGA-86-A4D0-01 | 0.317808219 | 0 | 4.798345713 | Low groups |
| TCGA-86-A4JF-01 | 1.194520548 | 1 | 4.851872246 | High groups |
| TCGA-86-A4P7-01 | 1.136986301 | 0 | 4.472852688 | Low groups |
| TCGA-86-A4P8-01 | 2.205479452 | 0 | 5.70341559 | High groups |
| TCGA-91-6828-01 | 0.884931507 | 0 | 5.590564895 | High groups |
| TCGA-91-6829-01 | 3.446575342 | 0 | 4.126050647 | Low groups |
| TCGA-91-6830-01 | 0.049315068 | 1 | 6.244256884 | High groups |
| TCGA-91-6831-01 | 0.849315068 | 0 | 4.073430979 | Low groups |
| TCGA-91-6835-01 | 0.216438356 | 0 | 4.244957062 | Low groups |
| TCGA-91-6836-01 | 1.142465753 | 0 | 4.700168965 | Low groups |
| TCGA-91-6840-01 | 1.019178082 | 0 | 7.373122329 | High groups |
| TCGA-91-6847-01 | 2.115068493 | 1 | 5.335884663 | High groups |
| TCGA-91-6848-01 | 0.61369863 | 0 | 5.800102898 | High groups |
| TCGA-91-6849-01 | 0.095890411 | 0 | 5.812895554 | High groups |
| TCGA-91-7771-01 | 1.347945205 | 0 | 3.491937732 | Low groups |
| TCGA-91-8496-01 | 1.383561644 | 0 | 5.307882757 | High groups |
| TCGA-91-8497-01 | 1.189041096 | 0 | 5.155575199 | High groups |
| TCGA-91-8499-01 | 0.098630137 | 0 | 5.146003859 | High groups |
| TCGA-91-A4BC-01 | 0.120547945 | 0 | 3.278902575 | Low groups |
| TCGA-91-A4BD-01 | 1.652054795 | 0 | 3.489576845 | Low groups |
| TCGA-93-7347-01 | 1.871232877 | 0 | 5.831500308 | High groups |
| TCGA-93-7348-01 | 1.454794521 | 0 | 3.85909711 | Low groups |
| TCGA-93-8067-01 | 0.509589041 | 0 | 5.915282583 | High groups |
| TCGA-93-A4JN-01 | 1.967123288 | 0 | 3.90395625 | Low groups |
| TCGA-93-A4JO-01 | 0.090410959 | 0 | 4.033853235 | Low groups |
| TCGA-93-A4JP-01 | 1.361643836 | 1 | 5.850247508 | High groups |
| TCGA-93-A4JQ-01 | 1.44109589 | 0 | 4.468995996 | Low groups |
| TCGA-95-7039-01 | 3.446575342 | 1 | 5.44185526 | High groups |
| TCGA-95-7043-01 | 1.378082192 | 1 | 6.507053126 | High groups |
| TCGA-95-7562-01 | 0.238356164 | 0 | 5.127230457 | High groups |
| TCGA-95-7567-01 | 1.556164384 | 0 | 3.551306089 | Low groups |
| TCGA-95-7944-01 | 1.032876712 | 0 | 4.484419616 | Low groups |
| TCGA-95-7947-01 | 1.306849315 | 0 | 5.377067165 | High groups |
| TCGA-95-7948-01 | 1.304109589 | 0 | 5.608375423 | High groups |
| TCGA-95-8039-01 | 0.624657534 | 1 | 4.754432768 | Low groups |
| TCGA-95-8494-01 | 0.230136986 | 0 | 4.779629068 | Low groups |
| TCGA-95-A4VK-01 | 1.35890411 | 1 | 4.498954361 | Low groups |
| TCGA-95-A4VN-01 | 1.515068493 | 0 | 4.623267858 | Low groups |
| TCGA-95-A4VP-01 | 0.591780822 | 1 | 4.36767509 | Low groups |
| TCGA-97-7546-01 | 3.438356164 | 1 | 4.896942464 | High groups |
| TCGA-97-7547-01 | 2.123287671 | 1 | 4.184114272 | Low groups |
| TCGA-97-7552-01 | 2.18630137 | 1 | 3.392904274 | Low groups |
| TCGA-97-7553-01 | 5.123287671 | 0 | 3.154767244 | Low groups |
| TCGA-97-7554-01 | 2.123287671 | 0 | 4.827975173 | High groups |
| TCGA-97-7937-01 | 1.545205479 | 0 | 3.930662883 | Low groups |
| TCGA-97-7938-01 | 0.049315068 | 0 | 2.759686145 | Low groups |
| TCGA-97-7941-01 | 1.326027397 | 0 | 6.372742328 | High groups |
| TCGA-97-8171-01 | 1.208219178 | 1 | 5.746188917 | High groups |
| TCGA-97-8172-01 | 1.493150685 | 0 | 5.990503653 | High groups |
| TCGA-97-8174-01 | 0.449315068 | 0 | 6.20909604 | High groups |
| TCGA-97-8175-01 | 0.805479452 | 1 | 5.980683323 | High groups |
| TCGA-97-8176-01 | 0.106849315 | 1 | 4.381208397 | Low groups |
| TCGA-97-8177-01 | 1.367123288 | 0 | 4.456810879 | Low groups |
| TCGA-97-8179-01 | 1.191780822 | 0 | 6.693539925 | High groups |
| TCGA-97-8547-01 | 1.8 | 0 | 5.632138774 | High groups |
| TCGA-97-8552-01 | 1.715068493 | 0 | 5.485996322 | High groups |
| TCGA-97-A4LX-01 | 1.682191781 | 0 | 5.336774463 | High groups |
| TCGA-97-A4M0-01 | 1.78630137 | 0 | 3.870178267 | Low groups |
| TCGA-97-A4M1-01 | 1.646575342 | 0 | 5.239323186 | High groups |
| TCGA-97-A4M2-01 | 1.709589041 | 0 | 5.034960205 | High groups |
| TCGA-97-A4M3-01 | 0.147945205 | 1 | 4.437635104 | Low groups |
| TCGA-97-A4M5-01 | 1.736986301 | 0 | 4.035163581 | Low groups |
| TCGA-97-A4M6-01 | 1.556164384 | 0 | 5.39925861 | High groups |
| TCGA-97-A4M7-01 | 1.723287671 | 0 | 4.686944361 | Low groups |
| TCGA-99-7458-01 | 2.046575342 | 0 | 4.281788147 | Low groups |
| TCGA-99-8025-01 | 2.904109589 | 0 | 5.025407585 | High groups |
| TCGA-99-8028-01 | 3.063013699 | 0 | 5.065397392 | High groups |
| TCGA-99-8032-01 | 0.120547945 | 0 | 6.207920663 | High groups |
| TCGA-99-8033-01 | 1.797260274 | 1 | 5.897437693 | High groups |
| TCGA-99-AA5R-01 | 1.802739726 | 0 | 5.017008936 | High groups |
| TCGA-J2-8192-01 | 1.320547945 | 1 | 6.613742325 | High groups |
| TCGA-J2-8194-01 | 1.287671233 | 1 | 6.329661008 | High groups |
| TCGA-J2-A4AD-01 | 1.435616438 | 1 | 5.982200958 | High groups |
| TCGA-J2-A4AE-01 | 2.956164384 | 0 | 5.098921688 | High groups |
| TCGA-J2-A4AG-01 | 2.706849315 | 0 | 4.599921945 | Low groups |
| TCGA-L4-A4E5-01 | 1.583561644 | 0 | 5.151713534 | High groups |
| TCGA-L4-A4E6-01 | 1.191780822 | 0 | 5.065485802 | High groups |
| TCGA-L9-A443-01 | 0.528767123 | 0 | 3.95282086 | Low groups |
| TCGA-L9-A444-01 | 0.84109589 | 0 | 4.852660936 | High groups |
| TCGA-L9-A50W-01 | 1.065753425 | 1 | 4.185469195 | Low groups |
| TCGA-L9-A5IP-01 | 0.134246575 | 1 | 4.379634061 | Low groups |
| TCGA-L9-A743-01 | 1.819178082 | 0 | 4.017261858 | Low groups |
| TCGA-L9-A7SV-01 | 1.547945205 | 0 | 5.565163571 | High groups |
| TCGA-L9-A8F4-01 | 1.304109589 | 0 | 5.422909778 | High groups |
| TCGA-MN-A4N1-01 | 2.265753425 | 0 | 4.676487452 | Low groups |
| TCGA-MN-A4N4-01 | 3.219178082 | 0 | 4.469684141 | Low groups |
| TCGA-MN-A4N5-01 | 0.230136986 | 0 | 5.329398364 | High groups |
| TCGA-MP-A4SV-01 | 7.178082192 | 0 | 4.609567463 | Low groups |
| TCGA-MP-A4SW-01 | 4.871232877 | 0 | 5.038243891 | High groups |
| TCGA-MP-A4SY-01 | 1.339726027 | 1 | 4.950607778 | High groups |
| TCGA-MP-A4T2-01 | 1.575342466 | 1 | 5.448325046 | High groups |
| TCGA-MP-A4T4-01 | 7.169863014 | 0 | 4.05846959 | Low groups |
| TCGA-MP-A4T6-01 | 4.904109589 | 0 | 7.322637005 | High groups |
| TCGA-MP-A4T7-01 | 0.457534247 | 0 | 5.144697394 | High groups |
| TCGA-MP-A4T8-01 | 0.44109589 | 0 | 4.625468759 | Low groups |
| TCGA-MP-A4T9-01 | 0.934246575 | 1 | 5.099160272 | High groups |
| TCGA-MP-A4TA-01 | 1.98630137 | 1 | 4.65168892 | Low groups |
| TCGA-MP-A4TC-01 | 0.202739726 | 0 | 4.267129974 | Low groups |
| TCGA-MP-A4TD-01 | 0.619178082 | 1 | 5.119051087 | High groups |
| TCGA-MP-A4TE-01 | 0.619178082 | 1 | 4.692060452 | Low groups |
| TCGA-MP-A4TF-01 | 0.534246575 | 1 | 5.718705119 | High groups |
| TCGA-MP-A4TH-01 | 2.030136986 | 0 | 5.409868033 | High groups |
| TCGA-MP-A4TI-01 | 0.230136986 | 1 | 3.57831961 | Low groups |
| TCGA-MP-A4TJ-01 | 0.928767123 | 0 | 4.965298395 | High groups |
| TCGA-MP-A4TK-01 | 1.087671233 | 1 | 6.244105578 | High groups |
| TCGA-MP-A5C7-01 | 6.15890411 | 0 | 5.858054123 | High groups |
| TCGA-NJ-A4YF-01 | 5.920547945 | 0 | 5.8775866 | High groups |
| TCGA-NJ-A4YG-01 | 6.194520548 | 0 | 3.834607874 | Low groups |
| TCGA-NJ-A4YI-01 | 0.010958904 | 0 | 4.825718985 | High groups |
| TCGA-NJ-A4YP-01 | 0.136986301 | 0 | 3.721988654 | Low groups |
| TCGA-NJ-A4YQ-01 | 3.923287671 | 0 | 4.538122367 | Low groups |
| TCGA-NJ-A55A-01 | 0.04109589 | 0 | 5.360424329 | High groups |
| TCGA-NJ-A55O-01 | 0.035616438 | 0 | 4.5411869 | Low groups |
| TCGA-NJ-A55R-01 | 1.652054795 | 0 | 5.465981841 | High groups |
| TCGA-NJ-A7XG-01 | 1.690410959 | 0 | 3.473517241 | Low groups |
| TCGA-O1-A52J-01 | 2.460273973 | 1 | 6.880015442 | High groups |
| TCGA-S2-AA1A-01 | 1.405479452 | 0 | 4.269215417 | Low groups |

**Table S9.** Raw counts of RNA-sequencing data of *KLHL3* in LUAD from the TCGA.

| sampleID | time | Staus | RS | Label |
| --- | --- | --- | --- | --- |
| TCGA-44-7660-01 | 0.693150685 | 1 | 1.549873753 | High groups |
| TCGA-78-8662-01 | 8.339726027 | 1 | 0.634654755 | Low groups |
| TCGA-75-7027-01 | 8.079452055 | 1 | 2.493548414 | High groups |
| TCGA-97-A4M6-01 | 1.556164384 | 0 | 2.070069163 | High groups |
| TCGA-MP-A4SV-01 | 7.178082192 | 0 | 2.395726264 | High groups |
| TCGA-38-4627-01 | 3.142465753 | 0 | 2.012155058 | High groups |
| TCGA-93-A4JO-01 | 0.090410959 | 0 | 1.222524442 | Low groups |
| TCGA-05-4426-01 | 1.252054795 | 1 | 1.01313197 | Low groups |
| TCGA-97-7937-01 | 1.545205479 | 0 | 0.793487179 | Low groups |
| TCGA-75-6206-01 | 7.095890411 | 0 | 1.885374372 | High groups |
| TCGA-62-A472-01 | 0.794520548 | 1 | 2.313302779 | High groups |
| TCGA-49-6743-01 | 4.44109589 | 0 | 0.970748179 | Low groups |
| TCGA-50-6597-01 | 3.473972603 | 0 | 0.572452621 | Low groups |
| TCGA-55-A490-01 | 0.271232877 | 0 | 1.073449306 | Low groups |
| TCGA-99-8032-01 | 0.120547945 | 0 | 1.487992827 | Low groups |
| TCGA-55-8299-01 | 0.750684932 | 1 | 1.713591464 | High groups |
| TCGA-64-1676-01 | 4.734246575 | 0 | 0.389058752 | Low groups |
| TCGA-86-8279-01 | 2.6 | 0 | 1.516438279 | High groups |
| TCGA-97-A4M5-01 | 1.736986301 | 0 | 1.813877838 | High groups |
| TCGA-78-7536-01 | 0.632876712 | 1 | 0.358534319 | Low groups |
| TCGA-55-8208-01 | 1.4 | 1 | 1.659731503 | High groups |
| TCGA-95-7562-01 | 0.238356164 | 0 | 1.002878059 | Low groups |
| TCGA-MP-A4TK-01 | 1.087671233 | 1 | 2.01962513 | High groups |
| TCGA-49-6761-01 | 0.969863014 | 0 | 0.812834803 | Low groups |
| TCGA-50-6593-01 | 0.728767123 | 1 | 1.300505181 | Low groups |
| TCGA-55-7728-01 | 1.928767123 | 0 | 3.244334115 | High groups |
| TCGA-86-8055-01 | 0.339726027 | 0 | 1.815209519 | High groups |
| TCGA-73-4668-01 | 0.646575342 | 1 | 0.710447849 | Low groups |
| TCGA-55-8090-01 | 1.501369863 | 1 | 0.874471658 | Low groups |
| TCGA-38-7271-01 | 0.832876712 | 1 | 1.343204868 | Low groups |
| TCGA-55-8514-01 | 1.424657534 | 0 | 0.720889221 | Low groups |
| TCGA-50-5932-01 | 2.98630137 | 1 | 0.820796264 | Low groups |
| TCGA-95-A4VN-01 | 1.515068493 | 0 | 1.625423154 | High groups |
| TCGA-86-7954-01 | 1.657534247 | 0 | 2.293164224 | High groups |
| TCGA-91-A4BC-01 | 0.120547945 | 0 | 2.062567236 | High groups |
| TCGA-MP-A4TJ-01 | 0.928767123 | 0 | 1.756718282 | High groups |
| TCGA-44-6146-01 | 1.728767123 | 1 | 2.529838685 | High groups |
| TCGA-62-A470-01 | 1.476712329 | 1 | 2.144292545 | High groups |
| TCGA-73-4677-01 | 0.104109589 | 1 | 0.725609374 | Low groups |
| TCGA-55-7911-01 | 1.410958904 | 1 | 1.262716152 | Low groups |
| TCGA-50-8460-01 | 2.271232877 | 0 | 1.013455135 | Low groups |
| TCGA-55-6981-01 | 3.778082192 | 0 | 2.306530855 | High groups |
| TCGA-44-8120-01 | 0.712328767 | 0 | 0.978632954 | Low groups |
| TCGA-55-8614-01 | 1.468493151 | 0 | 1.431145339 | Low groups |
| TCGA-64-1677-01 | 0.98630137 | 1 | 0.946225635 | Low groups |
| TCGA-97-8552-01 | 1.715068493 | 0 | 1.896813191 | High groups |
| TCGA-69-8253-01 | 1.167123288 | 0 | 0.799765243 | Low groups |
| TCGA-86-8668-01 | 1.15890411 | 0 | 2.169929969 | High groups |
| TCGA-86-8074-01 | 0.065753425 | 0 | 1.64240411 | High groups |
| TCGA-69-7765-01 | 0.452054795 | 0 | 1.828717702 | High groups |
| TCGA-55-8092-01 | 0.347945205 | 1 | 1.868213092 | High groups |
| TCGA-NJ-A4YQ-01 | 3.923287671 | 0 | 1.42390781 | Low groups |
| TCGA-55-6983-01 | 7.734246575 | 0 | 1.269248796 | Low groups |
| TCGA-62-8394-01 | 0.380821918 | 0 | 1.09572322 | Low groups |
| TCGA-44-2661-01 | 3.175342466 | 0 | 0.969613869 | Low groups |
| TCGA-44-7659-01 | 1.893150685 | 0 | 1.205222534 | Low groups |
| TCGA-55-6712-01 | 0.468493151 | 1 | 1.32393928 | Low groups |
| TCGA-05-4403-01 | 1.583561644 | 0 | 1.865191568 | High groups |
| TCGA-97-7553-01 | 5.123287671 | 0 | 1.660687288 | High groups |
| TCGA-78-8655-01 | 6.465753425 | 0 | 0.722147864 | Low groups |
| TCGA-55-7724-01 | 1.931506849 | 0 | 1.942162885 | High groups |
| TCGA-73-4659-01 | 0.095890411 | 1 | 1.405272525 | Low groups |
| TCGA-50-6673-01 | 0.060273973 | 0 | 1.200291216 | Low groups |
| TCGA-78-7542-01 | 0.879452055 | 0 | 1.432374221 | Low groups |
| TCGA-38-4625-01 | 8.145205479 | 0 | 0.764834094 | Low groups |
| TCGA-55-7570-01 | 2.257534247 | 0 | 2.148484801 | High groups |
| TCGA-69-7979-01 | 1.117808219 | 0 | 0.625871332 | Low groups |
| TCGA-55-A4DF-01 | 1.410958904 | 1 | 3.31283207 | High groups |
| TCGA-67-3773-01 | 1.169863014 | 0 | 0.853392496 | Low groups |
| TCGA-55-7573-01 | 1.334246575 | 0 | 2.157393744 | High groups |
| TCGA-50-5068-01 | 1.879452055 | 1 | 0.269212109 | Low groups |
| TCGA-49-AARN-01 | 3.109589041 | 0 | 1.490167295 | Low groups |
| TCGA-78-7150-01 | 0.380821918 | 1 | 1.56088226 | High groups |
| TCGA-MP-A4TA-01 | 1.98630137 | 1 | 1.321031431 | Low groups |
| TCGA-55-7907-01 | 0.805479452 | 1 | 0.752584366 | Low groups |
| TCGA-55-5899-01 | 2.547945205 | 0 | 1.69429329 | High groups |
| TCGA-55-7574-01 | 1.309589041 | 1 | 2.774862803 | High groups |
| TCGA-86-8073-01 | 2.02739726 | 0 | 2.354240689 | High groups |
| TCGA-L9-A7SV-01 | 1.547945205 | 0 | 2.391738851 | High groups |
| TCGA-93-A4JP-01 | 1.361643836 | 1 | 2.56497066 | High groups |
| TCGA-78-7163-01 | 19.85753425 | 0 | 1.065575209 | Low groups |
| TCGA-64-1678-01 | 3.257534247 | 0 | 0.430484279 | Low groups |
| TCGA-NJ-A55R-01 | 1.652054795 | 0 | 2.794423251 | High groups |
| TCGA-97-A4M7-01 | 1.723287671 | 0 | 2.591249888 | High groups |
| TCGA-38-A44F-01 | 0.364383562 | 0 | 2.068312109 | High groups |
| TCGA-62-8399-01 | 7.38630137 | 0 | 1.146391029 | Low groups |
| TCGA-49-4514-01 | 4.657534247 | 0 | 0.675086991 | Low groups |
| TCGA-44-6775-01 | 1.873972603 | 1 | 2.595031798 | High groups |
| TCGA-44-5643-01 | 2.775342466 | 0 | 1.15229138 | Low groups |
| TCGA-44-6147-01 | 2.315068493 | 0 | 1.87919484 | High groups |
| TCGA-J2-A4AE-01 | 2.956164384 | 0 | 1.866987466 | High groups |
| TCGA-44-2657-01 | 3.701369863 | 0 | 1.164506041 | Low groups |
| TCGA-78-7158-01 | 0.361643836 | 1 | 0.755215452 | Low groups |
| TCGA-93-7347-01 | 1.871232877 | 0 | 1.638595445 | High groups |
| TCGA-38-4630-01 | 1.435616438 | 1 | 1.224532964 | Low groups |
| TCGA-86-7953-01 | 2.731506849 | 0 | 1.632758712 | High groups |
| TCGA-50-5072-01 | 0.583561644 | 1 | 1.197191667 | Low groups |
| TCGA-62-A46S-01 | 1.443835616 | 1 | 2.876403082 | High groups |
| TCGA-53-A4EZ-01 | 2.934246575 | 0 | 1.315819799 | Low groups |
| TCGA-69-7973-01 | 0.630136986 | 0 | 1.221523013 | Low groups |
| TCGA-55-8204-01 | 1.410958904 | 0 | 1.225355734 | Low groups |
| TCGA-35-5375-01 | 0.723287671 | 0 | 0.778098029 | Low groups |
| TCGA-78-7539-01 | 1.884931507 | 1 | 1.161332175 | Low groups |
| TCGA-95-A4VK-01 | 1.35890411 | 1 | 1.723352813 | High groups |
| TCGA-50-5055-01 | 2.112328767 | 1 | 1.499847958 | Low groups |
| TCGA-44-7667-01 | 3.005479452 | 0 | 1.122830095 | Low groups |
| TCGA-MP-A4TI-01 | 0.230136986 | 1 | 2.043431399 | High groups |
| TCGA-55-6979-01 | 0.534246575 | 1 | 1.448200153 | Low groups |
| TCGA-55-7284-01 | 0.632876712 | 1 | 2.930691646 | High groups |
| TCGA-44-2656-01 | 1.556164384 | 1 | 1.182420943 | Low groups |
| TCGA-44-6778-01 | 5.106849315 | 0 | 2.189454818 | High groups |
| TCGA-35-4123-01 | 0.498630137 | 0 | 0.426047146 | Low groups |
| TCGA-44-6776-01 | 7.167123288 | 0 | 0.434638856 | Low groups |
| TCGA-73-4676-01 | 0.769863014 | 1 | 0.448429055 | Low groups |
| TCGA-86-8673-01 | 1.742465753 | 1 | 1.048649702 | Low groups |
| TCGA-62-8395-01 | 1.082191781 | 1 | 1.412084482 | Low groups |
| TCGA-44-A4SS-01 | 1.136986301 | 0 | 2.471032812 | High groups |
| TCGA-86-8358-01 | 1.789041096 | 0 | 1.82976978 | High groups |
| TCGA-55-6968-01 | 3.542465753 | 1 | 1.860981331 | High groups |
| TCGA-99-AA5R-01 | 1.802739726 | 0 | 2.040482509 | High groups |
| TCGA-55-8085-01 | 2.476712329 | 0 | 1.729744152 | High groups |
| TCGA-44-8117-01 | 1.054794521 | 0 | 1.318342205 | Low groups |
| TCGA-55-8506-01 | 0.030136986 | 0 | 0.673206927 | Low groups |
| TCGA-NJ-A7XG-01 | 1.690410959 | 0 | 0.733859785 | Low groups |
| TCGA-05-4384-01 | 0.501369863 | 1 | 2.418925878 | High groups |
| TCGA-64-1679-01 | 6.816438356 | 0 | 1.816561971 | High groups |
| TCGA-73-4666-01 | 2.191780822 | 0 | 1.282948814 | Low groups |
| TCGA-55-8616-01 | 0.131506849 | 0 | 0.775286429 | Low groups |
| TCGA-38-6178-01 | 1.22739726 | 0 | 1.54437078 | High groups |
| TCGA-93-7348-01 | 1.454794521 | 0 | 2.295128804 | High groups |
| TCGA-55-1594-01 | 3.22739726 | 0 | 1.941614874 | High groups |
| TCGA-73-4658-01 | 4.383561644 | 0 | 1.101429239 | Low groups |
| TCGA-55-8505-01 | 1.205479452 | 0 | 2.213854397 | High groups |
| TCGA-MP-A4T4-01 | 7.169863014 | 0 | 1.165356107 | Low groups |
| TCGA-49-4506-01 | 1.895890411 | 1 | 0.370256062 | Low groups |
| TCGA-97-8172-01 | 1.493150685 | 0 | 1.740193885 | High groups |
| TCGA-O1-A52J-01 | 2.460273973 | 1 | 2.477162374 | High groups |
| TCGA-78-7537-01 | 4.443835616 | 0 | 0.732337719 | Low groups |
| TCGA-73-4662-01 | 0.057534247 | 1 | 1.048833414 | Low groups |
| TCGA-62-8398-01 | 1.216438356 | 0 | 2.172892253 | High groups |
| TCGA-49-AAQV-01 | 1.394520548 | 1 | 2.037676719 | High groups |
| TCGA-55-8621-01 | 1.410958904 | 0 | 1.703114316 | High groups |
| TCGA-53-7626-01 | 2.369863014 | 1 | 1.253650945 | Low groups |
| TCGA-44-7669-01 | 1.068493151 | 1 | 2.213302592 | High groups |
| TCGA-93-8067-01 | 0.509589041 | 0 | 1.074159621 | Low groups |
| TCGA-97-A4M2-01 | 1.709589041 | 0 | 2.640924793 | High groups |
| TCGA-05-4425-01 | 1.832876712 | 0 | 1.569933489 | High groups |
| TCGA-69-7763-01 | 1.890410959 | 0 | 1.302978443 | Low groups |
| TCGA-86-8056-01 | 0.380821918 | 0 | 1.808431293 | High groups |
| TCGA-44-2662-01 | 0.671232877 | 1 | 2.124074853 | High groups |
| TCGA-50-5931-01 | 1.164383562 | 1 | 1.622245596 | High groups |
| TCGA-55-8512-01 | 1.663013699 | 1 | 2.353968262 | High groups |
| TCGA-75-7025-01 | 4.057534247 | 1 | 2.584775073 | High groups |
| TCGA-50-5930-01 | 0.484931507 | 1 | 1.589801974 | High groups |
| TCGA-75-6214-01 | 1.147945205 | 1 | 0.573696787 | Low groups |
| TCGA-L9-A743-01 | 1.819178082 | 0 | 1.513407901 | High groups |
| TCGA-50-5939-01 | 1.260273973 | 0 | 1.156857877 | Low groups |
| TCGA-78-7161-01 | 0.443835616 | 1 | 1.311564534 | Low groups |
| TCGA-44-5645-01 | 2.334246575 | 0 | 1.756659021 | High groups |
| TCGA-44-3398-01 | 3.18630137 | 0 | 1.397357158 | Low groups |
| TCGA-97-8547-01 | 1.8 | 0 | 2.578962369 | High groups |
| TCGA-91-6847-01 | 2.115068493 | 1 | 0.512139915 | Low groups |
| TCGA-55-8511-01 | 1.260273973 | 1 | 1.208382776 | Low groups |
| TCGA-50-5944-01 | 4.794520548 | 0 | 1.364610891 | Low groups |
| TCGA-55-1592-01 | 1.238356164 | 1 | 1.269324014 | Low groups |
| TCGA-50-8457-01 | 3.082191781 | 0 | 1.63334514 | High groups |
| TCGA-78-7159-01 | 5.408219178 | 0 | 1.012391299 | Low groups |
| TCGA-67-6215-01 | 0.476712329 | 0 | 1.143995991 | Low groups |
| TCGA-MP-A4T9-01 | 0.934246575 | 1 | 1.634591888 | High groups |
| TCGA-97-7938-01 | 0.049315068 | 0 | 1.702777524 | High groups |
| TCGA-05-5425-01 | 1.331506849 | 1 | 0.750153254 | Low groups |
| TCGA-55-7913-01 | 1.315068493 | 1 | 1.475590049 | Low groups |
| TCGA-05-4417-01 | 1.246575342 | 0 | 1.258524926 | Low groups |
| TCGA-05-4434-01 | 1.252054795 | 0 | 1.534524886 | High groups |
| TCGA-55-8096-01 | 1.550684932 | 1 | 2.30062399 | High groups |
| TCGA-49-6767-01 | 1.854794521 | 0 | 1.278359437 | Low groups |
| TCGA-44-2666-01 | 0.265753425 | 1 | 1.155376527 | Low groups |
| TCGA-MP-A4T6-01 | 4.904109589 | 0 | 2.439924332 | High groups |
| TCGA-MP-A4TE-01 | 0.619178082 | 1 | 0.744202051 | Low groups |
| TCGA-99-7458-01 | 2.046575342 | 0 | 1.53815874 | High groups |
| TCGA-91-6840-01 | 1.019178082 | 0 | 1.822631292 | High groups |
| TCGA-55-6986-01 | 8.934246575 | 0 | 0.972269982 | Low groups |
| TCGA-05-4395-01 | 0 | 0 | 0.336882772 | Low groups |
| TCGA-44-7672-01 | 1.969863014 | 0 | 0.735104137 | Low groups |
| TCGA-97-8175-01 | 0.805479452 | 1 | 1.823086684 | High groups |
| TCGA-55-8087-01 | 1.265753425 | 0 | 1.552032769 | High groups |
| TCGA-78-7160-01 | 1.909589041 | 0 | 1.954213747 | High groups |
| TCGA-L4-A4E5-01 | 1.583561644 | 0 | 0.866870925 | Low groups |
| TCGA-97-A4LX-01 | 1.682191781 | 0 | 2.445697736 | High groups |
| TCGA-86-8359-01 | 1.216438356 | 0 | 0.587629945 | Low groups |
| TCGA-44-6145-01 | 1.630136986 | 0 | 1.474631771 | Low groups |
| TCGA-78-7155-01 | 0.57260274 | 1 | 2.025676844 | High groups |
| TCGA-55-7816-01 | 1.282191781 | 1 | 2.578714221 | High groups |
| TCGA-99-8025-01 | 2.904109589 | 0 | 1.986273795 | High groups |
| TCGA-44-A47G-01 | 0.961643836 | 0 | 1.558178844 | High groups |
| TCGA-55-8615-01 | 0.61369863 | 1 | 2.258658547 | High groups |
| TCGA-73-7499-01 | 3.964383562 | 1 | 0.510670318 | Low groups |
| TCGA-86-7711-01 | 2.865753425 | 1 | 0.806321652 | Low groups |
| TCGA-86-8075-01 | 0.545205479 | 1 | 2.26145821 | High groups |
| TCGA-86-7955-01 | 2.246575342 | 1 | 1.129366688 | Low groups |
| TCGA-05-4415-01 | 0.164383562 | 1 | 0.929302931 | Low groups |
| TCGA-91-6830-01 | 0.049315068 | 1 | 2.052384725 | High groups |
| TCGA-49-6745-01 | 1.430136986 | 0 | 1.420333569 | Low groups |
| TCGA-86-A4P7-01 | 1.136986301 | 0 | 1.890559698 | High groups |
| TCGA-44-4112-01 | 1.695890411 | 1 | 1.482443577 | Low groups |
| TCGA-55-6975-01 | 0.323287671 | 1 | 1.620790624 | High groups |
| TCGA-95-7039-01 | 3.446575342 | 1 | 0.836965868 | Low groups |
| TCGA-05-4432-01 | 2.084931507 | 0 | 0.937130032 | Low groups |
| TCGA-49-4487-01 | 1.909589041 | 1 | 1.387418388 | Low groups |
| TCGA-55-7727-01 | 0.326027397 | 0 | 1.550825581 | High groups |
| TCGA-73-A9RS-01 | 0.293150685 | 1 | 0.669606889 | Low groups |
| TCGA-44-5644-01 | 2.364383562 | 0 | 2.438698505 | High groups |
| TCGA-55-7914-01 | 0.512328767 | 1 | 2.542205533 | High groups |
| TCGA-62-A46R-01 | 4.726027397 | 0 | 2.489280032 | High groups |
| TCGA-49-AARQ-01 | 18.44383562 | 0 | 2.772740602 | High groups |
| TCGA-53-7813-01 | 1.161643836 | 0 | 2.021770463 | High groups |
| TCGA-44-3918-01 | 1.4 | 1 | 1.709559003 | High groups |
| TCGA-64-1681-01 | 1.202739726 | 1 | 1.506293938 | High groups |
| TCGA-05-4410-01 | 0 | 0 | 0.897872987 | Low groups |
| TCGA-55-8620-01 | 1.02739726 | 1 | 2.086422835 | High groups |
| TCGA-64-5774-01 | 0.673972603 | 1 | 1.998088126 | High groups |
| TCGA-97-A4M1-01 | 1.646575342 | 0 | 2.169543856 | High groups |
| TCGA-83-5908-01 | 2.257534247 | 0 | 2.143170403 | High groups |
| TCGA-97-8179-01 | 1.191780822 | 0 | 2.268113393 | High groups |
| TCGA-64-1680-01 | 3.084931507 | 0 | 0.860072799 | Low groups |
| TCGA-73-4670-01 | 0.35890411 | 0 | 0.888149782 | Low groups |
| TCGA-44-3396-01 | 3.095890411 | 0 | 1.414849696 | Low groups |
| TCGA-80-5611-01 | 7.109589041 | 0 | 1.194297367 | Low groups |
| TCGA-53-7624-01 | 1.095890411 | 1 | 0.246901841 | Low groups |
| TCGA-91-6835-01 | 0.216438356 | 0 | 1.960101173 | High groups |
| TCGA-L9-A50W-01 | 1.065753425 | 1 | 0.722216073 | Low groups |
| TCGA-86-7713-01 | 3.169863014 | 0 | 1.518468273 | High groups |
| TCGA-50-5044-01 | 1.323287671 | 1 | 1.242399029 | Low groups |
| TCGA-50-6595-01 | 0.498630137 | 1 | 1.276226655 | Low groups |
| TCGA-55-7903-01 | 1.553424658 | 0 | 1.993740666 | High groups |
| TCGA-55-8508-01 | 1.690410959 | 0 | 1.698073387 | High groups |
| TCGA-67-3771-01 | 1.671232877 | 0 | 1.81222731 | High groups |
| TCGA-55-A4DG-01 | 1.665753425 | 0 | 2.324405301 | High groups |
| TCGA-91-7771-01 | 1.347945205 | 0 | 1.846435258 | High groups |
| TCGA-91-6849-01 | 0.095890411 | 0 | 1.185802974 | Low groups |
| TCGA-64-5781-01 | 0.263013699 | 1 | 0.451803755 | Low groups |
| TCGA-97-7552-01 | 2.18630137 | 1 | 2.388693104 | High groups |
| TCGA-80-5608-01 | 7.75890411 | 0 | 0.604956351 | Low groups |
| TCGA-91-6829-01 | 3.446575342 | 0 | 1.638158024 | High groups |
| TCGA-49-AARE-01 | 1.063013699 | 1 | 0.822084107 | Low groups |
| TCGA-97-7941-01 | 1.326027397 | 0 | 1.372878395 | Low groups |
| TCGA-86-7714-01 | 1.712328767 | 1 | 1.900490962 | High groups |
| TCGA-62-8402-01 | 2.115068493 | 1 | 1.258988971 | Low groups |
| TCGA-78-7162-01 | 6.076712329 | 1 | 1.094756193 | Low groups |
| TCGA-49-AAR0-01 | 13.05479452 | 0 | 1.717576081 | High groups |
| TCGA-35-4122-01 | 0.616438356 | 0 | 0.631902703 | Low groups |
| TCGA-55-7726-01 | 1.78630137 | 0 | 1.44023275 | Low groups |
| TCGA-62-A46Y-01 | 0.860273973 | 1 | 2.417858869 | High groups |
| TCGA-55-6980-01 | 5.778082192 | 0 | 2.23488962 | High groups |
| TCGA-05-4430-01 | 2.084931507 | 0 | 0.973854256 | Low groups |
| TCGA-67-3772-01 | 1.569863014 | 0 | 0.651855011 | Low groups |
| TCGA-MP-A4SY-01 | 1.339726027 | 1 | 1.39345343 | Low groups |
| TCGA-78-7148-01 | 0.498630137 | 1 | 1.114584323 | Low groups |
| TCGA-35-3615-01 | 0.038356164 | 0 | 0.839258567 | Low groups |
| TCGA-55-A493-01 | 0.076712329 | 0 | 2.06584961 | High groups |
| TCGA-49-6744-01 | 4.610958904 | 0 | 1.485479896 | Low groups |
| TCGA-49-4494-01 | 2.961643836 | 1 | 0.232344797 | Low groups |
| TCGA-78-7143-01 | 4.109589041 | 1 | 1.999092789 | High groups |
| TCGA-55-8513-01 | 0.868493151 | 1 | 1.902482162 | High groups |
| TCGA-MP-A4TC-01 | 0.202739726 | 0 | 0.990374466 | Low groups |
| TCGA-05-4420-01 | 2.498630137 | 0 | 0.659007514 | Low groups |
| TCGA-97-8174-01 | 0.449315068 | 0 | 2.353791923 | High groups |
| TCGA-44-6777-01 | 2.704109589 | 0 | 1.293603999 | Low groups |
| TCGA-62-A46P-01 | 0.731506849 | 1 | 1.163082776 | Low groups |
| TCGA-55-8089-01 | 1.923287671 | 0 | 1.071294791 | Low groups |
| TCGA-MN-A4N5-01 | 0.230136986 | 0 | 2.237569215 | High groups |
| TCGA-49-AAR9-01 | 0.712328767 | 1 | 0.78272076 | Low groups |
| TCGA-86-8671-01 | 2.298630137 | 0 | 2.419210299 | High groups |
| TCGA-49-4507-01 | 0.432876712 | 1 | 1.266146131 | Low groups |
| TCGA-62-A46V-01 | 6.024657534 | 0 | 1.509816105 | High groups |
| TCGA-05-4389-01 | 3.750684932 | 0 | 0.955698356 | Low groups |
| TCGA-50-6592-01 | 2.128767123 | 1 | 1.559464677 | High groups |
| TCGA-91-6831-01 | 0.849315068 | 0 | 1.396096113 | Low groups |
| TCGA-55-6987-01 | 5.854794521 | 0 | 1.551991252 | High groups |
| TCGA-49-4510-01 | 1.375342466 | 1 | 1.573053075 | High groups |
| TCGA-86-8672-01 | 0.052054795 | 0 | 1.521029811 | High groups |
| TCGA-44-2668-01 | 1.18630137 | 1 | 1.807011105 | High groups |
| TCGA-93-A4JQ-01 | 1.44109589 | 0 | 2.081697271 | High groups |
| TCGA-97-8177-01 | 1.367123288 | 0 | 1.29826126 | Low groups |
| TCGA-05-4396-01 | 0.830136986 | 0 | 2.000472008 | High groups |
| TCGA-55-6978-01 | 0.115068493 | 1 | 2.130007866 | High groups |
| TCGA-69-8453-01 | 0.942465753 | 1 | 1.942578834 | High groups |
| TCGA-50-7109-01 | 0.04109589 | 1 | 1.305695263 | Low groups |
| TCGA-49-4501-01 | 1.493150685 | 1 | 1.504523872 | High groups |
| TCGA-50-5049-01 | 4.295890411 | 1 | 0.9722944 | Low groups |
| TCGA-78-8660-01 | 0.663013699 | 1 | 1.078409043 | Low groups |
| TCGA-91-6828-01 | 0.884931507 | 0 | 1.656334084 | High groups |
| TCGA-50-6590-01 | 3.528767123 | 0 | 1.067856725 | Low groups |
| TCGA-97-8176-01 | 0.106849315 | 1 | 2.113407405 | High groups |
| TCGA-50-5935-01 | 1.789041096 | 0 | 0.870035492 | Low groups |
| TCGA-55-7281-01 | 0.928767123 | 1 | 0.738313106 | Low groups |
| TCGA-49-4488-01 | 1.736986301 | 1 | 0.699763504 | Low groups |
| TCGA-NJ-A55A-01 | 0.04109589 | 0 | 2.25533891 | High groups |
| TCGA-95-8494-01 | 0.230136986 | 0 | 0.48324712 | Low groups |
| TCGA-64-5775-01 | 0.169863014 | 1 | 0.88072446 | Low groups |
| TCGA-97-7547-01 | 2.123287671 | 1 | 1.391912853 | Low groups |
| TCGA-44-2655-01 | 2.764383562 | 1 | 1.549914523 | High groups |
| TCGA-75-6212-01 | 3.671232877 | 1 | 1.628002027 | High groups |
| TCGA-05-4390-01 | 1.082191781 | 1 | 0.71388067 | Low groups |
| TCGA-86-8674-01 | 0.915068493 | 1 | 1.088283171 | Low groups |
| TCGA-49-6742-01 | 0.58630137 | 1 | 1.232925732 | Low groups |
| TCGA-44-A47B-01 | 0.78630137 | 0 | 1.407456781 | Low groups |
| TCGA-MP-A4SW-01 | 4.871232877 | 0 | 1.630710583 | High groups |
| TCGA-64-5815-01 | 2.37260274 | 0 | 1.287550871 | Low groups |
| TCGA-MP-A4T8-01 | 0.44109589 | 0 | 1.345399873 | Low groups |
| TCGA-NJ-A4YI-01 | 0.010958904 | 0 | 0.906208721 | Low groups |
| TCGA-L4-A4E6-01 | 1.191780822 | 0 | 2.365263129 | High groups |
| TCGA-49-AAR4-01 | 2.408219178 | 1 | 1.670679997 | High groups |
| TCGA-97-A4M3-01 | 0.147945205 | 1 | 1.728856978 | High groups |
| TCGA-05-4397-01 | 2.002739726 | 0 | 0.402315699 | Low groups |
| TCGA-55-6984-01 | 1.983561644 | 1 | 2.359675546 | High groups |
| TCGA-05-4427-01 | 2.167123288 | 0 | 1.454172918 | Low groups |
| TCGA-86-8281-01 | 0 | 0 | 1.926177866 | High groups |
| TCGA-44-A4SU-01 | 0.712328767 | 1 | 1.745158546 | High groups |
| TCGA-75-5147-01 | 3.652054795 | 0 | 1.662213903 | High groups |
| TCGA-97-7546-01 | 3.438356164 | 1 | 1.52867228 | High groups |
| TCGA-86-8585-01 | 0.967123288 | 0 | 2.257050635 | High groups |
| TCGA-55-8301-01 | 0.652054795 | 1 | 1.378407677 | Low groups |
| TCGA-86-6562-01 | 0.750684932 | 1 | 1.325355505 | Low groups |
| TCGA-J2-A4AD-01 | 1.435616438 | 1 | 0.972290065 | Low groups |
| TCGA-50-5946-01 | 0.605479452 | 1 | 2.014241763 | High groups |
| TCGA-55-8510-01 | 1.476712329 | 0 | 1.27159533 | Low groups |
| TCGA-99-8033-01 | 1.797260274 | 1 | 2.585650595 | High groups |
| TCGA-69-7978-01 | 0.367123288 | 0 | 1.973006148 | High groups |
| TCGA-86-8054-01 | 3.145205479 | 0 | 2.290841503 | High groups |
| TCGA-55-8302-01 | 1.309589041 | 0 | 0.646402985 | Low groups |
| TCGA-05-4405-01 | 1.671232877 | 0 | 1.397505162 | Low groups |
| TCGA-78-7147-01 | 1.583561644 | 1 | 1.652494964 | High groups |
| TCGA-MP-A4TD-01 | 0.619178082 | 1 | 2.647890174 | High groups |
| TCGA-55-6971-01 | 3.835616438 | 0 | 1.690095651 | High groups |
| TCGA-50-6591-01 | 0.326027397 | 1 | 0.947867452 | Low groups |
| TCGA-95-7944-01 | 1.032876712 | 0 | 1.507102472 | High groups |
| TCGA-NJ-A4YF-01 | 5.920547945 | 0 | 1.125428253 | Low groups |
| TCGA-55-A48Z-01 | 1.468493151 | 1 | 1.622425158 | High groups |
| TCGA-05-5428-01 | 1.835616438 | 0 | 0.460826876 | Low groups |
| TCGA-69-8255-01 | 0.353424658 | 0 | 1.370725593 | Low groups |
| TCGA-75-5125-01 | 4.8 | 1 | 1.608076272 | High groups |
| TCGA-55-8097-01 | 1.304109589 | 0 | 1.796336555 | High groups |
| TCGA-86-A4D0-01 | 0.317808219 | 0 | 1.844058155 | High groups |
| TCGA-L9-A444-01 | 0.84109589 | 0 | 1.312063257 | Low groups |
| TCGA-MP-A4T7-01 | 0.457534247 | 0 | 1.92267544 | High groups |
| TCGA-91-A4BD-01 | 1.652054795 | 0 | 0.536839776 | Low groups |
| TCGA-05-4250-01 | 0.331506849 | 0 | 1.068628247 | Low groups |
| TCGA-55-7994-01 | 1.652054795 | 0 | 1.459755154 | Low groups |
| TCGA-99-8028-01 | 3.063013699 | 0 | 0.929068181 | Low groups |
| TCGA-97-8171-01 | 1.208219178 | 1 | 1.795405219 | High groups |
| TCGA-67-6216-01 | 0.38630137 | 0 | 1.524344439 | High groups |
| TCGA-91-6848-01 | 0.61369863 | 0 | 1.611385543 | High groups |
| TCGA-44-2665-01 | 3.564383562 | 0 | 2.576507437 | High groups |
| TCGA-86-7701-01 | 1.161643836 | 1 | 2.02304394 | High groups |
| TCGA-78-7156-01 | 2.673972603 | 0 | 2.582318207 | High groups |
| TCGA-05-5420-01 | 0.671232877 | 1 | 0.409901148 | Low groups |
| TCGA-55-6642-01 | 6.709589041 | 0 | 1.468777404 | Low groups |
| TCGA-78-7149-01 | 10.79452055 | 0 | 1.191836877 | Low groups |
| TCGA-05-4418-01 | 0.750684932 | 0 | 0.995973788 | Low groups |
| TCGA-05-4422-01 | 1 | 0 | 1.92812918 | High groups |
| TCGA-78-7146-01 | 0.473972603 | 0 | 0.808867754 | Low groups |
| TCGA-86-A4P8-01 | 2.205479452 | 0 | 2.738010288 | High groups |
| TCGA-67-6217-01 | 0.810958904 | 1 | 2.076623768 | High groups |
| TCGA-78-8640-01 | 19.34794521 | 0 | 0.694320519 | Low groups |
| TCGA-44-7670-01 | 2.416438356 | 0 | 0.537519284 | Low groups |
| TCGA-49-AARO-01 | 3.134246575 | 1 | 2.280868546 | High groups |
| TCGA-50-5941-01 | 4.038356164 | 0 | 0.856136519 | Low groups |
| TCGA-49-AARR-01 | 3.010958904 | 1 | 2.271445118 | High groups |
| TCGA-J2-8192-01 | 1.320547945 | 1 | 2.164529512 | High groups |
| TCGA-64-5779-01 | 2.178082192 | 1 | 2.097812382 | High groups |
| TCGA-62-A46O-01 | 2.583561644 | 1 | 2.813656841 | High groups |
| TCGA-50-8459-01 | 1.183561644 | 1 | 2.115885909 | High groups |
| TCGA-86-A4JF-01 | 1.194520548 | 1 | 1.182829416 | Low groups |
| TCGA-05-4249-01 | 4.17260274 | 0 | 0.990641371 | Low groups |
| TCGA-J2-A4AG-01 | 2.706849315 | 0 | 2.029185888 | High groups |
| TCGA-86-8280-01 | 1.920547945 | 0 | 1.780197166 | High groups |
| TCGA-78-7166-01 | 0.706849315 | 0 | 1.452967181 | Low groups |
| TCGA-91-8499-01 | 0.098630137 | 0 | 1.18701741 | Low groups |
| TCGA-71-6725-01 | 0.449315068 | 1 | 0.434356183 | Low groups |
| TCGA-69-7980-01 | 1.126027397 | 0 | 1.980510348 | High groups |
| TCGA-L9-A8F4-01 | 1.304109589 | 0 | 1.970696761 | High groups |
| TCGA-05-4433-01 | 2 | 0 | 2.411689611 | High groups |
| TCGA-55-A492-01 | 1.632876712 | 0 | 1.500747171 | Low groups |
| TCGA-73-4675-01 | 0.989041096 | 1 | 2.091852265 | High groups |
| TCGA-50-5936-01 | 0.410958904 | 1 | 1.889378999 | High groups |
| TCGA-44-7662-01 | 0.597260274 | 0 | 1.623976087 | High groups |
| TCGA-78-7154-01 | 1.624657534 | 0 | 1.461566351 | Low groups |
| TCGA-05-4244-01 | 0 | 0 | 2.398140968 | High groups |
| TCGA-73-7498-01 | 3.257534247 | 0 | 1.324310938 | Low groups |
| TCGA-50-5933-01 | 6.556164384 | 0 | 1.876721566 | High groups |
| TCGA-55-8507-01 | 1.145205479 | 0 | 1.662408544 | High groups |
| TCGA-S2-AA1A-01 | 1.405479452 | 0 | 1.814785969 | High groups |
| TCGA-95-7948-01 | 1.304109589 | 0 | 0.403735467 | Low groups |
| TCGA-67-3774-01 | 1.054794521 | 0 | 1.693652298 | High groups |
| TCGA-38-4628-01 | 2.967123288 | 1 | 0.576597839 | Low groups |
| TCGA-50-5051-01 | 0.504109589 | 1 | 1.784762982 | High groups |
| TCGA-86-6851-01 | 0.490410959 | 0 | 1.76706415 | High groups |
| TCGA-44-6148-01 | 1.928767123 | 0 | 2.578802785 | High groups |
| TCGA-MN-A4N1-01 | 2.265753425 | 0 | 1.421595027 | Low groups |
| TCGA-05-5429-01 | 0.753424658 | 0 | 1.933222274 | High groups |
| TCGA-55-8091-01 | 1.643835616 | 0 | 2.271822059 | High groups |
| TCGA-44-3917-01 | 3.24109589 | 0 | 1.763933595 | High groups |
| TCGA-49-4505-01 | 1.142465753 | 1 | 0.626183195 | Low groups |
| TCGA-62-8397-01 | 3.531506849 | 0 | 1.49909474 | Low groups |
| TCGA-50-5942-01 | 3.775342466 | 1 | 1.376977775 | Low groups |
| TCGA-55-8206-01 | 2.432876712 | 0 | 2.121308148 | High groups |
| TCGA-55-8094-01 | 1.482191781 | 0 | 1.549311941 | High groups |
| TCGA-05-4382-01 | 0.915068493 | 1 | 1.654323068 | High groups |
| TCGA-05-5423-01 | 0.41369863 | 0 | 1.266068231 | Low groups |
| TCGA-75-5146-01 | 4.857534247 | 1 | 0.834929536 | Low groups |
| TCGA-78-7167-01 | 1.989041096 | 1 | 2.008943615 | High groups |
| TCGA-J2-8194-01 | 1.287671233 | 1 | 1.702821995 | High groups |
| TCGA-97-A4M0-01 | 1.78630137 | 0 | 1.566901688 | High groups |
| TCGA-38-4632-01 | 1.863013699 | 1 | 0.284441865 | Low groups |
| TCGA-44-7661-01 | 0.917808219 | 1 | 2.268734693 | High groups |
| TCGA-55-8205-01 | 1.356164384 | 1 | 1.646875818 | High groups |
| TCGA-05-4424-01 | 0.419178082 | 1 | 1.243442079 | Low groups |
| TCGA-38-4631-01 | 0.969863014 | 1 | 0.344775545 | Low groups |
| TCGA-55-7283-01 | 1.668493151 | 0 | 0.630390203 | Low groups |
| TCGA-95-7567-01 | 1.556164384 | 0 | 1.017556061 | Low groups |
| TCGA-38-4629-01 | 1.038356164 | 1 | 0.865723462 | Low groups |
| TCGA-91-8497-01 | 1.189041096 | 0 | 1.701609551 | High groups |
| TCGA-78-7540-01 | 3.279452055 | 0 | 3.171134919 | High groups |
| TCGA-55-A48Y-01 | 1.726027397 | 0 | 1.250441581 | Low groups |
| TCGA-55-7995-01 | 1.282191781 | 1 | 2.469955165 | High groups |
| TCGA-44-3919-01 | 2.523287671 | 1 | 1.396260182 | Low groups |
| TCGA-69-7764-01 | 1.134246575 | 0 | 0.700801498 | Low groups |
| TCGA-95-7947-01 | 1.306849315 | 0 | 1.89124791 | High groups |
| TCGA-MP-A4TH-01 | 2.030136986 | 0 | 2.182868017 | High groups |
| TCGA-55-7725-01 | 1.210958904 | 0 | 1.616933298 | High groups |
| TCGA-L9-A5IP-01 | 0.134246575 | 1 | 1.657466771 | High groups |
| TCGA-97-7554-01 | 2.123287671 | 0 | 1.170484239 | Low groups |
| TCGA-55-8619-01 | 1.139726027 | 0 | 1.985131907 | High groups |
| TCGA-55-7227-01 | 0.698630137 | 1 | 1.883857101 | High groups |
| TCGA-67-3770-01 | 1.671232877 | 0 | 0.884390661 | Low groups |
| TCGA-78-7145-01 | 1.156164384 | 1 | 0.652235311 | Low groups |
| TCGA-49-AAR3-01 | 5.18630137 | 1 | 1.880317626 | High groups |
| TCGA-44-A479-01 | 1.197260274 | 1 | 1.894910388 | High groups |
| TCGA-4B-A93V-01 | 0.687671233 | 1 | 2.222569786 | High groups |
| TCGA-78-7633-01 | 3.97260274 | 1 | 1.838523466 | High groups |
| TCGA-NJ-A4YP-01 | 0.136986301 | 0 | 1.659690364 | High groups |
| TCGA-38-4626-01 | 6.898630137 | 1 | 1.739555593 | High groups |
| TCGA-78-7535-01 | 2.216438356 | 1 | 0.917067347 | Low groups |
| TCGA-55-6970-01 | 1.252054795 | 1 | 0.936654135 | Low groups |
| TCGA-55-6543-01 | 1.191780822 | 0 | 0.607195605 | Low groups |
| TCGA-05-4402-01 | 0.668493151 | 0 | 1.252639865 | Low groups |
| TCGA-55-1596-01 | 5.657534247 | 0 | 1.066282555 | Low groups |
| TCGA-49-4490-01 | 1.054794521 | 1 | 1.353459864 | Low groups |
| TCGA-62-A471-01 | 3.41369863 | 0 | 1.512071412 | High groups |
| TCGA-86-A456-01 | 2.454794521 | 0 | 1.269687671 | Low groups |
| TCGA-49-4512-01 | 2.479452055 | 1 | 2.708399349 | High groups |
| TCGA-55-A48X-01 | 1.684931507 | 1 | 1.563241765 | High groups |
| TCGA-86-8278-01 | 0.079452055 | 1 | 1.222917224 | Low groups |
| TCGA-55-6982-01 | 0.501369863 | 1 | 0.826958123 | Low groups |
| TCGA-50-5045-01 | 3.926027397 | 1 | 0.7381062 | Low groups |
| TCGA-05-4398-01 | 3.920547945 | 0 | 1.509270822 | High groups |
| TCGA-78-7152-01 | 3.293150685 | 1 | 1.09696795 | Low groups |
| TCGA-MP-A4TF-01 | 0.534246575 | 1 | 0.906040485 | Low groups |
| TCGA-67-4679-01 | 1.22739726 | 0 | 0.972851537 | Low groups |
| TCGA-91-6836-01 | 1.142465753 | 0 | 1.47486212 | Low groups |
| TCGA-78-8648-01 | 1.97260274 | 1 | 1.579124773 | High groups |
| TCGA-44-A47A-01 | 1.087671233 | 1 | 1.052819501 | Low groups |
| TCGA-55-A57B-01 | 1.495890411 | 0 | 2.483005496 | High groups |
| TCGA-55-8207-01 | 2.676712329 | 0 | 1.400678824 | Low groups |
| TCGA-55-7576-01 | 1.835616438 | 0 | 0.868343288 | Low groups |
| TCGA-NJ-A55O-01 | 0.035616438 | 0 | 1.751522426 | High groups |
| TCGA-55-A494-01 | 1.317808219 | 0 | 2.028049384 | High groups |
| TCGA-95-7043-01 | 1.378082192 | 1 | 0.935078112 | Low groups |
| TCGA-L9-A443-01 | 0.528767123 | 0 | 1.255509532 | Low groups |
| TCGA-69-7974-01 | 0.504109589 | 0 | 1.121130255 | Low groups |
| TCGA-NJ-A4YG-01 | 6.194520548 | 0 | 1.114432092 | Low groups |
| TCGA-69-7760-01 | 0.553424658 | 0 | 1.86343794 | High groups |
| TCGA-49-4486-01 | 5.602739726 | 1 | 0.79714197 | Low groups |
| TCGA-93-A4JN-01 | 1.967123288 | 0 | 1.95845006 | High groups |
| TCGA-44-7671-01 | 2.419178082 | 1 | 1.535930471 | High groups |
| TCGA-44-6774-01 | 1.802739726 | 0 | 2.092925249 | High groups |
| TCGA-50-5066-01 | 1.306849315 | 1 | 0.415160557 | Low groups |
| TCGA-50-6594-01 | 0.780821918 | 1 | 0.506971839 | Low groups |
| TCGA-44-8119-01 | 0.780821918 | 0 | 1.847138783 | High groups |
| TCGA-49-AAR2-01 | 6.093150685 | 0 | 1.758207247 | High groups |
| TCGA-44-6779-01 | 0.635616438 | 1 | 0.577237411 | Low groups |
| TCGA-86-8669-01 | 2.326027397 | 1 | 0.544853832 | Low groups |
| TCGA-05-5715-01 | 0.169863014 | 0 | 0.526048685 | Low groups |
| TCGA-69-A59K-01 | 1.619178082 | 0 | 1.062836525 | Low groups |
| TCGA-55-A491-01 | 1.715068493 | 0 | 1.526425796 | High groups |
| TCGA-71-8520-01 | 0.490410959 | 1 | 2.631029242 | High groups |
| TCGA-91-8496-01 | 1.383561644 | 0 | 0.416948584 | Low groups |
| TCGA-69-8254-01 | 1.120547945 | 0 | 0.93305767 | Low groups |
| TCGA-95-A4VP-01 | 0.591780822 | 1 | 1.974752007 | High groups |
| TCGA-95-8039-01 | 0.624657534 | 1 | 2.217145053 | High groups |
| TCGA-86-8076-01 | 2.720547945 | 0 | 1.277445121 | Low groups |
| TCGA-44-2659-01 | 3.139726027 | 1 | 1.271168461 | Low groups |
| TCGA-55-8203-01 | 1.498630137 | 0 | 0.947877872 | Low groups |
| TCGA-MP-A5C7-01 | 6.15890411 | 0 | 1.195596494 | Low groups |
| TCGA-78-7220-01 | 1.454794521 | 1 | 1.86413697 | High groups |
| TCGA-78-7153-01 | 9.95890411 | 0 | 0.776239674 | Low groups |
| TCGA-55-7910-01 | 2.789041096 | 1 | 1.936044482 | High groups |
| TCGA-64-5778-01 | 2.635616438 | 1 | 0.895467976 | Low groups |
| TCGA-55-7815-01 | 1.276712329 | 1 | 1.793937202 | High groups |
| TCGA-55-6985-01 | 3.378082192 | 0 | 1.396744203 | Low groups |
| TCGA-69-7761-01 | 0.509589041 | 0 | 2.55841662 | High groups |
| TCGA-MN-A4N4-01 | 3.219178082 | 0 | 1.822540317 | High groups |
| TCGA-55-6972-01 | 4.471232877 | 0 | 2.517390952 | High groups |

**Table S10.** Raw counts of RNA-sequencing data of hsa-let-7d-3p in LUSC from the TCGA.

| sampleID | time | Staus | RS | Label |
| --- | --- | --- | --- | --- |
| TCGA-18-3406-01 | 0.978082192 | 1 | 6.767459407 | Low groups |
| TCGA-18-3407-01 | 0.37260274 | 0 | 8.698087075 | High groups |
| TCGA-18-3408-01 | 4.912328767 | 1 | 8.559145399 | Low groups |
| TCGA-18-3410-01 | 0.4 | 0 | 8.288052585 | Low groups |
| TCGA-18-3411-01 | 9.797260274 | 0 | 7.314160944 | Low groups |
| TCGA-18-3412-01 | 0.747945205 | 1 | 8.682264276 | High groups |
| TCGA-18-3414-01 | 1.961643836 | 0 | 8.387017043 | Low groups |
| TCGA-18-3415-01 | 7.679452055 | 0 | 8.222639252 | Low groups |
| TCGA-18-3416-01 | 2.575342466 | 1 | 9.697402425 | High groups |
| TCGA-18-3417-01 | 3.005479452 | 0 | 7.610381898 | Low groups |
| TCGA-18-3419-01 | 7.701369863 | 0 | 8.626248675 | High groups |
| TCGA-18-3421-01 | 7.246575342 | 0 | 7.699370896 | Low groups |
| TCGA-18-4086-01 | 0.232876712 | 0 | 8.749507653 | High groups |
| TCGA-18-4721-01 | 12.86027397 | 0 | 8.73699012 | High groups |
| TCGA-18-5592-01 | 4.161643836 | 0 | 10.39684728 | High groups |
| TCGA-18-5595-01 | 2.265753425 | 0 | 10.13847622 | High groups |
| TCGA-21-1072-01 | 8.263013699 | 0 | 9.656648419 | High groups |
| TCGA-21-1075-01 | 5.846575342 | 0 | 9.407191059 | High groups |
| TCGA-21-1076-01 | 4.315068493 | 1 | 9.251217754 | High groups |
| TCGA-21-1077-01 | 1.764383562 | 1 | 7.84869016 | Low groups |
| TCGA-21-1078-01 | 0.704109589 | 1 | 8.762064162 | High groups |
| TCGA-21-1079-01 | 0.868493151 | 1 | 8.396419659 | Low groups |
| TCGA-21-1080-01 | 10.20273973 | 0 | 9.616091336 | High groups |
| TCGA-21-1083-01 | 3.602739726 | 0 | 8.198386743 | Low groups |
| TCGA-21-5782-01 | 2.635616438 | 0 | 9.001703516 | High groups |
| TCGA-21-5783-01 | 7.342465753 | 0 | 9.012683028 | High groups |
| TCGA-21-5784-01 | 3.473972603 | 0 | 8.049320917 | Low groups |
| TCGA-21-5786-01 | 2.238356164 | 1 | 8.228772043 | Low groups |
| TCGA-21-5787-01 | 0.282191781 | 1 | 9.551621082 | High groups |
| TCGA-21-A5DI-01 | 2.682191781 | 0 | 8.357911182 | Low groups |
| TCGA-22-0940-01 | 1.832876712 | 1 | 8.683220693 | High groups |
| TCGA-22-0944-01 | 0.610958904 | 0 | 8.676370981 | High groups |
| TCGA-22-1011-01 | 0.145205479 | 0 | 8.065506504 | Low groups |
| TCGA-22-1012-01 | 1.175342466 | 0 | 7.912059338 | Low groups |
| TCGA-22-4591-01 | 1.21369863 | 1 | 9.150848532 | High groups |
| TCGA-22-4593-01 | 2.923287671 | 0 | 8.615224375 | High groups |
| TCGA-22-4594-01 | 4.02739726 | 0 | 7.316941739 | Low groups |
| TCGA-22-4595-01 | 0.684931507 | 1 | 8.927535913 | High groups |
| TCGA-22-4596-01 | 0.046575342 | 0 | 8.159358332 | Low groups |
| TCGA-22-4601-01 | 1.438356164 | 1 | 10.51929588 | High groups |
| TCGA-22-4604-01 | 0.838356164 | 1 | 8.677547917 | High groups |
| TCGA-22-4605-01 | 2.668493151 | 0 | 8.898679165 | High groups |
| TCGA-22-4607-01 | 1.608219178 | 0 | 8.138548689 | Low groups |
| TCGA-22-4609-01 | 0.797260274 | 0 | 9.141827939 | High groups |
| TCGA-22-4613-01 | 0.980821918 | 0 | 9.860510178 | High groups |
| TCGA-22-5471-01 | 0.62739726 | 1 | 8.886003241 | High groups |
| TCGA-22-5472-01 | 2.043835616 | 1 | 9.539892053 | High groups |
| TCGA-22-5473-01 | 4.534246575 | 1 | 9.334135223 | High groups |
| TCGA-22-5474-01 | 1.219178082 | 0 | 8.753519466 | High groups |
| TCGA-22-5477-01 | 1.4 | 1 | 10.29020228 | High groups |
| TCGA-22-5478-01 | 0.065753425 | 0 | 8.510944914 | Low groups |
| TCGA-22-5479-01 | 7.191780822 | 0 | 8.65375045 | High groups |
| TCGA-22-5480-01 | 4.873972603 | 1 | 9.536268919 | High groups |
| TCGA-22-5481-01 | 0.947945205 | 1 | 10.23579933 | High groups |
| TCGA-22-5482-01 | 0.978082192 | 0 | 9.824281613 | High groups |
| TCGA-22-5483-01 | 1.556164384 | 1 | 8.2123636 | Low groups |
| TCGA-22-5485-01 | 2.301369863 | 1 | 10.60254661 | High groups |
| TCGA-22-5489-01 | 1.17260274 | 1 | 8.836796765 | High groups |
| TCGA-22-5491-01 | 4.693150685 | 0 | 9.02600658 | High groups |
| TCGA-22-5492-01 | 1.38630137 | 0 | 10.18261347 | High groups |
| TCGA-22-A5C4-01 | 1.838356164 | 0 | 9.031705889 | High groups |
| TCGA-33-4532-01 | 10.75068493 | 0 | 9.111334529 | High groups |
| TCGA-33-4533-01 | 11.14520548 | 0 | 8.612219231 | High groups |
| TCGA-33-4538-01 | 8.161643836 | 0 | 9.750750693 | High groups |
| TCGA-33-4547-01 | 6.62739726 | 0 | 8.27469338 | Low groups |
| TCGA-33-4566-01 | 14.48493151 | 0 | 9.88827384 | High groups |
| TCGA-33-4582-01 | 8.008219178 | 1 | 8.140198016 | Low groups |
| TCGA-33-4583-01 | 12.60547945 | 0 | 9.3412188 | High groups |
| TCGA-33-4586-01 | 0.463013699 | 1 | 8.382269523 | Low groups |
| TCGA-33-4587-01 | 4.536986301 | 1 | 8.073081777 | Low groups |
| TCGA-33-4589-01 | 0.128767123 | 0 | 8.09750219 | Low groups |
| TCGA-33-6737-01 | 1.646575342 | 1 | 9.131619578 | High groups |
| TCGA-33-6738-01 | 5.279452055 | 0 | 9.340334827 | High groups |
| TCGA-33-A4WN-01 | 0.391780822 | 0 | 8.029222339 | Low groups |
| TCGA-33-A5GW-01 | 0.024657534 | 0 | 8.880270342 | High groups |
| TCGA-33-AAS8-01 | 3.052054795 | 0 | 9.219602474 | High groups |
| TCGA-33-AASB-01 | 0.082191781 | 1 | 9.313866873 | High groups |
| TCGA-33-AASD-01 | 5.238356164 | 1 | 8.59143322 | High groups |
| TCGA-33-AASI-01 | 3.682191781 | 1 | 7.896780856 | Low groups |
| TCGA-33-AASJ-01 | 3.109589041 | 1 | 8.707990259 | High groups |
| TCGA-33-AASL-01 | 0.408219178 | 1 | 8.09931025 | Low groups |
| TCGA-34-2596-01 | 0.219178082 | 0 | 8.510220212 | Low groups |
| TCGA-34-2600-01 | 5.134246575 | 0 | 9.711422116 | High groups |
| TCGA-34-2604-01 | 2.624657534 | 0 | 8.679493647 | High groups |
| TCGA-34-2605-01 | 1.079452055 | 1 | 7.86597101 | Low groups |
| TCGA-34-2608-01 | 2.739726027 | 0 | 8.096533746 | Low groups |
| TCGA-34-2609-01 | 1.060273973 | 1 | 8.486655091 | Low groups |
| TCGA-34-5231-01 | 5.435616438 | 0 | 8.465321514 | Low groups |
| TCGA-34-5232-01 | 6.769863014 | 0 | 9.071274103 | High groups |
| TCGA-34-5234-01 | 6.221917808 | 0 | 6.596965397 | Low groups |
| TCGA-34-5236-01 | 0.309589041 | 1 | 8.289369534 | Low groups |
| TCGA-34-5239-01 | 4.191780822 | 1 | 8.167130582 | Low groups |
| TCGA-34-5240-01 | 4.221917808 | 0 | 8.180735816 | Low groups |
| TCGA-34-5241-01 | 1.410958904 | 0 | 7.908500346 | Low groups |
| TCGA-34-5927-01 | 0.671232877 | 1 | 8.43928985 | Low groups |
| TCGA-34-5928-01 | 3.276712329 | 0 | 9.082545861 | High groups |
| TCGA-34-5929-01 | 0.41369863 | 0 | 8.939113657 | High groups |
| TCGA-34-7107-01 | 0.093150685 | 0 | 10.06243436 | High groups |
| TCGA-34-8454-01 | 3.232876712 | 0 | 10.15497047 | High groups |
| TCGA-34-8455-01 | 0.336986301 | 1 | 8.921432106 | High groups |
| TCGA-34-8456-01 | 2.202739726 | 0 | 9.198046606 | High groups |
| TCGA-34-A5IX-01 | 2.824657534 | 0 | 8.560959 | Low groups |
| TCGA-37-3783-01 | 0.334246575 | 0 | 10.39136959 | High groups |
| TCGA-37-3789-01 | 0.035616438 | 0 | 7.686885801 | Low groups |
| TCGA-37-3792-01 | 0.032876712 | 0 | 7.615106384 | Low groups |
| TCGA-37-4130-01 | 0.676712329 | 0 | 6.788181018 | Low groups |
| TCGA-37-4133-01 | 0.652054795 | 0 | 9.329810014 | High groups |
| TCGA-37-4135-01 | 0.567123288 | 0 | 8.63529742 | High groups |
| TCGA-37-4141-01 | 0.032876712 | 0 | 8.486843064 | Low groups |
| TCGA-37-5819-01 | 0.282191781 | 0 | 8.392562059 | Low groups |
| TCGA-37-A5EL-01 | 2.591780822 | 1 | 8.881587784 | High groups |
| TCGA-37-A5EM-01 | 2.375342466 | 0 | 8.547353041 | Low groups |
| TCGA-37-A5EN-01 | 1.808219178 | 0 | 8.500861553 | Low groups |
| TCGA-39-5011-01 | 1.547945205 | 1 | 8.297320288 | Low groups |
| TCGA-39-5019-01 | 1.035616438 | 1 | 7.018539819 | Low groups |
| TCGA-39-5021-01 | 5.136986301 | 1 | 8.76251871 | High groups |
| TCGA-39-5022-01 | 3.967123288 | 1 | 8.147216273 | Low groups |
| TCGA-39-5024-01 | 6.876712329 | 0 | 8.444133229 | Low groups |
| TCGA-39-5027-01 | 8.44109589 | 1 | 8.296084434 | Low groups |
| TCGA-39-5028-01 | 0.142465753 | 0 | 7.979400957 | Low groups |
| TCGA-39-5029-01 | 1.345205479 | 1 | 8.134022206 | Low groups |
| TCGA-39-5030-01 | 0.161643836 | 0 | 7.769964028 | Low groups |
| TCGA-39-5031-01 | 5.043835616 | 0 | 8.882752562 | High groups |
| TCGA-39-5034-01 | 0.964383562 | 1 | 8.131398105 | Low groups |
| TCGA-39-5035-01 | 5.698630137 | 0 | 7.41476446 | Low groups |
| TCGA-39-5036-01 | 5.931506849 | 0 | 8.884446574 | High groups |
| TCGA-39-5037-01 | 4.630136986 | 0 | 8.158877906 | Low groups |
| TCGA-39-5039-01 | 1.490410959 | 0 | 7.850203218 | Low groups |
| TCGA-39-5040-01 | 1.210958904 | 1 | 10.03045127 | High groups |
| TCGA-43-2576-01 | 3.350684932 | 0 | 7.340189442 | Low groups |
| TCGA-43-2578-01 | 1.873972603 | 0 | 9.175770959 | High groups |
| TCGA-43-2581-01 | 3.221917808 | 0 | 8.382159696 | Low groups |
| TCGA-43-3394-01 | 1.512328767 | 1 | 8.901092853 | High groups |
| TCGA-43-3920-01 | 2.75890411 | 0 | 6.740894312 | Low groups |
| TCGA-43-5668-01 | 1.287671233 | 1 | 9.958974443 | High groups |
| TCGA-43-5670-01 | 2.326027397 | 0 | 9.368554041 | High groups |
| TCGA-43-6143-01 | 1.915068493 | 0 | 8.468246796 | Low groups |
| TCGA-43-6647-01 | 2.073972603 | 0 | 8.585581149 | Low groups |
| TCGA-43-6770-01 | 1.789041096 | 0 | 9.145571643 | High groups |
| TCGA-43-6771-01 | 0.421917808 | 1 | 8.830837397 | High groups |
| TCGA-43-6773-01 | 0.317808219 | 0 | 9.103610149 | High groups |
| TCGA-43-7656-01 | 1.632876712 | 0 | 8.580688956 | Low groups |
| TCGA-43-7657-01 | 0.646575342 | 0 | 9.090114273 | High groups |
| TCGA-43-7658-01 | 6.315068493 | 1 | 8.388280521 | Low groups |
| TCGA-43-8115-01 | 1.115068493 | 0 | 7.788355012 | Low groups |
| TCGA-43-8116-01 | 0.980821918 | 0 | 7.985318374 | Low groups |
| TCGA-43-8118-01 | 0.243835616 | 0 | 9.040821297 | High groups |
| TCGA-43-A474-01 | 0.967123288 | 0 | 8.486450842 | Low groups |
| TCGA-43-A475-01 | 0.810958904 | 0 | 8.319347151 | Low groups |
| TCGA-43-A56U-01 | 1.183561644 | 0 | 7.971911078 | Low groups |
| TCGA-43-A56V-01 | 0.97260274 | 1 | 7.976573918 | Low groups |
| TCGA-46-3765-01 | 1.109589041 | 0 | 8.416459051 | Low groups |
| TCGA-46-3766-01 | 1.01369863 | 0 | 7.235015204 | Low groups |
| TCGA-46-3767-01 | 1.084931507 | 0 | 7.179953564 | Low groups |
| TCGA-46-3768-01 | 0.819178082 | 0 | 9.562776943 | High groups |
| TCGA-46-3769-01 | 0.369863014 | 0 | 7.615020285 | Low groups |
| TCGA-46-6025-01 | 0.887671233 | 0 | 9.251572354 | High groups |
| TCGA-46-6026-01 | 1.15890411 | 0 | 8.778099476 | High groups |
| TCGA-51-4079-01 | 0.032876712 | 0 | 9.458286845 | High groups |
| TCGA-51-4080-01 | 0.032876712 | 0 | 8.316970331 | Low groups |
| TCGA-51-4081-01 | 2.495890411 | 0 | 8.945610337 | High groups |
| TCGA-51-6867-01 | 2.15890411 | 1 | 9.731404487 | High groups |
| TCGA-52-7622-01 | 2.361643836 | 0 | 8.548590057 | Low groups |
| TCGA-52-7809-01 | 0.454794521 | 1 | 9.061185257 | High groups |
| TCGA-52-7810-01 | 2.528767123 | 0 | 8.826811962 | High groups |
| TCGA-52-7811-01 | 0.419178082 | 1 | 8.039075154 | Low groups |
| TCGA-52-7812-01 | 2.167123288 | 1 | 8.753727035 | High groups |
| TCGA-56-1622-01 | 2.41369863 | 1 | 9.299274266 | High groups |
| TCGA-56-5897-01 | 1.035616438 | 0 | 8.514286803 | Low groups |
| TCGA-56-5898-01 | 1.520547945 | 0 | 8.717355739 | High groups |
| TCGA-56-6545-01 | 1.824657534 | 0 | 9.013131697 | High groups |
| TCGA-56-7221-01 | 1.665753425 | 0 | 8.006590102 | Low groups |
| TCGA-56-7222-01 | 1.169863014 | 1 | 9.413451918 | High groups |
| TCGA-56-7223-01 | 0.361643836 | 1 | 9.451818565 | High groups |
| TCGA-56-7579-01 | 0.408219178 | 1 | 9.206747141 | High groups |
| TCGA-56-7580-01 | 2.534246575 | 0 | 9.104810831 | High groups |
| TCGA-56-7582-01 | 1.646575342 | 0 | 8.605622373 | High groups |
| TCGA-56-7730-01 | 0.542465753 | 1 | 9.316045476 | High groups |
| TCGA-56-7731-01 | 0.008219178 | 0 | 8.903594844 | High groups |
| TCGA-56-7822-01 | 0.824657534 | 1 | 8.408445322 | Low groups |
| TCGA-56-7823-01 | 2.769863014 | 0 | 9.392470916 | High groups |
| TCGA-56-8082-01 | 1.246575342 | 0 | 10.04368366 | High groups |
| TCGA-56-8083-01 | 0.410958904 | 0 | 8.85190876 | High groups |
| TCGA-56-8201-01 | 1.087671233 | 1 | 8.329924572 | Low groups |
| TCGA-56-8304-01 | 0.290410959 | 0 | 7.920868798 | Low groups |
| TCGA-56-8305-01 | 0.287671233 | 0 | 7.666720808 | Low groups |
| TCGA-56-8307-01 | 2.24109589 | 0 | 7.969731576 | Low groups |
| TCGA-56-8308-01 | 1.416438356 | 0 | 8.5926735 | High groups |
| TCGA-56-8309-01 | 1.17260274 | 0 | 8.635666251 | High groups |
| TCGA-56-8503-01 | 0.112328767 | 0 | 8.195390418 | Low groups |
| TCGA-56-8504-01 | 1.397260274 | 0 | 8.640346464 | High groups |
| TCGA-56-8622-01 | 0.150684932 | 0 | 7.761569316 | Low groups |
| TCGA-56-8623-01 | 1.767123288 | 1 | 9.395510465 | High groups |
| TCGA-56-8624-01 | 1.150684932 | 0 | 8.768545681 | High groups |
| TCGA-56-8625-01 | 0.745205479 | 1 | 8.931114961 | High groups |
| TCGA-56-8626-01 | 0.82739726 | 0 | 9.014314756 | High groups |
| TCGA-56-8628-01 | 1.687671233 | 0 | 8.900888086 | High groups |
| TCGA-56-8629-01 | 1.317808219 | 0 | 8.208366841 | Low groups |
| TCGA-56-A49D-01 | 1.745205479 | 0 | 9.101072884 | High groups |
| TCGA-56-A4BW-01 | 1.602739726 | 0 | 8.394449104 | Low groups |
| TCGA-56-A4BX-01 | 1.109589041 | 0 | 9.599277433 | High groups |
| TCGA-56-A4BY-01 | 1.487671233 | 1 | 8.561054116 | Low groups |
| TCGA-56-A4ZJ-01 | 1.753424658 | 0 | 7.941849305 | Low groups |
| TCGA-56-A4ZK-01 | 1.561643836 | 0 | 8.886920528 | High groups |
| TCGA-56-A5DR-01 | 0.010958904 | 0 | 8.79646317 | High groups |
| TCGA-56-A62T-01 | 1.205479452 | 0 | 7.615361854 | Low groups |
| TCGA-58-8386-01 | 0.002739726 | 0 | 8.265519786 | Low groups |
| TCGA-58-8387-01 | 1.104109589 | 0 | 8.283707444 | Low groups |
| TCGA-58-8388-01 | 1.128767123 | 0 | 9.594042943 | High groups |
| TCGA-58-8390-01 | 2.495890411 | 0 | 9.071679019 | High groups |
| TCGA-58-8391-01 | 5.569863014 | 1 | 10.38871133 | High groups |
| TCGA-58-8392-01 | 0.263013699 | 1 | 8.762875411 | High groups |
| TCGA-58-8393-01 | 2.898630137 | 0 | 9.154428149 | High groups |
| TCGA-58-A46J-01 | 7.093150685 | 0 | 8.210718094 | Low groups |
| TCGA-58-A46K-01 | 2.01369863 | 1 | 7.8472111 | Low groups |
| TCGA-58-A46L-01 | 4.720547945 | 0 | 8.270898446 | Low groups |
| TCGA-58-A46M-01 | 2.936986301 | 0 | 9.116005473 | High groups |
| TCGA-58-A46N-01 | 1.726027397 | 1 | 9.067940627 | High groups |
| TCGA-60-2695-01 | 1.75890411 | 0 | 9.464772704 | High groups |
| TCGA-60-2696-01 | 0.298630137 | 0 | 9.611911598 | High groups |
| TCGA-60-2697-01 | 0.547945205 | 1 | 7.632376559 | Low groups |
| TCGA-60-2698-01 | 0.312328767 | 1 | 7.471920899 | Low groups |
| TCGA-60-2703-01 | 4.81369863 | 1 | 8.110490096 | Low groups |
| TCGA-60-2704-01 | 1.457534247 | 1 | 8.995632681 | High groups |
| TCGA-60-2706-01 | 7.726027397 | 0 | 7.718738349 | Low groups |
| TCGA-60-2708-01 | 6.704109589 | 0 | 8.129725135 | Low groups |
| TCGA-60-2709-01 | 4.123287671 | 0 | 9.031341983 | High groups |
| TCGA-60-2710-01 | 5.545205479 | 0 | 9.824423715 | High groups |
| TCGA-60-2711-01 | 3.452054795 | 0 | 9.594250209 | High groups |
| TCGA-60-2712-01 | 0.750684932 | 0 | 8.688513503 | High groups |
| TCGA-60-2713-01 | 3.715068493 | 1 | 8.685861223 | High groups |
| TCGA-60-2714-01 | 4.194520548 | 0 | 7.908451528 | Low groups |
| TCGA-60-2716-01 | 4.04109589 | 0 | 8.53484756 | Low groups |
| TCGA-60-2719-01 | 3.553424658 | 0 | 9.797968512 | High groups |
| TCGA-60-2720-01 | 0.265753425 | 0 | 7.339508614 | Low groups |
| TCGA-60-2721-01 | 2.693150685 | 0 | 8.110418788 | Low groups |
| TCGA-60-2722-01 | 2.331506849 | 1 | 9.53949036 | High groups |
| TCGA-60-2723-01 | 2.991780822 | 0 | 8.225705075 | Low groups |
| TCGA-60-2724-01 | 1.964383562 | 0 | 8.173035463 | Low groups |
| TCGA-60-2725-01 | 2.235616438 | 0 | 8.309473307 | Low groups |
| TCGA-63-6202-01 | 4.389041096 | 0 | 8.88768793 | High groups |
| TCGA-63-7020-01 | 5.843835616 | 0 | 8.897988146 | High groups |
| TCGA-63-7021-01 | 4.117808219 | 1 | 9.083158734 | High groups |
| TCGA-63-7022-01 | 5.679452055 | 0 | 8.587159209 | Low groups |
| TCGA-63-A5M9-01 | 0 | 0 | 7.889114649 | Low groups |
| TCGA-63-A5MB-01 | 8.556164384 | 0 | 8.781809731 | High groups |
| TCGA-63-A5MG-01 | 5.884931507 | 0 | 7.610917495 | Low groups |
| TCGA-63-A5MH-01 | 5.550684932 | 0 | 8.197688257 | Low groups |
| TCGA-63-A5MI-01 | 4.887671233 | 0 | 7.941193542 | Low groups |
| TCGA-63-A5MJ-01 | 4.997260274 | 0 | 8.487849116 | Low groups |
| TCGA-63-A5ML-01 | 3.797260274 | 0 | 8.756774361 | High groups |
| TCGA-63-A5MM-01 | 0.597260274 | 1 | 7.944394179 | Low groups |
| TCGA-63-A5MN-01 | 0.876712329 | 1 | 8.084955944 | Low groups |
| TCGA-63-A5MP-01 | 1.4 | 1 | 8.644302563 | High groups |
| TCGA-63-A5MR-01 | 7.44109589 | 0 | 8.582189901 | Low groups |
| TCGA-63-A5MS-01 | 6.523287671 | 0 | 8.603161196 | High groups |
| TCGA-63-A5MT-01 | 1.169863014 | 1 | 9.239519292 | High groups |
| TCGA-63-A5MU-01 | 1.150684932 | 1 | 9.001119345 | High groups |
| TCGA-63-A5MV-01 | 3.01369863 | 0 | 7.586079706 | Low groups |
| TCGA-63-A5MW-01 | 4.490410959 | 0 | 8.544350259 | Low groups |
| TCGA-63-A5MY-01 | 2.882191781 | 0 | 7.761837283 | Low groups |
| TCGA-66-2727-01 | 1.41369863 | 0 | 8.776701707 | High groups |
| TCGA-66-2734-01 | 3.591780822 | 0 | 8.278572674 | Low groups |
| TCGA-66-2737-01 | 0.167123288 | 0 | 7.827476153 | Low groups |
| TCGA-66-2742-01 | 1.756164384 | 0 | 7.533943594 | Low groups |
| TCGA-66-2744-01 | 0.082191781 | 0 | 6.843084642 | Low groups |
| TCGA-66-2753-01 | 0.084931507 | 0 | 8.45718016 | Low groups |
| TCGA-66-2754-01 | 0.167123288 | 0 | 6.398621432 | Low groups |
| TCGA-66-2755-01 | 0.076712329 | 0 | 8.712408175 | High groups |
| TCGA-66-2758-01 | 1.750684932 | 0 | 8.75202278 | High groups |
| TCGA-66-2759-01 | 2.087671233 | 0 | 8.834390238 | High groups |
| TCGA-66-2763-01 | 0.082191781 | 0 | 8.762058181 | High groups |
| TCGA-66-2765-01 | 0.167123288 | 0 | 8.662586849 | High groups |
| TCGA-66-2766-01 | 0.084931507 | 0 | 9.073148499 | High groups |
| TCGA-66-2767-01 | 0.167123288 | 0 | 8.143925596 | Low groups |
| TCGA-66-2768-01 | 0.167123288 | 0 | 9.370720401 | High groups |
| TCGA-66-2770-01 | 1.917808219 | 0 | 9.017956138 | High groups |
| TCGA-66-2771-01 | 1.583561644 | 0 | 7.332371385 | Low groups |
| TCGA-66-2777-01 | 0.167123288 | 0 | 8.385323608 | Low groups |
| TCGA-66-2780-01 | 1.002739726 | 0 | 8.616886119 | High groups |
| TCGA-66-2781-01 | 0.331506849 | 0 | 7.589157569 | Low groups |
| TCGA-66-2782-01 | 0.832876712 | 1 | 7.843398195 | Low groups |
| TCGA-66-2783-01 | 2.079452055 | 0 | 8.866259843 | High groups |
| TCGA-66-2785-01 | 0.164383562 | 0 | 7.911919052 | Low groups |
| TCGA-66-2786-01 | 2.164383562 | 0 | 9.521705743 | High groups |
| TCGA-66-2787-01 | 3.334246575 | 0 | 8.741955229 | High groups |
| TCGA-66-2788-01 | 1.915068493 | 0 | 7.688033521 | Low groups |
| TCGA-66-2789-01 | 0.336986301 | 0 | 8.235289696 | Low groups |
| TCGA-66-2790-01 | 1.915068493 | 1 | 7.698618415 | Low groups |
| TCGA-66-2791-01 | 0.419178082 | 0 | 8.728192806 | High groups |
| TCGA-66-2792-01 | 2.501369863 | 0 | 8.75126895 | High groups |
| TCGA-66-2793-01 | 0.838356164 | 0 | 8.395164493 | Low groups |
| TCGA-66-2794-01 | 4.506849315 | 0 | 8.008018558 | Low groups |
| TCGA-66-2795-01 | 0.334246575 | 0 | 8.619346245 | High groups |
| TCGA-66-2800-01 | 4.087671233 | 0 | 10.3082323 | High groups |
| TCGA-68-7755-01 | 0.22739726 | 0 | 9.043440478 | High groups |
| TCGA-68-7756-01 | 0.553424658 | 0 | 8.74746058 | High groups |
| TCGA-68-7757-01 | 0.578082192 | 0 | 7.48070192 | Low groups |
| TCGA-68-8250-01 | 0.668493151 | 0 | 8.754517096 | High groups |
| TCGA-68-8251-01 | 1.112328767 | 0 | 7.960991837 | Low groups |
| TCGA-68-A59I-01 | 0.435616438 | 1 | 7.777290761 | Low groups |
| TCGA-68-A59J-01 | 1.22739726 | 0 | 8.09588329 | Low groups |
| TCGA-70-6722-01 | 0.802739726 | 1 | 7.841716676 | Low groups |
| TCGA-70-6723-01 | 0.854794521 | 1 | 10.13011825 | High groups |
| TCGA-77-6842-01 | 0.550684932 | 1 | 9.558497821 | High groups |
| TCGA-77-6843-01 | 6.093150685 | 0 | 9.484710434 | High groups |
| TCGA-77-6844-01 | 4.898630137 | 1 | 9.376726224 | High groups |
| TCGA-77-6845-01 | 1.939726027 | 0 | 9.668424985 | High groups |
| TCGA-77-7138-01 | 0.931506849 | 0 | 8.308290151 | Low groups |
| TCGA-77-7139-01 | 11.6739726 | 0 | 8.953599854 | High groups |
| TCGA-77-7140-01 | 0.684931507 | 1 | 9.795261739 | High groups |
| TCGA-77-7141-01 | 0.04109589 | 0 | 9.211001823 | High groups |
| TCGA-77-7142-01 | 6.101369863 | 0 | 9.532148283 | High groups |
| TCGA-77-7335-01 | 5.843835616 | 0 | 8.411836352 | Low groups |
| TCGA-77-7337-01 | 8.912328767 | 0 | 8.668317365 | High groups |
| TCGA-77-7338-01 | 0.01369863 | 0 | 8.834062044 | High groups |
| TCGA-77-7463-01 | 3.898630137 | 0 | 9.050848698 | High groups |
| TCGA-77-7465-01 | 2.712328767 | 0 | 8.242985897 | Low groups |
| TCGA-77-8007-01 | 0.380821918 | 1 | 8.993333289 | High groups |
| TCGA-77-8008-01 | 7.230136986 | 0 | 7.784735078 | Low groups |
| TCGA-77-8009-01 | 1.663013699 | 1 | 9.129853387 | High groups |
| TCGA-77-8128-01 | 3.150684932 | 0 | 8.828358664 | High groups |
| TCGA-77-8130-01 | 12.24931507 | 1 | 8.207612853 | Low groups |
| TCGA-77-8131-01 | 1.049315068 | 0 | 7.698730916 | Low groups |
| TCGA-77-8133-01 | 4.493150685 | 0 | 8.521172671 | Low groups |
| TCGA-77-8136-01 | 0.824657534 | 1 | 8.273002482 | Low groups |
| TCGA-77-8138-01 | 0.791780822 | 1 | 8.135751553 | Low groups |
| TCGA-77-8139-01 | 8.673972603 | 0 | 8.247799374 | Low groups |
| TCGA-77-8140-01 | 0.961643836 | 0 | 8.672377852 | High groups |
| TCGA-77-8143-01 | 2.2 | 1 | 9.387717303 | High groups |
| TCGA-77-8144-01 | 2.282191781 | 0 | 8.912446582 | High groups |
| TCGA-77-8145-01 | 0.37260274 | 1 | 8.615929069 | High groups |
| TCGA-77-8146-01 | 8.736986301 | 0 | 8.09718022 | Low groups |
| TCGA-77-8148-01 | 5.542465753 | 0 | 8.682216336 | High groups |
| TCGA-77-8150-01 | 3.501369863 | 1 | 8.742395776 | High groups |
| TCGA-77-8153-01 | 5.457534247 | 0 | 10.09382371 | High groups |
| TCGA-77-8154-01 | 5.043835616 | 0 | 8.595353347 | High groups |
| TCGA-77-8156-01 | 3.030136986 | 0 | 8.205687065 | Low groups |
| TCGA-77-A5FZ-01 | 10.51506849 | 0 | 8.051323482 | Low groups |
| TCGA-77-A5G1-01 | 6.019178082 | 1 | 7.824761044 | Low groups |
| TCGA-77-A5G3-01 | 12.52054795 | 0 | 8.605673482 | High groups |
| TCGA-77-A5G6-01 | 1.073972603 | 1 | 8.72707412 | High groups |
| TCGA-77-A5G7-01 | 0.493150685 | 0 | 7.415410692 | Low groups |
| TCGA-77-A5G8-01 | 5.161643836 | 0 | 8.953947482 | High groups |
| TCGA-77-A5GA-01 | 3.506849315 | 0 | 7.976417083 | Low groups |
| TCGA-77-A5GB-01 | 0.62739726 | 1 | 7.5457698 | Low groups |
| TCGA-77-A5GF-01 | 1.978082192 | 1 | 7.80143237 | Low groups |
| TCGA-77-A5GH-01 | 3.238356164 | 0 | 8.071794091 | Low groups |
| TCGA-85-6175-01 | 0.805479452 | 1 | 8.59588825 | High groups |
| TCGA-85-6560-01 | 3.449315068 | 0 | 8.922500024 | High groups |
| TCGA-85-6561-01 | 3.353424658 | 0 | 8.749140456 | High groups |
| TCGA-85-6798-01 | 0.493150685 | 1 | 9.884876839 | High groups |
| TCGA-85-7696-01 | 3.043835616 | 0 | 8.442456711 | Low groups |
| TCGA-85-7697-01 | 2.912328767 | 0 | 8.190044207 | Low groups |
| TCGA-85-7698-01 | 1.780821918 | 1 | 8.975491333 | High groups |
| TCGA-85-7699-01 | 2.082191781 | 1 | 8.710259621 | High groups |
| TCGA-85-7710-01 | 0.115068493 | 0 | 9.209870761 | High groups |
| TCGA-85-7843-01 | 0.095890411 | 0 | 8.687011123 | High groups |
| TCGA-85-7844-01 | 2.495890411 | 0 | 9.025889513 | High groups |
| TCGA-85-7950-01 | 1.578082192 | 0 | 8.359591536 | Low groups |
| TCGA-85-8048-01 | 2.095890411 | 0 | 8.162794119 | Low groups |
| TCGA-85-8049-01 | 1.58630137 | 0 | 8.097515042 | Low groups |
| TCGA-85-8052-01 | 2.010958904 | 0 | 8.958046944 | High groups |
| TCGA-85-8070-01 | 2.630136986 | 0 | 9.331523982 | High groups |
| TCGA-85-8071-01 | 2.232876712 | 0 | 10.12880376 | High groups |
| TCGA-85-8072-01 | 2.553424658 | 0 | 9.251340241 | High groups |
| TCGA-85-8276-01 | 2.871232877 | 1 | 8.452123307 | Low groups |
| TCGA-85-8287-01 | 0.063013699 | 0 | 8.304122663 | Low groups |
| TCGA-85-8288-01 | 1.101369863 | 0 | 7.505748923 | Low groups |
| TCGA-85-8350-01 | 1.871232877 | 0 | 7.754223376 | Low groups |
| TCGA-85-8351-01 | 1.397260274 | 0 | 7.589665302 | Low groups |
| TCGA-85-8352-01 | 0.350684932 | 1 | 8.149332183 | Low groups |
| TCGA-85-8353-01 | 0.257534247 | 1 | 8.636088984 | High groups |
| TCGA-85-8354-01 | 2.726027397 | 0 | 10.36524722 | High groups |
| TCGA-85-8355-01 | 0.167123288 | 0 | 9.24540011 | High groups |
| TCGA-85-8479-01 | 1.282191781 | 0 | 10.26562442 | High groups |
| TCGA-85-8481-01 | 0.646575342 | 0 | 8.360649943 | Low groups |
| TCGA-85-8580-01 | 3.049315068 | 0 | 9.380348069 | High groups |
| TCGA-85-8582-01 | 3.178082192 | 0 | 8.639038679 | High groups |
| TCGA-85-8584-01 | 1.049315068 | 0 | 8.038161622 | Low groups |
| TCGA-85-8664-01 | 1.189041096 | 1 | 8.492349779 | Low groups |
| TCGA-85-8666-01 | 1.882191781 | 0 | 8.262763124 | Low groups |
| TCGA-85-A4CL-01 | 2.523287671 | 0 | 7.830911153 | Low groups |
| TCGA-85-A4CN-01 | 2.843835616 | 0 | 9.107951028 | High groups |
| TCGA-85-A4JB-01 | 2.580821918 | 0 | 7.338955165 | Low groups |
| TCGA-85-A4JC-01 | 1.501369863 | 1 | 7.186049645 | Low groups |
| TCGA-85-A4PA-01 | 2.030136986 | 0 | 8.464860311 | Low groups |
| TCGA-85-A4QQ-01 | 2.539726027 | 0 | 7.807954546 | Low groups |
| TCGA-85-A4QR-01 | 1.643835616 | 0 | 8.695845264 | High groups |
| TCGA-85-A50M-01 | 2.263013699 | 0 | 7.714201893 | Low groups |
| TCGA-85-A50Z-01 | 1.265753425 | 1 | 8.546174553 | Low groups |
| TCGA-85-A510-01 | 0.994520548 | 1 | 7.361755466 | Low groups |
| TCGA-85-A511-01 | 1.068493151 | 1 | 7.638522002 | Low groups |
| TCGA-85-A512-01 | 1.273972603 | 0 | 8.974975898 | High groups |
| TCGA-85-A513-01 | 2.493150685 | 0 | 7.832526637 | Low groups |
| TCGA-85-A53L-01 | 1.032876712 | 0 | 7.889263864 | Low groups |
| TCGA-85-A5B5-01 | 0.304109589 | 0 | 8.952580955 | High groups |
| TCGA-90-6837-01 | 2.076712329 | 0 | 9.595575455 | High groups |
| TCGA-90-7766-01 | 0.791780822 | 1 | 8.835828662 | High groups |
| TCGA-90-7767-01 | 0.243835616 | 0 | 8.770757537 | High groups |
| TCGA-90-7769-01 | 0.980821918 | 0 | 8.909520874 | High groups |
| TCGA-90-7964-01 | 1.17260274 | 0 | 8.200256175 | Low groups |
| TCGA-90-A4ED-01 | 1.684931507 | 0 | 8.89977834 | High groups |
| TCGA-90-A4EE-01 | 1.884931507 | 0 | 7.906560181 | Low groups |
| TCGA-90-A59Q-01 | 0.750684932 | 1 | 7.662045239 | Low groups |
| TCGA-92-7340-01 | 0.224657534 | 0 | 7.712157596 | Low groups |
| TCGA-92-7341-01 | 0.290410959 | 0 | 8.634629386 | High groups |
| TCGA-92-8063-01 | 0.334246575 | 0 | 9.55983218 | High groups |
| TCGA-92-8064-01 | 0.438356164 | 0 | 8.593049131 | High groups |
| TCGA-92-8065-01 | 0.191780822 | 0 | 8.843489905 | High groups |
| TCGA-94-7033-01 | 1.753424658 | 0 | 8.226787148 | Low groups |
| TCGA-94-7557-01 | 0.01369863 | 0 | 8.327110225 | Low groups |
| TCGA-94-7943-01 | 1.523287671 | 1 | 7.361860542 | Low groups |
| TCGA-94-8035-01 | 0.334246575 | 0 | 8.71997834 | High groups |
| TCGA-94-8490-01 | 0.419178082 | 0 | 9.407550539 | High groups |
| TCGA-94-8491-01 | 1.756164384 | 1 | 8.55283737 | Low groups |
| TCGA-94-A4VJ-01 | 1.178082192 | 0 | 8.280957334 | Low groups |
| TCGA-94-A5I4-01 | 0.860273973 | 1 | 8.62732259 | High groups |
| TCGA-94-A5I6-01 | 1.317808219 | 1 | 7.936518691 | Low groups |
| TCGA-96-7544-01 | 5.917808219 | 0 | 7.847191553 | Low groups |
| TCGA-96-7545-01 | 4.638356164 | 1 | 8.30800872 | Low groups |
| TCGA-96-8169-01 | 1.526027397 | 0 | 8.194649855 | Low groups |
| TCGA-96-8170-01 | 1.454794521 | 0 | 8.343931593 | Low groups |
| TCGA-96-A4JK-01 | 1.61369863 | 0 | 8.398269683 | Low groups |
| TCGA-96-A4JL-01 | 2.306849315 | 0 | 7.624461158 | Low groups |
| TCGA-98-7454-01 | 1.78630137 | 0 | 8.296014527 | Low groups |
| TCGA-98-8020-01 | 0.030136986 | 1 | 7.912152715 | Low groups |
| TCGA-98-8021-01 | 2.515068493 | 1 | 8.737797744 | High groups |
| TCGA-98-8022-01 | 2.556164384 | 0 | 7.609151399 | Low groups |
| TCGA-98-8023-01 | 1.778082192 | 0 | 8.209826079 | Low groups |
| TCGA-98-A538-01 | 2.263013699 | 0 | 8.294363292 | Low groups |
| TCGA-98-A539-01 | 0.473972603 | 1 | 7.90011466 | Low groups |
| TCGA-98-A53A-01 | 1.512328767 | 0 | 9.292542967 | High groups |
| TCGA-98-A53B-01 | 0.167123288 | 0 | 6.8979336 | Low groups |
| TCGA-98-A53C-01 | 2.252054795 | 0 | 8.260717989 | Low groups |
| TCGA-98-A53D-01 | 1.131506849 | 1 | 8.328910182 | Low groups |
| TCGA-98-A53H-01 | 1.169863014 | 1 | 7.767109381 | Low groups |
| TCGA-98-A53I-01 | 1.547945205 | 0 | 7.859046206 | Low groups |
| TCGA-98-A53J-01 | 1.726027397 | 0 | 7.609400464 | Low groups |
| TCGA-J1-A4AH-01 | 1.591780822 | 0 | 7.999495983 | Low groups |
| TCGA-L3-A4E7-01 | 1.073972603 | 0 | 8.48662207 | Low groups |
| TCGA-L3-A524-01 | 1.342465753 | 0 | 9.030510886 | High groups |
| TCGA-LA-A446-01 | 1.098630137 | 0 | 8.747856293 | High groups |
| TCGA-LA-A7SW-01 | 0.643835616 | 1 | 7.385391086 | Low groups |
| TCGA-MF-A522-01 | 0.473972603 | 1 | 8.893713133 | High groups |
| TCGA-NC-A5HD-01 | 0.005479452 | 0 | 9.159799301 | High groups |
| TCGA-NC-A5HE-01 | 6.4 | 0 | 8.624639757 | High groups |
| TCGA-NC-A5HF-01 | 0.361643836 | 1 | 9.024416926 | High groups |
| TCGA-NC-A5HG-01 | 5.378082192 | 0 | 8.223181276 | Low groups |
| TCGA-NC-A5HH-01 | 0.101369863 | 0 | 7.156991549 | Low groups |
| TCGA-NC-A5HI-01 | 0.191780822 | 1 | 7.659681854 | Low groups |
| TCGA-NC-A5HJ-01 | 0.898630137 | 1 | 8.816564957 | High groups |
| TCGA-NC-A5HK-01 | 0.350684932 | 0 | 8.588675064 | High groups |
| TCGA-NC-A5HL-01 | 0.24109589 | 0 | 8.619128868 | High groups |
| TCGA-NC-A5HM-01 | 3.320547945 | 0 | 8.641167568 | High groups |
| TCGA-NC-A5HN-01 | 4.106849315 | 0 | 8.76429871 | High groups |
| TCGA-NC-A5HO-01 | 3.660273973 | 0 | 8.282755592 | Low groups |
| TCGA-NC-A5HP-01 | 0.868493151 | 1 | 8.060226844 | Low groups |
| TCGA-NC-A5HQ-01 | 1.22739726 | 0 | 8.028044732 | Low groups |
| TCGA-NC-A5HR-01 | 3.408219178 | 0 | 9.179456137 | High groups |
| TCGA-NC-A5HT-01 | 0.556164384 | 1 | 7.539541617 | Low groups |
| TCGA-NK-A5CR-01 | 6.964383562 | 0 | 9.140112718 | High groups |
| TCGA-NK-A5CT-01 | 5.452054795 | 1 | 7.786074205 | Low groups |
| TCGA-NK-A5CX-01 | 0.304109589 | 0 | 8.042309833 | Low groups |
| TCGA-NK-A5D1-01 | 0.41369863 | 1 | 8.434512307 | Low groups |
| TCGA-NK-A7XE-01 | 0.035616438 | 0 | 8.517344755 | Low groups |
| TCGA-O2-A52N-01 | 2.756164384 | 0 | 7.858147704 | Low groups |
| TCGA-O2-A52Q-01 | 0.238356164 | 1 | 7.383216112 | Low groups |
| TCGA-O2-A52S-01 | 0.673972603 | 1 | 9.403831591 | High groups |
| TCGA-O2-A52V-01 | 1.890410959 | 1 | 7.085197093 | Low groups |
| TCGA-O2-A52W-01 | 0.715068493 | 0 | 7.754812293 | Low groups |
| TCGA-O2-A5IB-01 | 0.693150685 | 1 | 7.85475502 | Low groups |
| TCGA-XC-AA0X-01 | 0.016438356 | 0 | 8.902660007 | High groups |

**Table S11.** Raw counts of RNA-sequencing data of hsa-miR-186-5p in LUSC from the TCGA.

| sampleID | time | Staus | RS | Label |
| --- | --- | --- | --- | --- |
| TCGA-18-3406-01 | 0.978082192 | 1 | 8.434442542 | High groups |
| TCGA-18-3407-01 | 0.37260274 | 0 | 9.035621985 | High groups |
| TCGA-18-3408-01 | 4.912328767 | 1 | 8.243855175 | High groups |
| TCGA-18-3410-01 | 0.4 | 0 | 9.580419884 | High groups |
| TCGA-18-3411-01 | 9.797260274 | 0 | 8.752515459 | High groups |
| TCGA-18-3412-01 | 0.747945205 | 1 | 8.193087336 | High groups |
| TCGA-18-3414-01 | 1.961643836 | 0 | 8.553598194 | High groups |
| TCGA-18-3415-01 | 7.679452055 | 0 | 8.663323127 | High groups |
| TCGA-18-3416-01 | 2.575342466 | 1 | 9.64099375 | High groups |
| TCGA-18-3417-01 | 3.005479452 | 0 | 7.873043117 | Low groups |
| TCGA-18-3419-01 | 7.701369863 | 0 | 9.593071734 | High groups |
| TCGA-18-3421-01 | 7.246575342 | 0 | 8.988755116 | High groups |
| TCGA-18-4086-01 | 0.232876712 | 0 | 8.725350919 | High groups |
| TCGA-18-4721-01 | 12.86027397 | 0 | 8.5192613 | High groups |
| TCGA-18-5592-01 | 4.161643836 | 0 | 8.298006463 | High groups |
| TCGA-18-5595-01 | 2.265753425 | 0 | 9.494550217 | High groups |
| TCGA-21-1072-01 | 8.263013699 | 0 | 8.877470958 | High groups |
| TCGA-21-1075-01 | 5.846575342 | 0 | 8.687468909 | High groups |
| TCGA-21-1076-01 | 4.315068493 | 1 | 8.961886199 | High groups |
| TCGA-21-1077-01 | 1.764383562 | 1 | 8.616137001 | High groups |
| TCGA-21-1078-01 | 0.704109589 | 1 | 8.726290922 | High groups |
| TCGA-21-1079-01 | 0.868493151 | 1 | 7.685846264 | Low groups |
| TCGA-21-1080-01 | 10.20273973 | 0 | 9.040069669 | High groups |
| TCGA-21-1083-01 | 3.602739726 | 0 | 8.179173626 | Low groups |
| TCGA-21-5782-01 | 2.635616438 | 0 | 9.197837754 | High groups |
| TCGA-21-5783-01 | 7.342465753 | 0 | 7.894499669 | Low groups |
| TCGA-21-5784-01 | 3.473972603 | 0 | 8.534320004 | High groups |
| TCGA-21-5786-01 | 2.238356164 | 1 | 9.209801666 | High groups |
| TCGA-21-5787-01 | 0.282191781 | 1 | 10.55409361 | High groups |
| TCGA-21-A5DI-01 | 2.682191781 | 0 | 7.92728434 | Low groups |
| TCGA-22-0940-01 | 1.832876712 | 1 | 8.509208502 | High groups |
| TCGA-22-0944-01 | 0.610958904 | 0 | 7.956707787 | Low groups |
| TCGA-22-1011-01 | 0.145205479 | 0 | 9.105856665 | High groups |
| TCGA-22-1012-01 | 1.175342466 | 0 | 8.391165942 | High groups |
| TCGA-22-4591-01 | 1.21369863 | 1 | 9.344712831 | High groups |
| TCGA-22-4593-01 | 2.923287671 | 0 | 8.107919757 | Low groups |
| TCGA-22-4594-01 | 4.02739726 | 0 | 8.545046589 | High groups |
| TCGA-22-4595-01 | 0.684931507 | 1 | 8.420191917 | High groups |
| TCGA-22-4596-01 | 0.046575342 | 0 | 8.188247899 | High groups |
| TCGA-22-4601-01 | 1.438356164 | 1 | 9.938726715 | High groups |
| TCGA-22-4604-01 | 0.838356164 | 1 | 8.521831252 | High groups |
| TCGA-22-4605-01 | 2.668493151 | 0 | 7.585915342 | Low groups |
| TCGA-22-4607-01 | 1.608219178 | 0 | 7.574623346 | Low groups |
| TCGA-22-4609-01 | 0.797260274 | 0 | 7.684987005 | Low groups |
| TCGA-22-4613-01 | 0.980821918 | 0 | 9.14386926 | High groups |
| TCGA-22-5471-01 | 0.62739726 | 1 | 8.838651174 | High groups |
| TCGA-22-5472-01 | 2.043835616 | 1 | 9.29876644 | High groups |
| TCGA-22-5473-01 | 4.534246575 | 1 | 8.282216452 | High groups |
| TCGA-22-5474-01 | 1.219178082 | 0 | 8.814098229 | High groups |
| TCGA-22-5477-01 | 1.4 | 1 | 9.015884723 | High groups |
| TCGA-22-5478-01 | 0.065753425 | 0 | 9.279908142 | High groups |
| TCGA-22-5479-01 | 7.191780822 | 0 | 7.897560518 | Low groups |
| TCGA-22-5480-01 | 4.873972603 | 1 | 9.07777467 | High groups |
| TCGA-22-5481-01 | 0.947945205 | 1 | 9.668519958 | High groups |
| TCGA-22-5482-01 | 0.978082192 | 0 | 9.415008913 | High groups |
| TCGA-22-5483-01 | 1.556164384 | 1 | 7.349773773 | Low groups |
| TCGA-22-5485-01 | 2.301369863 | 1 | 9.913725887 | High groups |
| TCGA-22-5489-01 | 1.17260274 | 1 | 9.084003655 | High groups |
| TCGA-22-5491-01 | 4.693150685 | 0 | 8.579553538 | High groups |
| TCGA-22-5492-01 | 1.38630137 | 0 | 9.566720607 | High groups |
| TCGA-22-A5C4-01 | 1.838356164 | 0 | 8.750362507 | High groups |
| TCGA-33-4532-01 | 10.75068493 | 0 | 9.50579925 | High groups |
| TCGA-33-4533-01 | 11.14520548 | 0 | 8.816927322 | High groups |
| TCGA-33-4538-01 | 8.161643836 | 0 | 8.535596234 | High groups |
| TCGA-33-4547-01 | 6.62739726 | 0 | 8.404913125 | High groups |
| TCGA-33-4566-01 | 14.48493151 | 0 | 9.016350424 | High groups |
| TCGA-33-4582-01 | 8.008219178 | 1 | 7.985708873 | Low groups |
| TCGA-33-4583-01 | 12.60547945 | 0 | 8.267269763 | High groups |
| TCGA-33-4586-01 | 0.463013699 | 1 | 7.904942183 | Low groups |
| TCGA-33-4587-01 | 4.536986301 | 1 | 7.317148834 | Low groups |
| TCGA-33-4589-01 | 0.128767123 | 0 | 8.482844654 | High groups |
| TCGA-33-6737-01 | 1.646575342 | 1 | 8.55292657 | High groups |
| TCGA-33-6738-01 | 5.279452055 | 0 | 8.85764497 | High groups |
| TCGA-33-A4WN-01 | 0.391780822 | 0 | 8.131872802 | Low groups |
| TCGA-33-A5GW-01 | 0.024657534 | 0 | 9.513412576 | High groups |
| TCGA-33-AAS8-01 | 3.052054795 | 0 | 7.928666412 | Low groups |
| TCGA-33-AASB-01 | 0.082191781 | 1 | 8.155156875 | Low groups |
| TCGA-33-AASD-01 | 5.238356164 | 1 | 7.828812948 | Low groups |
| TCGA-33-AASI-01 | 3.682191781 | 1 | 7.769640513 | Low groups |
| TCGA-33-AASJ-01 | 3.109589041 | 1 | 8.008431872 | Low groups |
| TCGA-33-AASL-01 | 0.408219178 | 1 | 7.166483679 | Low groups |
| TCGA-34-2596-01 | 0.219178082 | 0 | 8.446367364 | High groups |
| TCGA-34-2600-01 | 5.134246575 | 0 | 8.944757735 | High groups |
| TCGA-34-2604-01 | 2.624657534 | 0 | 8.700758394 | High groups |
| TCGA-34-2605-01 | 1.079452055 | 1 | 8.418642743 | High groups |
| TCGA-34-2608-01 | 2.739726027 | 0 | 8.248009754 | High groups |
| TCGA-34-2609-01 | 1.060273973 | 1 | 8.51942919 | High groups |
| TCGA-34-5231-01 | 5.435616438 | 0 | 7.813681097 | Low groups |
| TCGA-34-5232-01 | 6.769863014 | 0 | 8.400953148 | High groups |
| TCGA-34-5234-01 | 6.221917808 | 0 | 8.304742376 | High groups |
| TCGA-34-5236-01 | 0.309589041 | 1 | 7.408040196 | Low groups |
| TCGA-34-5239-01 | 4.191780822 | 1 | 7.699943568 | Low groups |
| TCGA-34-5240-01 | 4.221917808 | 0 | 8.778423696 | High groups |
| TCGA-34-5241-01 | 1.410958904 | 0 | 8.084105733 | Low groups |
| TCGA-34-5927-01 | 0.671232877 | 1 | 9.394950385 | High groups |
| TCGA-34-5928-01 | 3.276712329 | 0 | 7.910349395 | Low groups |
| TCGA-34-5929-01 | 0.41369863 | 0 | 8.516125489 | High groups |
| TCGA-34-7107-01 | 0.093150685 | 0 | 7.772880982 | Low groups |
| TCGA-34-8454-01 | 3.232876712 | 0 | 7.953907161 | Low groups |
| TCGA-34-8455-01 | 0.336986301 | 1 | 7.97381645 | Low groups |
| TCGA-34-8456-01 | 2.202739726 | 0 | 7.64025817 | Low groups |
| TCGA-34-A5IX-01 | 2.824657534 | 0 | 9.015214037 | High groups |
| TCGA-37-3783-01 | 0.334246575 | 0 | 7.393341544 | Low groups |
| TCGA-37-3789-01 | 0.035616438 | 0 | 9.575353178 | High groups |
| TCGA-37-3792-01 | 0.032876712 | 0 | 9.292840404 | High groups |
| TCGA-37-4130-01 | 0.676712329 | 0 | 9.249927391 | High groups |
| TCGA-37-4133-01 | 0.652054795 | 0 | 8.82758914 | High groups |
| TCGA-37-4135-01 | 0.567123288 | 0 | 9.080801542 | High groups |
| TCGA-37-4141-01 | 0.032876712 | 0 | 9.479905737 | High groups |
| TCGA-37-5819-01 | 0.282191781 | 0 | 8.82702777 | High groups |
| TCGA-37-A5EL-01 | 2.591780822 | 1 | 7.184744057 | Low groups |
| TCGA-37-A5EM-01 | 2.375342466 | 0 | 8.071039843 | Low groups |
| TCGA-37-A5EN-01 | 1.808219178 | 0 | 8.753081434 | High groups |
| TCGA-39-5011-01 | 1.547945205 | 1 | 8.952554194 | High groups |
| TCGA-39-5019-01 | 1.035616438 | 1 | 7.42165436 | Low groups |
| TCGA-39-5021-01 | 5.136986301 | 1 | 9.352055678 | High groups |
| TCGA-39-5022-01 | 3.967123288 | 1 | 8.286458421 | High groups |
| TCGA-39-5024-01 | 6.876712329 | 0 | 7.78713812 | Low groups |
| TCGA-39-5027-01 | 8.44109589 | 1 | 7.969704699 | Low groups |
| TCGA-39-5028-01 | 0.142465753 | 0 | 8.830375637 | High groups |
| TCGA-39-5029-01 | 1.345205479 | 1 | 7.803920314 | Low groups |
| TCGA-39-5030-01 | 0.161643836 | 0 | 8.531248648 | High groups |
| TCGA-39-5031-01 | 5.043835616 | 0 | 8.697287143 | High groups |
| TCGA-39-5034-01 | 0.964383562 | 1 | 8.576061019 | High groups |
| TCGA-39-5035-01 | 5.698630137 | 0 | 8.139099032 | Low groups |
| TCGA-39-5036-01 | 5.931506849 | 0 | 8.627351484 | High groups |
| TCGA-39-5037-01 | 4.630136986 | 0 | 8.773359139 | High groups |
| TCGA-39-5039-01 | 1.490410959 | 0 | 8.018019643 | Low groups |
| TCGA-39-5040-01 | 1.210958904 | 1 | 8.280132782 | High groups |
| TCGA-43-2576-01 | 3.350684932 | 0 | 8.387705242 | High groups |
| TCGA-43-2578-01 | 1.873972603 | 0 | 8.611948859 | High groups |
| TCGA-43-2581-01 | 3.221917808 | 0 | 7.994960203 | Low groups |
| TCGA-43-3394-01 | 1.512328767 | 1 | 9.43716978 | High groups |
| TCGA-43-3920-01 | 2.75890411 | 0 | 8.790278177 | High groups |
| TCGA-43-5668-01 | 1.287671233 | 1 | 9.887394947 | High groups |
| TCGA-43-5670-01 | 2.326027397 | 0 | 7.351044254 | Low groups |
| TCGA-43-6143-01 | 1.915068493 | 0 | 8.005650933 | Low groups |
| TCGA-43-6647-01 | 2.073972603 | 0 | 7.833406951 | Low groups |
| TCGA-43-6770-01 | 1.789041096 | 0 | 8.81841649 | High groups |
| TCGA-43-6771-01 | 0.421917808 | 1 | 7.691456766 | Low groups |
| TCGA-43-6773-01 | 0.317808219 | 0 | 8.584121134 | High groups |
| TCGA-43-7656-01 | 1.632876712 | 0 | 8.241777512 | High groups |
| TCGA-43-7657-01 | 0.646575342 | 0 | 7.081577387 | Low groups |
| TCGA-43-7658-01 | 6.315068493 | 1 | 8.087351293 | Low groups |
| TCGA-43-8115-01 | 1.115068493 | 0 | 8.056965935 | Low groups |
| TCGA-43-8116-01 | 0.980821918 | 0 | 8.220388389 | High groups |
| TCGA-43-8118-01 | 0.243835616 | 0 | 8.229511354 | High groups |
| TCGA-43-A474-01 | 0.967123288 | 0 | 7.26854069 | Low groups |
| TCGA-43-A475-01 | 0.810958904 | 0 | 8.460823639 | High groups |
| TCGA-43-A56U-01 | 1.183561644 | 0 | 8.515306197 | High groups |
| TCGA-43-A56V-01 | 0.97260274 | 1 | 7.856234962 | Low groups |
| TCGA-46-3765-01 | 1.109589041 | 0 | 9.728805025 | High groups |
| TCGA-46-3766-01 | 1.01369863 | 0 | 8.157884751 | Low groups |
| TCGA-46-3767-01 | 1.084931507 | 0 | 6.828491194 | Low groups |
| TCGA-46-3768-01 | 0.819178082 | 0 | 8.854344182 | High groups |
| TCGA-46-3769-01 | 0.369863014 | 0 | 8.960033856 | High groups |
| TCGA-46-6025-01 | 0.887671233 | 0 | 8.591726647 | High groups |
| TCGA-46-6026-01 | 1.15890411 | 0 | 8.527349934 | High groups |
| TCGA-51-4079-01 | 0.032876712 | 0 | 8.686823872 | High groups |
| TCGA-51-4080-01 | 0.032876712 | 0 | 8.052539034 | Low groups |
| TCGA-51-4081-01 | 2.495890411 | 0 | 7.813755671 | Low groups |
| TCGA-51-6867-01 | 2.15890411 | 1 | 8.105961921 | Low groups |
| TCGA-52-7622-01 | 2.361643836 | 0 | 7.791755607 | Low groups |
| TCGA-52-7809-01 | 0.454794521 | 1 | 8.721049104 | High groups |
| TCGA-52-7810-01 | 2.528767123 | 0 | 8.165182118 | Low groups |
| TCGA-52-7811-01 | 0.419178082 | 1 | 7.569983547 | Low groups |
| TCGA-52-7812-01 | 2.167123288 | 1 | 7.074197456 | Low groups |
| TCGA-56-1622-01 | 2.41369863 | 1 | 9.091439618 | High groups |
| TCGA-56-5897-01 | 1.035616438 | 0 | 8.665620235 | High groups |
| TCGA-56-5898-01 | 1.520547945 | 0 | 9.000536487 | High groups |
| TCGA-56-6545-01 | 1.824657534 | 0 | 7.781365419 | Low groups |
| TCGA-56-7221-01 | 1.665753425 | 0 | 6.868888717 | Low groups |
| TCGA-56-7222-01 | 1.169863014 | 1 | 7.596941849 | Low groups |
| TCGA-56-7223-01 | 0.361643836 | 1 | 8.22518256 | High groups |
| TCGA-56-7579-01 | 0.408219178 | 1 | 8.048361317 | Low groups |
| TCGA-56-7580-01 | 2.534246575 | 0 | 8.237705675 | High groups |
| TCGA-56-7582-01 | 1.646575342 | 0 | 6.9685349 | Low groups |
| TCGA-56-7730-01 | 0.542465753 | 1 | 7.266301283 | Low groups |
| TCGA-56-7731-01 | 0.008219178 | 0 | 8.049883119 | Low groups |
| TCGA-56-7822-01 | 0.824657534 | 1 | 7.246478396 | Low groups |
| TCGA-56-7823-01 | 2.769863014 | 0 | 7.6315835 | Low groups |
| TCGA-56-8082-01 | 1.246575342 | 0 | 8.330216105 | High groups |
| TCGA-56-8083-01 | 0.410958904 | 0 | 9.855126674 | High groups |
| TCGA-56-8201-01 | 1.087671233 | 1 | 7.999832615 | Low groups |
| TCGA-56-8304-01 | 0.290410959 | 0 | 7.728032463 | Low groups |
| TCGA-56-8305-01 | 0.287671233 | 0 | 7.780627688 | Low groups |
| TCGA-56-8307-01 | 2.24109589 | 0 | 8.690418667 | High groups |
| TCGA-56-8308-01 | 1.416438356 | 0 | 7.515364747 | Low groups |
| TCGA-56-8309-01 | 1.17260274 | 0 | 7.873893401 | Low groups |
| TCGA-56-8503-01 | 0.112328767 | 0 | 8.005144001 | Low groups |
| TCGA-56-8504-01 | 1.397260274 | 0 | 7.601349467 | Low groups |
| TCGA-56-8622-01 | 0.150684932 | 0 | 7.142279558 | Low groups |
| TCGA-56-8623-01 | 1.767123288 | 1 | 7.868507751 | Low groups |
| TCGA-56-8624-01 | 1.150684932 | 0 | 7.999742321 | Low groups |
| TCGA-56-8625-01 | 0.745205479 | 1 | 7.882844218 | Low groups |
| TCGA-56-8626-01 | 0.82739726 | 0 | 7.781933661 | Low groups |
| TCGA-56-8628-01 | 1.687671233 | 0 | 9.172900495 | High groups |
| TCGA-56-8629-01 | 1.317808219 | 0 | 8.737800988 | High groups |
| TCGA-56-A49D-01 | 1.745205479 | 0 | 9.174615644 | High groups |
| TCGA-56-A4BW-01 | 1.602739726 | 0 | 7.672417563 | Low groups |
| TCGA-56-A4BX-01 | 1.109589041 | 0 | 8.23265692 | High groups |
| TCGA-56-A4BY-01 | 1.487671233 | 1 | 8.374482887 | High groups |
| TCGA-56-A4ZJ-01 | 1.753424658 | 0 | 8.154457036 | Low groups |
| TCGA-56-A4ZK-01 | 1.561643836 | 0 | 8.269393541 | High groups |
| TCGA-56-A5DR-01 | 0.010958904 | 0 | 8.48231891 | High groups |
| TCGA-56-A62T-01 | 1.205479452 | 0 | 7.457519437 | Low groups |
| TCGA-58-8386-01 | 0.002739726 | 0 | 7.463930396 | Low groups |
| TCGA-58-8387-01 | 1.104109589 | 0 | 8.145165493 | Low groups |
| TCGA-58-8388-01 | 1.128767123 | 0 | 8.96739329 | High groups |
| TCGA-58-8390-01 | 2.495890411 | 0 | 8.699268093 | High groups |
| TCGA-58-8391-01 | 5.569863014 | 1 | 7.641735457 | Low groups |
| TCGA-58-8392-01 | 0.263013699 | 1 | 7.70126383 | Low groups |
| TCGA-58-8393-01 | 2.898630137 | 0 | 7.787853598 | Low groups |
| TCGA-58-A46J-01 | 7.093150685 | 0 | 8.50568494 | High groups |
| TCGA-58-A46K-01 | 2.01369863 | 1 | 7.817664696 | Low groups |
| TCGA-58-A46L-01 | 4.720547945 | 0 | 7.482680518 | Low groups |
| TCGA-58-A46M-01 | 2.936986301 | 0 | 7.659154354 | Low groups |
| TCGA-58-A46N-01 | 1.726027397 | 1 | 7.523134024 | Low groups |
| TCGA-60-2695-01 | 1.75890411 | 0 | 9.289594503 | High groups |
| TCGA-60-2696-01 | 0.298630137 | 0 | 9.251647619 | High groups |
| TCGA-60-2697-01 | 0.547945205 | 1 | 7.58153451 | Low groups |
| TCGA-60-2698-01 | 0.312328767 | 1 | 8.447731384 | High groups |
| TCGA-60-2703-01 | 4.81369863 | 1 | 7.154828739 | Low groups |
| TCGA-60-2704-01 | 1.457534247 | 1 | 7.558371883 | Low groups |
| TCGA-60-2706-01 | 7.726027397 | 0 | 8.782491392 | High groups |
| TCGA-60-2708-01 | 6.704109589 | 0 | 7.972307865 | Low groups |
| TCGA-60-2709-01 | 4.123287671 | 0 | 7.944430785 | Low groups |
| TCGA-60-2710-01 | 5.545205479 | 0 | 9.118658474 | High groups |
| TCGA-60-2711-01 | 3.452054795 | 0 | 9.771550329 | High groups |
| TCGA-60-2712-01 | 0.750684932 | 0 | 8.417934771 | High groups |
| TCGA-60-2713-01 | 3.715068493 | 1 | 8.897925906 | High groups |
| TCGA-60-2714-01 | 4.194520548 | 0 | 9.07338646 | High groups |
| TCGA-60-2716-01 | 4.04109589 | 0 | 8.616061737 | High groups |
| TCGA-60-2719-01 | 3.553424658 | 0 | 10.1018142 | High groups |
| TCGA-60-2720-01 | 0.265753425 | 0 | 8.573394599 | High groups |
| TCGA-60-2721-01 | 2.693150685 | 0 | 7.92540727 | Low groups |
| TCGA-60-2722-01 | 2.331506849 | 1 | 8.779381013 | High groups |
| TCGA-60-2723-01 | 2.991780822 | 0 | 9.033203277 | High groups |
| TCGA-60-2724-01 | 1.964383562 | 0 | 8.800845005 | High groups |
| TCGA-60-2725-01 | 2.235616438 | 0 | 9.002442342 | High groups |
| TCGA-63-6202-01 | 4.389041096 | 0 | 8.197641329 | High groups |
| TCGA-63-7020-01 | 5.843835616 | 0 | 8.249995017 | High groups |
| TCGA-63-7021-01 | 4.117808219 | 1 | 8.065186362 | Low groups |
| TCGA-63-7022-01 | 5.679452055 | 0 | 7.782734209 | Low groups |
| TCGA-63-A5M9-01 | 0 | 0 | 7.845712083 | Low groups |
| TCGA-63-A5MB-01 | 8.556164384 | 0 | 8.214318149 | High groups |
| TCGA-63-A5MG-01 | 5.884931507 | 0 | 7.97218745 | Low groups |
| TCGA-63-A5MH-01 | 5.550684932 | 0 | 9.314969837 | High groups |
| TCGA-63-A5MI-01 | 4.887671233 | 0 | 9.661986116 | High groups |
| TCGA-63-A5MJ-01 | 4.997260274 | 0 | 7.659547499 | Low groups |
| TCGA-63-A5ML-01 | 3.797260274 | 0 | 7.674251741 | Low groups |
| TCGA-63-A5MM-01 | 0.597260274 | 1 | 8.432535929 | High groups |
| TCGA-63-A5MN-01 | 0.876712329 | 1 | 8.094044159 | Low groups |
| TCGA-63-A5MP-01 | 1.4 | 1 | 8.402907046 | High groups |
| TCGA-63-A5MR-01 | 7.44109589 | 0 | 7.974833169 | Low groups |
| TCGA-63-A5MS-01 | 6.523287671 | 0 | 8.337141652 | High groups |
| TCGA-63-A5MT-01 | 1.169863014 | 1 | 8.190971539 | High groups |
| TCGA-63-A5MU-01 | 1.150684932 | 1 | 7.80954744 | Low groups |
| TCGA-63-A5MV-01 | 3.01369863 | 0 | 8.392707327 | High groups |
| TCGA-63-A5MW-01 | 4.490410959 | 0 | 8.453730741 | High groups |
| TCGA-63-A5MY-01 | 2.882191781 | 0 | 9.095466914 | High groups |
| TCGA-66-2727-01 | 1.41369863 | 0 | 9.640330628 | High groups |
| TCGA-66-2734-01 | 3.591780822 | 0 | 8.543252772 | High groups |
| TCGA-66-2737-01 | 0.167123288 | 0 | 8.032906843 | Low groups |
| TCGA-66-2742-01 | 1.756164384 | 0 | 8.199510891 | High groups |
| TCGA-66-2744-01 | 0.082191781 | 0 | 8.696821511 | High groups |
| TCGA-66-2753-01 | 0.084931507 | 0 | 9.70363819 | High groups |
| TCGA-66-2754-01 | 0.167123288 | 0 | 9.846387849 | High groups |
| TCGA-66-2755-01 | 0.076712329 | 0 | 8.466200888 | High groups |
| TCGA-66-2758-01 | 1.750684932 | 0 | 8.375952299 | High groups |
| TCGA-66-2759-01 | 2.087671233 | 0 | 8.91073548 | High groups |
| TCGA-66-2763-01 | 0.082191781 | 0 | 8.672230989 | High groups |
| TCGA-66-2765-01 | 0.167123288 | 0 | 8.154420275 | Low groups |
| TCGA-66-2766-01 | 0.084931507 | 0 | 7.856144121 | Low groups |
| TCGA-66-2767-01 | 0.167123288 | 0 | 8.165580664 | Low groups |
| TCGA-66-2768-01 | 0.167123288 | 0 | 8.004220017 | Low groups |
| TCGA-66-2770-01 | 1.917808219 | 0 | 8.278844159 | High groups |
| TCGA-66-2771-01 | 1.583561644 | 0 | 8.865228347 | High groups |
| TCGA-66-2777-01 | 0.167123288 | 0 | 8.79636284 | High groups |
| TCGA-66-2780-01 | 1.002739726 | 0 | 8.415621236 | High groups |
| TCGA-66-2781-01 | 0.331506849 | 0 | 7.982281289 | Low groups |
| TCGA-66-2782-01 | 0.832876712 | 1 | 8.038081544 | Low groups |
| TCGA-66-2783-01 | 2.079452055 | 0 | 8.639226302 | High groups |
| TCGA-66-2785-01 | 0.164383562 | 0 | 8.314454636 | High groups |
| TCGA-66-2786-01 | 2.164383562 | 0 | 9.538678996 | High groups |
| TCGA-66-2787-01 | 3.334246575 | 0 | 8.969919321 | High groups |
| TCGA-66-2788-01 | 1.915068493 | 0 | 9.217424614 | High groups |
| TCGA-66-2789-01 | 0.336986301 | 0 | 9.412842271 | High groups |
| TCGA-66-2790-01 | 1.915068493 | 1 | 9.275231357 | High groups |
| TCGA-66-2791-01 | 0.419178082 | 0 | 8.467427444 | High groups |
| TCGA-66-2792-01 | 2.501369863 | 0 | 8.509907394 | High groups |
| TCGA-66-2793-01 | 0.838356164 | 0 | 9.750164856 | High groups |
| TCGA-66-2794-01 | 4.506849315 | 0 | 8.404997016 | High groups |
| TCGA-66-2795-01 | 0.334246575 | 0 | 7.906674476 | Low groups |
| TCGA-66-2800-01 | 4.087671233 | 0 | 8.442672343 | High groups |
| TCGA-68-7755-01 | 0.22739726 | 0 | 6.92963491 | Low groups |
| TCGA-68-7756-01 | 0.553424658 | 0 | 7.182828239 | Low groups |
| TCGA-68-7757-01 | 0.578082192 | 0 | 6.712458767 | Low groups |
| TCGA-68-8250-01 | 0.668493151 | 0 | 7.946702032 | Low groups |
| TCGA-68-8251-01 | 1.112328767 | 0 | 7.67415681 | Low groups |
| TCGA-68-A59I-01 | 0.435616438 | 1 | 7.656994892 | Low groups |
| TCGA-68-A59J-01 | 1.22739726 | 0 | 8.028533902 | Low groups |
| TCGA-70-6722-01 | 0.802739726 | 1 | 8.048068418 | Low groups |
| TCGA-70-6723-01 | 0.854794521 | 1 | 7.982858195 | Low groups |
| TCGA-77-6842-01 | 0.550684932 | 1 | 8.662978063 | High groups |
| TCGA-77-6843-01 | 6.093150685 | 0 | 8.961688841 | High groups |
| TCGA-77-6844-01 | 4.898630137 | 1 | 8.86957324 | High groups |
| TCGA-77-6845-01 | 1.939726027 | 0 | 8.242408438 | High groups |
| TCGA-77-7138-01 | 0.931506849 | 0 | 7.172590621 | Low groups |
| TCGA-77-7139-01 | 11.6739726 | 0 | 7.770197002 | Low groups |
| TCGA-77-7140-01 | 0.684931507 | 1 | 7.509601554 | Low groups |
| TCGA-77-7141-01 | 0.04109589 | 0 | 7.60649353 | Low groups |
| TCGA-77-7142-01 | 6.101369863 | 0 | 8.021919953 | Low groups |
| TCGA-77-7335-01 | 5.843835616 | 0 | 7.613366408 | Low groups |
| TCGA-77-7337-01 | 8.912328767 | 0 | 6.531805473 | Low groups |
| TCGA-77-7338-01 | 0.01369863 | 0 | 7.737736982 | Low groups |
| TCGA-77-7463-01 | 3.898630137 | 0 | 7.41284079 | Low groups |
| TCGA-77-7465-01 | 2.712328767 | 0 | 7.591690561 | Low groups |
| TCGA-77-8007-01 | 0.380821918 | 1 | 7.87650958 | Low groups |
| TCGA-77-8008-01 | 7.230136986 | 0 | 7.377489063 | Low groups |
| TCGA-77-8009-01 | 1.663013699 | 1 | 6.926193624 | Low groups |
| TCGA-77-8128-01 | 3.150684932 | 0 | 7.341165372 | Low groups |
| TCGA-77-8130-01 | 12.24931507 | 1 | 6.722907372 | Low groups |
| TCGA-77-8131-01 | 1.049315068 | 0 | 8.16863632 | Low groups |
| TCGA-77-8133-01 | 4.493150685 | 0 | 7.784752939 | Low groups |
| TCGA-77-8136-01 | 0.824657534 | 1 | 8.248822845 | High groups |
| TCGA-77-8138-01 | 0.791780822 | 1 | 8.033247334 | Low groups |
| TCGA-77-8139-01 | 8.673972603 | 0 | 7.793449392 | Low groups |
| TCGA-77-8140-01 | 0.961643836 | 0 | 8.747437821 | High groups |
| TCGA-77-8143-01 | 2.2 | 1 | 9.06547333 | High groups |
| TCGA-77-8144-01 | 2.282191781 | 0 | 7.963018701 | Low groups |
| TCGA-77-8145-01 | 0.37260274 | 1 | 7.660309863 | Low groups |
| TCGA-77-8146-01 | 8.736986301 | 0 | 7.250818831 | Low groups |
| TCGA-77-8148-01 | 5.542465753 | 0 | 7.891497444 | Low groups |
| TCGA-77-8150-01 | 3.501369863 | 1 | 7.701576364 | Low groups |
| TCGA-77-8153-01 | 5.457534247 | 0 | 8.245853383 | High groups |
| TCGA-77-8154-01 | 5.043835616 | 0 | 8.205262005 | High groups |
| TCGA-77-8156-01 | 3.030136986 | 0 | 8.040194196 | Low groups |
| TCGA-77-A5FZ-01 | 10.51506849 | 0 | 8.533452619 | High groups |
| TCGA-77-A5G1-01 | 6.019178082 | 1 | 9.137705236 | High groups |
| TCGA-77-A5G3-01 | 12.52054795 | 0 | 7.8283805 | Low groups |
| TCGA-77-A5G6-01 | 1.073972603 | 1 | 8.805216443 | High groups |
| TCGA-77-A5G7-01 | 0.493150685 | 0 | 8.92011057 | High groups |
| TCGA-77-A5G8-01 | 5.161643836 | 0 | 7.889030055 | Low groups |
| TCGA-77-A5GA-01 | 3.506849315 | 0 | 8.10986366 | Low groups |
| TCGA-77-A5GB-01 | 0.62739726 | 1 | 7.842693972 | Low groups |
| TCGA-77-A5GF-01 | 1.978082192 | 1 | 7.651976586 | Low groups |
| TCGA-77-A5GH-01 | 3.238356164 | 0 | 8.108144131 | Low groups |
| TCGA-85-6175-01 | 0.805479452 | 1 | 7.306360681 | Low groups |
| TCGA-85-6560-01 | 3.449315068 | 0 | 8.518439538 | High groups |
| TCGA-85-6561-01 | 3.353424658 | 0 | 7.100770603 | Low groups |
| TCGA-85-6798-01 | 0.493150685 | 1 | 8.5322928 | High groups |
| TCGA-85-7696-01 | 3.043835616 | 0 | 6.955554829 | Low groups |
| TCGA-85-7697-01 | 2.912328767 | 0 | 8.207696693 | High groups |
| TCGA-85-7698-01 | 1.780821918 | 1 | 7.257829353 | Low groups |
| TCGA-85-7699-01 | 2.082191781 | 1 | 7.589201023 | Low groups |
| TCGA-85-7710-01 | 0.115068493 | 0 | 6.948092568 | Low groups |
| TCGA-85-7843-01 | 0.095890411 | 0 | 7.93687511 | Low groups |
| TCGA-85-7844-01 | 2.495890411 | 0 | 7.887605994 | Low groups |
| TCGA-85-7950-01 | 1.578082192 | 0 | 7.741811776 | Low groups |
| TCGA-85-8048-01 | 2.095890411 | 0 | 7.94923399 | Low groups |
| TCGA-85-8049-01 | 1.58630137 | 0 | 7.665061068 | Low groups |
| TCGA-85-8052-01 | 2.010958904 | 0 | 8.115388503 | Low groups |
| TCGA-85-8070-01 | 2.630136986 | 0 | 9.255409712 | High groups |
| TCGA-85-8071-01 | 2.232876712 | 0 | 8.423857557 | High groups |
| TCGA-85-8072-01 | 2.553424658 | 0 | 8.18529753 | High groups |
| TCGA-85-8276-01 | 2.871232877 | 1 | 7.944690278 | Low groups |
| TCGA-85-8287-01 | 0.063013699 | 0 | 7.703644349 | Low groups |
| TCGA-85-8288-01 | 1.101369863 | 0 | 7.513148586 | Low groups |
| TCGA-85-8350-01 | 1.871232877 | 0 | 7.820239286 | Low groups |
| TCGA-85-8351-01 | 1.397260274 | 0 | 7.65154399 | Low groups |
| TCGA-85-8352-01 | 0.350684932 | 1 | 7.937661242 | Low groups |
| TCGA-85-8353-01 | 0.257534247 | 1 | 7.163536067 | Low groups |
| TCGA-85-8354-01 | 2.726027397 | 0 | 8.566829003 | High groups |
| TCGA-85-8355-01 | 0.167123288 | 0 | 8.05086064 | Low groups |
| TCGA-85-8479-01 | 1.282191781 | 0 | 7.739075187 | Low groups |
| TCGA-85-8481-01 | 0.646575342 | 0 | 7.240219864 | Low groups |
| TCGA-85-8580-01 | 3.049315068 | 0 | 9.421029819 | High groups |
| TCGA-85-8582-01 | 3.178082192 | 0 | 7.634969647 | Low groups |
| TCGA-85-8584-01 | 1.049315068 | 0 | 7.441636519 | Low groups |
| TCGA-85-8664-01 | 1.189041096 | 1 | 7.724059554 | Low groups |
| TCGA-85-8666-01 | 1.882191781 | 0 | 7.623440459 | Low groups |
| TCGA-85-A4CL-01 | 2.523287671 | 0 | 7.573750709 | Low groups |
| TCGA-85-A4CN-01 | 2.843835616 | 0 | 7.756275829 | Low groups |
| TCGA-85-A4JB-01 | 2.580821918 | 0 | 7.94593269 | Low groups |
| TCGA-85-A4JC-01 | 1.501369863 | 1 | 7.888718656 | Low groups |
| TCGA-85-A4PA-01 | 2.030136986 | 0 | 9.387993814 | High groups |
| TCGA-85-A4QQ-01 | 2.539726027 | 0 | 7.678237651 | Low groups |
| TCGA-85-A4QR-01 | 1.643835616 | 0 | 8.319193311 | High groups |
| TCGA-85-A50M-01 | 2.263013699 | 0 | 7.525637316 | Low groups |
| TCGA-85-A50Z-01 | 1.265753425 | 1 | 8.566878232 | High groups |
| TCGA-85-A510-01 | 0.994520548 | 1 | 9.355161882 | High groups |
| TCGA-85-A511-01 | 1.068493151 | 1 | 7.025302818 | Low groups |
| TCGA-85-A512-01 | 1.273972603 | 0 | 7.957672272 | Low groups |
| TCGA-85-A513-01 | 2.493150685 | 0 | 8.848510337 | High groups |
| TCGA-85-A53L-01 | 1.032876712 | 0 | 7.642895396 | Low groups |
| TCGA-85-A5B5-01 | 0.304109589 | 0 | 7.845283103 | Low groups |
| TCGA-90-6837-01 | 2.076712329 | 0 | 7.939365527 | Low groups |
| TCGA-90-7766-01 | 0.791780822 | 1 | 8.553621922 | High groups |
| TCGA-90-7767-01 | 0.243835616 | 0 | 6.659191326 | Low groups |
| TCGA-90-7769-01 | 0.980821918 | 0 | 7.567244018 | Low groups |
| TCGA-90-7964-01 | 1.17260274 | 0 | 8.211746013 | High groups |
| TCGA-90-A4ED-01 | 1.684931507 | 0 | 8.593848604 | High groups |
| TCGA-90-A4EE-01 | 1.884931507 | 0 | 8.297827684 | High groups |
| TCGA-90-A59Q-01 | 0.750684932 | 1 | 7.798901257 | Low groups |
| TCGA-92-7340-01 | 0.224657534 | 0 | 6.764698404 | Low groups |
| TCGA-92-7341-01 | 0.290410959 | 0 | 7.234094969 | Low groups |
| TCGA-92-8063-01 | 0.334246575 | 0 | 8.62511528 | High groups |
| TCGA-92-8064-01 | 0.438356164 | 0 | 7.77744056 | Low groups |
| TCGA-92-8065-01 | 0.191780822 | 0 | 7.628243056 | Low groups |
| TCGA-94-7033-01 | 1.753424658 | 0 | 8.043122785 | Low groups |
| TCGA-94-7557-01 | 0.01369863 | 0 | 7.555287469 | Low groups |
| TCGA-94-7943-01 | 1.523287671 | 1 | 7.931598795 | Low groups |
| TCGA-94-8035-01 | 0.334246575 | 0 | 8.659693412 | High groups |
| TCGA-94-8490-01 | 0.419178082 | 0 | 6.54305201 | Low groups |
| TCGA-94-8491-01 | 1.756164384 | 1 | 7.692818075 | Low groups |
| TCGA-94-A4VJ-01 | 1.178082192 | 0 | 7.422615577 | Low groups |
| TCGA-94-A5I4-01 | 0.860273973 | 1 | 8.086366932 | Low groups |
| TCGA-94-A5I6-01 | 1.317808219 | 1 | 8.317405152 | High groups |
| TCGA-96-7544-01 | 5.917808219 | 0 | 7.369738141 | Low groups |
| TCGA-96-7545-01 | 4.638356164 | 1 | 7.476084336 | Low groups |
| TCGA-96-8169-01 | 1.526027397 | 0 | 7.710188663 | Low groups |
| TCGA-96-8170-01 | 1.454794521 | 0 | 7.777358166 | Low groups |
| TCGA-96-A4JK-01 | 1.61369863 | 0 | 7.433417941 | Low groups |
| TCGA-96-A4JL-01 | 2.306849315 | 0 | 7.90617869 | Low groups |
| TCGA-98-7454-01 | 1.78630137 | 0 | 7.652428628 | Low groups |
| TCGA-98-8020-01 | 0.030136986 | 1 | 7.975052218 | Low groups |
| TCGA-98-8021-01 | 2.515068493 | 1 | 8.293845437 | High groups |
| TCGA-98-8022-01 | 2.556164384 | 0 | 7.646369482 | Low groups |
| TCGA-98-8023-01 | 1.778082192 | 0 | 7.7619871 | Low groups |
| TCGA-98-A538-01 | 2.263013699 | 0 | 8.205305705 | High groups |
| TCGA-98-A539-01 | 0.473972603 | 1 | 7.841115887 | Low groups |
| TCGA-98-A53A-01 | 1.512328767 | 0 | 9.550535078 | High groups |
| TCGA-98-A53B-01 | 0.167123288 | 0 | 7.716847166 | Low groups |
| TCGA-98-A53C-01 | 2.252054795 | 0 | 8.334959715 | High groups |
| TCGA-98-A53D-01 | 1.131506849 | 1 | 8.161409055 | Low groups |
| TCGA-98-A53H-01 | 1.169863014 | 1 | 8.152709424 | Low groups |
| TCGA-98-A53I-01 | 1.547945205 | 0 | 7.421744057 | Low groups |
| TCGA-98-A53J-01 | 1.726027397 | 0 | 8.121581175 | Low groups |
| TCGA-J1-A4AH-01 | 1.591780822 | 0 | 8.323822192 | High groups |
| TCGA-L3-A4E7-01 | 1.073972603 | 0 | 8.093547342 | Low groups |
| TCGA-L3-A524-01 | 1.342465753 | 0 | 8.118528964 | Low groups |
| TCGA-LA-A446-01 | 1.098630137 | 0 | 8.375064635 | High groups |
| TCGA-LA-A7SW-01 | 0.643835616 | 1 | 8.010875318 | Low groups |
| TCGA-MF-A522-01 | 0.473972603 | 1 | 7.584106473 | Low groups |
| TCGA-NC-A5HD-01 | 0.005479452 | 0 | 9.16833423 | High groups |
| TCGA-NC-A5HE-01 | 6.4 | 0 | 7.799825954 | Low groups |
| TCGA-NC-A5HF-01 | 0.361643836 | 1 | 8.65031695 | High groups |
| TCGA-NC-A5HG-01 | 5.378082192 | 0 | 8.583554059 | High groups |
| TCGA-NC-A5HH-01 | 0.101369863 | 0 | 8.792946754 | High groups |
| TCGA-NC-A5HI-01 | 0.191780822 | 1 | 8.679476862 | High groups |
| TCGA-NC-A5HJ-01 | 0.898630137 | 1 | 9.022093675 | High groups |
| TCGA-NC-A5HK-01 | 0.350684932 | 0 | 8.429672449 | High groups |
| TCGA-NC-A5HL-01 | 0.24109589 | 0 | 7.775282761 | Low groups |
| TCGA-NC-A5HM-01 | 3.320547945 | 0 | 8.512999157 | High groups |
| TCGA-NC-A5HN-01 | 4.106849315 | 0 | 7.958089007 | Low groups |
| TCGA-NC-A5HO-01 | 3.660273973 | 0 | 7.684046911 | Low groups |
| TCGA-NC-A5HP-01 | 0.868493151 | 1 | 7.247710808 | Low groups |
| TCGA-NC-A5HQ-01 | 1.22739726 | 0 | 7.994673001 | Low groups |
| TCGA-NC-A5HR-01 | 3.408219178 | 0 | 8.304375692 | High groups |
| TCGA-NC-A5HT-01 | 0.556164384 | 1 | 8.194616369 | High groups |
| TCGA-NK-A5CR-01 | 6.964383562 | 0 | 8.346423667 | High groups |
| TCGA-NK-A5CT-01 | 5.452054795 | 1 | 8.785492781 | High groups |
| TCGA-NK-A5CX-01 | 0.304109589 | 0 | 7.431847737 | Low groups |
| TCGA-NK-A5D1-01 | 0.41369863 | 1 | 10.40786469 | High groups |
| TCGA-NK-A7XE-01 | 0.035616438 | 0 | 7.877460038 | Low groups |
| TCGA-O2-A52N-01 | 2.756164384 | 0 | 7.779176708 | Low groups |
| TCGA-O2-A52Q-01 | 0.238356164 | 1 | 8.640825441 | High groups |
| TCGA-O2-A52S-01 | 0.673972603 | 1 | 8.338745651 | High groups |
| TCGA-O2-A52V-01 | 1.890410959 | 1 | 7.807154799 | Low groups |
| TCGA-O2-A52W-01 | 0.715068493 | 0 | 8.631422639 | High groups |
| TCGA-O2-A5IB-01 | 0.693150685 | 1 | 8.352416028 | High groups |
| TCGA-XC-AA0X-01 | 0.016438356 | 0 | 8.176669441 | Low groups |

**Table S12.** Raw counts of RNA-sequencing data of hsa-miR-199a-5p in LUSC from the TCGA.

| sampleID | time | Staus | RS | Label |
| --- | --- | --- | --- | --- |
| TCGA-18-3406-01 | 0.978082192 | 1 | 9.134031117 | Low groups |
| TCGA-18-3407-01 | 0.37260274 | 0 | 10.5616973 | High groups |
| TCGA-18-3408-01 | 4.912328767 | 1 | 9.164392341 | Low groups |
| TCGA-18-3410-01 | 0.4 | 0 | 9.47839538 | Low groups |
| TCGA-18-3411-01 | 9.797260274 | 0 | 9.646138422 | Low groups |
| TCGA-18-3412-01 | 0.747945205 | 1 | 8.931293318 | Low groups |
| TCGA-18-3414-01 | 1.961643836 | 0 | 8.800056129 | Low groups |
| TCGA-18-3415-01 | 7.679452055 | 0 | 8.842764768 | Low groups |
| TCGA-18-3416-01 | 2.575342466 | 1 | 6.832152849 | Low groups |
| TCGA-18-3417-01 | 3.005479452 | 0 | 11.13904862 | High groups |
| TCGA-18-3419-01 | 7.701369863 | 0 | 6.627671034 | Low groups |
| TCGA-18-3421-01 | 7.246575342 | 0 | 8.584177709 | Low groups |
| TCGA-18-4086-01 | 0.232876712 | 0 | 9.580611443 | Low groups |
| TCGA-18-4721-01 | 12.86027397 | 0 | 10.38485097 | High groups |
| TCGA-18-5592-01 | 4.161643836 | 0 | 9.724234564 | Low groups |
| TCGA-18-5595-01 | 2.265753425 | 0 | 9.313422119 | Low groups |
| TCGA-21-1072-01 | 8.263013699 | 0 | 9.47151149 | Low groups |
| TCGA-21-1075-01 | 5.846575342 | 0 | 8.517630338 | Low groups |
| TCGA-21-1076-01 | 4.315068493 | 1 | 10.3179559 | High groups |
| TCGA-21-1077-01 | 1.764383562 | 1 | 10.45031502 | High groups |
| TCGA-21-1078-01 | 0.704109589 | 1 | 9.13522138 | Low groups |
| TCGA-21-1079-01 | 0.868493151 | 1 | 9.165630857 | Low groups |
| TCGA-21-1080-01 | 10.20273973 | 0 | 9.452920311 | Low groups |
| TCGA-21-1083-01 | 3.602739726 | 0 | 9.339856905 | Low groups |
| TCGA-21-5782-01 | 2.635616438 | 0 | 11.30038133 | High groups |
| TCGA-21-5783-01 | 7.342465753 | 0 | 11.30585786 | High groups |
| TCGA-21-5784-01 | 3.473972603 | 0 | 11.18522724 | High groups |
| TCGA-21-5786-01 | 2.238356164 | 1 | 11.23650069 | High groups |
| TCGA-21-5787-01 | 0.282191781 | 1 | 8.91754564 | Low groups |
| TCGA-21-A5DI-01 | 2.682191781 | 0 | 10.40783453 | High groups |
| TCGA-22-0940-01 | 1.832876712 | 1 | 10.62331308 | High groups |
| TCGA-22-0944-01 | 0.610958904 | 0 | 9.399127505 | Low groups |
| TCGA-22-1011-01 | 0.145205479 | 0 | 9.880372381 | Low groups |
| TCGA-22-1012-01 | 1.175342466 | 0 | 9.818846312 | Low groups |
| TCGA-22-4591-01 | 1.21369863 | 1 | 8.465366005 | Low groups |
| TCGA-22-4593-01 | 2.923287671 | 0 | 10.09828414 | High groups |
| TCGA-22-4594-01 | 4.02739726 | 0 | 9.649891614 | Low groups |
| TCGA-22-4595-01 | 0.684931507 | 1 | 10.29860219 | High groups |
| TCGA-22-4596-01 | 0.046575342 | 0 | 9.729205293 | Low groups |
| TCGA-22-4601-01 | 1.438356164 | 1 | 8.831066922 | Low groups |
| TCGA-22-4604-01 | 0.838356164 | 1 | 10.65235124 | High groups |
| TCGA-22-4605-01 | 2.668493151 | 0 | 10.28213187 | High groups |
| TCGA-22-4607-01 | 1.608219178 | 0 | 9.593467317 | Low groups |
| TCGA-22-4609-01 | 0.797260274 | 0 | 10.36669137 | High groups |
| TCGA-22-4613-01 | 0.980821918 | 0 | 9.088943628 | Low groups |
| TCGA-22-5471-01 | 0.62739726 | 1 | 10.90794916 | High groups |
| TCGA-22-5472-01 | 2.043835616 | 1 | 10.36911888 | High groups |
| TCGA-22-5473-01 | 4.534246575 | 1 | 11.00125322 | High groups |
| TCGA-22-5474-01 | 1.219178082 | 0 | 9.38571683 | Low groups |
| TCGA-22-5477-01 | 1.4 | 1 | 8.924850084 | Low groups |
| TCGA-22-5478-01 | 0.065753425 | 0 | 10.72697244 | High groups |
| TCGA-22-5479-01 | 7.191780822 | 0 | 8.761616551 | Low groups |
| TCGA-22-5480-01 | 4.873972603 | 1 | 10.66248832 | High groups |
| TCGA-22-5481-01 | 0.947945205 | 1 | 9.842455949 | Low groups |
| TCGA-22-5482-01 | 0.978082192 | 0 | 11.45793702 | High groups |
| TCGA-22-5483-01 | 1.556164384 | 1 | 8.49549171 | Low groups |
| TCGA-22-5485-01 | 2.301369863 | 1 | 10.22059417 | High groups |
| TCGA-22-5489-01 | 1.17260274 | 1 | 11.08503607 | High groups |
| TCGA-22-5491-01 | 4.693150685 | 0 | 8.429971991 | Low groups |
| TCGA-22-5492-01 | 1.38630137 | 0 | 9.930755371 | Low groups |
| TCGA-22-A5C4-01 | 1.838356164 | 0 | 10.60668188 | High groups |
| TCGA-33-4532-01 | 10.75068493 | 0 | 8.210494314 | Low groups |
| TCGA-33-4533-01 | 11.14520548 | 0 | 12.06206722 | High groups |
| TCGA-33-4538-01 | 8.161643836 | 0 | 8.999130176 | Low groups |
| TCGA-33-4547-01 | 6.62739726 | 0 | 10.28539783 | High groups |
| TCGA-33-4566-01 | 14.48493151 | 0 | 9.355875657 | Low groups |
| TCGA-33-4582-01 | 8.008219178 | 1 | 10.604594 | High groups |
| TCGA-33-4583-01 | 12.60547945 | 0 | 9.957489369 | Low groups |
| TCGA-33-4586-01 | 0.463013699 | 1 | 10.15280161 | High groups |
| TCGA-33-4587-01 | 4.536986301 | 1 | 8.449002924 | Low groups |
| TCGA-33-4589-01 | 0.128767123 | 0 | 8.749024743 | Low groups |
| TCGA-33-6737-01 | 1.646575342 | 1 | 7.894930326 | Low groups |
| TCGA-33-6738-01 | 5.279452055 | 0 | 9.789608794 | Low groups |
| TCGA-33-A4WN-01 | 0.391780822 | 0 | 10.73095322 | High groups |
| TCGA-33-A5GW-01 | 0.024657534 | 0 | 9.253829998 | Low groups |
| TCGA-33-AAS8-01 | 3.052054795 | 0 | 9.705128998 | Low groups |
| TCGA-33-AASB-01 | 0.082191781 | 1 | 10.04096652 | High groups |
| TCGA-33-AASD-01 | 5.238356164 | 1 | 9.930756554 | Low groups |
| TCGA-33-AASI-01 | 3.682191781 | 1 | 9.076938381 | Low groups |
| TCGA-33-AASJ-01 | 3.109589041 | 1 | 10.2867041 | High groups |
| TCGA-33-AASL-01 | 0.408219178 | 1 | 8.578455344 | Low groups |
| TCGA-34-2596-01 | 0.219178082 | 0 | 9.970266261 | High groups |
| TCGA-34-2600-01 | 5.134246575 | 0 | 8.06191911 | Low groups |
| TCGA-34-2604-01 | 2.624657534 | 0 | 9.784801269 | Low groups |
| TCGA-34-2605-01 | 1.079452055 | 1 | 10.45617984 | High groups |
| TCGA-34-2608-01 | 2.739726027 | 0 | 10.34611888 | High groups |
| TCGA-34-2609-01 | 1.060273973 | 1 | 9.205993332 | Low groups |
| TCGA-34-5231-01 | 5.435616438 | 0 | 8.648924302 | Low groups |
| TCGA-34-5232-01 | 6.769863014 | 0 | 8.147891759 | Low groups |
| TCGA-34-5234-01 | 6.221917808 | 0 | 11.06053484 | High groups |
| TCGA-34-5236-01 | 0.309589041 | 1 | 10.12990324 | High groups |
| TCGA-34-5239-01 | 4.191780822 | 1 | 9.781462666 | Low groups |
| TCGA-34-5240-01 | 4.221917808 | 0 | 10.6407872 | High groups |
| TCGA-34-5241-01 | 1.410958904 | 0 | 10.57211536 | High groups |
| TCGA-34-5927-01 | 0.671232877 | 1 | 7.990592043 | Low groups |
| TCGA-34-5928-01 | 3.276712329 | 0 | 10.12534217 | High groups |
| TCGA-34-5929-01 | 0.41369863 | 0 | 8.798130893 | Low groups |
| TCGA-34-7107-01 | 0.093150685 | 0 | 10.6547998 | High groups |
| TCGA-34-8454-01 | 3.232876712 | 0 | 10.06628001 | High groups |
| TCGA-34-8455-01 | 0.336986301 | 1 | 9.933426874 | Low groups |
| TCGA-34-8456-01 | 2.202739726 | 0 | 9.494389008 | Low groups |
| TCGA-34-A5IX-01 | 2.824657534 | 0 | 10.49102241 | High groups |
| TCGA-37-3783-01 | 0.334246575 | 0 | 10.2439864 | High groups |
| TCGA-37-3789-01 | 0.035616438 | 0 | 7.725868804 | Low groups |
| TCGA-37-3792-01 | 0.032876712 | 0 | 6.257922691 | Low groups |
| TCGA-37-4130-01 | 0.676712329 | 0 | 7.163677146 | Low groups |
| TCGA-37-4133-01 | 0.652054795 | 0 | 9.377701961 | Low groups |
| TCGA-37-4135-01 | 0.567123288 | 0 | 8.161837812 | Low groups |
| TCGA-37-4141-01 | 0.032876712 | 0 | 9.61744965 | Low groups |
| TCGA-37-5819-01 | 0.282191781 | 0 | 8.90451872 | Low groups |
| TCGA-37-A5EL-01 | 2.591780822 | 1 | 9.080219624 | Low groups |
| TCGA-37-A5EM-01 | 2.375342466 | 0 | 10.15826633 | High groups |
| TCGA-37-A5EN-01 | 1.808219178 | 0 | 9.958116709 | High groups |
| TCGA-39-5011-01 | 1.547945205 | 1 | 10.01459994 | High groups |
| TCGA-39-5019-01 | 1.035616438 | 1 | 9.502815776 | Low groups |
| TCGA-39-5021-01 | 5.136986301 | 1 | 10.35000533 | High groups |
| TCGA-39-5022-01 | 3.967123288 | 1 | 9.126141221 | Low groups |
| TCGA-39-5024-01 | 6.876712329 | 0 | 9.174946399 | Low groups |
| TCGA-39-5027-01 | 8.44109589 | 1 | 8.303956965 | Low groups |
| TCGA-39-5028-01 | 0.142465753 | 0 | 10.50382206 | High groups |
| TCGA-39-5029-01 | 1.345205479 | 1 | 9.383129584 | Low groups |
| TCGA-39-5030-01 | 0.161643836 | 0 | 10.42837734 | High groups |
| TCGA-39-5031-01 | 5.043835616 | 0 | 10.64648788 | High groups |
| TCGA-39-5034-01 | 0.964383562 | 1 | 10.57490379 | High groups |
| TCGA-39-5035-01 | 5.698630137 | 0 | 9.47238054 | Low groups |
| TCGA-39-5036-01 | 5.931506849 | 0 | 9.819576543 | Low groups |
| TCGA-39-5037-01 | 4.630136986 | 0 | 9.586252948 | Low groups |
| TCGA-39-5039-01 | 1.490410959 | 0 | 11.06973438 | High groups |
| TCGA-39-5040-01 | 1.210958904 | 1 | 9.223788873 | Low groups |
| TCGA-43-2576-01 | 3.350684932 | 0 | 9.869608031 | Low groups |
| TCGA-43-2578-01 | 1.873972603 | 0 | 8.915237879 | Low groups |
| TCGA-43-2581-01 | 3.221917808 | 0 | 9.539285817 | Low groups |
| TCGA-43-3394-01 | 1.512328767 | 1 | 9.842688347 | Low groups |
| TCGA-43-3920-01 | 2.75890411 | 0 | 11.68574484 | High groups |
| TCGA-43-5668-01 | 1.287671233 | 1 | 8.984594391 | Low groups |
| TCGA-43-5670-01 | 2.326027397 | 0 | 9.172795411 | Low groups |
| TCGA-43-6143-01 | 1.915068493 | 0 | 8.119805704 | Low groups |
| TCGA-43-6647-01 | 2.073972603 | 0 | 9.378696283 | Low groups |
| TCGA-43-6770-01 | 1.789041096 | 0 | 9.268937035 | Low groups |
| TCGA-43-6771-01 | 0.421917808 | 1 | 9.28822058 | Low groups |
| TCGA-43-6773-01 | 0.317808219 | 0 | 10.75545495 | High groups |
| TCGA-43-7656-01 | 1.632876712 | 0 | 10.46677535 | High groups |
| TCGA-43-7657-01 | 0.646575342 | 0 | 11.15132382 | High groups |
| TCGA-43-7658-01 | 6.315068493 | 1 | 8.685037902 | Low groups |
| TCGA-43-8115-01 | 1.115068493 | 0 | 10.9602391 | High groups |
| TCGA-43-8116-01 | 0.980821918 | 0 | 9.742087667 | Low groups |
| TCGA-43-8118-01 | 0.243835616 | 0 | 10.34932414 | High groups |
| TCGA-43-A474-01 | 0.967123288 | 0 | 10.0739735 | High groups |
| TCGA-43-A475-01 | 0.810958904 | 0 | 9.749743081 | Low groups |
| TCGA-43-A56U-01 | 1.183561644 | 0 | 10.47094474 | High groups |
| TCGA-43-A56V-01 | 0.97260274 | 1 | 10.87922238 | High groups |
| TCGA-46-3765-01 | 1.109589041 | 0 | 9.168695833 | Low groups |
| TCGA-46-3766-01 | 1.01369863 | 0 | 9.398967239 | Low groups |
| TCGA-46-3767-01 | 1.084931507 | 0 | 9.940257856 | Low groups |
| TCGA-46-3768-01 | 0.819178082 | 0 | 9.466325569 | Low groups |
| TCGA-46-3769-01 | 0.369863014 | 0 | 9.916342431 | Low groups |
| TCGA-46-6025-01 | 0.887671233 | 0 | 9.035037421 | Low groups |
| TCGA-46-6026-01 | 1.15890411 | 0 | 8.03960216 | Low groups |
| TCGA-51-4079-01 | 0.032876712 | 0 | 10.12911916 | High groups |
| TCGA-51-4080-01 | 0.032876712 | 0 | 7.504370123 | Low groups |
| TCGA-51-4081-01 | 2.495890411 | 0 | 10.35140229 | High groups |
| TCGA-51-6867-01 | 2.15890411 | 1 | 10.51662537 | High groups |
| TCGA-52-7622-01 | 2.361643836 | 0 | 9.717659066 | Low groups |
| TCGA-52-7809-01 | 0.454794521 | 1 | 9.922443174 | Low groups |
| TCGA-52-7810-01 | 2.528767123 | 0 | 10.13684773 | High groups |
| TCGA-52-7811-01 | 0.419178082 | 1 | 10.6121596 | High groups |
| TCGA-52-7812-01 | 2.167123288 | 1 | 9.23641034 | Low groups |
| TCGA-56-1622-01 | 2.41369863 | 1 | 8.7124869 | Low groups |
| TCGA-56-5897-01 | 1.035616438 | 0 | 11.31746801 | High groups |
| TCGA-56-5898-01 | 1.520547945 | 0 | 10.4262918 | High groups |
| TCGA-56-6545-01 | 1.824657534 | 0 | 9.049260736 | Low groups |
| TCGA-56-7221-01 | 1.665753425 | 0 | 10.39475324 | High groups |
| TCGA-56-7222-01 | 1.169863014 | 1 | 9.041709522 | Low groups |
| TCGA-56-7223-01 | 0.361643836 | 1 | 9.180337457 | Low groups |
| TCGA-56-7579-01 | 0.408219178 | 1 | 9.333657634 | Low groups |
| TCGA-56-7580-01 | 2.534246575 | 0 | 11.33260193 | High groups |
| TCGA-56-7582-01 | 1.646575342 | 0 | 9.687667343 | Low groups |
| TCGA-56-7730-01 | 0.542465753 | 1 | 9.094803365 | Low groups |
| TCGA-56-7731-01 | 0.008219178 | 0 | 10.0912349 | High groups |
| TCGA-56-7822-01 | 0.824657534 | 1 | 10.19032818 | High groups |
| TCGA-56-7823-01 | 2.769863014 | 0 | 8.3509246 | Low groups |
| TCGA-56-8082-01 | 1.246575342 | 0 | 8.710198101 | Low groups |
| TCGA-56-8083-01 | 0.410958904 | 0 | 11.49352275 | High groups |
| TCGA-56-8201-01 | 1.087671233 | 1 | 9.874738819 | Low groups |
| TCGA-56-8304-01 | 0.290410959 | 0 | 10.06980472 | High groups |
| TCGA-56-8305-01 | 0.287671233 | 0 | 11.13090481 | High groups |
| TCGA-56-8307-01 | 2.24109589 | 0 | 9.978305904 | High groups |
| TCGA-56-8308-01 | 1.416438356 | 0 | 9.722458852 | Low groups |
| TCGA-56-8309-01 | 1.17260274 | 0 | 9.610301239 | Low groups |
| TCGA-56-8503-01 | 0.112328767 | 0 | 11.39035514 | High groups |
| TCGA-56-8504-01 | 1.397260274 | 0 | 11.02379246 | High groups |
| TCGA-56-8622-01 | 0.150684932 | 0 | 10.78283936 | High groups |
| TCGA-56-8623-01 | 1.767123288 | 1 | 10.14669353 | High groups |
| TCGA-56-8624-01 | 1.150684932 | 0 | 11.18845533 | High groups |
| TCGA-56-8625-01 | 0.745205479 | 1 | 11.79907613 | High groups |
| TCGA-56-8626-01 | 0.82739726 | 0 | 10.73404654 | High groups |
| TCGA-56-8628-01 | 1.687671233 | 0 | 10.91852197 | High groups |
| TCGA-56-8629-01 | 1.317808219 | 0 | 9.553064872 | Low groups |
| TCGA-56-A49D-01 | 1.745205479 | 0 | 9.336812026 | Low groups |
| TCGA-56-A4BW-01 | 1.602739726 | 0 | 10.71694615 | High groups |
| TCGA-56-A4BX-01 | 1.109589041 | 0 | 8.985967136 | Low groups |
| TCGA-56-A4BY-01 | 1.487671233 | 1 | 10.23094846 | High groups |
| TCGA-56-A4ZJ-01 | 1.753424658 | 0 | 11.21848532 | High groups |
| TCGA-56-A4ZK-01 | 1.561643836 | 0 | 8.821906704 | Low groups |
| TCGA-56-A5DR-01 | 0.010958904 | 0 | 11.39476658 | High groups |
| TCGA-56-A62T-01 | 1.205479452 | 0 | 9.851748573 | Low groups |
| TCGA-58-8386-01 | 0.002739726 | 0 | 9.139707789 | Low groups |
| TCGA-58-8387-01 | 1.104109589 | 0 | 9.396921326 | Low groups |
| TCGA-58-8388-01 | 1.128767123 | 0 | 10.00130312 | High groups |
| TCGA-58-8390-01 | 2.495890411 | 0 | 12.43335097 | High groups |
| TCGA-58-8391-01 | 5.569863014 | 1 | 9.603442937 | Low groups |
| TCGA-58-8392-01 | 0.263013699 | 1 | 9.644768947 | Low groups |
| TCGA-58-8393-01 | 2.898630137 | 0 | 9.025382585 | Low groups |
| TCGA-58-A46J-01 | 7.093150685 | 0 | 10.10035708 | High groups |
| TCGA-58-A46K-01 | 2.01369863 | 1 | 10.31468576 | High groups |
| TCGA-58-A46L-01 | 4.720547945 | 0 | 11.33667994 | High groups |
| TCGA-58-A46M-01 | 2.936986301 | 0 | 9.236063118 | Low groups |
| TCGA-58-A46N-01 | 1.726027397 | 1 | 7.983372731 | Low groups |
| TCGA-60-2695-01 | 1.75890411 | 0 | 8.511415972 | Low groups |
| TCGA-60-2696-01 | 0.298630137 | 0 | 9.889701836 | Low groups |
| TCGA-60-2697-01 | 0.547945205 | 1 | 10.07608525 | High groups |
| TCGA-60-2698-01 | 0.312328767 | 1 | 9.621699708 | Low groups |
| TCGA-60-2703-01 | 4.81369863 | 1 | 10.40287787 | High groups |
| TCGA-60-2704-01 | 1.457534247 | 1 | 11.05223994 | High groups |
| TCGA-60-2706-01 | 7.726027397 | 0 | 9.378820309 | Low groups |
| TCGA-60-2708-01 | 6.704109589 | 0 | 10.90364118 | High groups |
| TCGA-60-2709-01 | 4.123287671 | 0 | 9.806556308 | Low groups |
| TCGA-60-2710-01 | 5.545205479 | 0 | 10.36729101 | High groups |
| TCGA-60-2711-01 | 3.452054795 | 0 | 9.369229374 | Low groups |
| TCGA-60-2712-01 | 0.750684932 | 0 | 11.80524594 | High groups |
| TCGA-60-2713-01 | 3.715068493 | 1 | 10.59610122 | High groups |
| TCGA-60-2714-01 | 4.194520548 | 0 | 9.316014105 | Low groups |
| TCGA-60-2716-01 | 4.04109589 | 0 | 8.999117291 | Low groups |
| TCGA-60-2719-01 | 3.553424658 | 0 | 9.163515337 | Low groups |
| TCGA-60-2720-01 | 0.265753425 | 0 | 10.38734132 | High groups |
| TCGA-60-2721-01 | 2.693150685 | 0 | 10.33765011 | High groups |
| TCGA-60-2722-01 | 2.331506849 | 1 | 8.406510608 | Low groups |
| TCGA-60-2723-01 | 2.991780822 | 0 | 10.08889475 | High groups |
| TCGA-60-2724-01 | 1.964383562 | 0 | 10.9238209 | High groups |
| TCGA-60-2725-01 | 2.235616438 | 0 | 10.61101465 | High groups |
| TCGA-63-6202-01 | 4.389041096 | 0 | 8.610568302 | Low groups |
| TCGA-63-7020-01 | 5.843835616 | 0 | 10.03637956 | High groups |
| TCGA-63-7021-01 | 4.117808219 | 1 | 10.69479951 | High groups |
| TCGA-63-7022-01 | 5.679452055 | 0 | 11.66925226 | High groups |
| TCGA-63-A5M9-01 | 0 | 0 | 10.41508332 | High groups |
| TCGA-63-A5MB-01 | 8.556164384 | 0 | 9.626131363 | Low groups |
| TCGA-63-A5MG-01 | 5.884931507 | 0 | 11.29156615 | High groups |
| TCGA-63-A5MH-01 | 5.550684932 | 0 | 12.32015999 | High groups |
| TCGA-63-A5MI-01 | 4.887671233 | 0 | 10.65806346 | High groups |
| TCGA-63-A5MJ-01 | 4.997260274 | 0 | 10.66184711 | High groups |
| TCGA-63-A5ML-01 | 3.797260274 | 0 | 9.916884286 | Low groups |
| TCGA-63-A5MM-01 | 0.597260274 | 1 | 10.82975967 | High groups |
| TCGA-63-A5MN-01 | 0.876712329 | 1 | 11.13580899 | High groups |
| TCGA-63-A5MP-01 | 1.4 | 1 | 9.513799749 | Low groups |
| TCGA-63-A5MR-01 | 7.44109589 | 0 | 10.749994 | High groups |
| TCGA-63-A5MS-01 | 6.523287671 | 0 | 11.00212866 | High groups |
| TCGA-63-A5MT-01 | 1.169863014 | 1 | 10.40967786 | High groups |
| TCGA-63-A5MU-01 | 1.150684932 | 1 | 9.358874675 | Low groups |
| TCGA-63-A5MV-01 | 3.01369863 | 0 | 10.77295725 | High groups |
| TCGA-63-A5MW-01 | 4.490410959 | 0 | 12.02594317 | High groups |
| TCGA-63-A5MY-01 | 2.882191781 | 0 | 8.421313907 | Low groups |
| TCGA-66-2727-01 | 1.41369863 | 0 | 10.07069147 | High groups |
| TCGA-66-2734-01 | 3.591780822 | 0 | 9.479475124 | Low groups |
| TCGA-66-2737-01 | 0.167123288 | 0 | 10.30030245 | High groups |
| TCGA-66-2742-01 | 1.756164384 | 0 | 8.944107569 | Low groups |
| TCGA-66-2744-01 | 0.082191781 | 0 | 8.118458883 | Low groups |
| TCGA-66-2753-01 | 0.084931507 | 0 | 9.555215751 | Low groups |
| TCGA-66-2754-01 | 0.167123288 | 0 | 7.089127133 | Low groups |
| TCGA-66-2755-01 | 0.076712329 | 0 | 10.1563726 | High groups |
| TCGA-66-2758-01 | 1.750684932 | 0 | 9.050055139 | Low groups |
| TCGA-66-2759-01 | 2.087671233 | 0 | 9.606678438 | Low groups |
| TCGA-66-2763-01 | 0.082191781 | 0 | 9.742116137 | Low groups |
| TCGA-66-2765-01 | 0.167123288 | 0 | 9.939715544 | Low groups |
| TCGA-66-2766-01 | 0.084931507 | 0 | 8.884599356 | Low groups |
| TCGA-66-2767-01 | 0.167123288 | 0 | 9.922768277 | Low groups |
| TCGA-66-2768-01 | 0.167123288 | 0 | 7.892200961 | Low groups |
| TCGA-66-2770-01 | 1.917808219 | 0 | 10.87789268 | High groups |
| TCGA-66-2771-01 | 1.583561644 | 0 | 8.291810041 | Low groups |
| TCGA-66-2777-01 | 0.167123288 | 0 | 10.0163984 | High groups |
| TCGA-66-2780-01 | 1.002739726 | 0 | 10.85506444 | High groups |
| TCGA-66-2781-01 | 0.331506849 | 0 | 10.28743997 | High groups |
| TCGA-66-2782-01 | 0.832876712 | 1 | 10.30179911 | High groups |
| TCGA-66-2783-01 | 2.079452055 | 0 | 10.06372241 | High groups |
| TCGA-66-2785-01 | 0.164383562 | 0 | 8.78685051 | Low groups |
| TCGA-66-2786-01 | 2.164383562 | 0 | 10.04381351 | High groups |
| TCGA-66-2787-01 | 3.334246575 | 0 | 9.473704999 | Low groups |
| TCGA-66-2788-01 | 1.915068493 | 0 | 10.66511434 | High groups |
| TCGA-66-2789-01 | 0.336986301 | 0 | 10.11049414 | High groups |
| TCGA-66-2790-01 | 1.915068493 | 1 | 10.68974277 | High groups |
| TCGA-66-2791-01 | 0.419178082 | 0 | 9.686125097 | Low groups |
| TCGA-66-2792-01 | 2.501369863 | 0 | 9.343914542 | Low groups |
| TCGA-66-2793-01 | 0.838356164 | 0 | 8.759971148 | Low groups |
| TCGA-66-2794-01 | 4.506849315 | 0 | 8.605763325 | Low groups |
| TCGA-66-2795-01 | 0.334246575 | 0 | 9.929097646 | Low groups |
| TCGA-66-2800-01 | 4.087671233 | 0 | 10.28407475 | High groups |
| TCGA-68-7755-01 | 0.22739726 | 0 | 10.17734055 | High groups |
| TCGA-68-7756-01 | 0.553424658 | 0 | 10.9799088 | High groups |
| TCGA-68-7757-01 | 0.578082192 | 0 | 10.08001043 | High groups |
| TCGA-68-8250-01 | 0.668493151 | 0 | 9.060147117 | Low groups |
| TCGA-68-8251-01 | 1.112328767 | 0 | 10.84245626 | High groups |
| TCGA-68-A59I-01 | 0.435616438 | 1 | 10.34652005 | High groups |
| TCGA-68-A59J-01 | 1.22739726 | 0 | 10.13479388 | High groups |
| TCGA-70-6722-01 | 0.802739726 | 1 | 9.673682555 | Low groups |
| TCGA-70-6723-01 | 0.854794521 | 1 | 6.942250808 | Low groups |
| TCGA-77-6842-01 | 0.550684932 | 1 | 9.298503903 | Low groups |
| TCGA-77-6843-01 | 6.093150685 | 0 | 7.949991772 | Low groups |
| TCGA-77-6844-01 | 4.898630137 | 1 | 9.891524602 | Low groups |
| TCGA-77-6845-01 | 1.939726027 | 0 | 9.573098026 | Low groups |
| TCGA-77-7138-01 | 0.931506849 | 0 | 9.999388035 | High groups |
| TCGA-77-7139-01 | 11.6739726 | 0 | 9.535130594 | Low groups |
| TCGA-77-7140-01 | 0.684931507 | 1 | 10.16640351 | High groups |
| TCGA-77-7141-01 | 0.04109589 | 0 | 8.833877416 | Low groups |
| TCGA-77-7142-01 | 6.101369863 | 0 | 10.53629521 | High groups |
| TCGA-77-7335-01 | 5.843835616 | 0 | 11.09967064 | High groups |
| TCGA-77-7337-01 | 8.912328767 | 0 | 10.14410884 | High groups |
| TCGA-77-7338-01 | 0.01369863 | 0 | 10.48726194 | High groups |
| TCGA-77-7463-01 | 3.898630137 | 0 | 9.709879747 | Low groups |
| TCGA-77-7465-01 | 2.712328767 | 0 | 9.974571471 | High groups |
| TCGA-77-8007-01 | 0.380821918 | 1 | 9.682719006 | Low groups |
| TCGA-77-8008-01 | 7.230136986 | 0 | 11.68812628 | High groups |
| TCGA-77-8009-01 | 1.663013699 | 1 | 9.638779803 | Low groups |
| TCGA-77-8128-01 | 3.150684932 | 0 | 10.96926326 | High groups |
| TCGA-77-8130-01 | 12.24931507 | 1 | 10.26494663 | High groups |
| TCGA-77-8131-01 | 1.049315068 | 0 | 10.18147133 | High groups |
| TCGA-77-8133-01 | 4.493150685 | 0 | 9.69420856 | Low groups |
| TCGA-77-8136-01 | 0.824657534 | 1 | 9.866597083 | Low groups |
| TCGA-77-8138-01 | 0.791780822 | 1 | 10.62498658 | High groups |
| TCGA-77-8139-01 | 8.673972603 | 0 | 10.05824749 | High groups |
| TCGA-77-8140-01 | 0.961643836 | 0 | 9.497270172 | Low groups |
| TCGA-77-8143-01 | 2.2 | 1 | 9.168528217 | Low groups |
| TCGA-77-8144-01 | 2.282191781 | 0 | 8.614694755 | Low groups |
| TCGA-77-8145-01 | 0.37260274 | 1 | 11.40241591 | High groups |
| TCGA-77-8146-01 | 8.736986301 | 0 | 9.605360616 | Low groups |
| TCGA-77-8148-01 | 5.542465753 | 0 | 10.19589727 | High groups |
| TCGA-77-8150-01 | 3.501369863 | 1 | 9.481841602 | Low groups |
| TCGA-77-8153-01 | 5.457534247 | 0 | 9.515697867 | Low groups |
| TCGA-77-8154-01 | 5.043835616 | 0 | 10.54641316 | High groups |
| TCGA-77-8156-01 | 3.030136986 | 0 | 10.51168991 | High groups |
| TCGA-77-A5FZ-01 | 10.51506849 | 0 | 10.36154544 | High groups |
| TCGA-77-A5G1-01 | 6.019178082 | 1 | 10.96484933 | High groups |
| TCGA-77-A5G3-01 | 12.52054795 | 0 | 10.16602345 | High groups |
| TCGA-77-A5G6-01 | 1.073972603 | 1 | 9.846772068 | Low groups |
| TCGA-77-A5G7-01 | 0.493150685 | 0 | 10.31252972 | High groups |
| TCGA-77-A5G8-01 | 5.161643836 | 0 | 10.91146936 | High groups |
| TCGA-77-A5GA-01 | 3.506849315 | 0 | 10.51676245 | High groups |
| TCGA-77-A5GB-01 | 0.62739726 | 1 | 11.42438572 | High groups |
| TCGA-77-A5GF-01 | 1.978082192 | 1 | 9.800384416 | Low groups |
| TCGA-77-A5GH-01 | 3.238356164 | 0 | 10.89762079 | High groups |
| TCGA-85-6175-01 | 0.805479452 | 1 | 10.06240899 | High groups |
| TCGA-85-6560-01 | 3.449315068 | 0 | 8.208816002 | Low groups |
| TCGA-85-6561-01 | 3.353424658 | 0 | 8.876626065 | Low groups |
| TCGA-85-6798-01 | 0.493150685 | 1 | 10.57668132 | High groups |
| TCGA-85-7696-01 | 3.043835616 | 0 | 10.71946848 | High groups |
| TCGA-85-7697-01 | 2.912328767 | 0 | 9.955591937 | Low groups |
| TCGA-85-7698-01 | 1.780821918 | 1 | 10.30543479 | High groups |
| TCGA-85-7699-01 | 2.082191781 | 1 | 9.879369433 | Low groups |
| TCGA-85-7710-01 | 0.115068493 | 0 | 9.383225402 | Low groups |
| TCGA-85-7843-01 | 0.095890411 | 0 | 10.03213405 | High groups |
| TCGA-85-7844-01 | 2.495890411 | 0 | 10.30811305 | High groups |
| TCGA-85-7950-01 | 1.578082192 | 0 | 10.58681287 | High groups |
| TCGA-85-8048-01 | 2.095890411 | 0 | 9.883755513 | Low groups |
| TCGA-85-8049-01 | 1.58630137 | 0 | 10.1420007 | High groups |
| TCGA-85-8052-01 | 2.010958904 | 0 | 10.69385778 | High groups |
| TCGA-85-8070-01 | 2.630136986 | 0 | 8.965448386 | Low groups |
| TCGA-85-8071-01 | 2.232876712 | 0 | 8.805949768 | Low groups |
| TCGA-85-8072-01 | 2.553424658 | 0 | 9.929760276 | Low groups |
| TCGA-85-8276-01 | 2.871232877 | 1 | 10.4558443 | High groups |
| TCGA-85-8287-01 | 0.063013699 | 0 | 9.696911903 | Low groups |
| TCGA-85-8288-01 | 1.101369863 | 0 | 11.00816856 | High groups |
| TCGA-85-8350-01 | 1.871232877 | 0 | 10.4337174 | High groups |
| TCGA-85-8351-01 | 1.397260274 | 0 | 8.77756286 | Low groups |
| TCGA-85-8352-01 | 0.350684932 | 1 | 5.292303372 | Low groups |
| TCGA-85-8353-01 | 0.257534247 | 1 | 9.702354873 | Low groups |
| TCGA-85-8354-01 | 2.726027397 | 0 | 9.296185133 | Low groups |
| TCGA-85-8355-01 | 0.167123288 | 0 | 9.708678008 | Low groups |
| TCGA-85-8479-01 | 1.282191781 | 0 | 9.093498115 | Low groups |
| TCGA-85-8481-01 | 0.646575342 | 0 | 10.21923618 | High groups |
| TCGA-85-8580-01 | 3.049315068 | 0 | 10.47896847 | High groups |
| TCGA-85-8582-01 | 3.178082192 | 0 | 11.59899227 | High groups |
| TCGA-85-8584-01 | 1.049315068 | 0 | 10.03993442 | High groups |
| TCGA-85-8664-01 | 1.189041096 | 1 | 9.596837332 | Low groups |
| TCGA-85-8666-01 | 1.882191781 | 0 | 9.998465475 | High groups |
| TCGA-85-A4CL-01 | 2.523287671 | 0 | 9.101009175 | Low groups |
| TCGA-85-A4CN-01 | 2.843835616 | 0 | 11.09942737 | High groups |
| TCGA-85-A4JB-01 | 2.580821918 | 0 | 10.41025958 | High groups |
| TCGA-85-A4JC-01 | 1.501369863 | 1 | 10.60712065 | High groups |
| TCGA-85-A4PA-01 | 2.030136986 | 0 | 10.1471978 | High groups |
| TCGA-85-A4QQ-01 | 2.539726027 | 0 | 9.925332938 | Low groups |
| TCGA-85-A4QR-01 | 1.643835616 | 0 | 9.095044086 | Low groups |
| TCGA-85-A50M-01 | 2.263013699 | 0 | 10.15084244 | High groups |
| TCGA-85-A50Z-01 | 1.265753425 | 1 | 8.877724867 | Low groups |
| TCGA-85-A510-01 | 0.994520548 | 1 | 8.382493303 | Low groups |
| TCGA-85-A511-01 | 1.068493151 | 1 | 10.66446533 | High groups |
| TCGA-85-A512-01 | 1.273972603 | 0 | 10.13191521 | High groups |
| TCGA-85-A513-01 | 2.493150685 | 0 | 9.418612893 | Low groups |
| TCGA-85-A53L-01 | 1.032876712 | 0 | 10.54787383 | High groups |
| TCGA-85-A5B5-01 | 0.304109589 | 0 | 10.0808491 | High groups |
| TCGA-90-6837-01 | 2.076712329 | 0 | 10.29942097 | High groups |
| TCGA-90-7766-01 | 0.791780822 | 1 | 9.336180583 | Low groups |
| TCGA-90-7767-01 | 0.243835616 | 0 | 9.95133499 | Low groups |
| TCGA-90-7769-01 | 0.980821918 | 0 | 9.68803074 | Low groups |
| TCGA-90-7964-01 | 1.17260274 | 0 | 10.78084993 | High groups |
| TCGA-90-A4ED-01 | 1.684931507 | 0 | 11.05210447 | High groups |
| TCGA-90-A4EE-01 | 1.884931507 | 0 | 9.750990641 | Low groups |
| TCGA-90-A59Q-01 | 0.750684932 | 1 | 9.799598138 | Low groups |
| TCGA-92-7340-01 | 0.224657534 | 0 | 10.67865225 | High groups |
| TCGA-92-7341-01 | 0.290410959 | 0 | 10.99408786 | High groups |
| TCGA-92-8063-01 | 0.334246575 | 0 | 10.64368747 | High groups |
| TCGA-92-8064-01 | 0.438356164 | 0 | 10.31267266 | High groups |
| TCGA-92-8065-01 | 0.191780822 | 0 | 8.761999927 | Low groups |
| TCGA-94-7033-01 | 1.753424658 | 0 | 9.755293787 | Low groups |
| TCGA-94-7557-01 | 0.01369863 | 0 | 10.92736717 | High groups |
| TCGA-94-7943-01 | 1.523287671 | 1 | 10.21282562 | High groups |
| TCGA-94-8035-01 | 0.334246575 | 0 | 9.750040714 | Low groups |
| TCGA-94-8490-01 | 0.419178082 | 0 | 9.252195889 | Low groups |
| TCGA-94-8491-01 | 1.756164384 | 1 | 10.14059207 | High groups |
| TCGA-94-A4VJ-01 | 1.178082192 | 0 | 10.34291825 | High groups |
| TCGA-94-A5I4-01 | 0.860273973 | 1 | 10.06981936 | High groups |
| TCGA-94-A5I6-01 | 1.317808219 | 1 | 10.69397692 | High groups |
| TCGA-96-7544-01 | 5.917808219 | 0 | 10.57336743 | High groups |
| TCGA-96-7545-01 | 4.638356164 | 1 | 10.37971571 | High groups |
| TCGA-96-8169-01 | 1.526027397 | 0 | 10.24283612 | High groups |
| TCGA-96-8170-01 | 1.454794521 | 0 | 10.62927374 | High groups |
| TCGA-96-A4JK-01 | 1.61369863 | 0 | 10.66176652 | High groups |
| TCGA-96-A4JL-01 | 2.306849315 | 0 | 10.13337254 | High groups |
| TCGA-98-7454-01 | 1.78630137 | 0 | 10.75103727 | High groups |
| TCGA-98-8020-01 | 0.030136986 | 1 | 10.69752538 | High groups |
| TCGA-98-8021-01 | 2.515068493 | 1 | 10.89048705 | High groups |
| TCGA-98-8022-01 | 2.556164384 | 0 | 11.21041017 | High groups |
| TCGA-98-8023-01 | 1.778082192 | 0 | 10.95972365 | High groups |
| TCGA-98-A538-01 | 2.263013699 | 0 | 10.76320855 | High groups |
| TCGA-98-A539-01 | 0.473972603 | 1 | 10.35342398 | High groups |
| TCGA-98-A53A-01 | 1.512328767 | 0 | 10.09274935 | High groups |
| TCGA-98-A53B-01 | 0.167123288 | 0 | 10.05547632 | High groups |
| TCGA-98-A53C-01 | 2.252054795 | 0 | 10.52150583 | High groups |
| TCGA-98-A53D-01 | 1.131506849 | 1 | 11.23456425 | High groups |
| TCGA-98-A53H-01 | 1.169863014 | 1 | 10.65071538 | High groups |
| TCGA-98-A53I-01 | 1.547945205 | 0 | 9.726660864 | Low groups |
| TCGA-98-A53J-01 | 1.726027397 | 0 | 9.714062679 | Low groups |
| TCGA-J1-A4AH-01 | 1.591780822 | 0 | 10.00971933 | High groups |
| TCGA-L3-A4E7-01 | 1.073972603 | 0 | 10.70637151 | High groups |
| TCGA-L3-A524-01 | 1.342465753 | 0 | 9.74640347 | Low groups |
| TCGA-LA-A446-01 | 1.098630137 | 0 | 9.429472441 | Low groups |
| TCGA-LA-A7SW-01 | 0.643835616 | 1 | 9.607234871 | Low groups |
| TCGA-MF-A522-01 | 0.473972603 | 1 | 8.729326397 | Low groups |
| TCGA-NC-A5HD-01 | 0.005479452 | 0 | 9.799290365 | Low groups |
| TCGA-NC-A5HE-01 | 6.4 | 0 | 9.392431932 | Low groups |
| TCGA-NC-A5HF-01 | 0.361643836 | 1 | 10.15348287 | High groups |
| TCGA-NC-A5HG-01 | 5.378082192 | 0 | 8.729459472 | Low groups |
| TCGA-NC-A5HH-01 | 0.101369863 | 0 | 9.112065166 | Low groups |
| TCGA-NC-A5HI-01 | 0.191780822 | 1 | 10.06124953 | High groups |
| TCGA-NC-A5HJ-01 | 0.898630137 | 1 | 9.748183959 | Low groups |
| TCGA-NC-A5HK-01 | 0.350684932 | 0 | 10.48350252 | High groups |
| TCGA-NC-A5HL-01 | 0.24109589 | 0 | 9.417554327 | Low groups |
| TCGA-NC-A5HM-01 | 3.320547945 | 0 | 9.140134963 | Low groups |
| TCGA-NC-A5HN-01 | 4.106849315 | 0 | 7.175224374 | Low groups |
| TCGA-NC-A5HO-01 | 3.660273973 | 0 | 10.25973361 | High groups |
| TCGA-NC-A5HP-01 | 0.868493151 | 1 | 10.86413258 | High groups |
| TCGA-NC-A5HQ-01 | 1.22739726 | 0 | 9.720886752 | Low groups |
| TCGA-NC-A5HR-01 | 3.408219178 | 0 | 9.633188694 | Low groups |
| TCGA-NC-A5HT-01 | 0.556164384 | 1 | 10.40091123 | High groups |
| TCGA-NK-A5CR-01 | 6.964383562 | 0 | 10.20992083 | High groups |
| TCGA-NK-A5CT-01 | 5.452054795 | 1 | 9.8308495 | Low groups |
| TCGA-NK-A5CX-01 | 0.304109589 | 0 | 10.99054557 | High groups |
| TCGA-NK-A5D1-01 | 0.41369863 | 1 | 9.816912127 | Low groups |
| TCGA-NK-A7XE-01 | 0.035616438 | 0 | 9.895521815 | Low groups |
| TCGA-O2-A52N-01 | 2.756164384 | 0 | 10.87423069 | High groups |
| TCGA-O2-A52Q-01 | 0.238356164 | 1 | 8.701449771 | Low groups |
| TCGA-O2-A52S-01 | 0.673972603 | 1 | 8.005681582 | Low groups |
| TCGA-O2-A52V-01 | 1.890410959 | 1 | 9.999581079 | High groups |
| TCGA-O2-A52W-01 | 0.715068493 | 0 | 10.06427108 | High groups |
| TCGA-O2-A5IB-01 | 0.693150685 | 1 | 8.448425755 | Low groups |
| TCGA-XC-AA0X-01 | 0.016438356 | 0 | 9.810445193 | Low groups |

**Table S13.** Raw counts of RNA-sequencing data of hsa-miR-328-3p in LUSC from the TCGA.

| sampleID | time | Staus | RS | Label |
| --- | --- | --- | --- | --- |
| TCGA-18-3406-01 | 0.978082192 | 1 | 3.91454692 | Low groups |
| TCGA-18-3407-01 | 0.37260274 | 0 | 4.744790092 | Low groups |
| TCGA-18-3408-01 | 4.912328767 | 1 | 5.472698149 | High groups |
| TCGA-18-3410-01 | 0.4 | 0 | 2.837838718 | Low groups |
| TCGA-18-3411-01 | 9.797260274 | 0 | 3.755332532 | Low groups |
| TCGA-18-3412-01 | 0.747945205 | 1 | 4.714551611 | Low groups |
| TCGA-18-3414-01 | 1.961643836 | 0 | 5.26901631 | High groups |
| TCGA-18-3415-01 | 7.679452055 | 0 | 3.933019247 | Low groups |
| TCGA-18-3416-01 | 2.575342466 | 1 | 4.701213295 | Low groups |
| TCGA-18-3417-01 | 3.005479452 | 0 | 5.106322521 | Low groups |
| TCGA-18-3419-01 | 7.701369863 | 0 | 6.211148706 | High groups |
| TCGA-18-3421-01 | 7.246575342 | 0 | 4.966713958 | Low groups |
| TCGA-18-4086-01 | 0.232876712 | 0 | 5.025214525 | Low groups |
| TCGA-18-4721-01 | 12.86027397 | 0 | 5.907861539 | High groups |
| TCGA-18-5592-01 | 4.161643836 | 0 | 6.635573318 | High groups |
| TCGA-18-5595-01 | 2.265753425 | 0 | 6.342975127 | High groups |
| TCGA-21-1072-01 | 8.263013699 | 0 | 6.246339295 | High groups |
| TCGA-21-1075-01 | 5.846575342 | 0 | 6.37389212 | High groups |
| TCGA-21-1076-01 | 4.315068493 | 1 | 5.236112569 | High groups |
| TCGA-21-1077-01 | 1.764383562 | 1 | 4.184723589 | Low groups |
| TCGA-21-1078-01 | 0.704109589 | 1 | 5.367840354 | High groups |
| TCGA-21-1079-01 | 0.868493151 | 1 | 5.620200027 | High groups |
| TCGA-21-1080-01 | 10.20273973 | 0 | 4.320014679 | Low groups |
| TCGA-21-1083-01 | 3.602739726 | 0 | 5.061021739 | Low groups |
| TCGA-21-5782-01 | 2.635616438 | 0 | 5.632340058 | High groups |
| TCGA-21-5783-01 | 7.342465753 | 0 | 5.942094491 | High groups |
| TCGA-21-5784-01 | 3.473972603 | 0 | 5.787397044 | High groups |
| TCGA-21-5786-01 | 2.238356164 | 1 | 6.98371725 | High groups |
| TCGA-21-5787-01 | 0.282191781 | 1 | 5.639567036 | High groups |
| TCGA-21-A5DI-01 | 2.682191781 | 0 | 4.783098975 | Low groups |
| TCGA-22-0940-01 | 1.832876712 | 1 | 5.056718905 | Low groups |
| TCGA-22-0944-01 | 0.610958904 | 0 | 3.847039 | Low groups |
| TCGA-22-1011-01 | 0.145205479 | 0 | 5.547604246 | High groups |
| TCGA-22-1012-01 | 1.175342466 | 0 | 4.096775612 | Low groups |
| TCGA-22-4591-01 | 1.21369863 | 1 | 7.944051388 | High groups |
| TCGA-22-4593-01 | 2.923287671 | 0 | 6.577813395 | High groups |
| TCGA-22-4594-01 | 4.02739726 | 0 | 3.739976493 | Low groups |
| TCGA-22-4595-01 | 0.684931507 | 1 | 4.616915481 | Low groups |
| TCGA-22-4596-01 | 0.046575342 | 0 | 4.92121832 | Low groups |
| TCGA-22-4601-01 | 1.438356164 | 1 | 7.714486496 | High groups |
| TCGA-22-4604-01 | 0.838356164 | 1 | 4.749918542 | Low groups |
| TCGA-22-4605-01 | 2.668493151 | 0 | 5.83554466 | High groups |
| TCGA-22-4607-01 | 1.608219178 | 0 | 4.513241853 | Low groups |
| TCGA-22-4609-01 | 0.797260274 | 0 | 5.919447947 | High groups |
| TCGA-22-4613-01 | 0.980821918 | 0 | 6.768281472 | High groups |
| TCGA-22-5471-01 | 0.62739726 | 1 | 5.973412903 | High groups |
| TCGA-22-5472-01 | 2.043835616 | 1 | 7.096834781 | High groups |
| TCGA-22-5473-01 | 4.534246575 | 1 | 5.320823542 | High groups |
| TCGA-22-5474-01 | 1.219178082 | 0 | 6.039557496 | High groups |
| TCGA-22-5477-01 | 1.4 | 1 | 7.280869575 | High groups |
| TCGA-22-5478-01 | 0.065753425 | 0 | 7.005734235 | High groups |
| TCGA-22-5479-01 | 7.191780822 | 0 | 5.101477712 | Low groups |
| TCGA-22-5480-01 | 4.873972603 | 1 | 5.594315496 | High groups |
| TCGA-22-5481-01 | 0.947945205 | 1 | 6.692577843 | High groups |
| TCGA-22-5482-01 | 0.978082192 | 0 | 6.410388257 | High groups |
| TCGA-22-5483-01 | 1.556164384 | 1 | 4.267164215 | Low groups |
| TCGA-22-5485-01 | 2.301369863 | 1 | 7.898817209 | High groups |
| TCGA-22-5489-01 | 1.17260274 | 1 | 5.748907364 | High groups |
| TCGA-22-5491-01 | 4.693150685 | 0 | 6.320918938 | High groups |
| TCGA-22-5492-01 | 1.38630137 | 0 | 7.238076991 | High groups |
| TCGA-22-A5C4-01 | 1.838356164 | 0 | 6.850080671 | High groups |
| TCGA-33-4532-01 | 10.75068493 | 0 | 7.074490558 | High groups |
| TCGA-33-4533-01 | 11.14520548 | 0 | 5.212280693 | High groups |
| TCGA-33-4538-01 | 8.161643836 | 0 | 5.689835486 | High groups |
| TCGA-33-4547-01 | 6.62739726 | 0 | 4.876787632 | Low groups |
| TCGA-33-4566-01 | 14.48493151 | 0 | 4.641020991 | Low groups |
| TCGA-33-4582-01 | 8.008219178 | 1 | 5.887402758 | High groups |
| TCGA-33-4583-01 | 12.60547945 | 0 | 3.919765105 | Low groups |
| TCGA-33-4586-01 | 0.463013699 | 1 | 4.161679968 | Low groups |
| TCGA-33-4587-01 | 4.536986301 | 1 | 6.727669662 | High groups |
| TCGA-33-4589-01 | 0.128767123 | 0 | 5.012109244 | Low groups |
| TCGA-33-6737-01 | 1.646575342 | 1 | 3.47280344 | Low groups |
| TCGA-33-6738-01 | 5.279452055 | 0 | 6.45116412 | High groups |
| TCGA-33-A4WN-01 | 0.391780822 | 0 | 4.595926274 | Low groups |
| TCGA-33-A5GW-01 | 0.024657534 | 0 | 4.774250567 | Low groups |
| TCGA-33-AAS8-01 | 3.052054795 | 0 | 5.143118479 | High groups |
| TCGA-33-AASB-01 | 0.082191781 | 1 | 3.85399879 | Low groups |
| TCGA-33-AASD-01 | 5.238356164 | 1 | 6.794141728 | High groups |
| TCGA-33-AASI-01 | 3.682191781 | 1 | 4.616843925 | Low groups |
| TCGA-33-AASJ-01 | 3.109589041 | 1 | 4.297624307 | Low groups |
| TCGA-33-AASL-01 | 0.408219178 | 1 | 2.670132476 | Low groups |
| TCGA-34-2596-01 | 0.219178082 | 0 | 3.813926659 | Low groups |
| TCGA-34-2600-01 | 5.134246575 | 0 | 6.671932195 | High groups |
| TCGA-34-2604-01 | 2.624657534 | 0 | 5.164613894 | High groups |
| TCGA-34-2605-01 | 1.079452055 | 1 | 4.574927944 | Low groups |
| TCGA-34-2608-01 | 2.739726027 | 0 | 4.873016445 | Low groups |
| TCGA-34-2609-01 | 1.060273973 | 1 | 5.592590387 | High groups |
| TCGA-34-5231-01 | 5.435616438 | 0 | 5.013502592 | Low groups |
| TCGA-34-5232-01 | 6.769863014 | 0 | 4.817171449 | Low groups |
| TCGA-34-5234-01 | 6.221917808 | 0 | 4.436094528 | Low groups |
| TCGA-34-5236-01 | 0.309589041 | 1 | 3.555751257 | Low groups |
| TCGA-34-5239-01 | 4.191780822 | 1 | 4.662960836 | Low groups |
| TCGA-34-5240-01 | 4.221917808 | 0 | 4.647464586 | Low groups |
| TCGA-34-5241-01 | 1.410958904 | 0 | 4.676881642 | Low groups |
| TCGA-34-5927-01 | 0.671232877 | 1 | 4.701284668 | Low groups |
| TCGA-34-5928-01 | 3.276712329 | 0 | 5.178088833 | High groups |
| TCGA-34-5929-01 | 0.41369863 | 0 | 4.61752371 | Low groups |
| TCGA-34-7107-01 | 0.093150685 | 0 | 5.868430533 | High groups |
| TCGA-34-8454-01 | 3.232876712 | 0 | 6.517874917 | High groups |
| TCGA-34-8455-01 | 0.336986301 | 1 | 5.992362978 | High groups |
| TCGA-34-8456-01 | 2.202739726 | 0 | 7.694010972 | High groups |
| TCGA-34-A5IX-01 | 2.824657534 | 0 | 3.725669947 | Low groups |
| TCGA-37-3783-01 | 0.334246575 | 0 | 5.641342789 | High groups |
| TCGA-37-3789-01 | 0.035616438 | 0 | 2.731990404 | Low groups |
| TCGA-37-3792-01 | 0.032876712 | 0 | 3.969537607 | Low groups |
| TCGA-37-4130-01 | 0.676712329 | 0 | 4.397728361 | Low groups |
| TCGA-37-4133-01 | 0.652054795 | 0 | 5.834227506 | High groups |
| TCGA-37-4135-01 | 0.567123288 | 0 | 4.702903678 | Low groups |
| TCGA-37-4141-01 | 0.032876712 | 0 | 6.454353011 | High groups |
| TCGA-37-5819-01 | 0.282191781 | 0 | 6.369893488 | High groups |
| TCGA-37-A5EL-01 | 2.591780822 | 1 | 4.689098288 | Low groups |
| TCGA-37-A5EM-01 | 2.375342466 | 0 | 3.329840172 | Low groups |
| TCGA-37-A5EN-01 | 1.808219178 | 0 | 5.936975737 | High groups |
| TCGA-39-5011-01 | 1.547945205 | 1 | 4.925946645 | Low groups |
| TCGA-39-5019-01 | 1.035616438 | 1 | 3.265534563 | Low groups |
| TCGA-39-5021-01 | 5.136986301 | 1 | 4.827911171 | Low groups |
| TCGA-39-5022-01 | 3.967123288 | 1 | 4.925185443 | Low groups |
| TCGA-39-5024-01 | 6.876712329 | 0 | 4.403828873 | Low groups |
| TCGA-39-5027-01 | 8.44109589 | 1 | 4.316110833 | Low groups |
| TCGA-39-5028-01 | 0.142465753 | 0 | 4.679760785 | Low groups |
| TCGA-39-5029-01 | 1.345205479 | 1 | 5.186172293 | High groups |
| TCGA-39-5030-01 | 0.161643836 | 0 | 4.642579937 | Low groups |
| TCGA-39-5031-01 | 5.043835616 | 0 | 5.671032186 | High groups |
| TCGA-39-5034-01 | 0.964383562 | 1 | 5.523244403 | High groups |
| TCGA-39-5035-01 | 5.698630137 | 0 | 3.849124974 | Low groups |
| TCGA-39-5036-01 | 5.931506849 | 0 | 5.483474455 | High groups |
| TCGA-39-5037-01 | 4.630136986 | 0 | 4.837609467 | Low groups |
| TCGA-39-5039-01 | 1.490410959 | 0 | 4.777773021 | Low groups |
| TCGA-39-5040-01 | 1.210958904 | 1 | 4.94281099 | Low groups |
| TCGA-43-2576-01 | 3.350684932 | 0 | 4.03006791 | Low groups |
| TCGA-43-2578-01 | 1.873972603 | 0 | 5.12659405 | Low groups |
| TCGA-43-2581-01 | 3.221917808 | 0 | 3.939960811 | Low groups |
| TCGA-43-3394-01 | 1.512328767 | 1 | 5.379493085 | High groups |
| TCGA-43-3920-01 | 2.75890411 | 0 | 4.066127432 | Low groups |
| TCGA-43-5668-01 | 1.287671233 | 1 | 6.431696998 | High groups |
| TCGA-43-5670-01 | 2.326027397 | 0 | 5.264160208 | High groups |
| TCGA-43-6143-01 | 1.915068493 | 0 | 5.517193476 | High groups |
| TCGA-43-6647-01 | 2.073972603 | 0 | 5.001183291 | Low groups |
| TCGA-43-6770-01 | 1.789041096 | 0 | 4.960327238 | Low groups |
| TCGA-43-6771-01 | 0.421917808 | 1 | 3.517709948 | Low groups |
| TCGA-43-6773-01 | 0.317808219 | 0 | 5.289050469 | High groups |
| TCGA-43-7656-01 | 1.632876712 | 0 | 5.663095514 | High groups |
| TCGA-43-7657-01 | 0.646575342 | 0 | 6.095644291 | High groups |
| TCGA-43-7658-01 | 6.315068493 | 1 | 5.662288515 | High groups |
| TCGA-43-8115-01 | 1.115068493 | 0 | 4.791191547 | Low groups |
| TCGA-43-8116-01 | 0.980821918 | 0 | 4.373744805 | Low groups |
| TCGA-43-8118-01 | 0.243835616 | 0 | 5.55278374 | High groups |
| TCGA-43-A474-01 | 0.967123288 | 0 | 4.78183284 | Low groups |
| TCGA-43-A475-01 | 0.810958904 | 0 | 5.165140715 | High groups |
| TCGA-43-A56U-01 | 1.183561644 | 0 | 4.306089112 | Low groups |
| TCGA-43-A56V-01 | 0.97260274 | 1 | 4.965135688 | Low groups |
| TCGA-46-3765-01 | 1.109589041 | 0 | 3.685943399 | Low groups |
| TCGA-46-3766-01 | 1.01369863 | 0 | 5.196261436 | High groups |
| TCGA-46-3767-01 | 1.084931507 | 0 | 3.326751752 | Low groups |
| TCGA-46-3768-01 | 0.819178082 | 0 | 4.956220341 | Low groups |
| TCGA-46-3769-01 | 0.369863014 | 0 | 3.68714391 | Low groups |
| TCGA-46-6025-01 | 0.887671233 | 0 | 4.405071463 | Low groups |
| TCGA-46-6026-01 | 1.15890411 | 0 | 4.168893667 | Low groups |
| TCGA-51-4079-01 | 0.032876712 | 0 | 4.530953423 | Low groups |
| TCGA-51-4080-01 | 0.032876712 | 0 | 6.32819757 | High groups |
| TCGA-51-4081-01 | 2.495890411 | 0 | 4.525592594 | Low groups |
| TCGA-51-6867-01 | 2.15890411 | 1 | 7.352529467 | High groups |
| TCGA-52-7622-01 | 2.361643836 | 0 | 5.563915438 | High groups |
| TCGA-52-7809-01 | 0.454794521 | 1 | 5.734611947 | High groups |
| TCGA-52-7810-01 | 2.528767123 | 0 | 5.06954171 | Low groups |
| TCGA-52-7811-01 | 0.419178082 | 1 | 5.298131504 | High groups |
| TCGA-52-7812-01 | 2.167123288 | 1 | 4.783316307 | Low groups |
| TCGA-56-1622-01 | 2.41369863 | 1 | 6.051811348 | High groups |
| TCGA-56-5897-01 | 1.035616438 | 0 | 5.478186746 | High groups |
| TCGA-56-5898-01 | 1.520547945 | 0 | 5.885903765 | High groups |
| TCGA-56-6545-01 | 1.824657534 | 0 | 5.73905035 | High groups |
| TCGA-56-7221-01 | 1.665753425 | 0 | 4.616416808 | Low groups |
| TCGA-56-7222-01 | 1.169863014 | 1 | 4.942664913 | Low groups |
| TCGA-56-7223-01 | 0.361643836 | 1 | 7.471455664 | High groups |
| TCGA-56-7579-01 | 0.408219178 | 1 | 4.856060137 | Low groups |
| TCGA-56-7580-01 | 2.534246575 | 0 | 4.495599236 | Low groups |
| TCGA-56-7582-01 | 1.646575342 | 0 | 4.805159325 | Low groups |
| TCGA-56-7730-01 | 0.542465753 | 1 | 5.878158426 | High groups |
| TCGA-56-7731-01 | 0.008219178 | 0 | 6.033731551 | High groups |
| TCGA-56-7822-01 | 0.824657534 | 1 | 5.95692194 | High groups |
| TCGA-56-7823-01 | 2.769863014 | 0 | 5.307855077 | High groups |
| TCGA-56-8082-01 | 1.246575342 | 0 | 5.540874133 | High groups |
| TCGA-56-8083-01 | 0.410958904 | 0 | 6.640919916 | High groups |
| TCGA-56-8201-01 | 1.087671233 | 1 | 4.568217417 | Low groups |
| TCGA-56-8304-01 | 0.290410959 | 0 | 5.214872699 | High groups |
| TCGA-56-8305-01 | 0.287671233 | 0 | 4.992394795 | Low groups |
| TCGA-56-8307-01 | 2.24109589 | 0 | 4.178972726 | Low groups |
| TCGA-56-8308-01 | 1.416438356 | 0 | 5.045913395 | Low groups |
| TCGA-56-8309-01 | 1.17260274 | 0 | 5.282191686 | High groups |
| TCGA-56-8503-01 | 0.112328767 | 0 | 5.402965311 | High groups |
| TCGA-56-8504-01 | 1.397260274 | 0 | 5.99191407 | High groups |
| TCGA-56-8622-01 | 0.150684932 | 0 | 5.147479169 | High groups |
| TCGA-56-8623-01 | 1.767123288 | 1 | 6.059401789 | High groups |
| TCGA-56-8624-01 | 1.150684932 | 0 | 6.349234867 | High groups |
| TCGA-56-8625-01 | 0.745205479 | 1 | 5.67343072 | High groups |
| TCGA-56-8626-01 | 0.82739726 | 0 | 5.463731158 | High groups |
| TCGA-56-8628-01 | 1.687671233 | 0 | 7.408577706 | High groups |
| TCGA-56-8629-01 | 1.317808219 | 0 | 5.805304323 | High groups |
| TCGA-56-A49D-01 | 1.745205479 | 0 | 5.319655865 | High groups |
| TCGA-56-A4BW-01 | 1.602739726 | 0 | 4.974980543 | Low groups |
| TCGA-56-A4BX-01 | 1.109589041 | 0 | 6.353974898 | High groups |
| TCGA-56-A4BY-01 | 1.487671233 | 1 | 5.711196535 | High groups |
| TCGA-56-A4ZJ-01 | 1.753424658 | 0 | 5.256222504 | High groups |
| TCGA-56-A4ZK-01 | 1.561643836 | 0 | 5.355409843 | High groups |
| TCGA-56-A5DR-01 | 0.010958904 | 0 | 3.581736035 | Low groups |
| TCGA-56-A62T-01 | 1.205479452 | 0 | 5.725706806 | High groups |
| TCGA-58-8386-01 | 0.002739726 | 0 | 5.571068481 | High groups |
| TCGA-58-8387-01 | 1.104109589 | 0 | 3.735694272 | Low groups |
| TCGA-58-8388-01 | 1.128767123 | 0 | 6.602511367 | High groups |
| TCGA-58-8390-01 | 2.495890411 | 0 | 5.174569288 | High groups |
| TCGA-58-8391-01 | 5.569863014 | 1 | 5.03694636 | Low groups |
| TCGA-58-8392-01 | 0.263013699 | 1 | 5.97829678 | High groups |
| TCGA-58-8393-01 | 2.898630137 | 0 | 5.611395319 | High groups |
| TCGA-58-A46J-01 | 7.093150685 | 0 | 4.744236512 | Low groups |
| TCGA-58-A46K-01 | 2.01369863 | 1 | 4.411981861 | Low groups |
| TCGA-58-A46L-01 | 4.720547945 | 0 | 5.993836011 | High groups |
| TCGA-58-A46M-01 | 2.936986301 | 0 | 4.624252762 | Low groups |
| TCGA-58-A46N-01 | 1.726027397 | 1 | 4.486231935 | Low groups |
| TCGA-60-2695-01 | 1.75890411 | 0 | 3.920212587 | Low groups |
| TCGA-60-2696-01 | 0.298630137 | 0 | 6.594434939 | High groups |
| TCGA-60-2697-01 | 0.547945205 | 1 | 4.579057142 | Low groups |
| TCGA-60-2698-01 | 0.312328767 | 1 | 4.200500665 | Low groups |
| TCGA-60-2703-01 | 4.81369863 | 1 | 5.900792752 | High groups |
| TCGA-60-2704-01 | 1.457534247 | 1 | 5.397417427 | High groups |
| TCGA-60-2706-01 | 7.726027397 | 0 | 4.853311851 | Low groups |
| TCGA-60-2708-01 | 6.704109589 | 0 | 4.971621078 | Low groups |
| TCGA-60-2709-01 | 4.123287671 | 0 | 4.67105413 | Low groups |
| TCGA-60-2710-01 | 5.545205479 | 0 | 5.032328605 | Low groups |
| TCGA-60-2711-01 | 3.452054795 | 0 | 5.337392123 | High groups |
| TCGA-60-2712-01 | 0.750684932 | 0 | 5.866080911 | High groups |
| TCGA-60-2713-01 | 3.715068493 | 1 | 4.974112826 | Low groups |
| TCGA-60-2714-01 | 4.194520548 | 0 | 6.40708528 | High groups |
| TCGA-60-2716-01 | 4.04109589 | 0 | 6.08361399 | High groups |
| TCGA-60-2719-01 | 3.553424658 | 0 | 4.686250233 | Low groups |
| TCGA-60-2720-01 | 0.265753425 | 0 | 3.84668486 | Low groups |
| TCGA-60-2721-01 | 2.693150685 | 0 | 4.490135845 | Low groups |
| TCGA-60-2722-01 | 2.331506849 | 1 | 6.171447744 | High groups |
| TCGA-60-2723-01 | 2.991780822 | 0 | 3.256245606 | Low groups |
| TCGA-60-2724-01 | 1.964383562 | 0 | 6.26787265 | High groups |
| TCGA-60-2725-01 | 2.235616438 | 0 | 5.070519293 | Low groups |
| TCGA-63-6202-01 | 4.389041096 | 0 | 4.864426962 | Low groups |
| TCGA-63-7020-01 | 5.843835616 | 0 | 6.314553518 | High groups |
| TCGA-63-7021-01 | 4.117808219 | 1 | 4.816067173 | Low groups |
| TCGA-63-7022-01 | 5.679452055 | 0 | 5.879893197 | High groups |
| TCGA-63-A5M9-01 | 0 | 0 | 6.264475629 | High groups |
| TCGA-63-A5MB-01 | 8.556164384 | 0 | 5.155617293 | High groups |
| TCGA-63-A5MG-01 | 5.884931507 | 0 | 3.917833382 | Low groups |
| TCGA-63-A5MH-01 | 5.550684932 | 0 | 4.807093101 | Low groups |
| TCGA-63-A5MI-01 | 4.887671233 | 0 | 5.726997107 | High groups |
| TCGA-63-A5MJ-01 | 4.997260274 | 0 | 4.503464757 | Low groups |
| TCGA-63-A5ML-01 | 3.797260274 | 0 | 4.451130319 | Low groups |
| TCGA-63-A5MM-01 | 0.597260274 | 1 | 4.767523075 | Low groups |
| TCGA-63-A5MN-01 | 0.876712329 | 1 | 4.413044505 | Low groups |
| TCGA-63-A5MP-01 | 1.4 | 1 | 4.515715353 | Low groups |
| TCGA-63-A5MR-01 | 7.44109589 | 0 | 4.918268915 | Low groups |
| TCGA-63-A5MS-01 | 6.523287671 | 0 | 5.528833705 | High groups |
| TCGA-63-A5MT-01 | 1.169863014 | 1 | 4.939381231 | Low groups |
| TCGA-63-A5MU-01 | 1.150684932 | 1 | 5.323393741 | High groups |
| TCGA-63-A5MV-01 | 3.01369863 | 0 | 6.102545705 | High groups |
| TCGA-63-A5MW-01 | 4.490410959 | 0 | 6.39474066 | High groups |
| TCGA-63-A5MY-01 | 2.882191781 | 0 | 5.813624064 | High groups |
| TCGA-66-2727-01 | 1.41369863 | 0 | 4.944389213 | Low groups |
| TCGA-66-2734-01 | 3.591780822 | 0 | 5.305990469 | High groups |
| TCGA-66-2737-01 | 0.167123288 | 0 | 4.476711214 | Low groups |
| TCGA-66-2742-01 | 1.756164384 | 0 | 5.643170949 | High groups |
| TCGA-66-2744-01 | 0.082191781 | 0 | 3.037946116 | Low groups |
| TCGA-66-2753-01 | 0.084931507 | 0 | 5.482245916 | High groups |
| TCGA-66-2754-01 | 0.167123288 | 0 | 4.569046865 | Low groups |
| TCGA-66-2755-01 | 0.076712329 | 0 | 4.57836001 | Low groups |
| TCGA-66-2758-01 | 1.750684932 | 0 | 4.92567207 | Low groups |
| TCGA-66-2759-01 | 2.087671233 | 0 | 5.255867516 | High groups |
| TCGA-66-2763-01 | 0.082191781 | 0 | 6.392155712 | High groups |
| TCGA-66-2765-01 | 0.167123288 | 0 | 4.205104931 | Low groups |
| TCGA-66-2766-01 | 0.084931507 | 0 | 4.490872307 | Low groups |
| TCGA-66-2767-01 | 0.167123288 | 0 | 3.931604728 | Low groups |
| TCGA-66-2768-01 | 0.167123288 | 0 | 4.758453494 | Low groups |
| TCGA-66-2770-01 | 1.917808219 | 0 | 5.14950936 | High groups |
| TCGA-66-2771-01 | 1.583561644 | 0 | 4.356088299 | Low groups |
| TCGA-66-2777-01 | 0.167123288 | 0 | 4.862770891 | Low groups |
| TCGA-66-2780-01 | 1.002739726 | 0 | 4.237816535 | Low groups |
| TCGA-66-2781-01 | 0.331506849 | 0 | 4.854198518 | Low groups |
| TCGA-66-2782-01 | 0.832876712 | 1 | 4.247131154 | Low groups |
| TCGA-66-2783-01 | 2.079452055 | 0 | 4.246960204 | Low groups |
| TCGA-66-2785-01 | 0.164383562 | 0 | 4.357483176 | Low groups |
| TCGA-66-2786-01 | 2.164383562 | 0 | 5.290621758 | High groups |
| TCGA-66-2787-01 | 3.334246575 | 0 | 5.234391643 | High groups |
| TCGA-66-2788-01 | 1.915068493 | 0 | 4.835547843 | Low groups |
| TCGA-66-2789-01 | 0.336986301 | 0 | 4.690819787 | Low groups |
| TCGA-66-2790-01 | 1.915068493 | 1 | 6.233312029 | High groups |
| TCGA-66-2791-01 | 0.419178082 | 0 | 4.461864704 | Low groups |
| TCGA-66-2792-01 | 2.501369863 | 0 | 5.597454002 | High groups |
| TCGA-66-2793-01 | 0.838356164 | 0 | 6.061614857 | High groups |
| TCGA-66-2794-01 | 4.506849315 | 0 | 4.554194911 | Low groups |
| TCGA-66-2795-01 | 0.334246575 | 0 | 4.810059294 | Low groups |
| TCGA-66-2800-01 | 4.087671233 | 0 | 5.682199822 | High groups |
| TCGA-68-7755-01 | 0.22739726 | 0 | 5.004066136 | Low groups |
| TCGA-68-7756-01 | 0.553424658 | 0 | 5.196272532 | High groups |
| TCGA-68-7757-01 | 0.578082192 | 0 | 5.966840412 | High groups |
| TCGA-68-8250-01 | 0.668493151 | 0 | 6.21919718 | High groups |
| TCGA-68-8251-01 | 1.112328767 | 0 | 4.376249953 | Low groups |
| TCGA-68-A59I-01 | 0.435616438 | 1 | 3.758749819 | Low groups |
| TCGA-68-A59J-01 | 1.22739726 | 0 | 4.392917802 | Low groups |
| TCGA-70-6722-01 | 0.802739726 | 1 | 4.747795926 | Low groups |
| TCGA-70-6723-01 | 0.854794521 | 1 | 5.311903623 | High groups |
| TCGA-77-6842-01 | 0.550684932 | 1 | 5.975572411 | High groups |
| TCGA-77-6843-01 | 6.093150685 | 0 | 7.197281085 | High groups |
| TCGA-77-6844-01 | 4.898630137 | 1 | 7.441351259 | High groups |
| TCGA-77-6845-01 | 1.939726027 | 0 | 8.448024598 | High groups |
| TCGA-77-7138-01 | 0.931506849 | 0 | 5.294817604 | High groups |
| TCGA-77-7139-01 | 11.6739726 | 0 | 5.482663966 | High groups |
| TCGA-77-7140-01 | 0.684931507 | 1 | 5.391028727 | High groups |
| TCGA-77-7141-01 | 0.04109589 | 0 | 5.994549992 | High groups |
| TCGA-77-7142-01 | 6.101369863 | 0 | 6.138858693 | High groups |
| TCGA-77-7335-01 | 5.843835616 | 0 | 5.046791634 | Low groups |
| TCGA-77-7337-01 | 8.912328767 | 0 | 4.590351289 | Low groups |
| TCGA-77-7338-01 | 0.01369863 | 0 | 4.914123898 | Low groups |
| TCGA-77-7463-01 | 3.898630137 | 0 | 5.396372219 | High groups |
| TCGA-77-7465-01 | 2.712328767 | 0 | 5.394369536 | High groups |
| TCGA-77-8007-01 | 0.380821918 | 1 | 6.628044628 | High groups |
| TCGA-77-8008-01 | 7.230136986 | 0 | 4.972851525 | Low groups |
| TCGA-77-8009-01 | 1.663013699 | 1 | 6.28087978 | High groups |
| TCGA-77-8128-01 | 3.150684932 | 0 | 5.20021658 | High groups |
| TCGA-77-8130-01 | 12.24931507 | 1 | 4.525308253 | Low groups |
| TCGA-77-8131-01 | 1.049315068 | 0 | 4.470769942 | Low groups |
| TCGA-77-8133-01 | 4.493150685 | 0 | 5.044964527 | Low groups |
| TCGA-77-8136-01 | 0.824657534 | 1 | 4.699995744 | Low groups |
| TCGA-77-8138-01 | 0.791780822 | 1 | 5.431714746 | High groups |
| TCGA-77-8139-01 | 8.673972603 | 0 | 4.1053187 | Low groups |
| TCGA-77-8140-01 | 0.961643836 | 0 | 3.956579999 | Low groups |
| TCGA-77-8143-01 | 2.2 | 1 | 5.258939913 | High groups |
| TCGA-77-8144-01 | 2.282191781 | 0 | 4.660236177 | Low groups |
| TCGA-77-8145-01 | 0.37260274 | 1 | 4.913352026 | Low groups |
| TCGA-77-8146-01 | 8.736986301 | 0 | 5.244453843 | High groups |
| TCGA-77-8148-01 | 5.542465753 | 0 | 5.39683782 | High groups |
| TCGA-77-8150-01 | 3.501369863 | 1 | 3.55301097 | Low groups |
| TCGA-77-8153-01 | 5.457534247 | 0 | 5.582225488 | High groups |
| TCGA-77-8154-01 | 5.043835616 | 0 | 6.357600563 | High groups |
| TCGA-77-8156-01 | 3.030136986 | 0 | 5.015067716 | Low groups |
| TCGA-77-A5FZ-01 | 10.51506849 | 0 | 4.059796361 | Low groups |
| TCGA-77-A5G1-01 | 6.019178082 | 1 | 5.025203804 | Low groups |
| TCGA-77-A5G3-01 | 12.52054795 | 0 | 4.738654794 | Low groups |
| TCGA-77-A5G6-01 | 1.073972603 | 1 | 6.128772006 | High groups |
| TCGA-77-A5G7-01 | 0.493150685 | 0 | 4.487237496 | Low groups |
| TCGA-77-A5G8-01 | 5.161643836 | 0 | 3.752730718 | Low groups |
| TCGA-77-A5GA-01 | 3.506849315 | 0 | 3.897086659 | Low groups |
| TCGA-77-A5GB-01 | 0.62739726 | 1 | 3.239997054 | Low groups |
| TCGA-77-A5GF-01 | 1.978082192 | 1 | 4.726920565 | Low groups |
| TCGA-77-A5GH-01 | 3.238356164 | 0 | 4.463637496 | Low groups |
| TCGA-85-6175-01 | 0.805479452 | 1 | 4.723715109 | Low groups |
| TCGA-85-6560-01 | 3.449315068 | 0 | 5.035607635 | Low groups |
| TCGA-85-6561-01 | 3.353424658 | 0 | 3.97738242 | Low groups |
| TCGA-85-6798-01 | 0.493150685 | 1 | 5.055986472 | Low groups |
| TCGA-85-7696-01 | 3.043835616 | 0 | 5.532976122 | High groups |
| TCGA-85-7697-01 | 2.912328767 | 0 | 5.990030809 | High groups |
| TCGA-85-7698-01 | 1.780821918 | 1 | 5.421715954 | High groups |
| TCGA-85-7699-01 | 2.082191781 | 1 | 5.486904961 | High groups |
| TCGA-85-7710-01 | 0.115068493 | 0 | 6.16626172 | High groups |
| TCGA-85-7843-01 | 0.095890411 | 0 | 5.949610801 | High groups |
| TCGA-85-7844-01 | 2.495890411 | 0 | 5.771253495 | High groups |
| TCGA-85-7950-01 | 1.578082192 | 0 | 5.439030248 | High groups |
| TCGA-85-8048-01 | 2.095890411 | 0 | 5.212796542 | High groups |
| TCGA-85-8049-01 | 1.58630137 | 0 | 4.968227179 | Low groups |
| TCGA-85-8052-01 | 2.010958904 | 0 | 5.281854898 | High groups |
| TCGA-85-8070-01 | 2.630136986 | 0 | 7.143682794 | High groups |
| TCGA-85-8071-01 | 2.232876712 | 0 | 5.991839351 | High groups |
| TCGA-85-8072-01 | 2.553424658 | 0 | 5.886197234 | High groups |
| TCGA-85-8276-01 | 2.871232877 | 1 | 4.484196212 | Low groups |
| TCGA-85-8287-01 | 0.063013699 | 0 | 5.762267668 | High groups |
| TCGA-85-8288-01 | 1.101369863 | 0 | 4.914468085 | Low groups |
| TCGA-85-8350-01 | 1.871232877 | 0 | 5.643155447 | High groups |
| TCGA-85-8351-01 | 1.397260274 | 0 | 6.179375194 | High groups |
| TCGA-85-8352-01 | 0.350684932 | 1 | 5.637465923 | High groups |
| TCGA-85-8353-01 | 0.257534247 | 1 | 4.092212259 | Low groups |
| TCGA-85-8354-01 | 2.726027397 | 0 | 6.314743104 | High groups |
| TCGA-85-8355-01 | 0.167123288 | 0 | 5.915143853 | High groups |
| TCGA-85-8479-01 | 1.282191781 | 0 | 5.845995708 | High groups |
| TCGA-85-8481-01 | 0.646575342 | 0 | 4.576984923 | Low groups |
| TCGA-85-8580-01 | 3.049315068 | 0 | 6.208006124 | High groups |
| TCGA-85-8582-01 | 3.178082192 | 0 | 5.628482694 | High groups |
| TCGA-85-8584-01 | 1.049315068 | 0 | 4.128744173 | Low groups |
| TCGA-85-8664-01 | 1.189041096 | 1 | 5.784572295 | High groups |
| TCGA-85-8666-01 | 1.882191781 | 0 | 5.174361433 | High groups |
| TCGA-85-A4CL-01 | 2.523287671 | 0 | 4.468457027 | Low groups |
| TCGA-85-A4CN-01 | 2.843835616 | 0 | 4.392544458 | Low groups |
| TCGA-85-A4JB-01 | 2.580821918 | 0 | 4.708660532 | Low groups |
| TCGA-85-A4JC-01 | 1.501369863 | 1 | 3.868375932 | Low groups |
| TCGA-85-A4PA-01 | 2.030136986 | 0 | 6.352203957 | High groups |
| TCGA-85-A4QQ-01 | 2.539726027 | 0 | 3.781982299 | Low groups |
| TCGA-85-A4QR-01 | 1.643835616 | 0 | 5.269651472 | High groups |
| TCGA-85-A50M-01 | 2.263013699 | 0 | 4.478975557 | Low groups |
| TCGA-85-A50Z-01 | 1.265753425 | 1 | 6.033410226 | High groups |
| TCGA-85-A510-01 | 0.994520548 | 1 | 6.807453975 | High groups |
| TCGA-85-A511-01 | 1.068493151 | 1 | 4.068702536 | Low groups |
| TCGA-85-A512-01 | 1.273972603 | 0 | 5.204419335 | High groups |
| TCGA-85-A513-01 | 2.493150685 | 0 | 4.75959614 | Low groups |
| TCGA-85-A53L-01 | 1.032876712 | 0 | 5.272738833 | High groups |
| TCGA-85-A5B5-01 | 0.304109589 | 0 | 5.397535203 | High groups |
| TCGA-90-6837-01 | 2.076712329 | 0 | 6.310828329 | High groups |
| TCGA-90-7766-01 | 0.791780822 | 1 | 6.04558988 | High groups |
| TCGA-90-7767-01 | 0.243835616 | 0 | 5.396573684 | High groups |
| TCGA-90-7769-01 | 0.980821918 | 0 | 5.963200726 | High groups |
| TCGA-90-7964-01 | 1.17260274 | 0 | 5.391845722 | High groups |
| TCGA-90-A4ED-01 | 1.684931507 | 0 | 5.252555575 | High groups |
| TCGA-90-A4EE-01 | 1.884931507 | 0 | 5.493497568 | High groups |
| TCGA-90-A59Q-01 | 0.750684932 | 1 | 4.7402904 | Low groups |
| TCGA-92-7340-01 | 0.224657534 | 0 | 5.181745536 | High groups |
| TCGA-92-7341-01 | 0.290410959 | 0 | 5.690217133 | High groups |
| TCGA-92-8063-01 | 0.334246575 | 0 | 6.01534511 | High groups |
| TCGA-92-8064-01 | 0.438356164 | 0 | 4.991109874 | Low groups |
| TCGA-92-8065-01 | 0.191780822 | 0 | 4.913427331 | Low groups |
| TCGA-94-7033-01 | 1.753424658 | 0 | 5.283357433 | High groups |
| TCGA-94-7557-01 | 0.01369863 | 0 | 5.330777261 | High groups |
| TCGA-94-7943-01 | 1.523287671 | 1 | 5.895355703 | High groups |
| TCGA-94-8035-01 | 0.334246575 | 0 | 4.555154277 | Low groups |
| TCGA-94-8490-01 | 0.419178082 | 0 | 4.562736367 | Low groups |
| TCGA-94-8491-01 | 1.756164384 | 1 | 4.695226287 | Low groups |
| TCGA-94-A4VJ-01 | 1.178082192 | 0 | 6.079819258 | High groups |
| TCGA-94-A5I4-01 | 0.860273973 | 1 | 3.944053404 | Low groups |
| TCGA-94-A5I6-01 | 1.317808219 | 1 | 4.400110864 | Low groups |
| TCGA-96-7544-01 | 5.917808219 | 0 | 4.946375616 | Low groups |
| TCGA-96-7545-01 | 4.638356164 | 1 | 4.813434133 | Low groups |
| TCGA-96-8169-01 | 1.526027397 | 0 | 5.216408534 | High groups |
| TCGA-96-8170-01 | 1.454794521 | 0 | 5.474189408 | High groups |
| TCGA-96-A4JK-01 | 1.61369863 | 0 | 3.967309734 | Low groups |
| TCGA-96-A4JL-01 | 2.306849315 | 0 | 4.050841392 | Low groups |
| TCGA-98-7454-01 | 1.78630137 | 0 | 5.389088151 | High groups |
| TCGA-98-8020-01 | 0.030136986 | 1 | 5.413542837 | High groups |
| TCGA-98-8021-01 | 2.515068493 | 1 | 5.556368714 | High groups |
| TCGA-98-8022-01 | 2.556164384 | 0 | 5.566694437 | High groups |
| TCGA-98-8023-01 | 1.778082192 | 0 | 4.24023204 | Low groups |
| TCGA-98-A538-01 | 2.263013699 | 0 | 4.655582928 | Low groups |
| TCGA-98-A539-01 | 0.473972603 | 1 | 4.156976588 | Low groups |
| TCGA-98-A53A-01 | 1.512328767 | 0 | 4.629369502 | Low groups |
| TCGA-98-A53B-01 | 0.167123288 | 0 | 5.17694831 | High groups |
| TCGA-98-A53C-01 | 2.252054795 | 0 | 5.037411392 | Low groups |
| TCGA-98-A53D-01 | 1.131506849 | 1 | 5.001600053 | Low groups |
| TCGA-98-A53H-01 | 1.169863014 | 1 | 4.557038003 | Low groups |
| TCGA-98-A53I-01 | 1.547945205 | 0 | 4.552683974 | Low groups |
| TCGA-98-A53J-01 | 1.726027397 | 0 | 4.277284275 | Low groups |
| TCGA-J1-A4AH-01 | 1.591780822 | 0 | 5.723728377 | High groups |
| TCGA-L3-A4E7-01 | 1.073972603 | 0 | 5.631945464 | High groups |
| TCGA-L3-A524-01 | 1.342465753 | 0 | 5.571289511 | High groups |
| TCGA-LA-A446-01 | 1.098630137 | 0 | 5.423166682 | High groups |
| TCGA-LA-A7SW-01 | 0.643835616 | 1 | 5.715217045 | High groups |
| TCGA-MF-A522-01 | 0.473972603 | 1 | 3.871323436 | Low groups |
| TCGA-NC-A5HD-01 | 0.005479452 | 0 | 5.938012026 | High groups |
| TCGA-NC-A5HE-01 | 6.4 | 0 | 4.322082888 | Low groups |
| TCGA-NC-A5HF-01 | 0.361643836 | 1 | 5.957173101 | High groups |
| TCGA-NC-A5HG-01 | 5.378082192 | 0 | 4.871107726 | Low groups |
| TCGA-NC-A5HH-01 | 0.101369863 | 0 | 6.331977523 | High groups |
| TCGA-NC-A5HI-01 | 0.191780822 | 1 | 4.741444065 | Low groups |
| TCGA-NC-A5HJ-01 | 0.898630137 | 1 | 5.857953584 | High groups |
| TCGA-NC-A5HK-01 | 0.350684932 | 0 | 4.652154034 | Low groups |
| TCGA-NC-A5HL-01 | 0.24109589 | 0 | 4.822782502 | Low groups |
| TCGA-NC-A5HM-01 | 3.320547945 | 0 | 7.054115876 | High groups |
| TCGA-NC-A5HN-01 | 4.106849315 | 0 | 5.516823662 | High groups |
| TCGA-NC-A5HO-01 | 3.660273973 | 0 | 4.261150911 | Low groups |
| TCGA-NC-A5HP-01 | 0.868493151 | 1 | 4.786142573 | Low groups |
| TCGA-NC-A5HQ-01 | 1.22739726 | 0 | 4.958968245 | Low groups |
| TCGA-NC-A5HR-01 | 3.408219178 | 0 | 5.074908698 | Low groups |
| TCGA-NC-A5HT-01 | 0.556164384 | 1 | 5.604028908 | High groups |
| TCGA-NK-A5CR-01 | 6.964383562 | 0 | 4.361398867 | Low groups |
| TCGA-NK-A5CT-01 | 5.452054795 | 1 | 4.634732943 | Low groups |
| TCGA-NK-A5CX-01 | 0.304109589 | 0 | 4.356827238 | Low groups |
| TCGA-NK-A5D1-01 | 0.41369863 | 1 | 7.371611307 | High groups |
| TCGA-NK-A7XE-01 | 0.035616438 | 0 | 5.211963731 | High groups |
| TCGA-O2-A52N-01 | 2.756164384 | 0 | 4.018958228 | Low groups |
| TCGA-O2-A52Q-01 | 0.238356164 | 1 | 3.924966668 | Low groups |
| TCGA-O2-A52S-01 | 0.673972603 | 1 | 5.123874947 | Low groups |
| TCGA-O2-A52V-01 | 1.890410959 | 1 | 3.979405119 | Low groups |
| TCGA-O2-A52W-01 | 0.715068493 | 0 | 5.020988337 | Low groups |
| TCGA-O2-A5IB-01 | 0.693150685 | 1 | 5.64051835 | High groups |
| TCGA-XC-AA0X-01 | 0.016438356 | 0 | 5.510081164 | High groups |

**Table S14.** Raw counts of RNA-sequencing data of *KLHL3* in LUSC from the TCGA.

| sampleID | time | Staus | RS | Label |
| --- | --- | --- | --- | --- |
| TCGA-94-7943-01 | 1.523287671 | 1 | 1.337739598 | Low groups |
| TCGA-68-8251-01 | 1.112328767 | 0 | 1.102862107 | Low groups |
| TCGA-33-A5GW-01 | 0.024657534 | 0 | 1.445135421 | High groups |
| TCGA-85-8070-01 | 2.630136986 | 0 | 1.083411213 | Low groups |
| TCGA-85-8479-01 | 1.282191781 | 0 | 0.253026298 | Low groups |
| TCGA-22-5482-01 | 0.978082192 | 0 | 0.975943657 | Low groups |
| TCGA-21-1077-01 | 1.764383562 | 1 | 1.857330232 | High groups |
| TCGA-43-6773-01 | 0.317808219 | 0 | 1.269734205 | Low groups |
| TCGA-34-8455-01 | 0.336986301 | 1 | 1.737231217 | High groups |
| TCGA-85-7950-01 | 1.578082192 | 0 | 1.993690056 | High groups |
| TCGA-60-2715-01 | 2.945205479 | 1 | 2.558723015 | High groups |
| TCGA-85-A5B5-01 | 0.304109589 | 0 | 0.842061335 | Low groups |
| TCGA-33-4583-01 | 12.60547945 | 0 | 1.923922615 | High groups |
| TCGA-60-2725-01 | 2.235616438 | 0 | 1.305123146 | Low groups |
| TCGA-85-8072-01 | 2.553424658 | 0 | 1.473168754 | High groups |
| TCGA-22-4601-01 | 1.438356164 | 1 | 1.568690267 | High groups |
| TCGA-66-2781-01 | 0.331506849 | 0 | 0.946988011 | Low groups |
| TCGA-22-0940-01 | 1.832876712 | 1 | 1.629354203 | High groups |
| TCGA-43-6143-01 | 1.915068493 | 0 | 1.585727211 | High groups |
| TCGA-39-5021-01 | 5.136986301 | 1 | 0.658943969 | Low groups |
| TCGA-22-1012-01 | 1.175342466 | 0 | 0.57001681 | Low groups |
| TCGA-33-AASL-01 | 0.408219178 | 1 | 1.473942298 | High groups |
| TCGA-33-4533-01 | 11.14520548 | 0 | 1.218250061 | Low groups |
| TCGA-43-6647-01 | 2.073972603 | 0 | 1.236371944 | Low groups |
| TCGA-77-6845-01 | 1.939726027 | 0 | 1.319111289 | Low groups |
| TCGA-56-8504-01 | 1.397260274 | 0 | 1.910161352 | High groups |
| TCGA-56-8629-01 | 1.317808219 | 0 | 1.130217214 | Low groups |
| TCGA-63-A5MY-01 | 2.882191781 | 0 | 1.264329258 | Low groups |
| TCGA-37-4130-01 | 0.676712329 | 0 | 0.443682903 | Low groups |
| TCGA-22-1002-01 | 0.35890411 | 0 | 2.045049603 | High groups |
| TCGA-18-3410-01 | 0.4 | 0 | 0.865475 | Low groups |
| TCGA-39-5011-01 | 1.547945205 | 1 | 0.812257148 | Low groups |
| TCGA-56-7580-01 | 2.534246575 | 0 | 1.136509747 | Low groups |
| TCGA-98-8023-01 | 1.778082192 | 0 | 1.7852711 | High groups |
| TCGA-33-4586-01 | 0.463013699 | 1 | 1.180155104 | Low groups |
| TCGA-58-8388-01 | 1.128767123 | 0 | 1.877906154 | High groups |
| TCGA-77-A5G3-01 | 12.52054795 | 0 | 2.129747543 | High groups |
| TCGA-85-8071-01 | 2.232876712 | 0 | 2.195098009 | High groups |
| TCGA-33-A4WN-01 | 0.391780822 | 0 | 1.114838774 | Low groups |
| TCGA-77-7141-01 | 0.04109589 | 0 | 1.729279255 | High groups |
| TCGA-60-2716-01 | 4.04109589 | 0 | 2.463032384 | High groups |
| TCGA-85-7844-01 | 2.495890411 | 0 | 2.203358861 | High groups |
| TCGA-98-A53H-01 | 1.169863014 | 1 | 2.39569283 | High groups |
| TCGA-37-A5EM-01 | 2.375342466 | 0 | 0.853847868 | Low groups |
| TCGA-63-A5MU-01 | 1.150684932 | 1 | 1.739014947 | High groups |
| TCGA-39-5016-01 | 10.54794521 | 0 | 1.752843668 | High groups |
| TCGA-33-4582-01 | 8.008219178 | 1 | 0.893543858 | Low groups |
| TCGA-85-8666-01 | 1.882191781 | 0 | 1.254542205 | Low groups |
| TCGA-66-2791-01 | 0.419178082 | 0 | 1.800991155 | High groups |
| TCGA-66-2782-01 | 0.832876712 | 1 | 1.982097018 | High groups |
| TCGA-39-5036-01 | 5.931506849 | 0 | 0.762262864 | Low groups |
| TCGA-NK-A5CX-01 | 0.304109589 | 0 | 1.732253726 | High groups |
| TCGA-77-8154-01 | 5.043835616 | 0 | 2.105800809 | High groups |
| TCGA-37-4132-01 | 0.621917808 | 0 | 1.286712646 | Low groups |
| TCGA-98-8020-01 | 0.030136986 | 1 | 0.851898456 | Low groups |
| TCGA-56-A5DS-01 | 0.021917808 | 0 | 2.070791673 | High groups |
| TCGA-60-2707-01 | 1.58630137 | 1 | 1.031190915 | Low groups |
| TCGA-66-2734-01 | 3.591780822 | 0 | 1.308099157 | Low groups |
| TCGA-66-2742-01 | 1.756164384 | 0 | 1.711573927 | High groups |
| TCGA-22-5481-01 | 0.947945205 | 1 | 0.377448514 | Low groups |
| TCGA-21-5787-01 | 0.282191781 | 1 | 0.566873271 | Low groups |
| TCGA-66-2783-01 | 2.079452055 | 0 | 0.672717338 | Low groups |
| TCGA-92-8063-01 | 0.334246575 | 0 | 1.921893074 | High groups |
| TCGA-66-2757-01 | 2.498630137 | 1 | 0.967034029 | Low groups |
| TCGA-39-5022-01 | 3.967123288 | 1 | 1.535179966 | High groups |
| TCGA-94-A5I6-01 | 1.317808219 | 1 | 2.001524454 | High groups |
| TCGA-85-7698-01 | 1.780821918 | 1 | 1.284153722 | Low groups |
| TCGA-37-4141-01 | 0.032876712 | 0 | 1.729283996 | High groups |
| TCGA-66-2753-01 | 0.084931507 | 0 | 1.21835528 | Low groups |
| TCGA-43-A474-01 | 0.967123288 | 0 | 1.565810802 | High groups |
| TCGA-56-8625-01 | 0.745205479 | 1 | 1.425791361 | High groups |
| TCGA-63-A5MB-01 | 8.556164384 | 0 | 1.362401227 | Low groups |
| TCGA-22-5471-01 | 0.62739726 | 1 | 0.775320491 | Low groups |
| TCGA-56-A4BX-01 | 1.109589041 | 0 | 1.756805882 | High groups |
| TCGA-63-7021-01 | 4.117808219 | 1 | 1.262127883 | Low groups |
| TCGA-85-A511-01 | 1.068493151 | 1 | 1.305082631 | Low groups |
| TCGA-66-2755-01 | 0.076712329 | 0 | 1.248400875 | Low groups |
| TCGA-46-3769-01 | 0.369863014 | 0 | 0.745944007 | Low groups |
| TCGA-21-1070-01 | 9.961643836 | 0 | 1.506977118 | High groups |
| TCGA-22-5477-01 | 1.4 | 1 | 2.384227924 | High groups |
| TCGA-18-3412-01 | 0.747945205 | 1 | 0.96154497 | Low groups |
| TCGA-34-5929-01 | 0.41369863 | 0 | 1.107040542 | Low groups |
| TCGA-NK-A5CT-01 | 5.452054795 | 1 | 1.820536794 | High groups |
| TCGA-46-6025-01 | 0.887671233 | 0 | 1.408118285 | Low groups |
| TCGA-NK-A7XE-01 | 0.035616438 | 0 | 1.407264831 | Low groups |
| TCGA-33-AASI-01 | 3.682191781 | 1 | 1.689083852 | High groups |
| TCGA-56-A4ZJ-01 | 1.753424658 | 0 | 1.56057016 | High groups |
| TCGA-34-5239-01 | 4.191780822 | 1 | 1.623945999 | High groups |
| TCGA-66-2754-01 | 0.167123288 | 0 | 2.114195299 | High groups |
| TCGA-66-2756-01 | 0.082191781 | 0 | 1.256405347 | Low groups |
| TCGA-58-8390-01 | 2.495890411 | 0 | 1.040468377 | Low groups |
| TCGA-77-A5GB-01 | 0.62739726 | 1 | 0.820221491 | Low groups |
| TCGA-O2-A52S-01 | 0.673972603 | 1 | 1.459687936 | High groups |
| TCGA-60-2713-01 | 3.715068493 | 1 | 1.360567079 | Low groups |
| TCGA-37-A5EN-01 | 1.808219178 | 0 | 1.856162148 | High groups |
| TCGA-56-8626-01 | 0.82739726 | 0 | 1.586206798 | High groups |
| TCGA-L3-A4E7-01 | 1.073972603 | 0 | 1.335313602 | Low groups |
| TCGA-22-5472-01 | 2.043835616 | 1 | 1.901192532 | High groups |
| TCGA-63-A5MW-01 | 4.490410959 | 0 | 1.731819025 | High groups |
| TCGA-63-A5M9-01 | 0 | 0 | 1.847131125 | High groups |
| TCGA-60-2710-01 | 5.545205479 | 0 | 1.22195492 | Low groups |
| TCGA-77-6843-01 | 6.093150685 | 0 | 2.491460941 | High groups |
| TCGA-21-5786-01 | 2.238356164 | 1 | 1.575749868 | High groups |
| TCGA-68-7755-01 | 0.22739726 | 0 | 0.943828483 | Low groups |
| TCGA-85-A50Z-01 | 1.265753425 | 1 | 0.548566936 | Low groups |
| TCGA-66-2737-01 | 0.167123288 | 0 | 1.956486588 | High groups |
| TCGA-NC-A5HK-01 | 0.350684932 | 0 | 0.412325614 | Low groups |
| TCGA-85-A4JC-01 | 1.501369863 | 1 | 1.4358067 | High groups |
| TCGA-58-A46J-01 | 7.093150685 | 0 | 1.459293888 | High groups |
| TCGA-46-6026-01 | 1.15890411 | 0 | 1.745313022 | High groups |
| TCGA-46-3765-01 | 1.109589041 | 0 | 0.849554077 | Low groups |
| TCGA-O2-A52V-01 | 1.890410959 | 1 | 1.848680236 | High groups |
| TCGA-92-7341-01 | 0.290410959 | 0 | 1.341129756 | Low groups |
| TCGA-51-4080-01 | 0.032876712 | 0 | 1.786179041 | High groups |
| TCGA-21-1076-01 | 4.315068493 | 1 | 2.194565796 | High groups |
| TCGA-56-7730-01 | 0.542465753 | 1 | 1.618403702 | High groups |
| TCGA-NC-A5HO-01 | 3.660273973 | 0 | 1.827429983 | High groups |
| TCGA-21-1079-01 | 0.868493151 | 1 | 1.059645682 | Low groups |
| TCGA-NC-A5HT-01 | 0.556164384 | 1 | 3.032207951 | High groups |
| TCGA-56-6545-01 | 1.824657534 | 0 | 1.887259624 | High groups |
| TCGA-85-8582-01 | 3.178082192 | 0 | 2.023300058 | High groups |
| TCGA-66-2787-01 | 3.334246575 | 0 | 1.649253283 | High groups |
| TCGA-60-2698-01 | 0.312328767 | 1 | 1.922317905 | High groups |
| TCGA-O2-A5IB-01 | 0.693150685 | 1 | 1.468338743 | High groups |
| TCGA-22-1000-01 | 0.720547945 | 1 | 1.721340639 | High groups |
| TCGA-56-8083-01 | 0.410958904 | 0 | 1.628622419 | High groups |
| TCGA-66-2763-01 | 0.082191781 | 0 | 0.965217669 | Low groups |
| TCGA-77-8153-01 | 5.457534247 | 0 | 1.225756041 | Low groups |
| TCGA-21-1083-01 | 3.602739726 | 0 | 1.925073062 | High groups |
| TCGA-85-8049-01 | 1.58630137 | 0 | 2.066434506 | High groups |
| TCGA-34-8456-01 | 2.202739726 | 0 | 2.0354223 | High groups |
| TCGA-33-6737-01 | 1.646575342 | 1 | 0.986926982 | Low groups |
| TCGA-77-8140-01 | 0.961643836 | 0 | 1.004763783 | Low groups |
| TCGA-39-5028-01 | 0.142465753 | 0 | 0.761617733 | Low groups |
| TCGA-34-5240-01 | 4.221917808 | 0 | 0.81418752 | Low groups |
| TCGA-56-8308-01 | 1.416438356 | 0 | 1.361422672 | Low groups |
| TCGA-90-7964-01 | 1.17260274 | 0 | 1.082417924 | Low groups |
| TCGA-18-3408-01 | 4.912328767 | 1 | 1.992886806 | High groups |
| TCGA-18-4721-01 | 12.86027397 | 0 | 1.078981805 | Low groups |
| TCGA-22-5478-01 | 0.065753425 | 0 | 1.152679655 | Low groups |
| TCGA-66-2773-01 | 0.252054795 | 0 | 1.878147287 | High groups |
| TCGA-22-5479-01 | 7.191780822 | 0 | 1.026700976 | Low groups |
| TCGA-39-5019-01 | 1.035616438 | 1 | 1.537927614 | High groups |
| TCGA-NK-A5CR-01 | 6.964383562 | 0 | 1.824262682 | High groups |
| TCGA-39-5040-01 | 1.210958904 | 1 | 0.819369988 | Low groups |
| TCGA-77-A5G6-01 | 1.073972603 | 1 | 1.445129535 | High groups |
| TCGA-98-A539-01 | 0.473972603 | 1 | 0.951971611 | Low groups |
| TCGA-85-6175-01 | 0.805479452 | 1 | 2.152392903 | High groups |
| TCGA-34-5927-01 | 0.671232877 | 1 | 1.604141423 | High groups |
| TCGA-85-8350-01 | 1.871232877 | 0 | 2.456343029 | High groups |
| TCGA-NC-A5HM-01 | 3.320547945 | 0 | 1.988639024 | High groups |
| TCGA-22-4591-01 | 1.21369863 | 1 | 0.827242879 | Low groups |
| TCGA-85-6561-01 | 3.353424658 | 0 | 1.373559267 | Low groups |
| TCGA-98-A53B-01 | 0.167123288 | 0 | 2.177203448 | High groups |
| TCGA-39-5039-01 | 1.490410959 | 0 | 0.756381923 | Low groups |
| TCGA-68-A59I-01 | 0.435616438 | 1 | 1.198146154 | Low groups |
| TCGA-90-7766-01 | 0.791780822 | 1 | 0.56348271 | Low groups |
| TCGA-66-2744-01 | 0.082191781 | 0 | 1.180410782 | Low groups |
| TCGA-22-4594-01 | 4.02739726 | 0 | 1.086591517 | Low groups |
| TCGA-77-A5GF-01 | 1.978082192 | 1 | 1.920874022 | High groups |
| TCGA-60-2711-01 | 3.452054795 | 0 | 1.462768106 | High groups |
| TCGA-33-4566-01 | 14.48493151 | 0 | 0.270794437 | Low groups |
| TCGA-43-2576-01 | 3.350684932 | 0 | 1.234297813 | Low groups |
| TCGA-37-4135-01 | 0.567123288 | 0 | 0.147304557 | Low groups |
| TCGA-98-7454-01 | 1.78630137 | 0 | 1.878624351 | High groups |
| TCGA-92-8065-01 | 0.191780822 | 0 | 1.576689663 | High groups |
| TCGA-52-7812-01 | 2.167123288 | 1 | 0.699996978 | Low groups |
| TCGA-63-A5MM-01 | 0.597260274 | 1 | 1.452032448 | High groups |
| TCGA-56-A5DR-01 | 0.010958904 | 0 | 0.450216558 | Low groups |
| TCGA-56-8082-01 | 1.246575342 | 0 | 2.36033719 | High groups |
| TCGA-98-A538-01 | 2.263013699 | 0 | 0.617672473 | Low groups |
| TCGA-94-8490-01 | 0.419178082 | 0 | 1.308351435 | Low groups |
| TCGA-52-7810-01 | 2.528767123 | 0 | 0.24875914 | Low groups |
| TCGA-NC-A5HJ-01 | 0.898630137 | 1 | 1.918377753 | High groups |
| TCGA-52-7809-01 | 0.454794521 | 1 | 2.084087508 | High groups |
| TCGA-NC-A5HN-01 | 4.106849315 | 0 | 1.265642389 | Low groups |
| TCGA-18-3414-01 | 1.961643836 | 0 | 1.206399393 | Low groups |
| TCGA-96-A4JL-01 | 2.306849315 | 0 | 1.826393569 | High groups |
| TCGA-18-5595-01 | 2.265753425 | 0 | 0.567605327 | Low groups |
| TCGA-77-7140-01 | 0.684931507 | 1 | 0.865831916 | Low groups |
| TCGA-33-AASB-01 | 0.082191781 | 1 | 0.768641834 | Low groups |
| TCGA-63-7022-01 | 5.679452055 | 0 | 1.12968741 | Low groups |
| TCGA-56-8628-01 | 1.687671233 | 0 | 2.640086577 | High groups |
| TCGA-NK-A5D1-01 | 0.41369863 | 1 | 2.242461829 | High groups |
| TCGA-NC-A5HH-01 | 0.101369863 | 0 | 2.409764702 | High groups |
| TCGA-77-8133-01 | 4.493150685 | 0 | 1.718294983 | High groups |
| TCGA-34-8454-01 | 3.232876712 | 0 | 2.083328308 | High groups |
| TCGA-90-6837-01 | 2.076712329 | 0 | 1.170067923 | Low groups |
| TCGA-70-6722-01 | 0.802739726 | 1 | 1.409666493 | High groups |
| TCGA-60-2712-01 | 0.750684932 | 0 | 0.989399103 | Low groups |
| TCGA-66-2727-01 | 1.41369863 | 0 | 0.951562483 | Low groups |
| TCGA-18-3409-01 | 6.276712329 | 1 | 2.604818398 | High groups |
| TCGA-77-8009-01 | 1.663013699 | 1 | 1.804701151 | High groups |
| TCGA-85-A50M-01 | 2.263013699 | 0 | 0.866858556 | Low groups |
| TCGA-37-4129-01 | 0.663013699 | 0 | 2.558814787 | High groups |
| TCGA-46-3766-01 | 1.01369863 | 0 | 1.484066676 | High groups |
| TCGA-37-5819-01 | 0.282191781 | 0 | 0.883034474 | Low groups |
| TCGA-60-2706-01 | 7.726027397 | 0 | 1.370337522 | Low groups |
| TCGA-85-A4JB-01 | 2.580821918 | 0 | 1.574973594 | High groups |
| TCGA-77-8007-01 | 0.380821918 | 1 | 1.184385942 | Low groups |
| TCGA-43-2581-01 | 3.221917808 | 0 | 1.124255267 | Low groups |
| TCGA-56-8309-01 | 1.17260274 | 0 | 2.197253388 | High groups |
| TCGA-43-A475-01 | 0.810958904 | 0 | 1.766123724 | High groups |
| TCGA-63-A5MV-01 | 3.01369863 | 0 | 2.453083539 | High groups |
| TCGA-22-1016-01 | 2.252054795 | 0 | 2.425922726 | High groups |
| TCGA-85-8288-01 | 1.101369863 | 0 | 1.225255354 | Low groups |
| TCGA-22-4613-01 | 0.980821918 | 0 | 1.104450802 | Low groups |
| TCGA-56-A62T-01 | 1.205479452 | 0 | 2.130314602 | High groups |
| TCGA-18-3411-01 | 9.797260274 | 0 | 0.658362925 | Low groups |
| TCGA-60-2721-01 | 2.693150685 | 0 | 1.041224636 | Low groups |
| TCGA-66-2771-01 | 1.583561644 | 0 | 0.627695337 | Low groups |
| TCGA-58-8387-01 | 1.104109589 | 0 | 0.766378464 | Low groups |
| TCGA-77-8148-01 | 5.542465753 | 0 | 1.031206074 | Low groups |
| TCGA-46-3768-01 | 0.819178082 | 0 | 1.093120152 | Low groups |
| TCGA-85-A4QQ-01 | 2.539726027 | 0 | 0.318515439 | Low groups |
| TCGA-43-7658-01 | 6.315068493 | 1 | 1.305875566 | Low groups |
| TCGA-56-7823-01 | 2.769863014 | 0 | 1.878370768 | High groups |
| TCGA-77-8138-01 | 0.791780822 | 1 | 0.841069044 | Low groups |
| TCGA-22-5489-01 | 1.17260274 | 1 | 1.156746425 | Low groups |
| TCGA-56-7822-01 | 0.824657534 | 1 | 1.159341784 | Low groups |
| TCGA-77-A5G8-01 | 5.161643836 | 0 | 2.012344989 | High groups |
| TCGA-18-5592-01 | 4.161643836 | 0 | 1.56928532 | High groups |
| TCGA-22-4595-01 | 0.684931507 | 1 | 1.476462903 | High groups |
| TCGA-37-3783-01 | 0.334246575 | 0 | 0.261256175 | Low groups |
| TCGA-77-7139-01 | 11.6739726 | 0 | 1.863134464 | High groups |
| TCGA-98-A53D-01 | 1.131506849 | 1 | 2.176967094 | High groups |
| TCGA-66-2758-01 | 1.750684932 | 0 | 1.243596173 | Low groups |
| TCGA-33-6738-01 | 5.279452055 | 0 | 0.750960165 | Low groups |
| TCGA-98-8021-01 | 2.515068493 | 1 | 1.954004034 | High groups |
| TCGA-56-1622-01 | 2.41369863 | 1 | 0.573696817 | Low groups |
| TCGA-90-7769-01 | 0.980821918 | 0 | 0.929688513 | Low groups |
| TCGA-85-8664-01 | 1.189041096 | 1 | 1.991544778 | High groups |
| TCGA-77-8008-01 | 7.230136986 | 0 | 1.558707957 | High groups |
| TCGA-58-8386-01 | 0.002739726 | 0 | 2.615001572 | High groups |
| TCGA-77-6844-01 | 4.898630137 | 1 | 1.670677873 | High groups |
| TCGA-66-2790-01 | 1.915068493 | 1 | 2.66541639 | High groups |
| TCGA-85-8352-01 | 0.350684932 | 1 | 1.593402966 | High groups |
| TCGA-77-8156-01 | 3.030136986 | 0 | 1.75574061 | High groups |
| TCGA-60-2696-01 | 0.298630137 | 0 | 0.888312242 | Low groups |
| TCGA-60-2703-01 | 4.81369863 | 1 | 1.036827915 | Low groups |
| TCGA-60-2714-01 | 4.194520548 | 0 | 0.941803702 | Low groups |
| TCGA-39-5031-01 | 5.043835616 | 0 | 1.413003596 | High groups |
| TCGA-60-2722-01 | 2.331506849 | 1 | 0.3809486 | Low groups |
| TCGA-NC-A5HR-01 | 3.408219178 | 0 | 1.305254362 | Low groups |
| TCGA-22-4605-01 | 2.668493151 | 0 | 1.455345265 | High groups |
| TCGA-43-6770-01 | 1.789041096 | 0 | 1.521508031 | High groups |
| TCGA-77-7138-01 | 0.931506849 | 0 | 0.907708117 | Low groups |
| TCGA-60-2704-01 | 1.457534247 | 1 | 2.354030771 | High groups |
| TCGA-77-8146-01 | 8.736986301 | 0 | 1.780572843 | High groups |
| TCGA-NC-A5HG-01 | 5.378082192 | 0 | 0.336477172 | Low groups |
| TCGA-63-6202-01 | 4.389041096 | 0 | 0.989191165 | Low groups |
| TCGA-63-A5MG-01 | 5.884931507 | 0 | 1.193934962 | Low groups |
| TCGA-22-1011-01 | 0.145205479 | 0 | 2.234643308 | High groups |
| TCGA-34-A5IX-01 | 2.824657534 | 0 | 0.584757678 | Low groups |
| TCGA-34-2608-01 | 2.739726027 | 0 | 0.999251025 | Low groups |
| TCGA-63-A5MI-01 | 4.887671233 | 0 | 1.135196186 | Low groups |
| TCGA-56-7582-01 | 1.646575342 | 0 | 2.053691702 | High groups |
| TCGA-33-4587-01 | 4.536986301 | 1 | 1.526539965 | High groups |
| TCGA-33-4538-01 | 8.161643836 | 0 | 1.775599087 | High groups |
| TCGA-43-A56U-01 | 1.183561644 | 0 | 1.311844846 | Low groups |
| TCGA-77-7142-01 | 6.101369863 | 0 | 1.604574392 | High groups |
| TCGA-77-7463-01 | 3.898630137 | 0 | 0.635976578 | Low groups |
| TCGA-77-8143-01 | 2.2 | 1 | 0.287613998 | Low groups |
| TCGA-21-5782-01 | 2.635616438 | 0 | 1.714560609 | High groups |
| TCGA-98-A53C-01 | 2.252054795 | 0 | 2.402152983 | High groups |
| TCGA-43-7656-01 | 1.632876712 | 0 | 1.539833189 | High groups |
| TCGA-21-1078-01 | 0.704109589 | 1 | 2.277263986 | High groups |
| TCGA-85-8048-01 | 2.095890411 | 0 | 2.007531432 | High groups |
| TCGA-LA-A7SW-01 | 0.643835616 | 1 | 1.792634733 | High groups |
| TCGA-56-8624-01 | 1.150684932 | 0 | 1.465041412 | High groups |
| TCGA-18-3407-01 | 0.37260274 | 0 | 1.476221184 | High groups |
| TCGA-18-4086-01 | 0.232876712 | 0 | 1.106334856 | Low groups |
| TCGA-34-5241-01 | 1.410958904 | 0 | 1.087252985 | Low groups |
| TCGA-90-A59Q-01 | 0.750684932 | 1 | 2.333808521 | High groups |
| TCGA-34-2596-01 | 0.219178082 | 0 | 1.784380896 | High groups |
| TCGA-85-8351-01 | 1.397260274 | 0 | 1.512315699 | High groups |
| TCGA-NC-A5HI-01 | 0.191780822 | 1 | 1.070580045 | Low groups |
| TCGA-22-5474-01 | 1.219178082 | 0 | 1.261984539 | Low groups |
| TCGA-66-2768-01 | 0.167123288 | 0 | 1.508090929 | High groups |
| TCGA-52-7622-01 | 2.361643836 | 0 | 1.029352392 | Low groups |
| TCGA-77-A5GH-01 | 3.238356164 | 0 | 1.379456911 | Low groups |
| TCGA-18-3417-01 | 3.005479452 | 0 | 0.383146766 | Low groups |
| TCGA-34-2600-01 | 5.134246575 | 0 | 1.309762187 | Low groups |
| TCGA-NC-A5HQ-01 | 1.22739726 | 0 | 2.3524994 | High groups |
| TCGA-66-2795-01 | 0.334246575 | 0 | 1.433193279 | High groups |
| TCGA-77-A5GA-01 | 3.506849315 | 0 | 1.863355296 | High groups |
| TCGA-63-7020-01 | 5.843835616 | 0 | 1.330635105 | Low groups |
| TCGA-63-A5MR-01 | 7.44109589 | 0 | 1.826913729 | High groups |
| TCGA-33-AAS8-01 | 3.052054795 | 0 | 2.058929866 | High groups |
| TCGA-77-8136-01 | 0.824657534 | 1 | 1.903707109 | High groups |
| TCGA-68-7757-01 | 0.578082192 | 0 | 2.018624284 | High groups |
| TCGA-39-5030-01 | 0.161643836 | 0 | 1.034671601 | Low groups |
| TCGA-22-A5C4-01 | 1.838356164 | 0 | 2.34981886 | High groups |
| TCGA-18-4083-01 | 0.515068493 | 0 | 3.027591649 | High groups |
| TCGA-56-A4BY-01 | 1.487671233 | 1 | 2.430361938 | High groups |
| TCGA-21-5783-01 | 7.342465753 | 0 | 2.441442693 | High groups |
| TCGA-68-8250-01 | 0.668493151 | 0 | 1.714244322 | High groups |
| TCGA-18-3419-01 | 7.701369863 | 0 | 0.252481073 | Low groups |
| TCGA-56-7579-01 | 0.408219178 | 1 | 0.633787731 | Low groups |
| TCGA-21-1075-01 | 5.846575342 | 0 | 1.872345844 | High groups |
| TCGA-66-2765-01 | 0.167123288 | 0 | 0.478631666 | Low groups |
| TCGA-66-2778-01 | 1.583561644 | 0 | 0.367886993 | Low groups |
| TCGA-56-8503-01 | 0.112328767 | 0 | 1.527448528 | High groups |
| TCGA-NC-A5HE-01 | 6.4 | 0 | 1.944639809 | High groups |
| TCGA-LA-A446-01 | 1.098630137 | 0 | 2.050040604 | High groups |
| TCGA-51-6867-01 | 2.15890411 | 1 | 1.800890011 | High groups |
| TCGA-96-A4JK-01 | 1.61369863 | 0 | 1.815996864 | High groups |
| TCGA-77-7338-01 | 0.01369863 | 0 | 1.469006778 | High groups |
| TCGA-85-7843-01 | 0.095890411 | 0 | 1.036600617 | Low groups |
| TCGA-77-8150-01 | 3.501369863 | 1 | 1.351445491 | Low groups |
| TCGA-46-3767-01 | 1.084931507 | 0 | 1.972552816 | High groups |
| TCGA-92-8064-01 | 0.438356164 | 0 | 0.864624253 | Low groups |
| TCGA-92-7340-01 | 0.224657534 | 0 | 0.989609206 | Low groups |
| TCGA-60-2697-01 | 0.547945205 | 1 | 1.583983219 | High groups |
| TCGA-43-6771-01 | 0.421917808 | 1 | 0.174606952 | Low groups |
| TCGA-85-6798-01 | 0.493150685 | 1 | 0.995620029 | Low groups |
| TCGA-NC-A5HF-01 | 0.361643836 | 1 | 2.094570836 | High groups |
| TCGA-77-8130-01 | 12.24931507 | 1 | 1.129406811 | Low groups |
| TCGA-43-2578-01 | 1.873972603 | 0 | 1.530914941 | High groups |
| TCGA-77-8145-01 | 0.37260274 | 1 | 1.616773865 | High groups |
| TCGA-39-5035-01 | 5.698630137 | 0 | 0.978996918 | Low groups |
| TCGA-58-8392-01 | 0.263013699 | 1 | 1.712474707 | High groups |
| TCGA-66-2786-01 | 2.164383562 | 0 | 1.152792015 | Low groups |
| TCGA-68-7756-01 | 0.553424658 | 0 | 1.831330759 | High groups |
| TCGA-33-4589-01 | 0.128767123 | 0 | 0.017829581 | Low groups |
| TCGA-22-0944-01 | 0.610958904 | 0 | 0.758257454 | Low groups |
| TCGA-98-A53J-01 | 1.726027397 | 0 | 2.092397712 | High groups |
| TCGA-70-6723-01 | 0.854794521 | 1 | 2.033347443 | High groups |
| TCGA-43-7657-01 | 0.646575342 | 0 | 1.587477875 | High groups |
| TCGA-63-A5MT-01 | 1.169863014 | 1 | 1.034103664 | Low groups |
| TCGA-NC-A5HP-01 | 0.868493151 | 1 | 2.422831078 | High groups |
| TCGA-39-5024-01 | 6.876712329 | 0 | 1.200250094 | Low groups |
| TCGA-63-7023-01 | 4.608219178 | 0 | 1.086046148 | Low groups |
| TCGA-J1-A4AH-01 | 1.591780822 | 0 | 1.329811377 | Low groups |
| TCGA-85-8355-01 | 0.167123288 | 0 | 2.142822227 | High groups |
| TCGA-96-7545-01 | 4.638356164 | 1 | 1.695892545 | High groups |
| TCGA-85-8353-01 | 0.257534247 | 1 | 1.48001327 | High groups |
| TCGA-58-A46N-01 | 1.726027397 | 1 | 2.46109042 | High groups |
| TCGA-22-1017-01 | 2.476712329 | 1 | 0.736661313 | Low groups |
| TCGA-XC-AA0X-01 | 0.016438356 | 0 | 1.268182478 | Low groups |
| TCGA-94-A5I4-01 | 0.860273973 | 1 | 1.434270084 | High groups |
| TCGA-85-8354-01 | 2.726027397 | 0 | 1.01441831 | Low groups |
| TCGA-22-5483-01 | 1.556164384 | 1 | 1.526359345 | High groups |
| TCGA-90-A4EE-01 | 1.884931507 | 0 | 1.862667551 | High groups |
| TCGA-34-5231-01 | 5.435616438 | 0 | 1.051988572 | Low groups |
| TCGA-56-8623-01 | 1.767123288 | 1 | 1.47667964 | High groups |
| TCGA-66-2759-01 | 2.087671233 | 0 | 1.006598558 | Low groups |
| TCGA-66-2770-01 | 1.917808219 | 0 | 2.253727901 | High groups |
| TCGA-56-8307-01 | 2.24109589 | 0 | 2.196581642 | High groups |
| TCGA-43-3394-01 | 1.512328767 | 1 | 1.650209796 | High groups |
| TCGA-60-2695-01 | 1.75890411 | 0 | 0.781566066 | Low groups |
| TCGA-22-4593-01 | 2.923287671 | 0 | 1.980000385 | High groups |
| TCGA-56-7222-01 | 1.169863014 | 1 | 0.449043597 | Low groups |
| TCGA-22-5491-01 | 4.693150685 | 0 | 0.982322282 | Low groups |
| TCGA-98-A53A-01 | 1.512328767 | 0 | 2.191298525 | High groups |
| TCGA-85-A4PA-01 | 2.030136986 | 0 | 3.229331729 | High groups |
| TCGA-21-1080-01 | 10.20273973 | 0 | 2.264972531 | High groups |
| TCGA-18-3406-01 | 0.978082192 | 1 | 0.7810592 | Low groups |
| TCGA-58-8391-01 | 5.569863014 | 1 | 1.712336507 | High groups |
| TCGA-85-7696-01 | 3.043835616 | 0 | 2.365088427 | High groups |
| TCGA-22-5480-01 | 4.873972603 | 1 | 0.77034776 | Low groups |
| TCGA-43-8116-01 | 0.980821918 | 0 | 1.531195818 | High groups |
| TCGA-22-4607-01 | 1.608219178 | 0 | 0.860324073 | Low groups |
| TCGA-60-2726-01 | 0.980821918 | 0 | 1.739139801 | High groups |
| TCGA-85-7699-01 | 2.082191781 | 1 | 0.860220281 | Low groups |
| TCGA-34-5236-01 | 0.309589041 | 1 | 0.704673799 | Low groups |
| TCGA-63-A5ML-01 | 3.797260274 | 0 | 1.643352066 | High groups |
| TCGA-33-AASJ-01 | 3.109589041 | 1 | 0.928304818 | Low groups |
| TCGA-56-5898-01 | 1.520547945 | 0 | 0.821462738 | Low groups |
| TCGA-56-8622-01 | 0.150684932 | 0 | 1.02674697 | Low groups |
| TCGA-66-2800-01 | 4.087671233 | 0 | 1.039519127 | Low groups |
| TCGA-77-7335-01 | 5.843835616 | 0 | 0.701236593 | Low groups |
| TCGA-43-8118-01 | 0.243835616 | 0 | 1.790576238 | High groups |
| TCGA-22-4596-01 | 0.046575342 | 0 | 1.314967835 | Low groups |
| TCGA-56-7731-01 | 0.008219178 | 0 | 0.891821079 | Low groups |
| TCGA-56-8304-01 | 0.290410959 | 0 | 0.374624371 | Low groups |
| TCGA-85-A53L-01 | 1.032876712 | 0 | 1.859304404 | High groups |
| TCGA-52-7811-01 | 0.419178082 | 1 | 1.225495329 | Low groups |
| TCGA-85-7710-01 | 0.115068493 | 0 | 1.894501466 | High groups |
| TCGA-85-8481-01 | 0.646575342 | 0 | 0.826766533 | Low groups |
| TCGA-85-A512-01 | 1.273972603 | 0 | 0.489444591 | Low groups |
| TCGA-56-7221-01 | 1.665753425 | 0 | 0.669283096 | Low groups |
| TCGA-22-5485-01 | 2.301369863 | 1 | 1.222337505 | Low groups |
| TCGA-56-8305-01 | 0.287671233 | 0 | 1.342663427 | Low groups |
| TCGA-66-2789-01 | 0.336986301 | 0 | 1.907746729 | High groups |
| TCGA-96-8169-01 | 1.526027397 | 0 | 1.131314072 | Low groups |
| TCGA-63-A5MP-01 | 1.4 | 1 | 2.145572366 | High groups |
| TCGA-85-8584-01 | 1.049315068 | 0 | 1.538041961 | High groups |
| TCGA-63-A5MJ-01 | 4.997260274 | 0 | 1.552180768 | High groups |
| TCGA-94-7557-01 | 0.01369863 | 0 | 1.81053611 | High groups |
| TCGA-85-8287-01 | 0.063013699 | 0 | 1.510908742 | High groups |
| TCGA-21-A5DI-01 | 2.682191781 | 0 | 1.19640186 | Low groups |
| TCGA-60-2719-01 | 3.553424658 | 0 | 1.041476454 | Low groups |
| TCGA-56-7223-01 | 0.361643836 | 1 | 2.977360165 | High groups |
| TCGA-33-4532-01 | 10.75068493 | 0 | 1.681294648 | High groups |
| TCGA-85-A510-01 | 0.994520548 | 1 | 3.33630026 | High groups |
| TCGA-37-3792-01 | 0.032876712 | 0 | 0.663480536 | Low groups |
| TCGA-34-7107-01 | 0.093150685 | 0 | 1.907109369 | High groups |
| TCGA-77-8144-01 | 2.282191781 | 0 | 0.895239167 | Low groups |
| TCGA-22-4609-01 | 0.797260274 | 0 | 1.6896118 | High groups |
| TCGA-O2-A52Q-01 | 0.238356164 | 1 | 1.425027642 | High groups |
| TCGA-43-8115-01 | 1.115068493 | 0 | 2.178552073 | High groups |
| TCGA-77-8128-01 | 3.150684932 | 0 | 1.292418685 | Low groups |
| TCGA-94-7033-01 | 1.753424658 | 0 | 1.136999641 | Low groups |
| TCGA-63-A5MH-01 | 5.550684932 | 0 | 1.542900909 | High groups |
| TCGA-66-2780-01 | 1.002739726 | 0 | 1.00402764 | Low groups |
| TCGA-85-7697-01 | 2.912328767 | 0 | 1.424754138 | High groups |
| TCGA-56-A4BW-01 | 1.602739726 | 0 | 2.26495835 | High groups |
| TCGA-85-A4CN-01 | 2.843835616 | 0 | 1.498442337 | High groups |
| TCGA-96-8170-01 | 1.454794521 | 0 | 1.959056425 | High groups |
| TCGA-94-A4VJ-01 | 1.178082192 | 0 | 1.19750072 | Low groups |
| TCGA-22-4604-01 | 0.838356164 | 1 | 1.426732055 | High groups |
| TCGA-58-A46L-01 | 4.720547945 | 0 | 1.144653188 | Low groups |
| TCGA-85-8580-01 | 3.049315068 | 0 | 1.503242818 | High groups |
| TCGA-21-5784-01 | 3.473972603 | 0 | 0.815121892 | Low groups |
| TCGA-NC-A5HL-01 | 0.24109589 | 0 | 2.084413173 | High groups |
| TCGA-90-A4ED-01 | 1.684931507 | 0 | 2.338378295 | High groups |
| TCGA-68-A59J-01 | 1.22739726 | 0 | 1.123240753 | Low groups |
| TCGA-60-2720-01 | 0.265753425 | 0 | 0.333050268 | Low groups |
| TCGA-94-8491-01 | 1.756164384 | 1 | 1.019383457 | Low groups |
| TCGA-66-2793-01 | 0.838356164 | 0 | 1.768210378 | High groups |
| TCGA-77-A5FZ-01 | 10.51506849 | 0 | 1.544082209 | High groups |
| TCGA-21-1081-01 | 0.778082192 | 1 | 1.374803489 | Low groups |
| TCGA-43-3920-01 | 2.75890411 | 0 | 1.092052879 | Low groups |
| TCGA-37-A5EL-01 | 2.591780822 | 1 | 1.12706233 | Low groups |
| TCGA-37-3789-01 | 0.035616438 | 0 | 0.73613403 | Low groups |
| TCGA-60-2724-01 | 1.964383562 | 0 | 1.080514878 | Low groups |
| TCGA-77-7337-01 | 8.912328767 | 0 | 1.066910838 | Low groups |
| TCGA-85-8276-01 | 2.871232877 | 1 | 0.955840828 | Low groups |
| TCGA-98-8022-01 | 2.556164384 | 0 | 1.643164555 | High groups |
| TCGA-66-2766-01 | 0.084931507 | 0 | 0.948713028 | Low groups |
| TCGA-66-2792-01 | 2.501369863 | 0 | 2.220183953 | High groups |
| TCGA-56-5897-01 | 1.035616438 | 0 | 1.390502676 | Low groups |
| TCGA-85-8277-01 | 0.84109589 | 0 | 1.297048985 | Low groups |
| TCGA-39-5034-01 | 0.964383562 | 1 | 1.555571628 | High groups |
| TCGA-22-5473-01 | 4.534246575 | 1 | 1.55196715 | High groups |
| TCGA-66-2767-01 | 0.167123288 | 0 | 1.236673575 | Low groups |
| TCGA-37-4133-01 | 0.652054795 | 0 | 0.63527271 | Low groups |
| TCGA-56-A49D-01 | 1.745205479 | 0 | 1.17868863 | Low groups |
| TCGA-60-2723-01 | 2.991780822 | 0 | 0.321381198 | Low groups |
| TCGA-94-8035-01 | 0.334246575 | 0 | 0.954095076 | Low groups |
| TCGA-18-3421-01 | 7.246575342 | 0 | 0.27464008 | Low groups |
| TCGA-22-4599-01 | 3.180821918 | 0 | 1.581951202 | High groups |
| TCGA-63-A5MS-01 | 6.523287671 | 0 | 1.66881373 | High groups |
| TCGA-56-8201-01 | 1.087671233 | 1 | 1.229019921 | Low groups |
| TCGA-85-8052-01 | 2.010958904 | 0 | 1.788907645 | High groups |
| TCGA-22-5492-01 | 1.38630137 | 0 | 1.205797579 | Low groups |
| TCGA-O2-A52N-01 | 2.756164384 | 0 | 1.839100169 | High groups |
| TCGA-34-5234-01 | 6.221917808 | 0 | 0.574534922 | Low groups |
| TCGA-98-A53I-01 | 1.547945205 | 0 | 0.880396921 | Low groups |
| TCGA-66-2785-01 | 0.164383562 | 0 | 1.097844411 | Low groups |
| TCGA-63-A5MN-01 | 0.876712329 | 1 | 2.385055967 | High groups |
| TCGA-85-A4QR-01 | 1.643835616 | 0 | 2.316775838 | High groups |
| TCGA-39-5027-01 | 8.44109589 | 1 | 1.962696796 | High groups |
| TCGA-66-2788-01 | 1.915068493 | 0 | 1.212356307 | Low groups |
| TCGA-21-1072-01 | 8.263013699 | 0 | 1.947707323 | High groups |
| TCGA-51-4081-01 | 2.495890411 | 0 | 1.857525875 | High groups |
| TCGA-22-1005-01 | 5.350684932 | 0 | 1.355830639 | Low groups |
| TCGA-L3-A524-01 | 1.342465753 | 0 | 0.939433123 | Low groups |
| TCGA-O2-A52W-01 | 0.715068493 | 0 | 0.661004469 | Low groups |
| TCGA-60-2708-01 | 6.704109589 | 0 | 1.338002417 | Low groups |
| TCGA-85-A513-01 | 2.493150685 | 0 | 0.951794284 | Low groups |
| TCGA-77-7465-01 | 2.712328767 | 0 | 0.945989994 | Low groups |
| TCGA-58-8393-01 | 2.898630137 | 0 | 1.540353614 | High groups |
| TCGA-77-A5G1-01 | 6.019178082 | 1 | 0.704111798 | Low groups |
| TCGA-34-5232-01 | 6.769863014 | 0 | 1.603412603 | High groups |
| TCGA-60-2709-01 | 4.123287671 | 0 | 1.477331977 | High groups |
| TCGA-43-A56V-01 | 0.97260274 | 1 | 1.39573626 | Low groups |
| TCGA-34-5928-01 | 3.276712329 | 0 | 1.076157079 | Low groups |
| TCGA-56-A4ZK-01 | 1.561643836 | 0 | 1.871522672 | High groups |
| TCGA-39-5037-01 | 4.630136986 | 0 | 0.964647284 | Low groups |
| TCGA-77-8131-01 | 1.049315068 | 0 | 1.071731745 | Low groups |
| TCGA-77-6842-01 | 0.550684932 | 1 | 2.14884416 | High groups |
| TCGA-96-7544-01 | 5.917808219 | 0 | 1.567501898 | High groups |
| TCGA-18-3415-01 | 7.679452055 | 0 | 0.901346409 | Low groups |
| TCGA-66-2794-01 | 4.506849315 | 0 | 1.372131468 | Low groups |
| TCGA-90-7767-01 | 0.243835616 | 0 | 1.409750566 | High groups |
| TCGA-85-6560-01 | 3.449315068 | 0 | 1.697146279 | High groups |
| TCGA-58-A46M-01 | 2.936986301 | 0 | 2.057891434 | High groups |
| TCGA-66-2777-01 | 0.167123288 | 0 | 1.419168733 | High groups |
| TCGA-77-8139-01 | 8.673972603 | 0 | 1.235229334 | Low groups |
| TCGA-58-A46K-01 | 2.01369863 | 1 | 2.548519764 | High groups |
| TCGA-77-A5G7-01 | 0.493150685 | 0 | 0.548850116 | Low groups |
| TCGA-85-A4CL-01 | 2.523287671 | 0 | 1.440465922 | High groups |
| TCGA-18-3416-01 | 2.575342466 | 1 | 2.354126106 | High groups |
| TCGA-43-5668-01 | 1.287671233 | 1 | 0.770781931 | Low groups |
| TCGA-43-5670-01 | 2.326027397 | 0 | 0.481995039 | Low groups |
| TCGA-21-1082-01 | 9.983561644 | 0 | 1.206943526 | Low groups |
| TCGA-NC-A5HD-01 | 0.005479452 | 0 | 2.972692285 | High groups |
| TCGA-66-2769-01 | 0.589041096 | 0 | 1.131678034 | Low groups |
| TCGA-33-4547-01 | 6.62739726 | 0 | 1.643326121 | High groups |
| TCGA-21-1071-01 | 2.720547945 | 1 | 2.725131013 | High groups |
| TCGA-33-AASD-01 | 5.238356164 | 1 | 1.147659772 | Low groups |
| TCGA-39-5029-01 | 1.345205479 | 1 | 0.52015766 | Low groups |
| TCGA-MF-A522-01 | 0.473972603 | 1 | 0.105316221 | Low groups |
| TCGA-51-4079-01 | 0.032876712 | 0 | 0.383540218 | Low groups |

**Table S15:** Clinicopathological characteristics of the TCGA cohort of NSCLC.

|  |  | LUAD | | LUSC | |
| --- | --- | --- | --- | --- | --- |
|  |  | Number | Percentage | Number | Percentage |
| Total |  | 504 | 100% | 473 | 100% |
| Age |  |  |  |  |  |
|  | ≤65 | 237 | 47.02% | 178 | 37.63% |
|  | ＞65 | 257 | 51.00% | 290 | 61.31% |
|  | unknow | 10 | 1.98% | 5 | 1..06% |
| Gender |  |  |  |  |  |
|  | Female | 269 | 53.37% | 123 | 26.00% |
|  | Male | 235 | 46.63% | 350 | 74.00% |
| Stage |  |  |  |  |  |
|  | Stage I | 273 | 54.17% | 228 | 48.20% |
|  | Stage II | 119 | 23.61% | 156 | 32.98% |
|  | Stage III | 81 | 16.07% | 79 | 16.70% |
|  | Stage IV | 24 | 4.76% | 6 | 1.27% |
|  | unknow | 7 | 1.39% | 4 | 0.85% |
| Tumor(T) |  |  |  |  |  |
|  | T1 | 169 | 33.53% | 105 | 22.20% |
|  | T2 | 268 | 53.17% | 278 | 58.77% |
|  | T3 | 45 | 8.93% | 69 | 14.59% |
|  | T4 | 19 | 3.77% | 21 | 4.44% |
|  | TX | 3 | 0.60% | 0 | 0 |
| Lymph Node(N) |  |  |  |  |  |
|  | N0 | 325 | 64.48% | 297 | 62.79% |
|  | N1 | 94 | 18.65% | 126 | 26.64% |
|  | N2 | 71 | 14.09% | 40 | 8.46% |
|  | N3 | 2 | 0.40% | 4 | 0.85% |
|  | NX | 12 | 2.38% | 6 | 1.26% |
| Metastasis(M) |  |  |  |  |  |
|  | M0 | 337 | 66.87% | 387 | 81.82% |
|  | M1 | 23 | 4.56% | 6 | 1.26% |
|  | MX | 140 | 27.78% | 76 | 16.07% |
|  | unknow | 4 | 0.79% | 4 | 0.85% |

**Table S16.** The immunohistochemical score of KLHL3 in LUAD patients.

| The immunohistochemical score of KLHL3 in LUAD patients. | | | | |
| --- | --- | --- | --- | --- |
| id | Age | Intensity | Quantity | I×Q |
| 1249 | 44 | 0 | 0 | 0 |
| 1303 | 68 | 3 | 50 | 150 |
| 1327 | 64 | 0 | 0 | 0 |
| 406 | 48 | 0 | 0 | 0 |
| 970 | 76 | 3 | 25 | 75 |
| 4855 | 74 | 2 | 25 | 50 |
| 2663 | 74 | 0 | 0 | 0 |
| 447 | 62 | 1 | 25 | 25 |

**Table S17.** The immunohistochemical score of KLHL3 in LUSC patients.

| The immunohistochemical score of KLHL3 in LUSC patients. | | | | |
| --- | --- | --- | --- | --- |
| id | Age | Intensity | Quantity | I×Q |
| 4890 | 60 | 0 | 0 | 0 |
| 4898 | 76 | 0 | 0 | 0 |
| 376 | 64 | 0 | 0 | 0 |
| 4128 | 66 | 0 | 0 | 0 |

**Table S18.** The immunohistochemical score of KLHL3 in normal group.

| The immunohistochemical score of KLHL3 in normal group. | | | | |
| --- | --- | --- | --- | --- |
| id | Age | Intensity | Quantity | I×Q |
| 1678 | 57 | 1 | 50 | 50 |
| 2208 | 67 | 1 | 50 | 50 |
| 1470 | 65 | 1 | 50 | 50 |
